# Genetic and functional characterization of disease associations explains comorbidity

**DOI:** 10.1038/s41598-017-04939-4

**Published:** 2017-07-24

**Authors:** Carlota Rubio-Perez, Emre Guney, Daniel Aguilar, Janet Piñero, Javier Garcia-Garcia, Barbara Iadarola, Ferran Sanz, Narcís Fernandez-Fuentes, Laura I. Furlong, Baldo Oliva

**Affiliations:** 1 Joint IRB-BSC-CRG Program in Computational Biology, Institute for Research in Biomedicine (IRB Barcelona), 08028 Barcelona, Spain; 2 Structural Bioinformatics Group, GRIB, IMIM, Department of Experimental and Life Sciences, Universitat Pompeu Fabra, 08003, Barcelona, Catalonia Spain; 3 Center for Complex Network Research and Department of Physics, Northeastern University, Boston, 02115 MA USA; 4 Barcelona Institute for Global Health (ISGlobal), 08003 Barcelona, Catalonia Spain; 5 Integrative Biomedical Informatics Group, GRIB, IMIM, Department of Experimental and Life Sciences, Universitat Pompeu Fabra, Barcelona, 08003 Catalonia Spain; 6 Institute of Biological, Environmental and Rural Sciences, Aberystwyth University, Aberystwyth, SY23 3EB United Kingdom

## Abstract

Understanding relationships between diseases, such as comorbidities, has important socio-economic implications, ranging from clinical study design to health care planning. Most studies characterize disease comorbidity using shared genetic origins, ignoring pathway-based commonalities between diseases. In this study, we define the disease pathways using an interactome-based extension of known disease-genes and introduce several measures of functional overlap. The analysis reveals 206 significant links among 94 diseases, giving rise to a highly clustered disease association network. We observe that around 95% of the links in the disease network, though not identified by genetic overlap, are discovered by functional overlap. This disease network portraits rheumatoid arthritis, asthma, atherosclerosis, pulmonary diseases and Crohn’s disease as hubs and thus pointing to common inflammatory processes underlying disease pathophysiology. We identify several described associations such as the inverse comorbidity relationship between Alzheimer’s disease and neoplasms. Furthermore, we investigate the disruptions in protein interactions by mapping mutations onto the domains involved in the interaction, suggesting hypotheses on the causal link between diseases. Finally, we provide several proof-of-principle examples in which we model the effect of the mutation and the change of the association strength, which could explain the observed comorbidity between diseases caused by the same genetic alterations.

## Introduction

Comorbidity is the co-occurrence of two or more diseases in the same patient. It has been proposed that in some cases, comorbidities can be explained by shared genetic components such as disease-associated genes or biological pathways. Thus, disease comorbidity could be studied through shared disease-associated genes. In this sense, recent studies have provided insights on comorbidity patterns among patients^1–4^. They found that many pairs of diseases sharing genes fail to show significant comorbidity^5, 6^ and *vice versa*: comorbid diseases may not have a common genetic component^7^. As gene products rarely act in isolation, we need to consider the interactions between the disease-associated gene products to fully understand comorbidities between diseases. For instance, there are diseases that may be linked through the interaction of two proteins, each associated with a different disease. Thus, although there may be no genes in common, comorbidity relationships can be governed by protein-protein interactions^8, 9^.

On one hand, protein interaction networks (PIN), or interactomes, have helped extending our view of the causes, sometimes genetic, of common diseases^10, 11^. To this end, we have developed BIANA, a platform that integrates protein-protein interaction data from various publicly available resources^12^. Through the use of BIANA in a previous study, we found that the integration of protein interaction sources could highlight the well-known comorbidity between diabetes type 2 and Alzheimer’s disease^13, 14^, simply by quantifying the overlap in the direct neighbourhood of the disease genes^12^. More recently, Menche and co-workers showed that, albeit incomplete, the current view of the human interactome provides enough information to uncover molecular mechanisms of related diseases, including comorbidities^15^. They calculated the shortest distance between proteins associated to each disease (measuring inter-disease and intra-disease pairs of proteins) and defined the network-based separation (named Sab) to establish a quantitative measure of disease-disease relationships. Therefore, approaches that integrate different aspects of cellular networks (i.e. protein-protein interactions, metabolic relationships and signalling pathways) and disease information are required to better understand the molecular basis of comorbidity^5, 15^.

On the other hand, several network-based methods have been developed to expand our incomplete knowledge of the disease-associated interactome. Based on the “guilt-by-association” principle suggesting that genes that interact with each other tend to exhibit similar functions, these methods prioritize genes for their association to a given disease by leveraging their connectedness to known disease-associated genes in the interactome^16, 17^. The interactome also provides a powerful framework for understanding the mechanisms underlying diseases through the analysis of the interfaces of protein-protein interactions involved in the modules associated with the diseases^18, 19^ and for characterizing edgetic disease-disease associations^8, 9^.

In this study, we have extended our knowledge about disease comorbidity by: i) integrating data from several protein interaction databases to uncover latent connections between diseases; and ii) analysing their common disease-associated genes and their functions. Firstly, we have used BIANA^12^ to integrate several databases into a protein interaction network (PIN). Secondly, we have used the guilt-by-association principle, applying the Genes Underlying Inheritance Disorders (GUILD) method^16, 20^, to extend the number of genes associated to each disease in the network and assuage the incompleteness of gene-disease associations. Thirdly, to consider gene pleiotropy, in which different genomic alterations in the same gene can have different functional impact and give rise to different pathophenotypes^21, 22^, we have incorporated functional information. Given that comorbidity does not only arise from common genes involved in diseases but also from shared disease-pathways^7^, we have used disease-disease functional associations to characterize potential comorbidity links. Adding a functional view can occasionally be more specific for the association of two diseases than sharing a gene. Finally, we have automatically gathered structural information of the interfaces of protein interactions associated with potential comorbidities to support the prediction of disease-disease relationships.

## Results

### Characterization of disease-disease associations using genetic and functional measures

**Figure 1:**
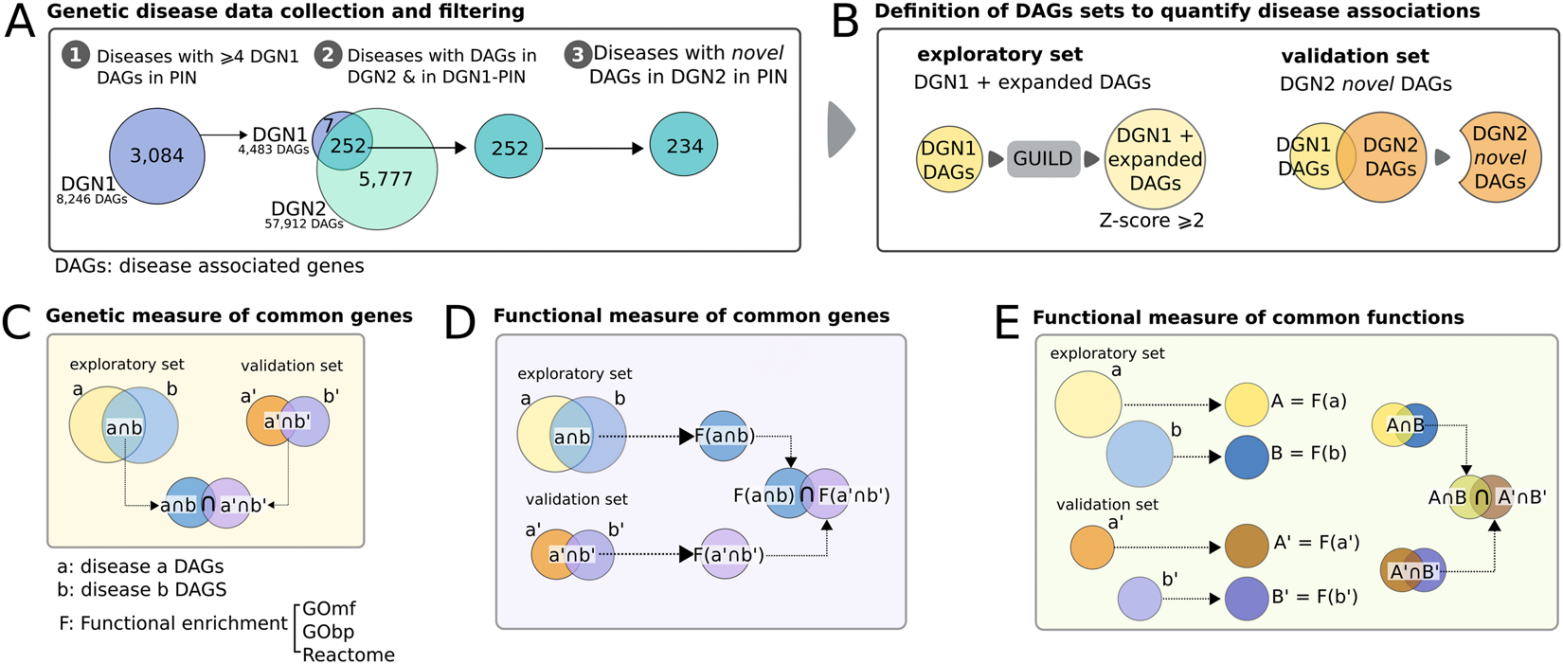
Schema of data collection and methods applied. (**A**) Disease data collection and filtering (left panel). Venn diagrams show the number of diseases in DGN1 and DGN2, for the diseases having at least four disease-associated genes (DAG). (**B**) Schematic representation of the exploratory and validation sets. Methods to quantify disease relationships: (**C**) Genetic score of common genes: a′ ∩ b′ and a ∩ b are the common DAGs of diseases “a” and “b” in the validation and exploratory sets, respectively. The intersection of both sets is used to compute the p-value with respect to a background of genes. (**D**) Functional score of common genes: a′ ∩ b′ and a ∩ b are defined as before, while F(a ∩ b) and F(a′ ∩ b′) are the sets of functions enriched with genes in a ∩ b and a′ ∩ b′, respectively. The set of significant common enriched-functions (after multiple testing correction) is used to compute the p-value of this relationship with respect to a background set of functions. (**E**) Functional score of common functions: We first find the set of functions enriched among the DAGs of diseases “a” and “b” in the exploratory (**A** and **B**) and validation (A′ and B′) sets;. Next, we extract the set of common functions of diseases “a” and “b”, A ∩ B and A′ ∩ B′ respectively. The final p-value of the relationship is then calculated with the set of significant common enriched-functions (after multiple testing correction) with respect to a background set of functions. A multiple testing correction is applied after computing final p-values of scoring methods introduced in C to E (see Methods).

To understand the common mechanisms underlying disease comorbidity, we start with 3,084 diseases annotated in DisGeNET v1.0 database (DGN1) and investigate the genetic overlap among all possible 4,753,986 disease associations (Fig. 1A). Only 13,064 disease-disease associations (DDAs) (0.0027%) have at least one gene in common and most of these pairs (97.8%) share 3 or less genes (Figure S1). To assess the significance of the genetic overlap of these DDAs we use one-sided Fisher’s exact test (see methods for details) and we find 11,395 out of 13.064 pairs with significant overlap (P-value < 0.05) (Figure S2). When corrected for multiple hypothesis testing using Bonferroni method, the percentage of the significant DDAs among all potential disease pairs is even lower (≪0.001%), highlighting limitations of using common genes to characterize relationships between diseases.

The low number of the significant DDAs can be attributed to the incompleteness of the current knowledge of disease-associated genes^15, 23, 24^. To overcome this hurdle, we expand the information on the genes associated to a disease using interactions of known disease-associated genes (seeds) in the human interactome. For this expansion, we use GUILD, a network-based disease-gene prioritization tool to identify genes that are likely to participate in the disease-related biological processes based on their topological closeness in the interactome to the seeds)^16, 20^. We define seeds as the disease-associated genes in DGN1 for the diseases that have at least 4 proteins in the interactome, accounting for 259 diseases (Fig. 1A). Using only these diseases from DGN1, we find 2,494 significant DDAs based on seed overlap. Then, after GUILD expansion, we assess the significance of the genetic overlap between them (Figure S2A, Supplementary Methods) and the number of significant DDAs increases to 8,012 (Figure S2C). Moreover, we demonstrate that network-based prioritization of disease genes can unravel disease relationships even when two diseases do not have seeds in common (see Figure S2B and C in Supplementary Methods).

Next, we ask whether network-based expansion of disease-associated gene information produces biologically meaningful associations between diseases. We use DisGeNET v2.0 (DGN2), an updated version of DGN1 containing text-mining and curated associations and focus on 234 diseases that are both represented in DGN1 and DGN2 and for which we can apply the expansion with GUILD (Fig. 1A, see Methods for details). We group the disease-associated genes of these 234 diseases into two sets: exploratory and validation sets, containing the genes in the GUILD expansion and the genes that are in DGN2 but not in DGN1, respectively (Fig. 1B). In the validation set, the disease-associated genes from DGN1 are removed to avoid circularity. Then, we define the “common gene” measure (CG) quantifying the significance of the overlap between two data sets (assessed by one-sided Fisher’s exact test, see Fig. 1C and methods). CG measure ensures that the DDAs identified by genetic overlap in network-based expansion (exploratory set) are also supported by new evidences in the updated version of DisGeNET (validation set).

The study of the shared genes between two diseases has been used to explain the association between diseases in previous studies^6, 15^. Nevertheless, the genetic overlap provides only a partial picture of comorbid diseases mechanisms. Thus, in addition to the CG measure relying on common genes between two diseases in the two data sets, we design two functional measures based on the overlap of the biological pathways between the diseases: i) a functional measure of common genes (FCG); and ii) a functional measure of common functions (FCF). FCG extends the CG by calculating the significance of the overlap between the exploratory and validation data sets in terms of biological functions performed by the genes shared by two diseases (Fig. 1D). FCF, on the other hand, first checks the enrichment of biological functions carried out by the disease-associated genes and then quantifies the significance of the overlap between the exploratory and validation data sets in terms of the biological functions shared by two diseases (Fig. 1E). These measures aim to characterize the associations between diseases not only by the genes involved in each of them but also by the biological processes in which these genes are involved, giving a more holistic view of the potential DDAs. For both functional measures, we define the functional terms using Gene Ontology (GO) biological processes (named FCG-GObp and FCF-GObp measures), GO molecular functions (named FCG-GOmf and FCF-GOmf measures) and Reactome pathways (named FCG-RP and FCF-RP measures).

**Figure 2:**
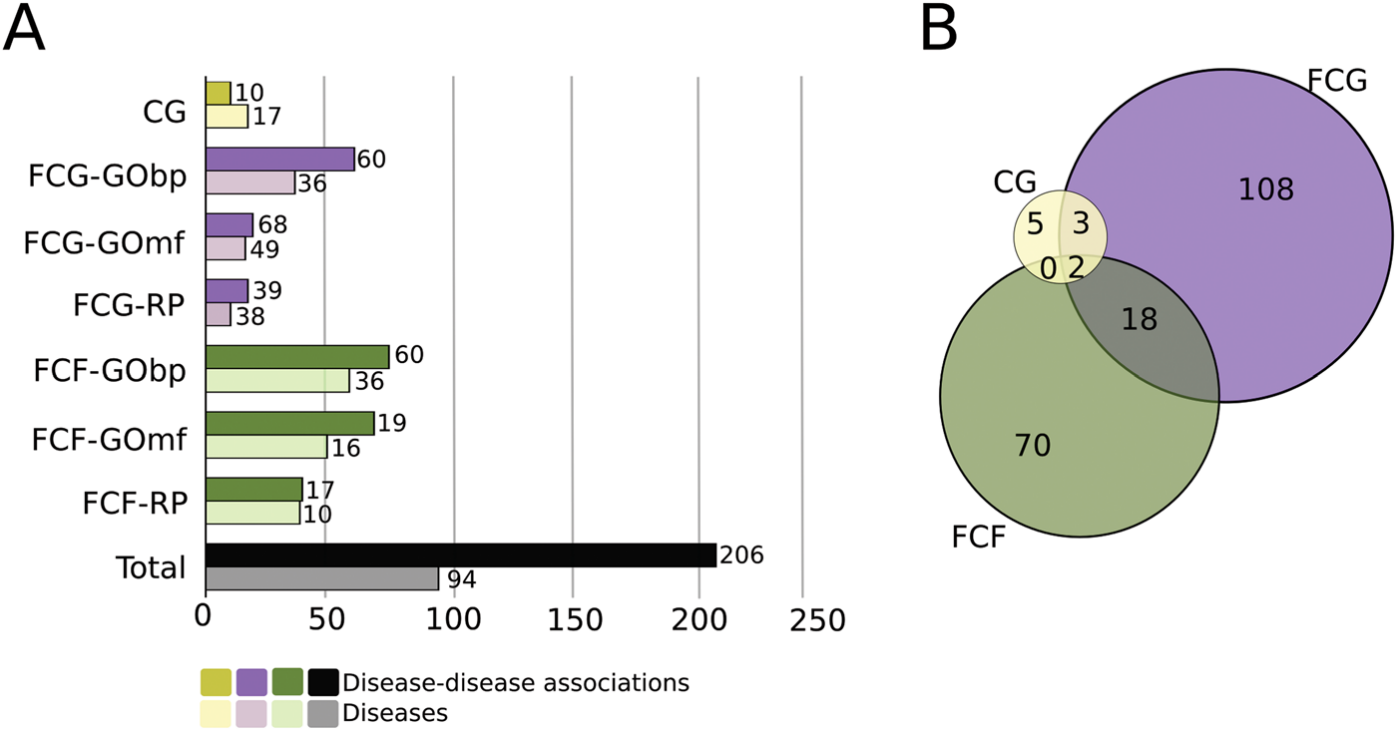
Number of disease-disease associations according to the strict criterion. (**A**) Light bars represent the number of diseases considered in each measure. Dark bars represent the number of significantly associated disease pairs identified according to each measure (CG in yellow colour, FCG in purple and FCF in green) using the strict multiple hypothesis testing criterion. The number of disease pairs linked by at least one of the measures are given in black, showing the total number of associations obtained. (**B**) Venn diagram of the sets of associations found by the CG, FCG and FCG measures and their intersections.

**Figure 3:**
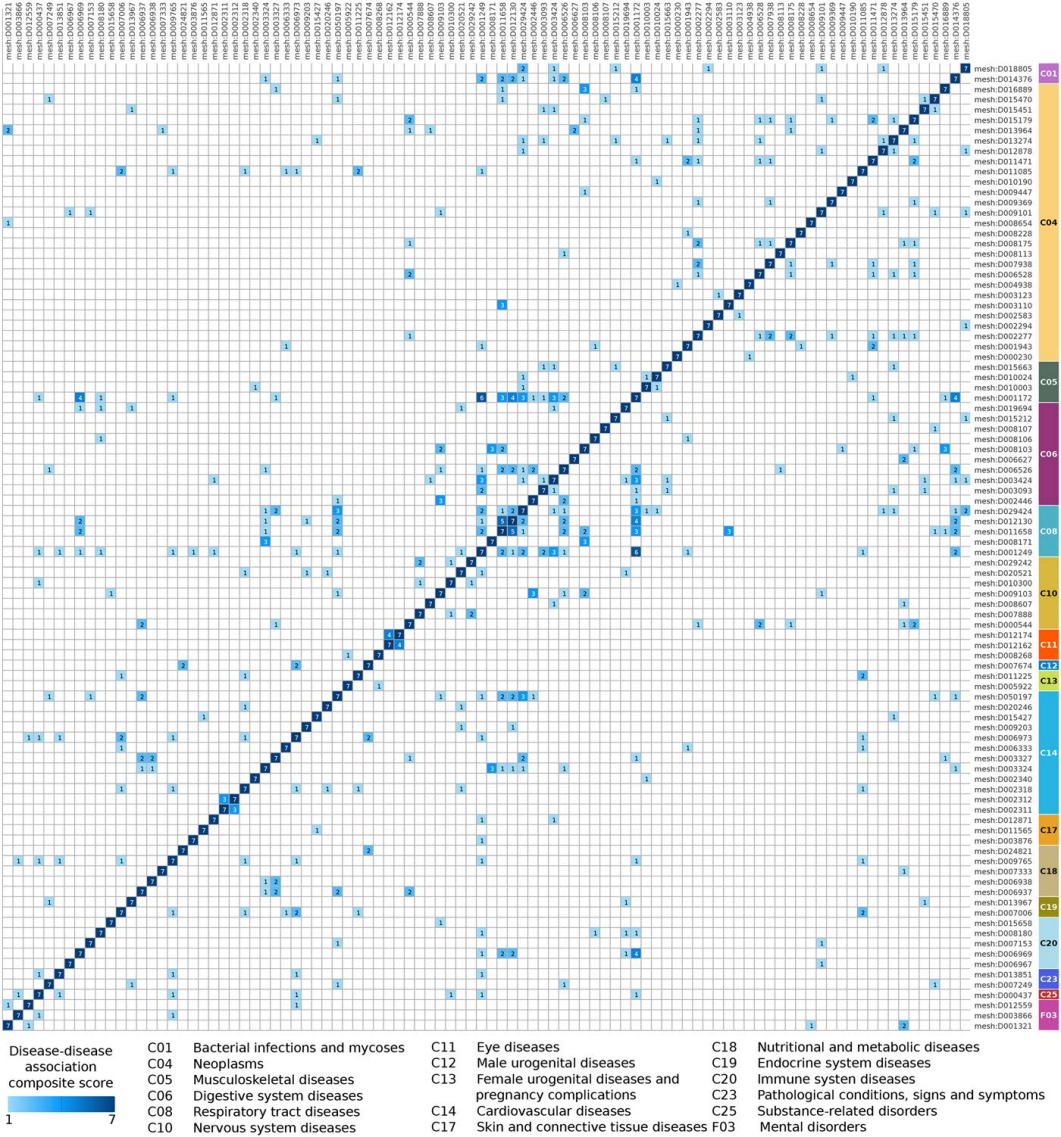
Heatmap of the composite score of disease-disease associations according to the strict criterion. Heatmap of the disease relationships showing the disease-disease association composite score, from 1 (pale blue) to 7 (dark blue) (see Methods for details on the score). Diseases are shown with their MeSH disease IDs (see the corresponding disease names in Table S1) and were ordered according their diseases class. Right bar of the heatmap shows the disease class where each disease belongs to.

Next, we seek to characterize the DDAs between the 234 diseases using the seven measures defined above, i.e., CG, FCG-GObp, FCG-GOmf, FCG-RP, FCF-GObp, FCF-GOmf, FCF-RP. We identify 206 DDAs involving 94 diseases, in agreement with a strict criterion of multiple testing correction (see Methods and Fig. 2) and 2,688 DDAs involving 158 diseases, considering a relaxed multiple testing criterion (Figure S3). Strict criterion, which minimizes the inclusion of false positives, has been used for the investigation of the Disease Network and the case studies, introduced in following sections. As each pair of diseases can be identified by any of the seven measures above, we define a composite score as the number of significant measures supporting each DDA. Figure 3 shows the composite scores of all DDAs found in accordance with the strict criterion ranging from 1 to 7. The list of all DDAs is provided in Table S2 and a summary of the significant DDAs using both criteria can be found in Table S3.

It is noteworthy that the functional measures reveal novel disease-disease links, complementing the genetic overlap based approach. Indeed, 196 significant relationships between 71 diseases are found using the strict criterion by means of FCG and FCF measures but not CG, spanning 95% of all identified significant DDAs.

### Comparison to previous works (state-of-the-art)

**Figure 4:**
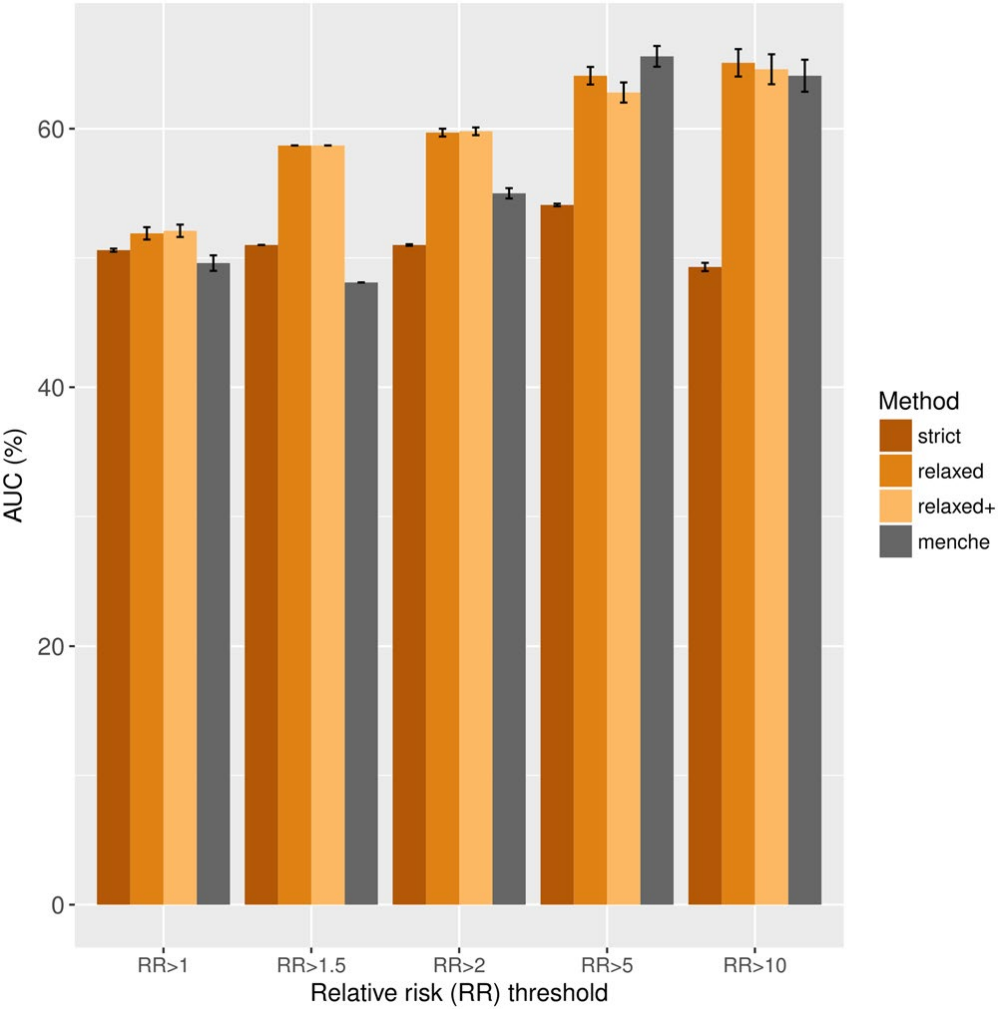
Prediction accuracy of composite score and network-based separation approach in characterizing disease comorbidities. The bars show the area under ROC curve (AUC) using the disease-disease associations reported previously by Hidalgo *et al*.^25^, at varying relative risk (RR) thresholds as the gold standard. For each RR threshold, we calculate the true positive rate and false positive rate over various prediction score cutoffs to generate a ROC curve and find the area under ROC curve. We randomly sample among the unknown (negative) associations to balance the number of positive associations with the number of negative associations and repeat the procedure 100 times to have robust estimates for mean AUC. The bars represent mean AUC and the error bar correspond to the standard error over 100 runs. The measures are the composite score calculated using genetic and functional overlap based on strict and relaxed criteria, their combination (relaxed+) or the interactome based separation measure introduced recently^15^.

To systematically compare our approach to previous works we turn to the data set by Hidalgo and colleagues, containing information on how often two diseases appear together in health insurance claims^25^. Although, this data set is not manually curated, one can compute the relative risk (RR) based on the co-occurrence frequencies of a given disease pair and use it as an indicator of disease comorbidity. Accordingly, we compare the prediction accuracy of the composite score calculated using genetic and functional overlap to the interactome based separation measure introduced recently^15^ using the disease-disease associations identified in health insurance claims at a range of RR values. For each RR threshold, we calculate the true positive rate and false positive rate over various prediction score cut-offs (composite score or separation measure) to generate a ROC curve and calculate the area under ROC curve (AUC, Fig. 4). Interestingly, the AUC increases as disease pairs with low RR values are filtered from the gold standard, an observation we attribute to the skewness of RR toward higher values in the original data set (Figure S4A). Furthermore, we observe that when the disease-disease associations with a RR higher than 2 are considered, the composite score outperforms the separation measure. We also find that integrating functional information of the disease-associated genes (FCG and FCF measures) improves the prediction accuracy compared to using common genes alone (Figure S4A).

In addition to the large-scale comparison using clinical data, we perform an exhaustive search on the relevant literature describing disease comorbidities. The proposed functional measures (FCG-GObp, FCG-GOmf, FCF-GObp, FCF-GOmf and FCF-RP) reveal connections between seemingly unrelated diseases that belong to different MeSH classes^26^, yet still share common functional processes. For example, multiple sclerosis and rheumatoid arthritis are significantly linked at the functional level (corrected p-values 2.8 × 10^−4^ and 2.5 × 10^−7^ using FCG-GObp and FCF-GObp measures in the relaxed criterion, respectively) through GO terms related to inflammation (i.e. GO:0019221, GO:0050852 and GO:0002606). These two diseases have been recently associated using the network-based separation^15^ and had already been highlighted by Hidalgo *et al*.^25^, in their comorbidity analysis on MedPAR records–clinical records of patients admitted in Medicare system. Similarly, the association between asthma and celiac disease identified in both studies^15, 25^, are also identified using FCG-GObp, FCF-GObp and FCF-RP measures (corrected p-values 1.7 × 10^−4^, 3.6 × 10^−3^ and 5.1 × 10^−4^, respectively).

Moreover, we compare our work to that of Menche’s^15^ (Table S4). Using the 71 diseases common in both studies, we find a considerable overlap between our significant (corrected p-value < 0.05) DDAs and DDAs in Menche’s work: 36 diseases with 55 DDAs and 67 diseases with 582 DDAs, according strict and relaxed criteria, respectively. From the mapped DDAs, most of them have a Sab close to 0 or negative (separation Sab is the criterion in Menche’s study to establish disease-disease links). Precisely, from the previously mentioned DDAs, 15 out of 55 and 96 out of 582 have a Sab < 0 (see Sab distribution in Figure S4B). Of the 15 DDAs found through the strict criterion and with a Sab < 0, only two have genetic support in our study (i.e. significant using the CG measure) and the rest are associated through functional measures (either FCF or FCG).

### Clusters in the disease network highlight known comorbidity links

**Figure 5:**
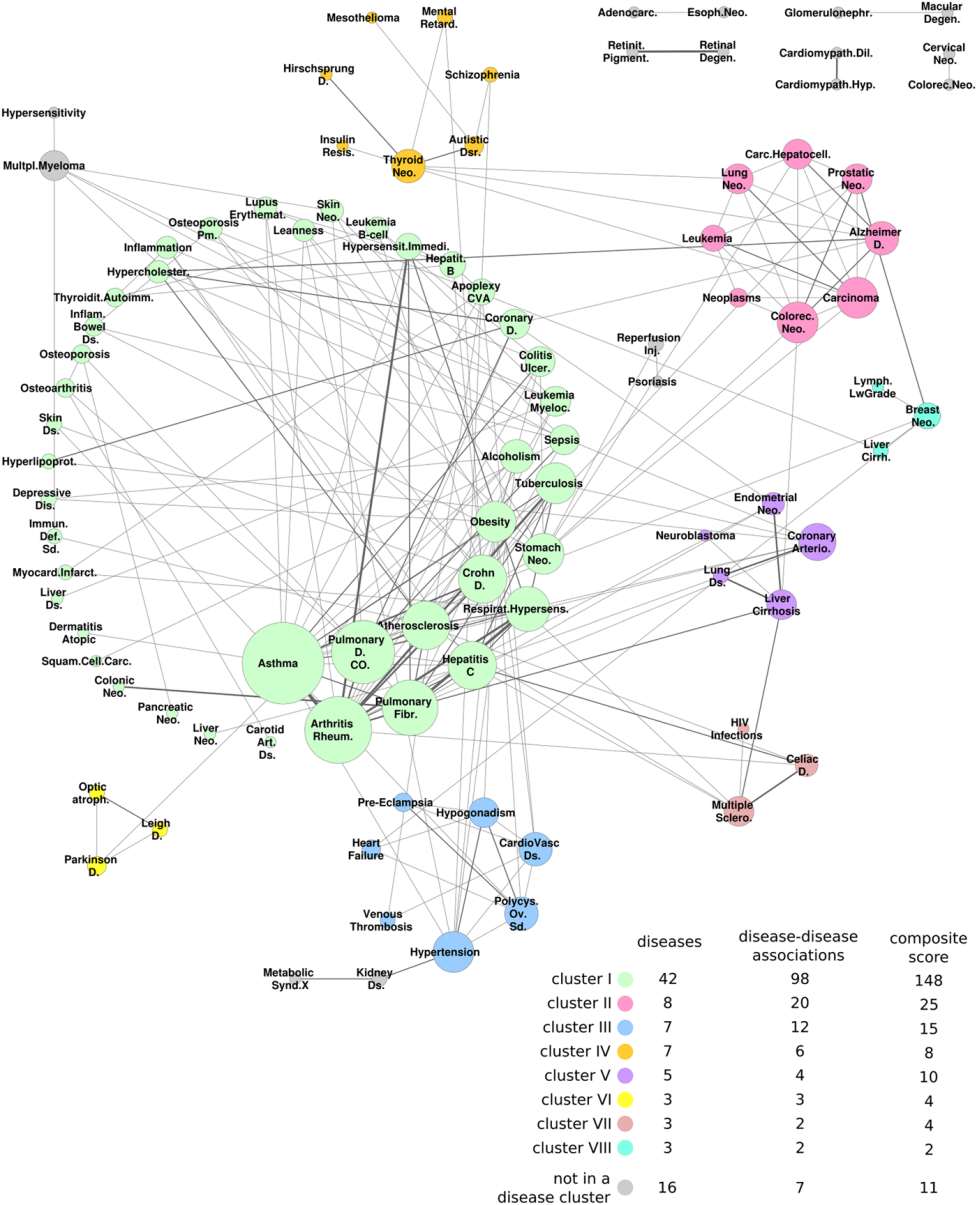
Disease-disease association (DDA) network. Disease network formed by 94 diseases (nodes) and 206 DDAs (edges) clustered using the MCL algorithm. Edge width is proportional to the composite score. Node size is proportional to its degree. Colour labels of clusters are shown at the bottom, along with the total number of diseases, the number of DDAs in the cluster and the sum of the composite score within the cluster.

To gain a better understanding of the interplay between diseases and their relationships, we assemble a weighted disease network (DN) connecting 94 diseases with the 206 identified links using the strict criterion (Fig. 5). In the DN, nodes represent the diseases and edges are weighted by the composite score, that is, the number of measures that identified significant links (i.e. CG, FCG-GObp, FCG-GOmf, FCG-RP, FCF-GObp, FCF-GOmf and FCF-RP, see Tables S3 and S4).

The disease with the highest degree (connectivity) in the DN is asthma (k_asthma_ = 20), followed by rheumatoid arthritis and chronic obstructive pulmonary disease with k_RA_ = 16 and k_COPD_ = 15, respectively. Note that rheumatoid arthritis is well-known in the clinic for having several comorbid conditions^27^, thus, it is not surprising that it is among the most connected diseases in the DN. We observe that the connections between asthma, rheumatoid arthritis, chronic obstructive pulmonary disease and respiratory hypersensitivity tend to be supported by multiple measures, hinting toward the common inflammatory processes underlying them.

Clustering the DN using MCL^28^, yields a total of 8 clusters, each with more than three diseases, and 7 non-clustered pairs (Fig. 5). In agreement with previous observations^25^, most of the clusters are represented by a homogeneous pathophysiology (i.e. cluster III is mostly formed by cardiovascular diseases). Moreover, seemingly heterogeneous clusters (i.e. cluster IV including neoplasms with mental disorders and cluster II including neoplasms and Alzheimer diseases) are supported by the literature^29–32^.

Cluster I includes diseases with the highest degree in the DN and those with highest composite score. Although it involves diseases in different MeSH disease classes, most diseases in this cluster have a prominent inflammatory component. The remaining clusters are much smaller in size, each consisting of 3 to 8 diseases. Among these, the largest clusters are cluster II, enriched in neoplasms and including Alzheimer disease; cluster III, enriched in cardiovascular diseases and cluster IV, enriched in neoplams and mental disorders.

**Figure 6:**
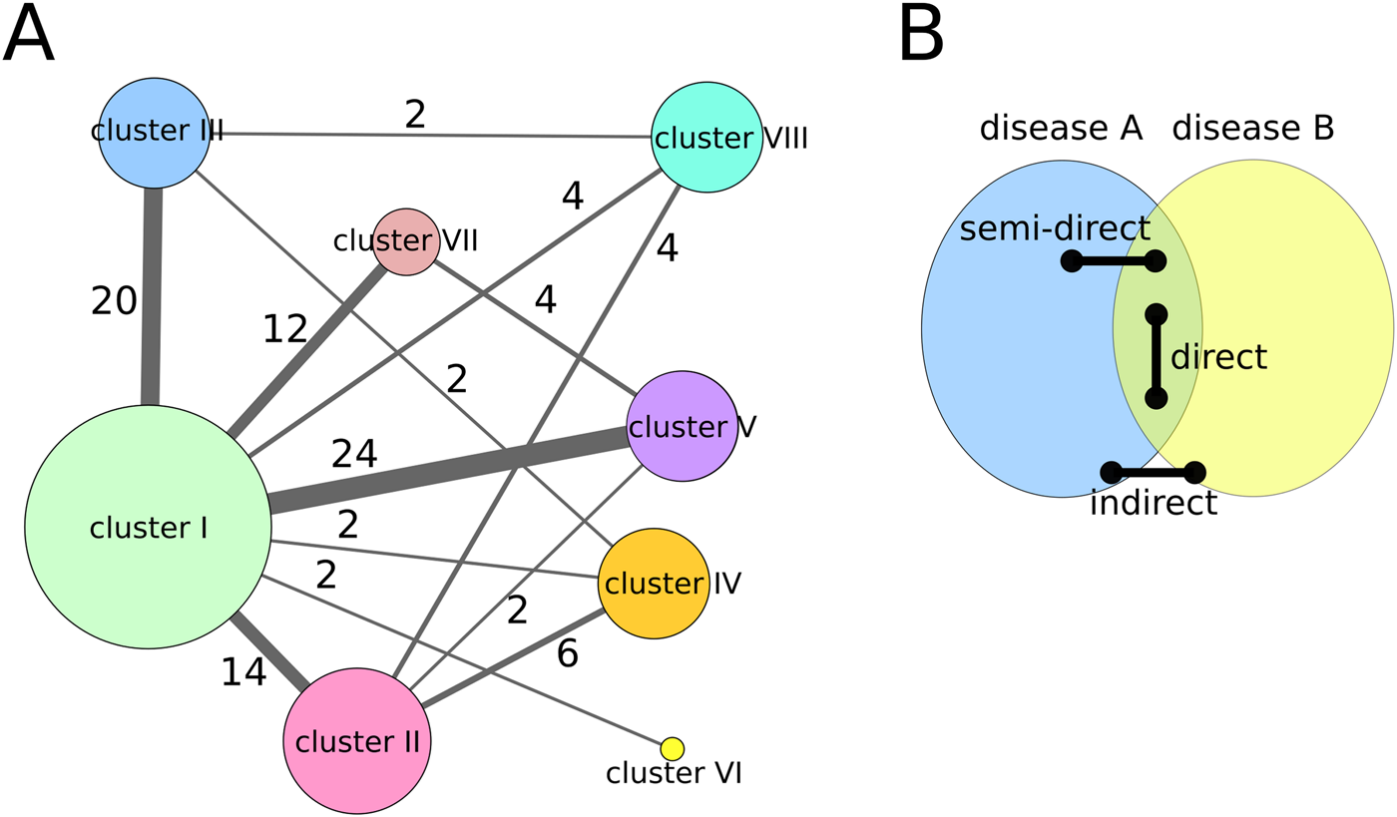
Clusters in the disease network. (**A**) Disease network represented by clusters of diseases. A node corresponds to a cluster of diseases and its size is proportional to its degree. Edges represent inter-cluster relationships and are weighed as the sum of the composite scores over inter-cluster DDAs. Numbers on top of the edges show the number of DDAs connecting the clusters. (**B**) Schematic representation of categorical links between diseases A and B: direct, semi-direct and indirect.

Coronary atherosclerosis (in cluster V) and hypertension (in cluster III) present the highest number of inter-cluster links mainly with cluster I, 7 and 6 links respectively (see Fig. 5 and Fig. 6A). In fact, the strong association between cluster I and V is mainly because of coronary atherosclerosis DDAs (Fig. 6A) whereas the association between cluster I and III is mainly due to hypertensions’ DDAs.

### Protein-protein interactions mediating potential comorbid relationships between diseases

Having established i) the pair-wise relationships between diseases by means of common genes and shared functions and ii) the highly connected modules of diseases, we turn to investigate the molecular basis entailing these links. To this end, we first consider the interactions between proteins that are involved in identified disease pairs. For any given pair of diseases, three different categories of links are defined based on the interactions of proteins (i.e. gene products) associated with each disease (Fig. 6B): (i) *direct*, when the two proteins of the interaction are associated with both diseases; (ii) *semi-direct*, when one of the proteins is associated with both diseases and the other is associated with only one disease; and (iii) *indirect*, when the interacting proteins are solely related to one or the other disease, i.e. none of the genes that produce them are common of both diseases.

Semi-direct and indirect links are of interest because they can potentially explain associations between diseases as the result of mutations at protein interaction interfaces. A mutation at the interface may disrupt the interaction between key proteins, or produce an edgetic perturbation^21^, which in turn would explain why perturbations on a single protein can lead to different phenotypes^22^. In total, about 39% of PPIs are indirect, 44% are semi-direct, and 17% direct (see Table S5). Therefore, although most predicted associations between diseases are derived from proteins associated and shared with both diseases, more than one third of them are purely caused by “edge” (indirect) contributions.

To further understand the mechanistic and structural details of these disease relationships, point mutations from different sources are compiled and mapped onto PPIs whose interface has been resolved at an atomic level or it can be inferred (see Materials and Methods). Details on disease-disease associations with mutations affecting the interfaces of the PPIs that link both diseases, for which at least one of the mutations is directly associated with a disease, are provided in Table S5B and S5C.

In Figure S5, we show the example with available structural information of a protein complex that associates two diseases. This protein complex is formed by the interaction between the transforming growth factor ß receptor type-2 (*TGFBR2*, accession number P37173) and the transforming growth factor ß-3 (*TGFB3*, accession number P10600). The genes that produce *TGFBR2* and *TGFB3* are associated with diseases in cluster I. *TGFBR2* is also associated with diseases in cluster II. This molecular interaction yields semi-direct types of links between colonic/pancreatic neoplasms and *i*) skin neoplasms, *ii*) osteoporosis and *iii*) pulmonary fibrosis. *TGFBR2* (TGF-beta type I serine/threonine kinase receptor) is part of the *TGF-β* signaling pathway, one of the most commonly altered pathways in cancer^33^. It binds isoforms of TGF-β (TGFB3 among them), which are tumour suppressors in the healthy intestinal epithelium, inhibiting cell proliferation and promoting apoptosis^34^. A mutation affecting the association between *TGFBR2* and *TGFB3* would potentially affect the transduction of *TGFB3* signal to the cytoplasm, affecting the expression of cell-cycle checkpoint genes. Furthermore, both proteins are associated with multiple types of neoplasms, while *TGFB3* has been associated with lung neoplasms, liver cirrhosis, osteoporosis and pulmonary fibrosis^35, 36^.

We observe that *TGFBR2* and *TGFB3* interact through PFAM domains PF08917 (ectodomain of the transforming growth factor ß receptor type-2) and PF00019 (transforming growth factor ß-like domain), respectively. The three-dimensional structure of this interaction is known^37^ and deposited in the PDB^38^ (PDB code 2PJY). The structural analysis reveals that the substitution I50V in *TGFBR2* (i.e. the residue I73V in the UniProt sequence of P37173) affects the interface between both proteins. The analysis of the effect of the mutation on the interaction with ROSETTA^39^ shows a decrease of binding energy in the mutant form I50V of *TGFBR2*, with ΔΔG≈ + 1 kcal/mol (see Table 1), indicating a potential loss of the interaction. Likewise, PCRPi-Webserver^40^ assigns the highest probability among all interface residues of *TGFBR2* to I50, as a critical (i.e. hot spot) residue in the interaction. Furthermore, this mutation has been associated with colorectal cancer^41^, thus further supporting the significant association we identify between colorectal and lung cancer through functional measures (Table S3). The combined functional and molecular level evidence suggests I50V mutation on *TGFBR2* as a potential biomarker for lung cancer.

**Table 1:** Energy analysis of interface mutations in TGFBR2-TGFB3 and BAX-BID complexes.

Protein interaction	SNP	ΔG_wildtype_	ΔG_SNP_	ΔΔG	Hot-spot probability
TGFBR2-TGFB3	TGFBR2 I50V	−10.03	−7.34	2.69	0.9947
BAX-BID	BAX R65L	−16.75	−13.29	3.46	N/A*
BAX-BID	BAX G108V	−16.75	138.88	155.63	0.9999
BAX-BID	BID R84W	−16.75	−14.11	2.64	0.0526
BAX-BID	BID G94E	−16.75	36.02	52.77	0.9999
BAX-BID	BID L105P	−16.75	6.09	22.84	0.9864

The first and second columns indicate the complex and the SNP affecting the interface. The next two columns show the ΔG calculated with Rosetta of the wild-type complex (ΔG_wild-type_) and the SNP mutant form (ΔG_SNP_). The third column shows the (ΔΔG = ΔG_wild-type_ − ΔG_SNP_) –energies are represented in Rosetta units, which are approximately the same as kcal/mol. The fourth column show the probability calculated by PCRPi-Webserver. *Residue not considered by PCRPi-Webserver as part of the interface.

### Alzheimer’s disease and cancer comorbidity: from genetics to structural characterization

Alzheimer’s disease comorbidity with cancer has recently raised the interest of the scientific community. Nonetheless, the mechanism underlying the link between these two diseases is still not well understood. While some early studies argue that this is a case of direct comorbidity^42, 43^, more recent evidence suggests that it would be an example of inverse comorbidity^44, 45^. Inverse comorbidity is a lower-than-expected probability of developing a disease in individuals who have been diagnosed of a previous condition^32^. In this case, patients with Alzheimer’s disease have a decreased probability of developing cancer and *vice versa*.

On the basis of these epidemiological studies, the current evidence suggests that the inverse comorbidity stems from the pathogenesis of neurodegeneration, involving the following players: *PIN1* (altered or under-expressed in Alzheimer’s disease and over-expressed in cancer); *TP53* (promoting cell cycle arrest in Alzheimer’s disease and preventing from it in cancer, due to its loss or mutation), γ-secretase inhibition and trade-off effects of *APOE*; and impairment of the proteasome (with a dysfunction in Alzheimer’s disease which increases the deposit of Lewy bodies and an up-regulation in cancer)^44, 45^. Yet, the functional evidence of the molecular basis underlying the inverse comorbidity has been reported recently in the studies of Sorrentino *et al*.^46^ and Ibáñez *et al*.^31^, attributing mitochondrial apoptosis deregulation by *PIN1*.

In our DN, we observe significant associations between Alzheimer’s disease and five different types of neoplasms (hepatocellular carcinoma, the generic term carcinoma, lung-, colorectal- and thyroid- neoplasms), giving rise to a disease cluster containing Alzheimer disease and 7 neoplasms (cluster II in Fig. 5). Moreover, if the relaxed criterion is used, we identify 19 additional neoplasm types associated with Alzheimer (including non-solid tumors, e.g. leukemia). Interestingly, the associations between Alzheimer disease and neoplasms are found by means of commonly enriched GO terms -FCF-GObp and FCG-GObp, see (Table S3A).

The GO terms enriched both in Alzheimer disease and neoplasms point to induction of apoptosis through caspase activation by the mitochondrial cytochrome C, implicating programmed cell death as the underlying common mechanism behind the comorbidity of these diseases (see Table S6A and B). These findings suggest that apoptosis triggered by neurodegeneration in Alzheimer’s disease may play a protective role in various cancer types by promoting programmed death of cancer cells and *vice versa*.

An analysis of the semi-direct and direct PPIs between Alzheimer’s disease and neoplasms identifies the interaction between the apoptosis regulator *BAX*, from BCL2 apoptosis regulators family (PF00452) and the BH3-interacting domain death agonist *BID* (PF06393) (PDB code 4BD2^47^), a pro-apoptotic regulator which promotes *BAX* oligomerization (see Reed^48^ and references therein). Both proteins are known to be associated with neoplastic processes. *BAX* has also been associated with Alzheimer’s disease^49, 50^. Based on our findings, a perturbation of this interaction may lead to an association between Alzheimer’s disease and some neoplasms. This interaction is predicted as indirect links between hepatocellular carcinoma and Alzheimer’s disease and between stomach neoplasm and Alzheimer’s disease. The mutation G108V of *BAX*, in the interface of the interaction with *BID*, has been associated with Burkitt Lymphoma^51, 52^. We find that the predicted impact of the mutation in the interaction can be important (Table 1). Moreover, the mutation R65L, also near the interface, has been associated with lymphoblastic leukemia^51, 52^ and it can play a role in the interaction. We also identify three mutations of *BID* in the interface (L105P, R84W and G94E). Although they are not currently associated with any disease, the energy analysis and the predictions of its relative importance in the interaction (i.e. hot spots) shows that these mutations can greatly impair the interaction between *BAX* and *BID* (Table 1).

**Figure 7:**
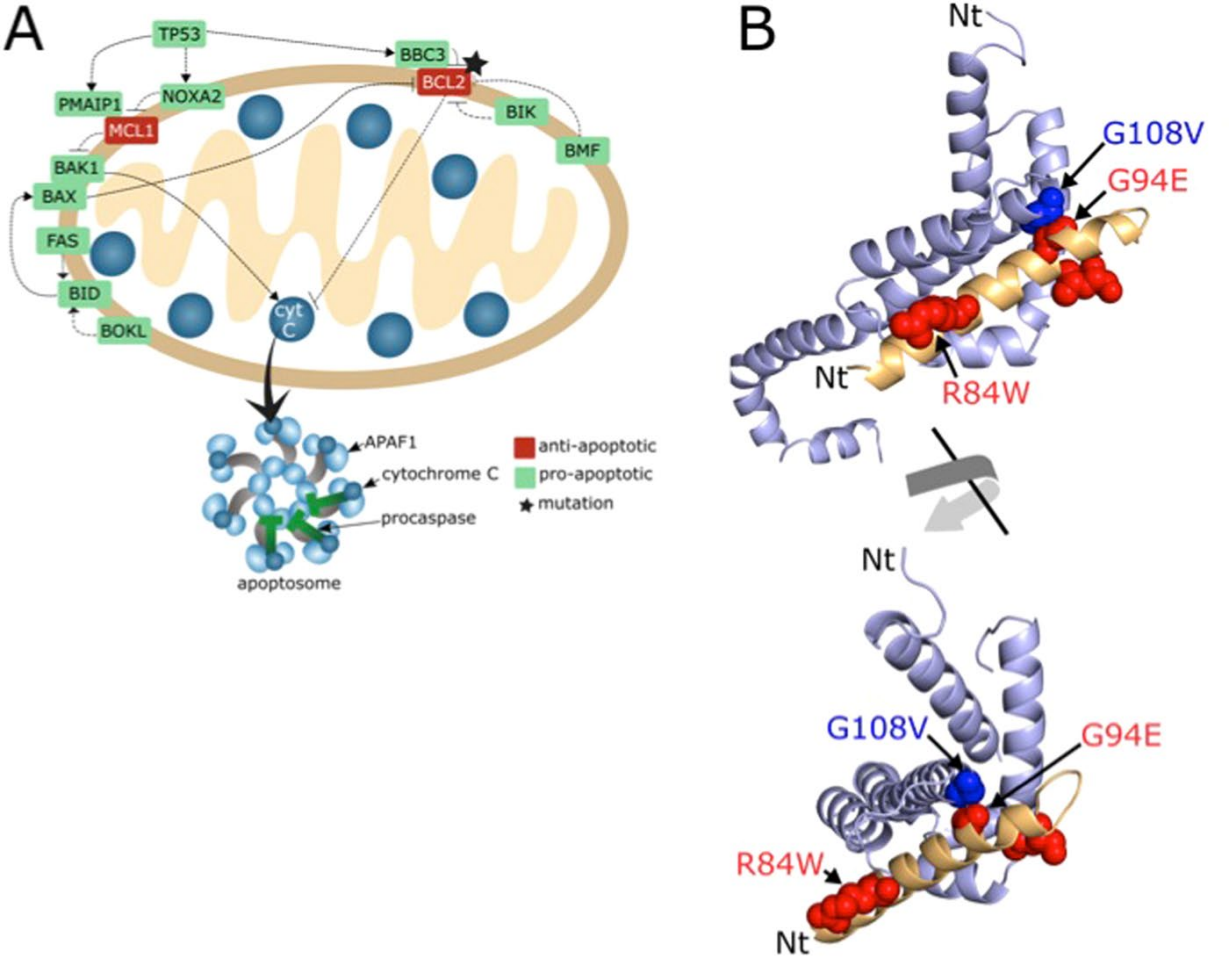
Proposed molecular mechanism underlying Alzheimer’s and neoplasms comorbidity. (**A**) Diagram of mitochondrial meditated apoptosis and TP53. Representation of the apoptosis pathway. Note that most genes cited in the text are pro-apoptotic molecules. (**B**) Interaction between the apoptosis regulator BAX and BH3-interacting domain death agonist BID. Cartoon representation of BAX (light blue) and BID (light orange) protein complex. Residues associated with mutations involved in neoplasm are shown in sphere representation in red for BID (L105P, R84W and G94E) and in blue for BAX (G108V). It is worth noting that G108 in BAX faces G94 in BID and that R65L mutation is not detected in the structure of the complex.

It has been shown that the elimination of *BAX* prevents almost half of apoptotic cell-deaths by chemotherapeutic agents, which otherwise induce apoptosis in embryonic fibroblast in a p53-dependent manner^53^. Also, *BID* is up-regulated by p53^54^ and mediates apoptosis by interacting with *BAX*, leading to its insertion in the mitochondrion outer membrane to open the mitochondrial voltage-dependent channel and forming an oligomeric pore complex known as Mitochondrion Apoptosis-induced channel (Fig. 7A). Therefore, the loss of BAX-BID interaction produces the wane of apoptosis and diminishes its p53-dependent induction. Hypothetically, this could produce predisposition for cancer and an inhibition of neurodegeneration, resulting in the inverse relationship between both diseases. Consequently, the functional analysis (i.e. based on GO enriched functions) and the molecular structural analysis (i.e. detailed by the BAX-BID interaction, Fig. 7B) may explain the inverse comorbidity between cancer and Alzheimer’s disease.

## Discussion

The relationship between diseases is often characterized by their common genes. Yet, despite the availability of GWAS and the “omics” revolution in the recent years, our understanding of the genetic basis of complex diseases and disease associations remains limited. Motivated by the observation that the interactome neighbourhood of the disease genes contain relevant information on the disease, we have used network-based disease-gene prioritization to extend our knowledge on disease-disease associations. We have proposed functional measures to define relationships between diseases and we have showed that expanding the disease-gene information could recover existing relationships between diseases. The expansion of associations based on the protein interaction network has helped us to find new potential disease-disease associations by filling the gaps in our current experimental and clinical knowledge. We have defined a composite score based on seven measures quantifying genetic and functional overlap between diseases and using this score, we have obtained 206 disease-disease associations. Indeed, we have found that many disease-disease associations were predicted by functional overlap and would be missed if only genetic overlap is considered.

We have contrasted our work with previous studies and we found that our results recapitulate known disease associations, obtained not only by network-based approaches, but also associations supported by epidemiological data. We have compared our predictions to those obtained by network-based separation^15^, finding support for 25% of the associations among diseases appeared in both studies. The associations in Menche’s work correspond to the closeness of disease related proteins in the network but also supports the functional overlap that we have identified. Taken together with other disease-disease associations, these results suggest that protein-protein interactions between genes associated with one or more diseases can be used to explain disease-disease relationships, extending our knowledge about the molecular underpinning of human diseases. Interestingly, the measures based on functional overlap can detect and confirm associations of diseases described in previous works, such as multiple sclerosis and rheumatoid arthritis or celiac disease. In addition to the network-based connectivity, some DDAs are supported by functional measures, such as asthma and respiratory hypersensitivity, which share several associated genes, or the connections between asthma, liver diseases, celiac disease and rheumatoid arthritis that were highlighted in previous works. Our functional overlap based approach also uncovers a substantial amount of disease relationships in which none of the genes are common to both diseases. Moreover, through a systematic comparison we verify that the predicted DDAs coincide with the epidemiological data and achieve favourable accuracy compared to the network-based separation measure. We also identify the modules of related diseases and investigate the implications of the protein-protein interactions in characterizing the links between diseases.

The clusters of similar diseases in the disease network show that many related diseases form modules. The largest interconnected cluster contains mostly diseases associated with the digestive system as well as rheumatoid arthritis, asthma and Chron’s disease. The remaining seven clusters are much smaller, containing DDAs that span various disease categories. Although many DDAs are within the cluster, some DDAs occur between pairs of clusters, such as coronary atherosclerosis in cluster V linked with diseases with an inflammatory component in cluster I.

We have also focused on the protein-protein interactions involved in DDAs. We have categorized DDAs according to the involvement of interacting proteins in one or both diseases. We have defined direct, semi-direct and indirect disease-disease associations and have used these definitions to analyse the disease network. The number of indirect and semi-direct links among different clusters (inter-cluster) is much larger than the number of indirect and semi-direct links within clusters (intra-cluster), while the number of direct intra-cluster links is about the same as the number of direct inter-cluster links. These results indicate that most links between diseases grouped in different clusters involve interactions of proteins whose genes are associated with one or other disease but not shared by both. Thus, the molecular analysis of protein-protein interfaces of interactions involved in disease-links becomes crucial to understand potential comorbidity relationships, capturing disease associations that would be missed by analysing only the genes in common.

In the analysis of mutations found in protein-protein interactions of genes associated with disease pairs, we find 15 and 96 similar DDAs, as per strict and relaxed criteria, respectively, that are also found in Menche’s study with Sab < 0. We have predicted the location of each mutation in the interface using the alignment of the protein sequence with the Hidden Markov profile and the structure of its known interactions in the 3DiD structural database^55^. Most DDAs found by our approach, with mutations in the interface of at least one of the proteins involved in interactions associated with both diseases (direct, semi-direct or indirectly associated), are found for diseases within the same cluster (i.e., intra-cluster diseases). Among them, we find some examples where the mutations were directly associated with at least one of the diseases. For example, the association between rheumatoid arthritis and liver cirrhosis is an indirect link between the metalloproteinase Inhibitor 2 (Uniprot accession P16035), associated with cirrhosis and the ubiquitinous collagenase type 2 (Uniprot accession P08253), associated with rheumatoid arthritis, with three mutations in the interface directly associated with rheumatoid arthritis. Another example is the semi-direct link through the Tumor Necrosis Factor (*TNF*) and its receptor in the superfamily 1B (*TNFR1B*), associated with rheumatoid arthritis and asthma, respectively. In this example, we highlight one mutation in the interface of the interaction directly associated with rheumatoid arthritis. It is worth noting that the link between asthma and rheumatoid arthritis was already described both in Hidalgo *et al*.^25^ and Menche *et al*.^15^. Moreover, in the past decade, several strategies have been proposed to target *TNF* receptor as a treatment for asthma (i.e. adalimumab, etanercep, infliximab)^56, 57^ and rheumatoid arthritis (i.e. in the DREAM Challenge 8.5^58^, in several cohorts with similar surveys^59–61^ and in ref. 62).

In this work, we have also provided a potential explanation for the inverse comorbidity observed between Alzheimer’s disease and cancer (such as leukemia, lung, colorectal, stomach and prostatic neoplasms and carcinoma) by means of the functional study and the enrichment of functions associated with apoptosis. The association between leukemia and Alzheimer’s disease was previously found by Hidalgo *et al*.^25^, but in Menche’s work this association was disregarded (Sab = 0.36), in which, among all cancer types, lung neoplasm had the smallest network-based separation with Alzheimer’s disease (Sab = 0.29). Furthermore, according to separation measure, Alzheimer’s disease is not associated with other diseases, the separation with arteriosclerosis being the smallest (Sab = 0.06). In agreement with this, by using our composite score we have linked Alzheimer’s disease with coronary heart disease and hypercholesterolemia. Interestingly, Alzheimer’s disease and myocardial infarction produces one of the few inter-cluster links with known mutations in the interface of the proteins involved in this association. However, our criteria on the genes/functions overlap significance and the network-based separation (with Sab > 0) are not sufficient to consider this link.

Finally, to demonstrate the molecular implications of the mutations in the interfaces of relevant interactions linking two diseases, we have characterized the interfaces of several examples for which the structure of the complex had been obtained by X-ray crystallography. We have characterised the protein interaction of *TGFBR2* associated with potential colorectal and lung cancer comorbidity, explaining how the mutation of Ile 73 to Val (residue 50 in the PDB structure) can cause the loss of the interaction and this, in turn, distorts the function. We have also analysed the interaction between BAX and BID, providing molecular evidence on the inverse comorbidity between cancer and Alzheimer’s diseases by means of changes in the pathways associated with apoptosis.

In conclusion, we have developed a new approach to uncover genetic and functional relationships between diseases that can be applied to investigate disease comorbidities. As a proof of principle, we have highlighted the interactions with known mutations associated with several disease pairs, corroborating the implication of mutations and their effect on the loss (or decrease of the strength) of relevant interactions that affect the network rewiring. Our results shed light on the common genetic and molecular mechanisms potentially giving rise to comorbidity between two diseases.

## Material and Methods

### Protein-Protein interaction network

The human interactome was derived using BIANA^12^ (see supplementary information), by integration of interactomic data from: HPRD^63^, DIP^64^, MIPS^65^, BioGRID^66^, BIND^67^, IntAct^68^ and MINT^69^ databases. The resulting protein interaction network (PIN) was formed by 11,123 proteins and 149,931 interactions.

### Genetic disease data

The collection of genes associated with diseases was obtained from DisGeNET database^70, 71^. DisGeNET contains gene-disease and variant-disease associations from different sources, including but not limited to the GWAS catalog^72^, Orphanet^73^, the Comparative Toxicogenomics Database^74^, UniProt^75^. Two different versions of DisGeNET were used in this study: DisGeNET v1.0^71^ (DGN1) and DisGeNET v2.0^70^ (DGN2). DGN1 contains 8,246 disease-gene associations from curated data (see Supplementary Methods for more information) and DGN2, which also includes disease-gene associations derived from text mining, has 57,912 disease-gene associations. In comparison to DGN1, DGN2, includes more data sources and integrates text-mining information, representing a more complete picture of the genetic underpinnings of human diseases. For our analysis, we focused on 234 diseases in DGN1 that had at least four genes in the PIN and that had additional disease-associated genes in DGN2 (Fig. 1A).

### Network-based prioritization of disease-associated genes

DGN1 genes were used as seeds, known set of disease-associated genes, for the network prioritization. We defined the genes in the GUILD expansion of DGN1 seeds as the exploratory set and the genes that were in DGN2 but not in DGN1 as the validation set. The exploratory set was obtained with the NetScore method in GUILD (see supplementary information). The genes annotated in DGN1 for each disease were initialized with a seed score of 1.0 and all the remaining proteins were assigned the score of 0.01. For each disease, GUILD calculates a disease-association score for all the genes by considering alternative shortest paths between seeds and all the other nodes in the network. GUILD scores for disease-associated genes were normalized to Z-scores (as described in ref. 20) and genes with a Z-score >= 2 were selected as part of the exploratory set of genes associated to the specific disease (see Fig. 1B).

### Quantifying disease-disease associations using genetic and functional measures

Uncovering links between diseases by expanding the number of genes can identify disease-disease associations (DDAs). To quantify the associations we defined a score, named *composite score*, which was formed by the implementation of seven different measures: G*enetic measure of common genes* (CG), based on the overlap of common genes between diseases; *Functional measure of common genes* (FCG)–for Gene Ontology (GO) biological processes (FCG-GObp), GO molecular functions (FCG-GOmf) and Reactome pathways (FCG-RP) - based on the functions enriched in the set of common genes between two diseases; and *Functional measure of common functions* (FCF)–FCF-GObp, FCF-GOmf and FCF-RP–based on the independent enrichment of functions by each disease and the overlap of them (Fig. 1C to E shows a schematic representation of the different measures and more details are provided in Supplementary Methods).

These seven measures were then used to assess the statistical significance of a link between two diseases using the exploratory and validation sets. For each pair of diseases, we compared the overlap of: genes (CG), gene-related functions (FCG-RP, FCG-GObp, FCG-GOmf) and functions (FCF-RP, FCF-GObp, FCF-GOmf) in the exploratory set and in the validation set. Then, we used the hypergeometric distribution to calculate a p-value, as in Eq. 1, for each measure.

1 $$p\mbox{--}value=\sum _{i=k}^{n}\frac{(\begin{array}{c}n\\ i\end{array})(\begin{array}{c}N-n\\ K-i\end{array})}{(\begin{array}{c}N\\ K\end{array})}$$

For instance, to calculate the CG measure for a DDA: *N* is the sample size (number of genes in the PIN), *K* is the number of common genes associated to the diseases in the *exploratory set*, n is the number of common genes associated to the diseases in the *validation set*, *k* is the number of correct predictions (i.e. number of genes of K set within n) (see Supplementary Methods the details for the other measures).

We defined two different criteria for multiple testing p-value correction, strict and relaxed, applying Bonferroni or Benjamini Hochberg FDR correction, respectively. P-values were corrected at the level of functional enrichment and the final p-value obtained for each pair of diseases. After correcting all p-values, we computed the *composite* score (from 1 to 7) for each DDA, corresponding to the number of significant measures with corrected p-value smaller than 0.05 (a 0 value on composite score implies no DDA).

The full implementation of the seven measures is available at: https://bitbucket.org/carlotarp1/disease_disease_associations.

### Benchmarking identified DDAs using relative risk

To compare the results obtained by our approach with those of the work by Menche *et al*. and evaluate the performance of the method, we used a data set containing data from hospital claims for over 30,000,000 of patients^25^. We downloaded the file corresponding to the ICD9 codes at the 5 digit level from Hudine data resource^25^ (http://barabasilab.neu.edu/projects/hudine/resource/data/data.html). We performed the mappings of the ICD9 codes to the disease identifiers used in DisGeNET using the Unified Medical Language System (UMLS) Metathesaurus (version 2016AA). We obtained the Relative Risk (RR) as computed by Hidalgo *et al*.^25^. The RR is the ratio of the co-occurrence of a given disease pair and the random expectation based on each disease prevalence in the population under study. RR is sensitive to diseases with low prevalence, that is, diseases seen in a small number of patients and the RR values for such diseases is exceptionally high (≫10). Accordingly, we filtered the disease pairs with less than 100 patients in common, focusing on the disease pairs with strong underlying evidence for potential comorbidity. We retrieved the network-based separation values from the supplementary material of Menche *et al*.^15^. We mapped the diseases in this file to the vocabularies used in DisGeNET by strict matching of the disease names to the diseases in the Unified Medical Language System (UMLS) Metathesaurus (version 2016AA), using only MeSH as source vocabulary. Next, to compare the prediction accuracy of the composite score calculated using genetic and functional overlap to the interactome based separation measure we calculated the true positive rate and false positive rate, where the disease-disease associations larger than a certain RR value were used as positive associations and negative associations are randomly sampled from the remaining associations to match the size of the positive associations. We have used 1, 1.5, 2, 5, 10 RR thresholds to call positive associations and reported the mean AUC over 100 different samplings of negative associations.

### Deriving a disease network and clustering diseases

A disease network (DN) was derived as explained in the text: diseases (nodes) were connected (edges) based on the number of significant measures that yields the composite-score. Diseases on the DN are have been clustered using the network-based Markov Cluster Algorithm (MCL)^28^ with default parameters.

### Mapping mutations to protein interfaces

To further understand the structural basis between two linked diseases, we have selected the interactions between pairs of proteins produced by the genes associated with each disease and mapped in the proteins the missense mutations extracted from: SNPdb^76^, the Human Polymorphisms and Disease Mutations database at UniProt^75^, the ClinVar database^77^, the Genetic Association Database^78^ and the NHGRI GWAS Catalog^72^. Overall, 5,241 proteins have mutational information of which 2,472 are associated to at least one disease. Of those, 108 complexes, i.e. pairs of proteins, have structural information and mutations associated to them can be analysed in their structural context.

Whenever possible, we have characterized the mutations located in protein-protein interfaces by using the structure of protein complexes from 3DiD^55^. In the case of protein complexes with known structure, the interface is defined as the set of residues of each protein within less than 12 Angstroms Cβ-Cβ distance, as defined in 3DiD. For complexes of proteins highly similar to the known interactions (usually referred as interologs), we inferred the residues in the interface from the alignment with the corresponding template structure using Align^79^.

### Calculation of ΔG of a protein-protein interaction and hot spot predictions

We have used the program InterfaceAnalyzer of ROSETTA package to calculate the ΔG^39^. A single mutation in the interface is modelled and optimized with MODELLER^80^ and the energy of the interaction is calculated using ROSETTA. The difference between ΔG of the mutant and the wild-type form is the ΔΔG. Hot spots on interfaces are predicted using PCRPi-Webserver^81^ employing a naïve Bayesian Network trained in a dataset that does not include Antigen-Antibody complexes (as described in the original method^40^).

## Electronic supplementary material

Supplementary Information

Table S1

Table S2

Table S3

Table S4

Table S5

Table S6

Dataset_S5
