# Supplementary material for: Genetic and functional characterization of disease associations explains comorbidity
Source: Sci Rep. 2017 Jul 24;7:6207. doi: 10.1038/s41598-017-04939-4 (PMC5524755; doi:10.1038/s41598-017-04939-4)
Supplement: Supplementary file 1 — Supplementary Information [file 41598_2017_4939_MOESM1_ESM.pdf]

Supplementary Material for:
Genetic and functional characterization of disease associations explains comorbidity

Carlota Rubio-Perez, Emre Guney, Daniel Aguilar, Janet Piñero, Javier Garcia-Garcia, Barbara Iadarola, Ferran Sanz, Narcís Fernandez-Fuentes, Laura I. Furlong and Baldo Oliva

SUPPLEMENTARY METHODS 2

SUPPLEMENTARY FIGURES 9

- Figure S1 9
- Figure S2 10
- Figure S3 11
- Figure S4 13
- Figure S5 14

SUPPLEMENTARY TABLES 15

- Table S1 15
- Table S2 15
- Table S3 15
- Table S4 15
- Table S5 16
- Table S6 16

SUPPLEMENTARY METHODS

Protein-Protein interaction network

The human protein interaction network used in this study, hereinafter referred to as PIN, was derived using BIANA[1], by integration of interactomic data from: HPRD [2], DIP[3], MIPS[4], BioGRID[5], BIND[6], IntAct[7] and MINT[8] databases using the protein sequence, UniProt Accession number [9] and NCBI Entrez gene identifiers [10] as unifying criteria. The resulting PIN was composed of 11,123 nodes (i.e. proteins) and 149,931 edges (i.e. interactions.) The mapping of proteins in the PIN and its corresponding disease-associated genes was done using using the NCBI Entrez gene identifiers (see next section).

Genetic disease data

The collection of genes associated with diseases was obtained from DisGeNET database [11], Two different snapshot versions of DisGeNET were used in this study: DisGeNET v.1.0[12], referred as DGN1 and DisGeNET v2.0[11], referred as DGN2 throughout the text. The mapping between genes in DGN1 and DGN2 was done using the Unified Medical Language System (UMLS) [13] disease identifiers in the Medical Subject Headings (MeSH) [14]. For this mapping, whenever possible, the Online Mendelian Inheritance in Man (OMIM) identifiers [15] were matched to corresponding MeSH terms using UMLS.

BIANA approach to integrate several resources of protein-protein interaction data

BIANA (Biologic Interactions and Network Analysis) is a platform designed to compile and integrate interactomic data from multiple sources in a comprehensive and traceable manner [1]. BIANA uses a high-level abstraction schema to integrate and define external repositories or databases compiling interactomics and protein-ligand (small chemical) information. The two unique features of BIANA are: (i) its unification protocol (i.e. a set of rules defined by the users that determine how data from various sources is combined) offering also the possibility of cross-checking data across different databases; and (ii) its traceability: merged entities can always be traced back to its original source.

GUILD: Network-based disease gene-prioritization predictions

GUILD is a network-based tool to predict and rank genes linked to biological processes and disease phenotypes by combining experimental data and graph-based guilt-by-association algorithms [16]. The hypothesis of these methods is that, for a given phenotype and a set of core genes (e.g. known disease-genes or user-defined genes), the interactors of these genes can also

be associated with the same phenotype. Based on this principle, GUILD predicts and ranks potential novel genes associated with the given phenotype based on the connectedness to a core set of genes through the underlying PPI network. GUILD uses BIANA to create an integrated knowledge base of protein-coding genes, their functional and disease annotations and the interactions between them. GUILD combines the results of three algorithms based on the message passing of information (NetScore and NetZcore) and the shortest paths between a gene and the set of core genes associated with a disease (NetShort).

“Guilt-by-association” strategy to tackle the incompleteness of disease-gene relationships

A common approach to establish relationships between diseases is finding disease-related genes that are common to both diseases[17,18]. To investigate disease-disease relationships originating from shared genes, we used information of 3084 diseases annotated in DisGeNET v1.0 database (DGN1), yielding 4,753,986 possible disease pairs.

We used GUILD [16,19], a network-based disease-gene prioritization tool based on guilt-by-association to extend the number of potential genes associated to a given disease. GUILD exploits the underlying PIN to rank all the genes in the network in respect to their topological closeness to the set of disease-associated genes (*seeds*). GUILD has previously been used to identify novel candidate genes in biological and pathological processes such as apoptosis[20] and brain and lung metastases [21]. In this project, we studied whether GUILD predictions could reduce our lack of completeness in two issues: (i) expand the knowledge on existing relationships, i.e. diseases with common known genes, by including new common genes; and (ii) unveil novel links between diseases which would have been otherwise hidden considering only known genes, i.e. by using only annotated data in DisGeNET database.

Accordingly, we compared potential comorbidities based on disease-gene annotation, i.e. those obtained through common genes from DGN1 only, with relationships found after expanding DGN1 disease-associated genes with GUILD. We performed two independent tests: 1) DGN1-GUILD test, that compares potential comorbidities based on disease-annotation with the relationships obtained after expanding original disease-disease associations (DDAs) with GUILD (Figure S2A panel); and 2) Expanded-DGN1-GUILD test, where the overlap was obtained with the extended set of genes but discarding the shared *seeds* before expansion (Figure S2B panel). The significance of the predictions of DDAs was calculated in terms of recovery of genes linked to the partner disease, using a p-value derived from a hypergeometric distribution (p-value < 0.05):

$$p-value=\sum_{i=k}^{i=n}\frac{\binom{n}{i}\binom{N-n}{K-i}}{\binom{N}{K}}$$ [Eqn 1]

where *N* is the sample size, *K* is the number of genes associated to the disease dis2 as per DGN1, *n* is the size of the set predicted for dis1 (seeds from DGN1 and extended set, i.e. genes with a predicted Z-score > 2), *k* is the number of correct predictions (i.e. number of genes of K set within n, i.e. intersection between K and n).

To address the first issue above (i), i.e. expanding our knowledge on existing DDAs, we studied GUILD predictions with DGN1-GUILD test. To address the second issue (ii), i.e. unveil novel links between diseases, we used the Expanded-DGN1-GUILD test. It must be noted that, in this test, predictions were performed using orthogonal seed genes, i.e. genes that are unique to either of the diseases. Besides, p-values of association between diseases were significant in both directions, e.g. in the test between two diseases, A and B, the enrichment of genes associated with disease B was obtained with orthogonal genes of disease A used as *seeds*, and *vice-versa* (Figure S2).

Quantifying disease relationships using shared genes and gene functions

To quantify the DDAs, a composite score based on the number of common genes and gene-functions was devised. The composite score was formed by seven independent measures: (i) genetic measure of Common Genes (*CG)*; (ii) Functional measure of Common Genes through Reactome Pathways *(FCG-RP)*, (iii) through Gene Ontology biological processes *(FCG-GObp),* (iv) through Gene Ontology molecular functions (*FCG-GOm)f;* (v) Functional measure of Common Functions through Reactome Pathways *(FCF-RP),* (vi) through Gene Ontology biological processes *(FCF-GObp)*; and (vii) through Gene Ontology molecular functions *(FCF-GOmf*). These seven measures were used to assess the statistical significance of the overlap between the DDAs using the updated version of DisGeNET (DGN2) as a reference. First, for each pair of diseases, GUILD predictions were derived as described before using annotated genes in DGN1 as seeds. Subsequently, the overlap of: genes (CG), gene-related functions (FCG-RP, FCG-GObp, FCG-GOmf) and functions (FCF-RP, FCF-GObp, FCF-GOmf) in the extended set were compared to curated sets in DGN2. And last, a p-value was computed as follows:

(i) *For CG measure*: The significance of the relationship between a given pair of diseases was considered by looking at the overlap between genes (Figure 1C). In this case, the variables N, K, n and k in Eqn 1 represent: the size of PIN of both diseases (i.e. number of nodes associated to both diseases including seeds and expanded set); total number of genes annotated in DGN2 that are common to both diseases; number predicted genes shared

between both diseases excluding the seed genes, i.e. GUILD expanded set; and the number of correct predictions, i.e. intersection between K and n (Figure 1C)

(ii) *For FCG-RP, FCG-GObp, and FCG-GOmf measures*: Links between diseases were computed as in CG but considering the functions of the genes instead of the genes themselves (Figure 1D). Thus, genes were converted into function descriptors using three different sources of functional annotations1) Reactome database [22]: FCG-RP; 2) GO [23] term describing the biological function: FCG-GObp; and 3) GO term on molecular function: FCG-GOmf. In this case, the variables N, K, n and k in Eqn 1 represent: all functions represented in both diseases; functions of the genes annotated in DGN2 that are common to both diseases; functions shared between common genes in both diseases excluding those deriving from the seed genes; and the number of correct predictions, i.e. intersection between K and n.

(iii) *For FCF-RP, FCF-GObp, and FCF-GOmf measures:* The final level of abstraction to compute the significance of links between diseases is based on common functions (Figure 1E). It is comparable to *FCG-RP, FCG-GObp, FCG-GOmf* but instead of looking at the overlap of functions induced by common genes, the relationship between diseases is derived from the overlap of functions induced by the disease-associated genes of the two diseases. For each pair of diseases, functions represented in these diseases were derived and compared to those derived from the annotated sets in DGN2. Thus, the variables N, K, n and k in Eqn 1 represent: all functions represented in both diseases; functions of the genes annotated in DGN2; functions shared between both diseases excluding those deriving from the seed genes; and the number of correct predictions, i.e. intersection between K and n.

Note that in (ii) and (iii), we apply the hypergeometric test twice, first to identify functions induced by common genes and second to identify the significance of the overlap between these functions. During functional enrichment calculations, we only considered significantly enriched functions, that is, those with corrected p-value < 0.05.

We tried two multiple testing correction strategies, Bonferroni and FDR (Benjamini Hochberg), to identify significantly linked DDAs (corrected p-value < 0.05). Depending on the multiple testing correction considered, we identify disease-disease associations with strict (Bonferroni) or relaxed (FDR) criteria. A DDA will have a score between 0 and 7, named composite score, being 0 for no-association and 1-7 for the number of significant measures.

Clustering diseases of the disease network.

Once the DDAs were identified, a disease network (DN) was derived using the DDAs identified according to strict criterion. Diseases (nodes) were connected based on the composite score (edges, see above). The idea behind deriving a DN was to facilitate the complex analysis between groups of diseases. With this analysis, we gain further understanding on the interplay between diseases and relationships among them, as the DN provides a more comprehensive representation than isolated pair-wise relationships. The next level of abstraction was to cluster the diseases by identifying highly connected modules within the network. Accordingly, the weighted adjacency matrix representing the DN was clustered using the network-based Markov Cluster Algorithm (MCL) [24] with default parameters.

The DN was also characterized in terms of betweenness centrality of the edges. *Edge betweenness centrality* was assigned to disease-disease links by using the *Networkx* python package [25]. This topological measure used in graph theory informs on the role of a given edge as a gatekeeper of the information flow in the network[26]. In the context of the DN, a high edge betweenness suggests a central role in the relationship between two diseases. To find which of the seven measures produced the higher betweenness-centrality values, we compared the betweenness centrality distribution of each single measure with the remaining six approaches. Then, we used a Kolmogorov-Smirnov test of the distributions to confirm the significance of the measure.

Mapping mutations to protein interfaces

To further understand the molecular basis between two linked diseases, we characterized the interactions between pairs of proteins produced by the genes linking them in the DN and mapped missense mutations extracted from: SNPdbe[27], the Human Polymorphisms and Disease Mutations database at UniProt [9], the ClinVar database [28], the Genetic Association Database [29] and the NHGRI GWAS Catalog [30].

For any given pair of diseases, three different types of links were defined by considering the types of interactions between proteins (i.e. gene products) associated with each disease: (i) direct; (ii) semi-direct and (iii) indirect.

We compared the betweenness centrality of the edges of the DN and the type of disease-disease links (i.e. indirect, semi-direct and direct*)* using the Spearman's correlation coefficient. Indirect links had the only significant correlation with betweenness centrality in the DN (r = 0.20, p-value = 0.004). On the other hand, the correlations between centrality and semi-direct links (r = 0.047, p-

value = 0.51) and between centrality and direct links (r = -0.09, p-value = 0.195) were not significant.

Whenever possible, we characterized the mutations linked to protein interfaces by using the structure of protein complexes from 3DiD [31]. In the case of protein complexes with known structure, the interface was defined as the set of residues of each protein at less than 12 Angstroms Cα-Cα distance, as defined in 3DiD. For complexes of proteins highly similar to the known interactions (usually referred as interologs), we inferred the residues in the interface from the alignment with the corresponding template structure using Align [32].

Assigning diseases to a unique disease MeSH category

We used a heatmap to show the pairs of significantly associated diseases. For the sake of the representation, we manually ordered the diseases represented according to MeSH disease classification tree. Since a disease can belong to more than one MeSH category, we manually created a hierarchical order to assign a unique MeSH category to each disease. The hierarchy considers the relevance of diseases in the MeSH order of categories and it is defined as follows:

1) Cancer related diseases:
    - Neoplasms (C04)
2) General system Diseases:
    - Musculoskeletal Diseases (C05)
    - Digestive System Diseases (C06)
    - Respiratory Tract Diseases (C08)
    - Nervous System Diseases (C10)
    - Male Urogenital Diseases (C12)
    - Female Urogenital Diseases and Pregnancy Complications (C13)
    - Cardiovascular Diseases (C14)
    - Endocrine System Diseases (C19)
    - Hemic and Lymphatic Diseases (C15)
3) Minor general system/organ:
    - Otorhinolaryngologic Diseases (C09)
    - Eye Diseases (C11)
    - Skin and Connective Tissue Diseases (C17)
    - Immune System Diseases (C20)
    - Stomatognathic Diseases (C07)
    - Mental Disorders (F03)
4) Classes related to disease aetiology:

Nutritional and Metabolic Diseases (C18)
Bacterial Infections and Mycoses (C01)
Virus Diseases (C02)
Parasitic Diseases (C03)
Disorders of Environmental Origin (C21)
Substance-Related Disorders (C25)

5) Other:

Occupational Diseases (C24)
Wounds and Injuries (C26)
Congenital, Hereditary, and Neonatal Diseases and Abnormalities (C16)
Pathological Conditions, Signs and Symptoms (C23)
Behavior and Behavior Mechanisms (F01)
Psychological Phenomena and Processes (F02)
Behavioral Disciplines and Activities (F04)

For example, if a disease is associated to C16 MeSH category and C04 MeSH category, we manually classified this disease as C04.

SUPPLEMENTARY FIGURES

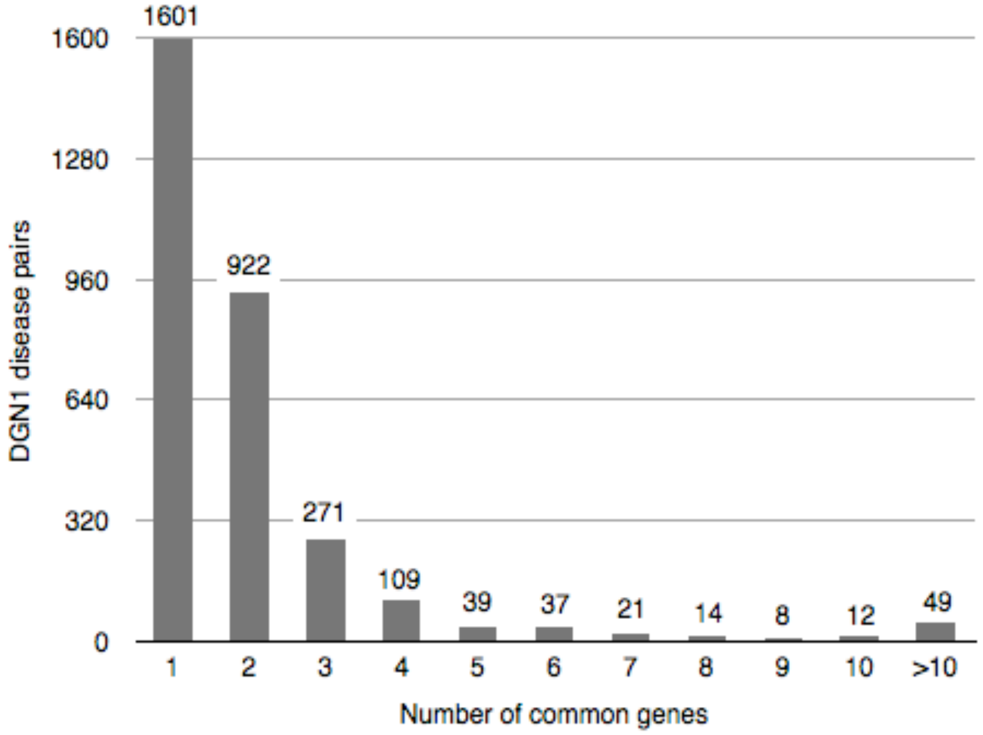

Figure S1. Histogram of the number of DDAs that can be explained in DGN1 data according to the number of shared genes.

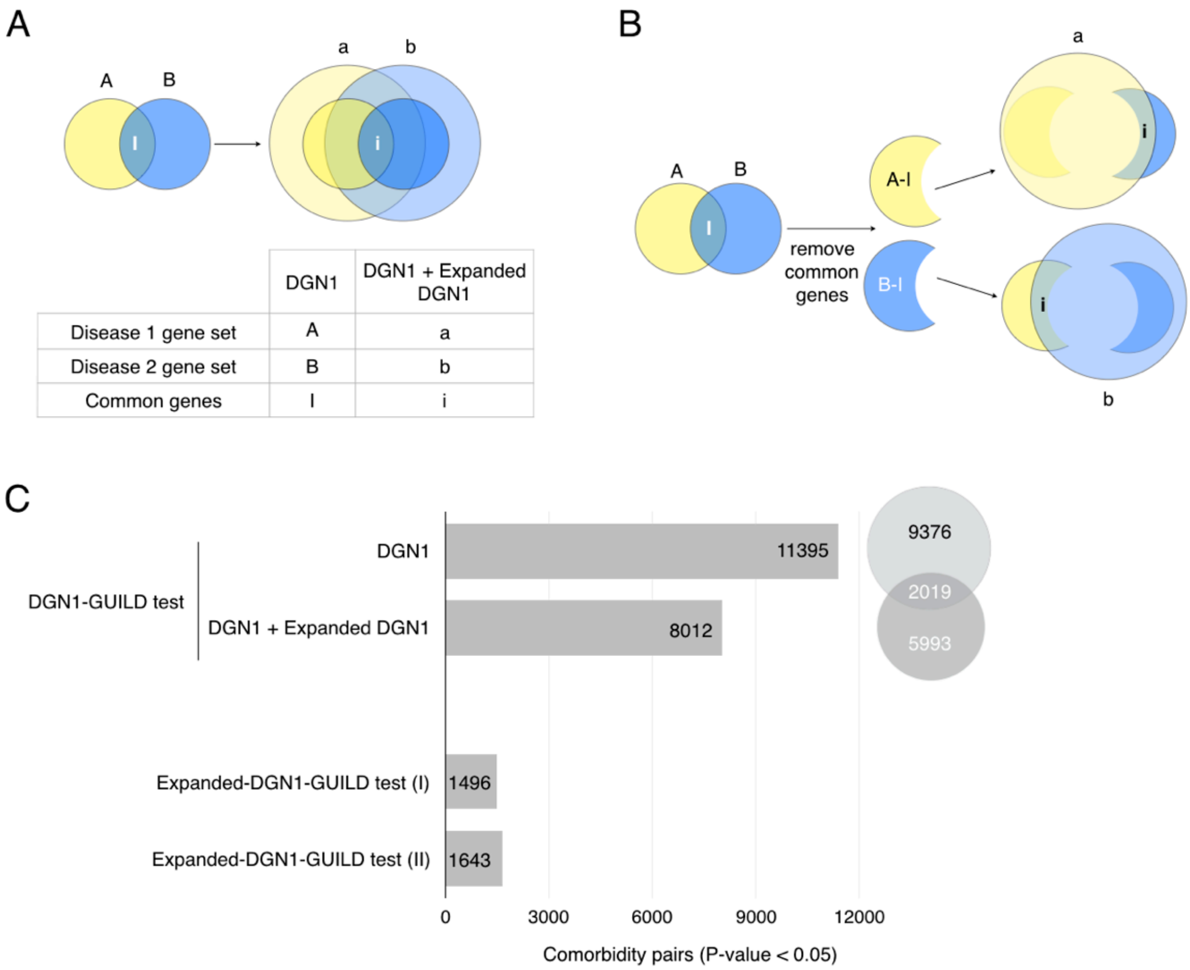

Figure S2. Validation of GUILD approach in the discovery of disease-disease links.

Circles represent the set of genes associated to two different diseases A and B retrieved from DGN1 or from DGN1 plus genes expanded with GUILD approach (a and b), see the embeded table. **(A)** The DGN1-GUILD test. **(B)** The Expanded-DGN1-GUILD test. Note that in this latter test, all common DGN1-genes of A and B were removed (A-I and B-I). **(C)** Results of DGN1-GUILD test and Expanded-DGN1-GUILD test considering the expansion of A-I in a disease pair (I) or the expansion of B-I in the same disease pair (II). In DGN1-GUILD test (DGN1) all possible disease-disease associations within DGN1 have been considered while in DGN1 + Expanded DGN1 only associations between the 259 diseases for which we ran GUILD were considered.

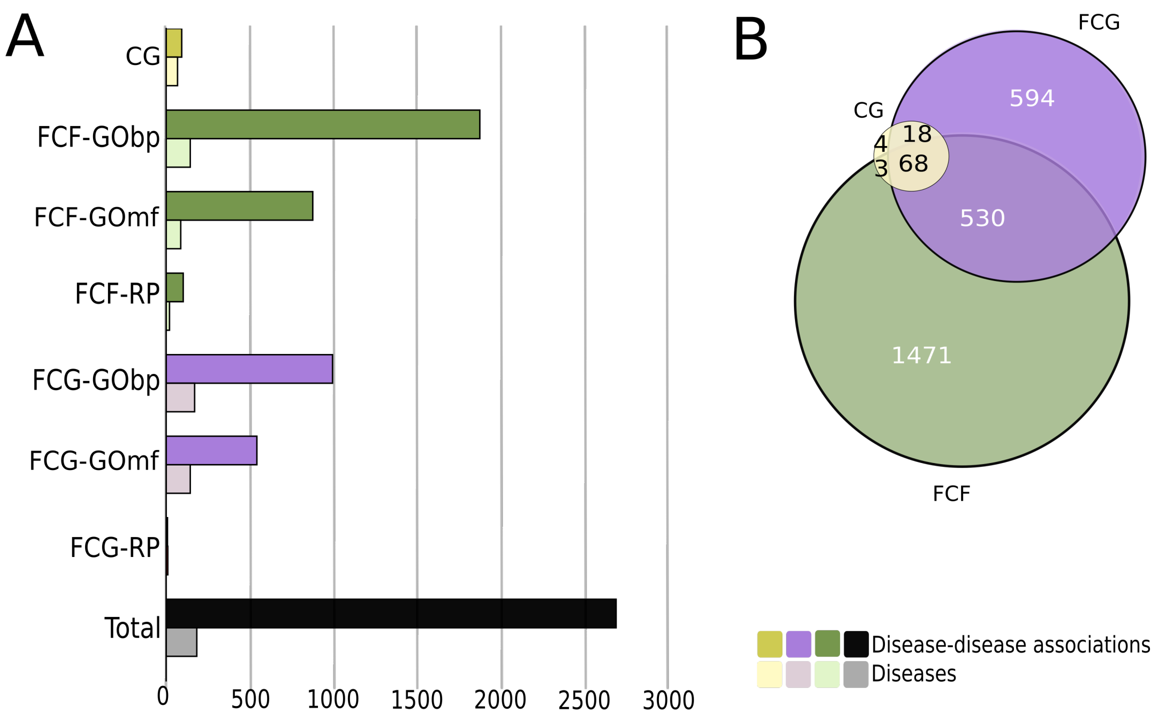

Figure S3. Number of DDAs found by the relaxed criterion.

(A) Light bars represent the number of diseases considered in each measure. Dark bars represent the number of disease-disease associations with respect to the strict criterion (CG in yeast colour, FCG in purple and FCF in green). The total number of associations obtained (by at least one of the measures) are shown in black.

(B) Venn diagram of the sets of associations found by the CG, FCG and FCG measures.

A

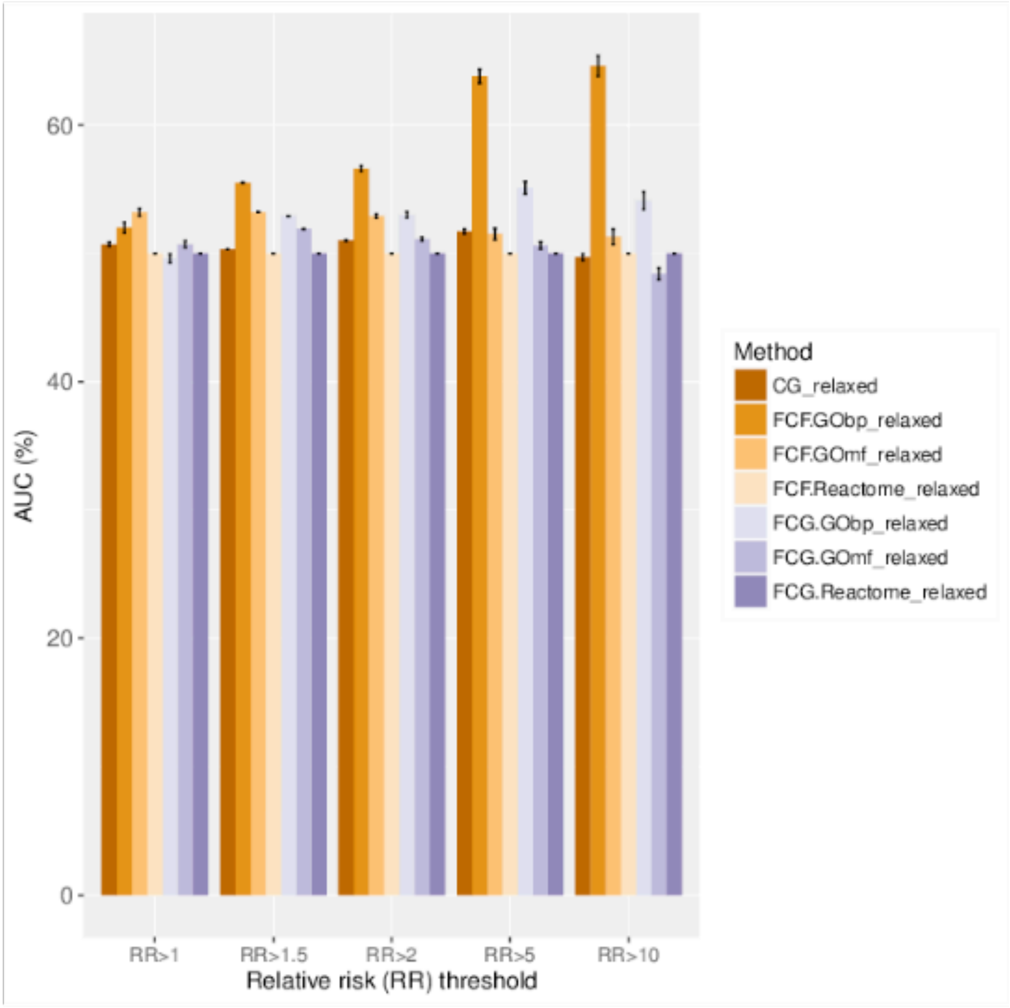
60
40
20
0
AUC (%)
RR>1
RR>1.5
RR>2
RR>5
RR>10
Relative risk (RR) threshold
Method
CG_relaxed
FCF.GObp_relaxed
FCF.GOmf_relaxed
FCF.Reactome_relaxed
FCG.GObp_relaxed
FCG.GOmf_relaxed
FCG.Reactome_relaxed

B

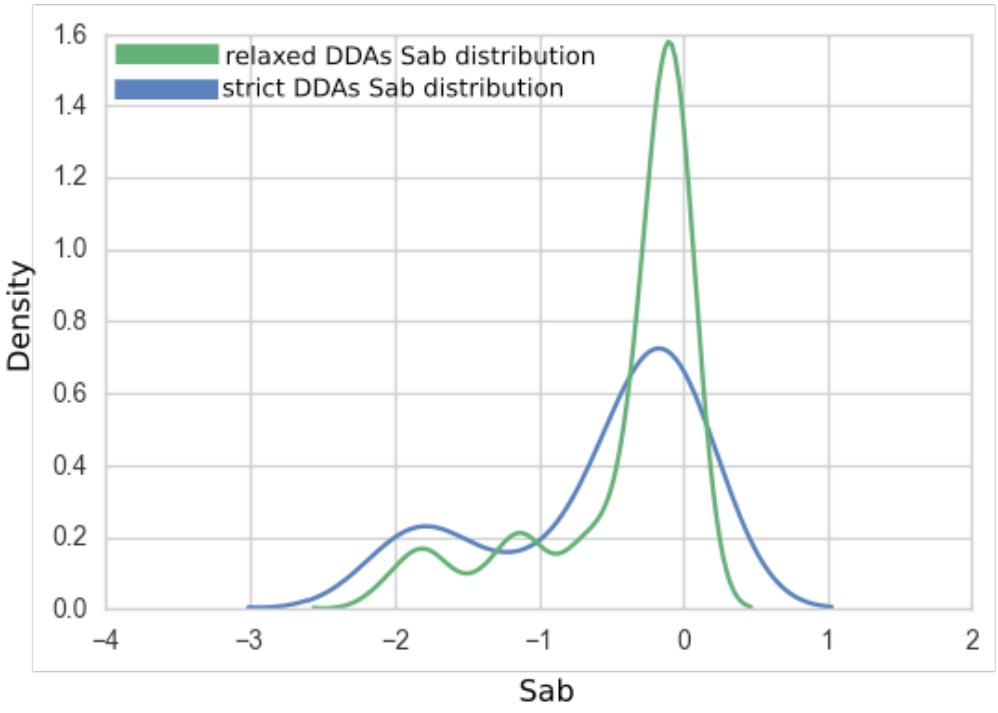
relaxed DDAs Sab distribution
strict DDAs Sab distribution
Density
1.6
1.4
1.2
1.0
0.8
0.6
0.4
0.2
0.0
−4
−3
−2
−1
0
1
2
Sab

Figure S4. Comparison with previous work.

(A) Prediction accuracy of genetic and overlap based measures in characterizing disease comorbidities. The bars show the area under ROC curve (AUC) using the disease-disease associations reported previously by Hidalgo et al., at varying relative risk (RR) thresholds as the gold standard. For each RR threshold, we calculate the true positive rate and false positive rate over various prediction score cutoffs to generate a ROC curve and calculated the area under ROC curve. We randomly sample among the unknown (negative) associations to balance the number of positive associations with the number of negative associations, and repeat the procedure 100 times to have robust estimates for mean AUC. The bars represent mean AUC and the error bar correspond to the standard error over 100 runs. The measures are genetic overlap (CG_relaxed), functional overlap using GO biological processes (FCF.GObp_relaxed), GO molecular functions (FCF.GOmf_relaxed), Reactome pathways (FCF.Reactome_relaxed) and functional overlap using functions of common genes from the same sources (FCGGObp_relaxed, FCGGOmf_relaxed, FCGReactome_relaxed) all based on relaxed criterion.

(B) Kernel density estimate for the distribution of Sab (Menche et al) of mapped DDAs identified in this study by means of strict (blue) and relaxed (green) criteria.

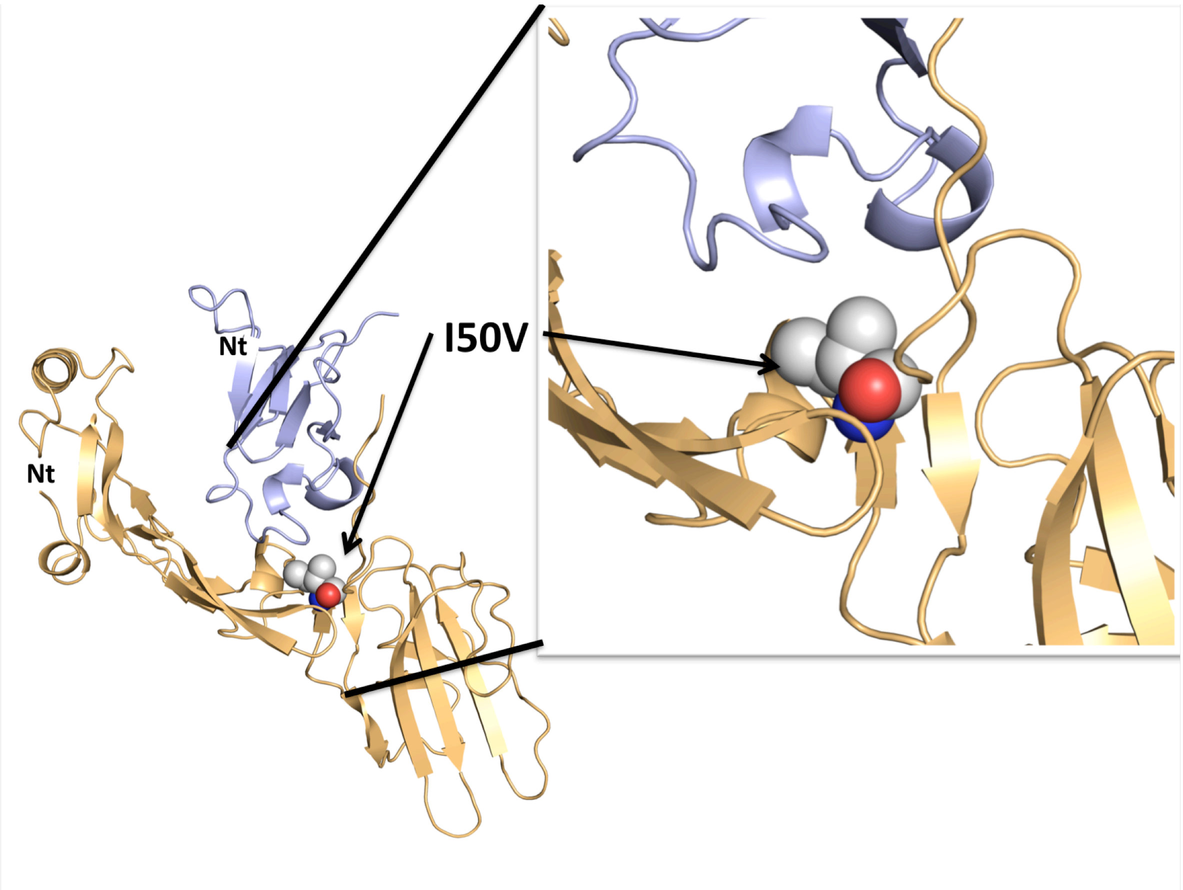

Figure S5. Structural details of the TGF-ß receptor type-2 and the TGF ß-3.

Cartoon representation of TGF-ß receptor type-2 (pale yellow) interacting with the transforming growth factor ß-3 (pale blue). Mutated residue, I50V in the TGF-ß receptor type-2 (i.e. I73 in the full sequence from UniProt), is shown in ball-and-stick representation with each atom coloured according to type: red, blue and white for oxygen, nitrogen and carbon respectively. Inset portraits a close-up on the interface region and mutated residue.

SUPPLEMENTARY TABLES

Table S1. Number of genes associated to each disease. Number of genes associated to each disease (see filtering steps in Figure 1A and Methods): according to DGN1, according to DGN1 plus its GUILD expansion (exploratory set) and according to DGN2 but not in DGN1 (validation set).

Table S2. Details of the DDAs by means of strict and relaxed criteria.

Tables with the details of all disease-disease associations with k > 1 (see Methods Eqn 1). Each table shows: the disease-disease association (through its disease mesh IDs separated by comma), the coefficients of the final hypergeometric distribution (x , m, N and k) of each measure (see Methods), the uncorrected P-value of the association (PVAL), the corrected P-value (using Bonferroni or FDR correction, see Methods) (PVAL_c) and a Boolean choice (TRUE/FALSE) for the significance of the corrected P-value (PVAL_c_SIG) (True if PVAL_c > 0.05). Finally, disease-disease associations by FCG and FCF approach show in the last column the function association type (by Reactome or GO, labelled as "SOURCE").

Table S2A: CG disease-disease associations (strict).

Table S2B: CG disease-disease associations (relaxed).

Table S2C: FCG disease-disease associations (strict).

Table S2D: FCG disease-disease associations (relaxed).

Table S2E: FCF disease-disease associations (strict).

Table S2F: FCF disease-disease associations (relaxed).

Table S3. DDAs by strict and relaxed criteria.

List of all DDAs by means of strict (Table S3A) and relaxed criteria (Table S3B). Each column contains the corrected p-value of association for each of the seven measures used (CG, FCF-GObp, FCF-GOmf, FCF-Reactome, FCG-GObp, FCG-GOmf and FCG-Reactome). The last column of each table shows the composite score of the seven measures (the total number of measures through which both diseases were significantly associated).

Table S4. Comparison of significant measures versus distance Sab criterion by Menche et al.

The Sab values for pairs of diseases with significant measures under the strict (S4A) and relaxed (S4B) criteria (Sab as defined by Meche et al. [33].). The first two columns show the MesH IDs of the disease-disease pair. Sab column shows the separation of both diseases according to Menches' study and the last column shows the names of the measures at which we have found a significant association of both diseases.

Table S5. Analysis of edges connecting diseases and clusters of diseases.

Table S5A: Pairs of clusters (including self-associations) within the disease-network (DN), showing the number of direct, semi-direct and indirect associations between diseases. The first two columns show the name and numbering of the cluster and the last three columns the numbers of each association-type.

Table S5B: The mutations on the interafaces of the interacting proteins associated with disease pairs. The table gives the DDAs predicted with our approach (composite score between 1 and 7) and protein-protein interactions associated with them, along with the number of mutations located in the interface of the interaction (and/or the PFAM domain involved in the interaction). Columns "disease 1" and "disease 2" show the names of the two diseases with putative comorbidity. The first columns "cluster 1" and "cluster 2" show the name of the cluster to which diseases 1 and 2 belong, respectively. Columns "Ac1" and "Pfam1" show the accession numbers and PFAM domains of the proteins associated with disease 1, while "Ac2" and "Pfam2" are for disease 2. The classification of the interaction as indirect, direct or semi-direct is shown in column "type". Column "mut1" indicates the number of mutations located in the interface of the first protein (Ac1), and column "assoc1" shows how many of them are associated with a disease. Columns mut2 and assoc2 are defined as mut1 and assoc1 but for the second protein (Ac2). Clusters formed by less than three diseases are also included. In addition to the names given to the clusters, they are also numbered to include the additional clusters, up to a total of 8 clusters.

Table S5C: The same as S5B but only for pairs for which mutations can be assigned to the corresponding disease (i.e. assoc1 with disease 1 and assoc2 with disease 2). We have highlighted in orange the pairs involving asthma and arthritis rheumatoid when the mutation alters the TNF binding, as many works are recently studying the use of anti-TNF drugs for both diseases[34]. For the rest of pairs, the association is done by searching any of the mesh terms associated with the phenotype described in ClinVar or humsavar. Highlighted in yellow are shown the pairs with at least one exact match, otherwise the match can be with any of the parents or child terms in the MeSH tree. The list of mutations used to generate Table S5B and S5C is also given as a text file ("Dataset_S5").

Table S6. Functional relationship between Alzheimer diseases and cancer. Table S5A: Enrichment of GO terms by DDAs found through FCF method. Table S5B) Enrichment of GO terms by DDAs found through FCG method. GO terms related to apoptosis have been highlighted in yellow.

SUPPLEMENTARY REFERENCES

1 Garcia-Garcia, J., Guney, E., Aragues, R., Planas-Iglesias, J. & Oliva, B. Biana: a software framework for compiling biological interactions and analyzing networks. *BMC Bioinformatics* **11**, 56, doi:1471-2105-11-56 [pii] 10.1186/1471-2105-11-56 (2011).

2 Keshava Prasad, T. S. *et al.* Human Protein Reference Database--2009 update. *Nucleic Acids Res* **37**, D767-772 (2009).

3 Salwinski, L. *et al.* The Database of Interacting Proteins: 2004 update. *Nucleic Acids Res* **32**, D449-451 (2004).

4 Mewes, H. W. *et al.* MIPS: curated databases and comprehensive secondary data resources in 2010. *Nucleic Acids Res* **39**, D220-224, doi:gkq1157 [pii]10.1093/nar/gkq1157 (2010).

5 Chatr-Aryamontri, A. *et al.* The BioGRID interaction database: 2015 update. *Nucleic Acids Res* **43**, D470-478, doi:gku1204 [pii] 10.1093/nar/gku1204 (2015).

6 Bader, G. D., Betel, D. & Hogue, C. W. BIND: the Biomolecular Interaction Network Database. *Nucleic Acids Res* **31**, 248-250 (2003).

7 Kerrien, S. *et al.* The IntAct molecular interaction database in 2012. *Nucleic Acids Res* **40**, D841-846 (2012).

8 Licata, L. *et al.* MINT, the molecular interaction database: 2012 update. *Nucleic Acids Res* **40**, D857-861 (2012).

9 Wu, C. H. *et al.* The Universal Protein Resource (UniProt): an expanding universe of protein information. *Nucleic Acids Res* **34**, D187-191, doi:34/suppl_1/D187 [pii]10.1093/nar/gkj161 (2006).

10 Maglott, D., Ostell, J., Pruitt, K. D. & Tatusova, T. Entrez Gene: gene-centered information at NCBI. *Nucleic Acids Res* **39**, D52-57, doi:gkq1237 [pii10.1093/nar/gkq1237 (2010).

11 Pinero, J. *et al.* DisGeNET: a discovery platform for the dynamical exploration of human diseases and their genes. *Database (Oxford)* **2015**, doi:bav028 [pii]10.1093/database/bav028 (2015).

12 Bauer-Mehren, A., Rautschka, M., Sanz, F. & Furlong, L. I. DisGeNET: a Cytoscape plugin to visualize, integrate, search and analyze gene-disease networks. *Bioinformatics* **26**, 2924-2926, doi:btq538 [pii] 10.1093/bioinformatics/btq538 (2010).

13 Bodenreider, O. The Unified Medical Language System (UMLS): integrating biomedical terminology. *Nucleic Acids Res* **32**, D267-270, doi:10.1093/nar/gkh061 32/suppl_1/D267 [pii] (2004).

14 Lipscomb, C. E. Medical Subject Headings (MeSH). *Bull Med Libr Assoc* **88**, 265-266 (2000).
15 Amberger, J., Bocchini, C. A., Scott, A. F. & Hamosh, A. McKusick's Online Mendelian Inheritance in Man (OMIM). *Nucleic Acids Res* **37**, D793-796 (2009).
16 Guney, E. & Oliva, B. Exploiting protein-protein interaction networks for genome-wide disease-gene prioritization. *PLoS One* **7**, e43557, doi:10.1371/journal.pone.0043557
PONE-D-12-08079 [pii] (2012).
17 Goh, K. I. C., M.E.; Valle, D.; Childs, B.; Vidal, M. & Barabasi, A.L. The human disease network. *Proceedings of the National Academy of Sciences* **104**, 8685 (2007).
18 van Driel, M. A., Bruggeman, J., Vriend, G., Brunner, H. G. & Leunissen, J. A. A text-mining analysis of the human phenome. *Eur J Hum Genet* **14**, 535-542, doi:5201585 [pii]
10.1038/sj.ejhg.5201585 (2006).
19 Guney, E., Garcia-Garcia, J. & Oliva, B. GUILDify: a web server for phenotypic characterization of genes through biological data integration and network-based prioritization algorithms. *Bioinformatics* **30**, 1789-1790, doi:btu092 [pii]
10.1093/bioinformatics/btu092 (2014).
20 Planas-Iglesias, J. *et al.* Extending signaling pathways with protein-interaction networks. Application to apoptosis. *OMICS* **16**, 245-256, doi:10.1089/omi.2011.0130 (2012).
21 Engin, H. B., Guney, E., Keskin, O., Oliva, B. & Gursoy, A. Integrating structure to protein-protein interaction networks that drive metastasis to brain and lung in breast cancer. *PLoS One* **8**, e81035, doi:10.1371/journal.pone.0081035
PONE-D-13-25934 [pii] (2013).
22 Croft, D. *et al.* Reactome: a database of reactions, pathways and biological processes. *Nucleic Acids Res* **39**, D691-697 (2011).
23 Berardini, T. Z., Khodiyar, V. K., Lovering, R. C. & Talmud, P. J. The Gene Ontology in 2010: extensions and refinements. *Nucleic Acids Res* **38**, D331-335 (2010).
24 Enright, A. J., Van Dongen, S. & Ouzounis, C. A. An efficient algorithm for large-scale detection of protein families. *Nucleic Acids Res* **30**, 1575-1584 (2002).
25 Hagberg, A., Schult, D. & Swart, P. in *Proceedings of the 7th Python in Science Conference (SciPy2008)*
26 Girvan, M. & Newman, M. E. Community structure in social and biological networks. *Proc Natl Acad Sci U S A* **99**, 7821-7826, doi:10.1073/pnas.122653799
99/12/7821 [pii] (2002).
27 Schaefer, C., Meier, A., Rost, B. & Bromberg, Y. SNPdbe: constructing an nsSNP functional impacts database. *Bioinformatics* **28**, 601-602, doi:btr705 [pii]10.1093/bioinformatics/btr705 (2012).
28 Landrum, M. J. *et al.* ClinVar: public archive of relationships among sequence variation and human phenotype. *Nucleic Acids Res* **42**, D980-985, doi:gkt1113 [pii]10.1093/nar/gkt1113 (2014).
29 Zhang, Y. *et al.* Systematic analysis, comparison, and integration of disease based human genetic association data and mouse genetic phenotypic information. *BMC Med Genomics* **3**, 1, doi:1755-8794-3-1 [pii]10.1186/1755-8794-3-1 (2010).
30 Welter, D. *et al.* The NHGRI GWAS Catalog, a curated resource of SNP-trait associations. *Nucleic Acids Res* **42**, D1001-1006, doi:gkt1229 [pii]10.1093/nar/gkt1229 (2013).
31 Mosca, R., Ceol, A., Stein, A., Olivella, R. & Aloy, P. 3did: a catalog of domain-based interactions of known three-dimensional structure. *Nucleic Acids Res* **42**, D374-379, doi:gkt887 [pii]10.1093/nar/gkt887 (2014).
32 Myers, E. W. & Miller, W. Optimal alignments in linear space. *Comput Appl Biosci* **4**, 11-17 (1988).
33 Menche, J. *et al.* Disease networks. Uncovering disease-disease relationships through the

incomplete interactome. *Science* **347**, 1257601, doi:347/6224/1257601 [pii]10.1126/science.1257601 (2015).

34 Sieberts, S. K. *et al.* Crowdsourced assessment of common genetic contribution to predicting anti-TNF treatment response in rheumatoid arthritis. *Nat Commun* **7**, 12460, doi:10.1038/ncomms12460 (2016).
