# Supplementary material for: Genetic and functional characterization of disease associations explains comorbidity
Source: Sci Rep. 2017 Jul 24;7:6207. doi: 10.1038/s41598-017-04939-4 (PMC5524755; doi:10.1038/s41598-017-04939-4)
Supplement: Supplementary file 8 — Dataset_S5 [file 41598_2017_4939_MOESM8_ESM.doc]

######## DDA with STRICT approach ###########################################

#Function: calc_interface_clusters with pairs with file: TableS3_strict.tsv #

#Total mutations: 299866 #

##############################################################################

MESH1 mesh:D000230

NAME1 Adenocarcinoma

CLUSTER1 clust9

MESH2 mesh:D004938

NAME2 Esophageal Neoplasms

CLUSTER2 clust9

AC1 Q15831

PFAM1 PF00069

PDB1 2WTK:C

AC2 Q7RTN6

PFAM2 PF00069

PDB2 2WTK:C

INT semi-edgetic

AA1 106R;Interacts with:110E

AA1 133V;Interacts with:253L

AA1 186T;Interacts with:253L,252N,231H,251Q

AA1 187G;Interacts with:231H

AA1 50L;Interacts with:285T

AA1 67L;Interacts with:288L,285T

MUT1 L67Afs;clinvar:22490, phenotype=Peutz-Jeghers syndrome;PEUTZ-JEGHERS SYNDROME;

MUT1 L67P;955903;humsavar:VAR_006202, phenotype=Peutz-Jeghers syndrome (PJS);clinvar:22484, phenotype=Peutz-Jeghers syndrome;PEUTZ-JEGHERS SYNDROME;

AA1 69S;Interacts with:284A,285T

AA1 70E;Interacts with:238V,241L,284A

AA1 71T;Interacts with:251Q,241L,238V,233F,237S

AA1 72L;Interacts with:251Q,233F,285T,249L,284A,241L,288L

AA1 73C;Interacts with:233F,251Q

AA1 74R;Interacts with:250Q,251Q

MUT1 R74A;1375192;

AA2 110E;Interacts with:106R

AA2 231H;Interacts with:186T,187G

MUT2 H231A;1375241;

AA2 233F;Interacts with:72L,73C,71T

MUT2 F233A;1375242;

AA2 237S;Interacts with:71T

MUT2 S237G;dbSNP:rs141993862;dbSNP:rs141993862;dbSNP:rs141993862;

AA2 238V;Interacts with:70E,71T

AA2 241L;Interacts with:71T,72L,70E

MUT2 L241A;1375243;

AA2 249L;Interacts with:72L

AA2 250Q;Interacts with:74R

AA2 251Q;Interacts with:74R,71T,72L,73C,186T

MUT2 Q251A;1375245;

AA2 252N;Interacts with:186T

AA2 253L;Interacts with:186T,133V

AA2 284A;Interacts with:69S,72L,70E

AA2 285T;Interacts with:67L,69S,72L,50L

AA2 288L;Interacts with:67L,72L

//

MESH1 mesh:D000230

NAME1 Adenocarcinoma

CLUSTER1 clust9

MESH2 mesh:D004938

NAME2 Esophageal Neoplasms

CLUSTER2 clust9

AC1 Q7RTN6

PFAM1 PF00069

PDB1 2WTK:B

AC2 Q9Y376

PFAM2 PF08569

PDB2 2WTK:B

INT semi-edgetic

AA1 104L;Interacts with:223Y,227R,269N,224V

AA1 105E;Interacts with:268R,269N,267S,227R,223Y

AA1 107C;Interacts with:269N,227R

AA1 108S;Interacts with:227R

AA1 109N;Interacts with:227R,231K,273E

MUT1 N109S;dbSNP:rs144919702;dbSNP:rs144919702;dbSNP:rs144919702;

AA1 113T;Interacts with:228Q

AA1 116Q;Interacts with:228Q,182S

AA1 119L;Interacts with:178F,179D

AA1 120H;Interacts with:183D,141L,145I

AA1 123K;Interacts with:141L,179D,92F

AA1 124L;Interacts with:141L,92F,96K,145I

AA1 125F;Interacts with:92F

AA1 126N;Interacts with:93E,92F

AA1 136T;Interacts with:179D,178F,177T

AA1 137F;Interacts with:178F,176S

AA1 138I;Interacts with:224V,176S,178F,222N

AA1 140D;Interacts with:223Y,224V,222N

AA1 141N;Interacts with:223Y

AA1 143L;Interacts with:178F

AA1 185Y;Interacts with:96K

MUT1 Y185F;1375244;

AA1 188H;Interacts with:51K,52E

AA1 221I;Interacts with:55Y

AA1 223H;Interacts with:107R

AA1 224G;Interacts with:55Y,104N

AA1 225Q;Interacts with:55Y,104N

AA1 226R;Interacts with:56G,55Y,57T,58N,60K

AA2 104N;Interacts with:224G,225Q

AA2 107R;Interacts with:223H

AA2 141L;Interacts with:124L,123K,120H

AA2 145I;Interacts with:124L,120H

AA2 176S;Interacts with:138I,137F

AA2 177T;Interacts with:136T

AA2 178F;Interacts with:137F,136T,119L,143L,138I

AA2 179D;Interacts with:136T,123K,119L

AA2 182S;Interacts with:116Q

AA2 183D;Interacts with:120H

AA2 222N;Interacts with:138I,140D

AA2 223Y;Interacts with:140D,141N,104L,105E

AA2 224V;Interacts with:138I,104L,140D

AA2 227R;Interacts with:109N,105E,104L,108S,107C

AA2 228Q;Interacts with:116Q,113T

AA2 231K;Interacts with:109N

AA2 267S;Interacts with:105E

AA2 268R;Interacts with:105E

AA2 269N;Interacts with:105E,104L,107C

AA2 273E;Interacts with:109N

AA2 51K;Interacts with:188H

AA2 52E;Interacts with:188H

AA2 55Y;Interacts with:224G,225Q,226R,221I

AA2 56G;Interacts with:226R

AA2 57T;Interacts with:226R

AA2 58N;Interacts with:226R

AA2 60K;Interacts with:226R

AA2 92F;Interacts with:124L,126N,125F,123K

AA2 93E;Interacts with:126N

AA2 96K;Interacts with:185Y,124L

//

MESH1 mesh:D000544

NAME1 Alzheimer Disease

CLUSTER1 clust2

MESH2 mesh:D002277

NAME2 Carcinoma

CLUSTER2 clust2

AC1 O43521

PFAM1 PF08945

PDB1 2NL9:B

AC2 Q07820

PFAM2 PF00452

PDB2 2NL9:B

INT non-edgetic

AA1 143R;Interacts with:234K

MUT1 R143H;dbSNP:rs141962978;dbSNP:rs141962978;dbSNP:rs141962978;dbSNP:rs141962978;dbSNP:rs141962978;

AA1 144P;Interacts with:234K,235L

AA1 145E;Interacts with:248R,249V,245S,252H,235L

AA1 146I;Interacts with:252H

AA1 147W;Interacts with:230G,231M

AA1 148I;Interacts with:270F,235L,234K,249V,231M

AA1 149A;Interacts with:249V,252H,253V

AA1 151E;Interacts with:231M

AA1 152L;Interacts with:228F,270F,253V,249V,266T,267L,231M

AA1 153R;Interacts with:252H,253V,254F,255S,256D,263R

MUT1 R153W;dbSNP:rs146318804;dbSNP:rs146318804;dbSNP:rs146318804;dbSNP:rs146318804;dbSNP:rs146318804;

AA1 155I;Interacts with:266T,220V,228F,227A,224H,231M

AA1 156G;Interacts with:262G,260N,266T,263R

AA1 157D;Interacts with:260N,263R,262G

AA1 158E;Interacts with:224H

AA1 159F;Interacts with:265V,220V,262G,216V,219G,224H

AA1 160N;Interacts with:261W,262G,260N,263R

AA1 163Y;Interacts with:215R,216V

AA2 215R;Interacts with:163Y

AA2 216V;Interacts with:159F,163Y

AA2 219G;Interacts with:159F

AA2 220V;Interacts with:159F,155I

AA2 224H;Interacts with:158E,155I,159F

AA2 227A;Interacts with:155I

MUT2 A227V;169059;dbSNP:rs11580946;humsavar:VAR_024022;rs11580946;

AA2 228F;Interacts with:152L,155I

AA2 230G;Interacts with:147W

AA2 231M;Interacts with:147W,151E,148I,152L,155I

MUT2 M231L;898693;dbSNP:rs140449444;humsavar:VAR_054157;

AA2 234K;Interacts with:148I,144P,143R

MUT2 K234R;898704;

AA2 235L;Interacts with:148I,145E,144P

AA2 245S;Interacts with:145E

AA2 248R;Interacts with:145E

AA2 249V;Interacts with:149A,152L,145E,148I

AA2 252H;Interacts with:153R,149A,146I,145E

AA2 253V;Interacts with:152L,153R,149A

AA2 254F;Interacts with:153R

AA2 255S;Interacts with:153R

AA2 256D;Interacts with:153R

AA2 260N;Interacts with:156G,157D,160N

AA2 261W;Interacts with:160N

AA2 262G;Interacts with:159F,156G,160N,157D

AA2 263R;Interacts with:160N,156G,153R,157D

AA2 265V;Interacts with:159F

AA2 266T;Interacts with:155I,152L,156G

AA2 267L;Interacts with:152L

AA2 270F;Interacts with:148I,152L

//

MESH1 mesh:D000544

NAME1 Alzheimer Disease

CLUSTER1 clust2

MESH2 mesh:D002277

NAME2 Carcinoma

CLUSTER2 clust2

AC1 O43521

PFAM1 PF08945

PDB1 2K7W:B

AC2 Q07812

PFAM2 PF00452

PDB2 2K7W:B

INT non-edgetic

AA1 145E;Interacts with:146E,142D,89R

AA1 146I;Interacts with:142D

AA1 148I;Interacts with:142D

AA1 149A;Interacts with:138G,142D,139W

AA1 152L;Interacts with:142D,141L,145R,138G

AA1 153R;Interacts with:138G,131E,135T,134R

MUT1 R153W;dbSNP:rs146318804;dbSNP:rs146318804;dbSNP:rs146318804;dbSNP:rs146318804;dbSNP:rs146318804;

AA1 156G;Interacts with:141L,137M,134R

AA1 157D;Interacts with:134R

AA1 160N;Interacts with:134R

AA2 131E;Interacts with:153R

AA2 134R;Interacts with:157D,153R,156G,160N

AA2 135T;Interacts with:153R

AA2 137M;Interacts with:156G

AA2 138G;Interacts with:149A,153R,152L

AA2 139W;Interacts with:149A

MUT2 W139C;COSMIC:267934;

AA2 141L;Interacts with:156G,152L

AA2 142D;Interacts with:145E,149A,152L,148I,146I

AA2 145R;Interacts with:152L

AA2 146E;Interacts with:145E

AA2 89R;Interacts with:145E

MUT2 R89Q;1KG:1191554;1KG:1224249;1KG:1224241;

//

MESH1 mesh:D000544

NAME1 Alzheimer Disease

CLUSTER1 clust2

MESH2 mesh:D002277

NAME2 Carcinoma

CLUSTER2 clust2

AC1 P10415

PFAM1 PF00452

PDB1 4AQ3:A

AC2 Q07817

PFAM2 PF00452

PDB2 4AQ3:C

INT non-edgetic

AA1 102D;Interacts with:100R

AA1 103D;Interacts with:100R,99L,97F

AA1 104F;Interacts with:96E

AA1 106R;Interacts with:96E

AA1 107R;Interacts with:95D,96E,92E,91R

AA1 108Y;Interacts with:92E

AA1 115M;Interacts with:117G

AA1 118Q;Interacts with:116P,115T,117G

AA1 119L;Interacts with:117G

AA1 120H;Interacts with:115T

AA1 129R;Interacts with:117G

MUT1 R129C;828636;

AA1 132T;Interacts with:117G

AA1 133V;Interacts with:117G

AA1 135E;Interacts with:120Y

AA1 136E;Interacts with:120Y

MUT1 E136R;PMID:11461956;

AA1 98R;Interacts with:100R

AA1 99Q;Interacts with:100R,101Y

AA2 100R;Interacts with:103D,102D,99Q,98R

AA2 101Y;Interacts with:99Q

AA2 115T;Interacts with:118Q,120H

AA2 116P;Interacts with:118Q

AA2 117G;Interacts with:132T,129R,115M,133V,119L,118Q

AA2 120Y;Interacts with:135E,136E

AA2 91R;Interacts with:107R

AA2 92E;Interacts with:108Y,107R

AA2 95D;Interacts with:107R

AA2 96E;Interacts with:107R,104F,106R

AA2 97F;Interacts with:103D

AA2 99L;Interacts with:103D

//

MESH1 mesh:D000544

NAME1 Alzheimer Disease

CLUSTER1 clust2

MESH2 mesh:D002277

NAME2 Carcinoma

CLUSTER2 clust2

AC1 P10415

PFAM1 PF02180

PDB1 4AQ3:A

AC2 Q07817

PFAM2 PF00452

PDB2 4AQ3:A

INT non-edgetic

AA1 11N;Interacts with:188W,171A,175N,174L,179E

AA1 12R;Interacts with:171A,175N

MUT1 R12Q;PMID:9463381;

AA1 14I;Interacts with:90L,188W,144F

AA1 15V;Interacts with:170M,174L,167A,144F,171A

AA1 16M;Interacts with:167A

AA1 17K;Interacts with:91R

AA1 18Y;Interacts with:145S,144F,90L,91R,148G,170M,95D

AA1 19I;Interacts with:163V,167A,170M

AA1 21Y;Interacts with:91R

AA1 22K;Interacts with:95D,152V,145S,98E,91R

AA1 23L;Interacts with:155V,163V,152V,151C

AA1 25Q;Interacts with:95D,183Q

AA1 26R;Interacts with:179E,184E,180P,183Q

AA1 27G;Interacts with:183Q

AA1 28Y;Interacts with:155V

AA1 30W;Interacts with:167A,163V,164S,160Q

AA1 92V;Interacts with:90L

AA1 9Y;Interacts with:188W

AA2 144F;Interacts with:18Y,15V,14I

AA2 145S;Interacts with:18Y,22K

AA2 148G;Interacts with:18Y

MUT2 G148E;827593;

AA2 151C;Interacts with:23L

AA2 152V;Interacts with:23L,22K

AA2 155V;Interacts with:23L,28Y

AA2 160Q;Interacts with:30W

AA2 163V;Interacts with:19I,23L,30W

AA2 164S;Interacts with:30W

AA2 167A;Interacts with:15V,19I,30W,16M

MUT2 A167V;1KG:1359392;

AA2 170M;Interacts with:15V,19I,18Y

AA2 171A;Interacts with:11N,15V,12R

AA2 174L;Interacts with:15V,11N

AA2 175N;Interacts with:11N,12R

AA2 179E;Interacts with:26R,11N

AA2 180P;Interacts with:26R

AA2 183Q;Interacts with:25Q,27G,26R

AA2 184E;Interacts with:26R

AA2 188W;Interacts with:11N,14I,9Y

AA2 90L;Interacts with:14I,18Y,92V

AA2 91R;Interacts with:17K,21Y,18Y,22K

AA2 95D;Interacts with:22K,25Q,18Y

AA2 98E;Interacts with:22K

//

MESH1 mesh:D000544

NAME1 Alzheimer Disease

CLUSTER1 clust2

MESH2 mesh:D002277

NAME2 Carcinoma

CLUSTER2 clust2

AC1 O43521

PFAM1 PF08945

PDB1 3FDL:B

AC2 Q07817

PFAM2 PF00452

PDB2 3FDL:B

INT non-edgetic

AA1 143R;Interacts with:111Q

MUT1 R143H;dbSNP:rs141962978;dbSNP:rs141962978;dbSNP:rs141962978;dbSNP:rs141962978;dbSNP:rs141962978;

AA1 144P;Interacts with:112L,111Q

AA1 145E;Interacts with:126V,113H,125Q,122S,112L

AA1 146I;Interacts with:125Q

AA1 147W;Interacts with:107D,108L

AA1 148I;Interacts with:126V,108L,146F,111Q,112L,104A

AA1 149A;Interacts with:126V,129E,130L

AA1 150Q;Interacts with:129E

AA1 151E;Interacts with:108L

AA1 152L;Interacts with:142A,130L,97F,146F,105F,108L,126V,145S,104A

AA1 153R;Interacts with:130L,133D,139R,129E

MUT1 R153W;dbSNP:rs146318804;dbSNP:rs146318804;dbSNP:rs146318804;dbSNP:rs146318804;dbSNP:rs146318804;

AA1 155I;Interacts with:97F,101Y,105F,108L,104A

AA1 156G;Interacts with:136N,97F,142A,139R,138G,101Y

AA1 157D;Interacts with:138G,139R,136N

AA1 158E;Interacts with:100R,101Y

AA1 159F;Interacts with:97F,96E,93A,141V,138G,101Y,100R

AA1 160N;Interacts with:137W,136N,138G,139R

AA1 162Y;Interacts with:100R

AA1 163Y;Interacts with:100R

AA1 1M;Interacts with:113H,111Q,112L

AA2 100R;Interacts with:158E,159F,162Y,163Y

AA2 101Y;Interacts with:155I,159F,158E,156G

AA2 104A;Interacts with:155I,152L,148I

AA2 105F;Interacts with:155I,152L

AA2 107D;Interacts with:147W

AA2 108L;Interacts with:155I,147W,148I,152L,151E

MUT2 L108R;dbSNP:rs139457299;dbSNP:rs139457299;

AA2 111Q;Interacts with:143R,148I,1M,144P

AA2 112L;Interacts with:148I,1M,145E,144P

AA2 113H;Interacts with:1M,145E

AA2 122S;Interacts with:145E

AA2 125Q;Interacts with:145E,146I

AA2 126V;Interacts with:145E,149A,148I,152L

AA2 129E;Interacts with:149A,150Q,153R

AA2 130L;Interacts with:153R,152L,149A

AA2 133D;Interacts with:153R

AA2 136N;Interacts with:156G,160N,157D

AA2 137W;Interacts with:160N

AA2 138G;Interacts with:157D,160N,159F,156G

MUT2 G138A;827592;

AA2 139R;Interacts with:157D,156G,153R,160N

AA2 141V;Interacts with:159F

AA2 142A;Interacts with:156G,152L

AA2 145S;Interacts with:152L

AA2 146F;Interacts with:152L,148I

AA2 93A;Interacts with:159F

AA2 96E;Interacts with:159F

AA2 97F;Interacts with:155I,159F,156G,152L

//

MESH1 mesh:D000544

NAME1 Alzheimer Disease

CLUSTER1 clust2

MESH2 mesh:D002277

NAME2 Carcinoma

CLUSTER2 clust2

AC1 Q13794

PFAM1 PF15150

PDB1 3MQP:B

AC2 Q16548

PFAM2 PF00452

PDB2 3MQP:B

INT non-edgetic

AA1 21L;Interacts with:55C,59V,58N

AA1 22E;Interacts with:74V,73Q

MUT1 E22K;dbSNP:rs1126436;

AA1 24E;Interacts with:52L

AA1 25C;Interacts with:74V,52L

AA1 26A;Interacts with:77K,78E,74V

AA1 28Q;Interacts with:51N

AA1 29L;Interacts with:91T,48V,74V,78E,95F

MUT1 L29A;823237;

MUT1 L29E;823238;

AA1 30R;Interacts with:78E,81D,77K,80E,88R,91T

MUT1 R30delR;COSMIC:166155;

AA1 32F;Interacts with:47E,91T,48V

MUT1 F32I;823240;

MUT1 F32E;823239;

AA1 33G;Interacts with:91T,88R,85N,87G,78E

AA1 34D;Interacts with:87G,85N,88R

AA1 36L;Interacts with:87G,44V,40V,90V

MUT1 L36E;823242;

AA1 37N;Interacts with:86W,87G,88R,85N

AA2 40V;Interacts with:36L

AA2 44V;Interacts with:36L

AA2 47E;Interacts with:32F

AA2 48V;Interacts with:32F,29L

AA2 51N;Interacts with:28Q

AA2 52L;Interacts with:25C,24E

AA2 55C;Interacts with:21L

AA2 58N;Interacts with:21L

MUT2 N58S;dbSNP:rs141166047;dbSNP:rs141166047;

AA2 59V;Interacts with:21L

AA2 73Q;Interacts with:22E

AA2 74V;Interacts with:25C,29L,26A,22E

AA2 77K;Interacts with:26A,30R

AA2 78E;Interacts with:26A,30R,29L,33G

AA2 80E;Interacts with:30R

AA2 81D;Interacts with:30R

AA2 85N;Interacts with:34D,33G,37N

AA2 86W;Interacts with:37N

AA2 87G;Interacts with:36L,34D,37N,33G

AA2 88R;Interacts with:33G,37N,30R,34D

AA2 90V;Interacts with:36L

AA2 91T;Interacts with:29L,32F,33G,30R

AA2 95F;Interacts with:29L

//

MESH1 mesh:D000544

NAME1 Alzheimer Disease

CLUSTER1 clust2

MESH2 mesh:D002277

NAME2 Carcinoma

CLUSTER2 clust2

AC1 O43521

PFAM1 PF08945

PDB1 2VM6:B

AC2 Q16548

PFAM2 PF00452

PDB2 2VM6:B

INT non-edgetic

AA1 142M;Interacts with:55C,58N

MUT1 M142R;dbSNP:rs114585494;dbSNP:rs114585494;dbSNP:rs114585494;dbSNP:rs114585494;dbSNP:rs114585494;

AA1 144P;Interacts with:70L,74V

AA1 145E;Interacts with:70L,77K,73Q,74V

AA1 147W;Interacts with:54S,55C,52L,51N

AA1 148I;Interacts with:70L,74V,52L,95F,56L,59V

AA1 149A;Interacts with:77K,78E,74V

AA1 151E;Interacts with:51N,48V

AA1 152L;Interacts with:78E,48V,95F,91T,74V

AA1 153R;Interacts with:79F,88R,80E,78E,81D,91T,77K

MUT1 R153W;dbSNP:rs146318804;dbSNP:rs146318804;dbSNP:rs146318804;dbSNP:rs146318804;dbSNP:rs146318804;

AA1 154R;Interacts with:47E,51N

AA1 155I;Interacts with:48V,91T,44V,47E

AA1 156G;Interacts with:87G,91T,85N,88R

AA1 157D;Interacts with:88R,87G,85N

AA1 159F;Interacts with:40V,43S,44V,87G

AA1 160N;Interacts with:86W,87G,85N

AA1 163Y;Interacts with:40V

AA2 40V;Interacts with:163Y,159F

AA2 43S;Interacts with:159F

AA2 44V;Interacts with:155I,159F

AA2 47E;Interacts with:154R,155I

AA2 48V;Interacts with:155I,152L,151E

AA2 51N;Interacts with:147W,154R,151E

AA2 52L;Interacts with:148I,147W

AA2 54S;Interacts with:147W

AA2 55C;Interacts with:147W,142M

AA2 56L;Interacts with:148I

AA2 58N;Interacts with:142M

MUT2 N58S;dbSNP:rs141166047;dbSNP:rs141166047;

AA2 59V;Interacts with:148I

AA2 70L;Interacts with:145E,148I,144P

AA2 73Q;Interacts with:145E

AA2 74V;Interacts with:148I,144P,152L,149A,145E

AA2 77K;Interacts with:149A,145E,153R

AA2 78E;Interacts with:152L,153R,149A

AA2 79F;Interacts with:153R

AA2 80E;Interacts with:153R

AA2 81D;Interacts with:153R

AA2 85N;Interacts with:157D,156G,160N

AA2 86W;Interacts with:160N

AA2 87G;Interacts with:157D,156G,160N,159F

AA2 88R;Interacts with:157D,153R,156G

AA2 91T;Interacts with:152L,155I,156G,153R

AA2 95F;Interacts with:152L,148I

//

MESH1 mesh:D000544

NAME1 Alzheimer Disease

CLUSTER1 clust2

MESH2 mesh:D002277

NAME2 Carcinoma

CLUSTER2 clust2

AC1 Q07817

PFAM1 PF00452

PDB1 2P1L:A

AC2 Q14457

PFAM2 PF15285

PDB2 2P1L:A

INT semi-edgetic

AA1 100R;Interacts with:122L,119T,123F

AA1 101Y;Interacts with:116L,115R,119T,112L

AA1 105F;Interacts with:115R,118V

AA1 106S;Interacts with:115R

AA1 108L;Interacts with:116L,112L

MUT1 L108R;dbSNP:rs139457299;dbSNP:rs139457299;

AA1 111Q;Interacts with:115R,108T,111N,112L

AA1 112L;Interacts with:108T,109M,112L

AA1 113H;Interacts with:3G,108T,109M

AA1 122S;Interacts with:109M

AA1 125Q;Interacts with:4S,106G

AA1 126V;Interacts with:109M,112L,113S,116L

AA1 129E;Interacts with:113S,117K,114R

AA1 130L;Interacts with:117K,116L,113S

AA1 133D;Interacts with:117K

AA1 136N;Interacts with:120G,124D,121D,125I

AA1 137W;Interacts with:124D,125I,126M

AA1 138G;Interacts with:124D,123F,121D,120G

MUT1 G138A;827592;

AA1 139R;Interacts with:121D,120G,117K,124D

AA1 141V;Interacts with:123F

AA1 142A;Interacts with:116L,120G

AA1 146F;Interacts with:112L,116L

AA1 185N;Interacts with:126M

AA1 93A;Interacts with:123F

AA1 96E;Interacts with:123F

AA1 97F;Interacts with:119T,123F,120G,116L

AA2 106G;Interacts with:125Q

AA2 108T;Interacts with:112L,111Q,113H

AA2 109M;Interacts with:126V,122S,112L,113H

AA2 111N;Interacts with:111Q

AA2 112L;Interacts with:146F,126V,108L,112L,101Y,111Q

AA2 113S;Interacts with:129E,126V,130L

MUT2 S113R;dbSNP:rs80236238;

AA2 114R;Interacts with:129E

AA2 115R;Interacts with:111Q,105F,101Y,106S

AA2 116L;Interacts with:101Y,130L,142A,146F,108L,126V,97F

AA2 117K;Interacts with:130L,139R,133D,129E

AA2 118V;Interacts with:105F

AA2 119T;Interacts with:97F,100R,101Y

AA2 120G;Interacts with:139R,136N,97F,142A,138G

AA2 121D;Interacts with:139R,138G,136N

AA2 122L;Interacts with:100R

AA2 123F;Interacts with:97F,93A,141V,138G,96E,100R

AA2 124D;Interacts with:138G,136N,137W,139R

AA2 125I;Interacts with:137W,136N

AA2 126M;Interacts with:185N,137W

AA2 3G;Interacts with:113H

AA2 4S;Interacts with:125Q

//

MESH1 mesh:D000544

NAME1 Alzheimer Disease

CLUSTER1 clust2

MESH2 mesh:D002277

NAME2 Carcinoma

CLUSTER2 clust2

AC1 Q07812

PFAM1 PF00452

PDB1 4BD2:A

AC2 P55957

PFAM2 PF06393

PDB2 4BD2:A

INT semi-edgetic

AA1 106N;Interacts with:98D,94G,95D

AA1 108G;Interacts with:94G,97M

MUT1 G108V;828239;humsavar:VAR_013576, phenotype=A Burkitt lymphoma;

AA1 109R;Interacts with:91A,95D,94G

AA1 111V;Interacts with:97M

AA1 112A;Interacts with:93V,94G,90L

AA1 115Y;Interacts with:90L

AA1 116F;Interacts with:90L,86I

AA1 65R;Interacts with:103P,102P

MUT1 R65L;dbSNP:rs142278713;dbSNP:rs142278713;dbSNP:rs142278713;

AA1 66I;Interacts with:104G,97M,105L,103P

AA1 69E;Interacts with:105L,104G

AA1 70L;Interacts with:105L,93V

AA1 73N;Interacts with:89H

AA1 75E;Interacts with:89H

AA1 76L;Interacts with:89H,90L

AA1 79M;Interacts with:89H,86I,82I

AA1 80I;Interacts with:86I

AA1 82A;Interacts with:79Q,82I

AA1 83V;Interacts with:82I,86I,79Q,83I

AA1 84D;Interacts with:79Q

AA1 91V;Interacts with:83I

AA1 94R;Interacts with:83I,80E

AA1 95V;Interacts with:83I,90L,87A,86I

AA1 98D;Interacts with:87A,84R

AA1 99M;Interacts with:91A,90L,87A

AA2 102P;Interacts with:65R

AA2 103P;Interacts with:65R,66I

AA2 104G;Interacts with:66I,69E

AA2 105L;Interacts with:70L,69E,66I

MUT2 L105P;dbSNP:rs143734092;dbSNP:rs143734092;

AA2 79Q;Interacts with:82A,83V,84D

AA2 80E;Interacts with:94R

AA2 82I;Interacts with:83V,82A,79M

AA2 83I;Interacts with:91V,94R,83V,95V

AA2 84R;Interacts with:98D

MUT2 R84W;dbSNP:rs148107209;dbSNP:rs148107209;

AA2 86I;Interacts with:83V,116F,79M,80I,95V

AA2 87A;Interacts with:98D,99M,95V

AA2 89H;Interacts with:76L,75E,79M,73N

AA2 90L;Interacts with:116F,76L,95V,112A,99M,115Y

AA2 91A;Interacts with:109R,99M

AA2 93V;Interacts with:70L,112A

AA2 94G;Interacts with:108G,106N,112A,109R

MUT2 G94E;PMID:11121101;

AA2 95D;Interacts with:109R,106N

AA2 97M;Interacts with:66I,111V,108G

AA2 98D;Interacts with:106N

//

MESH1 mesh:D000544

NAME1 Alzheimer Disease

CLUSTER1 clust2

MESH2 mesh:D002277

NAME2 Carcinoma

CLUSTER2 clust2

AC1 Q07820

PFAM1 PF00452

PDB1 2KBW:A

AC2 P55957

PFAM2 PF06393

PDB2 2KBW:A

INT semi-edgetic

AA1 216V;Interacts with:97M

AA1 220V;Interacts with:97M

AA1 224H;Interacts with:93V

AA1 227A;Interacts with:93V

MUT1 A227V;169059;dbSNP:rs11580946;humsavar:VAR_024022;rs11580946;

AA1 231M;Interacts with:93V,90L,89H,86I

MUT1 M231L;898693;dbSNP:rs140449444;humsavar:VAR_054157;

AA1 234K;Interacts with:82I,86I

MUT1 K234R;898704;

AA1 235L;Interacts with:86I,82I,83I,79Q

AA1 236D;Interacts with:79Q

AA1 242D;Interacts with:79Q

AA1 245S;Interacts with:83I,79Q

AA1 248R;Interacts with:83I

AA1 249V;Interacts with:86I,83I,90L,87A

AA1 252H;Interacts with:84R,87A

AA1 253V;Interacts with:88R,90L,91A,87A

AA1 260N;Interacts with:95D,98D,94G

AA1 261W;Interacts with:98D

AA1 262G;Interacts with:95D,97M,94G,98D

AA1 263R;Interacts with:95D,98D,91A,94G

AA1 265V;Interacts with:97M

AA1 266T;Interacts with:93V,97M,90L,95D,94G

AA1 267L;Interacts with:90L

AA1 270F;Interacts with:90L

AA2 79Q;Interacts with:242D,236D,245S,235L

AA2 82I;Interacts with:234K,235L

AA2 83I;Interacts with:245S,249V,235L,248R

AA2 84R;Interacts with:252H

MUT2 R84W;dbSNP:rs148107209;dbSNP:rs148107209;

AA2 86I;Interacts with:235L,249V,234K,231M

AA2 87A;Interacts with:252H,253V,249V

AA2 88R;Interacts with:253V

AA2 89H;Interacts with:231M

AA2 90L;Interacts with:253V,231M,249V,266T,270F,267L

AA2 91A;Interacts with:253V,263R

AA2 93V;Interacts with:266T,231M,227A,224H

AA2 94G;Interacts with:263R,262G,266T,260N

MUT2 G94E;PMID:11121101;

AA2 95D;Interacts with:263R,266T,262G,260N

AA2 97M;Interacts with:266T,265V,216V,262G,220V

AA2 98D;Interacts with:261W,263R,262G,260N

//

MESH1 mesh:D000544

NAME1 Alzheimer Disease

CLUSTER1 clust2

MESH2 mesh:D002277

NAME2 Carcinoma

CLUSTER2 clust2

AC1 Q92843

PFAM1 PF00452

PDB1 1ZY3:A

AC2 P55957

PFAM2 PF06393

PDB2 1ZY3:A

INT semi-edgetic

AA1 102F;Interacts with:90L

AA1 52E;Interacts with:99R

AA1 53F;Interacts with:98D

AA1 56R;Interacts with:98D,100S,97M

AA1 57F;Interacts with:97M,93V,94G

AA1 64L;Interacts with:90L,93V

AA1 67Q;Interacts with:90L,89H

AA1 68L;Interacts with:89H,90L,86I

AA1 71T;Interacts with:83I,86I

AA1 82V;Interacts with:86I,83I,90L,87A

AA1 83S;Interacts with:90L

AA1 85E;Interacts with:82I,87A,83I,84R

AA1 86L;Interacts with:84R,88R,91A,87A

AA1 88Q;Interacts with:84R

AA1 92N;Interacts with:95D

AA1 93W;Interacts with:95D

AA1 94G;Interacts with:95D

AA1 95R;Interacts with:91A,87A,95D,90L

AA1 98A;Interacts with:90L

AA2 100S;Interacts with:56R

AA2 82I;Interacts with:85E

AA2 83I;Interacts with:71T,82V,85E

AA2 84R;Interacts with:88Q,86L,85E

MUT2 R84W;dbSNP:rs148107209;dbSNP:rs148107209;

AA2 86I;Interacts with:82V,71T,68L

AA2 87A;Interacts with:95R,85E,82V,86L

AA2 88R;Interacts with:86L

AA2 89H;Interacts with:68L,67Q

AA2 90L;Interacts with:67Q,64L,98A,102F,83S,68L,82V,95R

AA2 91A;Interacts with:95R,86L

AA2 93V;Interacts with:57F,64L

AA2 94G;Interacts with:57F

MUT2 G94E;PMID:11121101;

AA2 95D;Interacts with:92N,93W,95R,94G

AA2 97M;Interacts with:57F,56R

AA2 98D;Interacts with:53F,56R

AA2 99R;Interacts with:52E

//

MESH1 mesh:D000544

NAME1 Alzheimer Disease

CLUSTER1 clust2

MESH2 mesh:D006528

NAME2 Carcinoma, Hepatocellular

CLUSTER2 clust2

AC1 Q13794

PFAM1 PF15150

PDB1 3MQP:B

AC2 Q16548

PFAM2 PF00452

PDB2 3MQP:B

INT non-edgetic

AA1 21L;Interacts with:55C,59V,58N

AA1 22E;Interacts with:74V,73Q

MUT1 E22K;dbSNP:rs1126436;

AA1 24E;Interacts with:52L

AA1 25C;Interacts with:74V,52L

AA1 26A;Interacts with:77K,78E,74V

AA1 28Q;Interacts with:51N

AA1 29L;Interacts with:91T,48V,74V,78E,95F

MUT1 L29A;823237;

MUT1 L29E;823238;

AA1 30R;Interacts with:78E,81D,77K,80E,88R,91T

MUT1 R30delR;COSMIC:166155;

AA1 32F;Interacts with:47E,91T,48V

MUT1 F32I;823240;

MUT1 F32E;823239;

AA1 33G;Interacts with:91T,88R,85N,87G,78E

AA1 34D;Interacts with:87G,85N,88R

AA1 36L;Interacts with:87G,44V,40V,90V

MUT1 L36E;823242;

AA1 37N;Interacts with:86W,87G,88R,85N

AA2 40V;Interacts with:36L

AA2 44V;Interacts with:36L

AA2 47E;Interacts with:32F

AA2 48V;Interacts with:32F,29L

AA2 51N;Interacts with:28Q

AA2 52L;Interacts with:25C,24E

AA2 55C;Interacts with:21L

AA2 58N;Interacts with:21L

MUT2 N58S;dbSNP:rs141166047;dbSNP:rs141166047;

AA2 59V;Interacts with:21L

AA2 73Q;Interacts with:22E

AA2 74V;Interacts with:25C,29L,26A,22E

AA2 77K;Interacts with:26A,30R

AA2 78E;Interacts with:26A,30R,29L,33G

AA2 80E;Interacts with:30R

AA2 81D;Interacts with:30R

AA2 85N;Interacts with:34D,33G,37N

AA2 86W;Interacts with:37N

AA2 87G;Interacts with:36L,34D,37N,33G

AA2 88R;Interacts with:33G,37N,30R,34D

AA2 90V;Interacts with:36L

AA2 91T;Interacts with:29L,32F,33G,30R

AA2 95F;Interacts with:29L

//

MESH1 mesh:D000544

NAME1 Alzheimer Disease

CLUSTER1 clust2

MESH2 mesh:D006528

NAME2 Carcinoma, Hepatocellular

CLUSTER2 clust2

AC1 P10415

PFAM1 PF00452

PDB1 4AQ3:A

AC2 Q07817

PFAM2 PF00452

PDB2 4AQ3:C

INT semi-edgetic

AA1 102D;Interacts with:100R

AA1 103D;Interacts with:100R,99L,97F

AA1 104F;Interacts with:96E

AA1 106R;Interacts with:96E

AA1 107R;Interacts with:95D,96E,92E,91R

AA1 108Y;Interacts with:92E

AA1 115M;Interacts with:117G

AA1 118Q;Interacts with:116P,115T,117G

AA1 119L;Interacts with:117G

AA1 120H;Interacts with:115T

AA1 129R;Interacts with:117G

MUT1 R129C;828636;

AA1 132T;Interacts with:117G

AA1 133V;Interacts with:117G

AA1 135E;Interacts with:120Y

AA1 136E;Interacts with:120Y

MUT1 E136R;PMID:11461956;

AA1 98R;Interacts with:100R

AA1 99Q;Interacts with:100R,101Y

AA2 100R;Interacts with:103D,102D,99Q,98R

AA2 101Y;Interacts with:99Q

AA2 115T;Interacts with:118Q,120H

AA2 116P;Interacts with:118Q

AA2 117G;Interacts with:132T,129R,115M,133V,119L,118Q

AA2 120Y;Interacts with:135E,136E

AA2 91R;Interacts with:107R

AA2 92E;Interacts with:108Y,107R

AA2 95D;Interacts with:107R

AA2 96E;Interacts with:107R,104F,106R

AA2 97F;Interacts with:103D

AA2 99L;Interacts with:103D

//

MESH1 mesh:D000544

NAME1 Alzheimer Disease

CLUSTER1 clust2

MESH2 mesh:D006528

NAME2 Carcinoma, Hepatocellular

CLUSTER2 clust2

AC1 P10415

PFAM1 PF02180

PDB1 4AQ3:A

AC2 Q07817

PFAM2 PF00452

PDB2 4AQ3:A

INT semi-edgetic

AA1 11N;Interacts with:188W,171A,175N,174L,179E

AA1 12R;Interacts with:171A,175N

MUT1 R12Q;PMID:9463381;

AA1 14I;Interacts with:90L,188W,144F

AA1 15V;Interacts with:170M,174L,167A,144F,171A

AA1 16M;Interacts with:167A

AA1 17K;Interacts with:91R

AA1 18Y;Interacts with:145S,144F,90L,91R,148G,170M,95D

AA1 19I;Interacts with:163V,167A,170M

AA1 21Y;Interacts with:91R

AA1 22K;Interacts with:95D,152V,145S,98E,91R

AA1 23L;Interacts with:155V,163V,152V,151C

AA1 25Q;Interacts with:95D,183Q

AA1 26R;Interacts with:179E,184E,180P,183Q

AA1 27G;Interacts with:183Q

AA1 28Y;Interacts with:155V

AA1 30W;Interacts with:167A,163V,164S,160Q

AA1 92V;Interacts with:90L

AA1 9Y;Interacts with:188W

AA2 144F;Interacts with:18Y,15V,14I

AA2 145S;Interacts with:18Y,22K

AA2 148G;Interacts with:18Y

MUT2 G148E;827593;

AA2 151C;Interacts with:23L

AA2 152V;Interacts with:23L,22K

AA2 155V;Interacts with:23L,28Y

AA2 160Q;Interacts with:30W

AA2 163V;Interacts with:19I,23L,30W

AA2 164S;Interacts with:30W

AA2 167A;Interacts with:15V,19I,30W,16M

MUT2 A167V;1KG:1359392;

AA2 170M;Interacts with:15V,19I,18Y

AA2 171A;Interacts with:11N,15V,12R

AA2 174L;Interacts with:15V,11N

AA2 175N;Interacts with:11N,12R

AA2 179E;Interacts with:26R,11N

AA2 180P;Interacts with:26R

AA2 183Q;Interacts with:25Q,27G,26R

AA2 184E;Interacts with:26R

AA2 188W;Interacts with:11N,14I,9Y

AA2 90L;Interacts with:14I,18Y,92V

AA2 91R;Interacts with:17K,21Y,18Y,22K

AA2 95D;Interacts with:22K,25Q,18Y

AA2 98E;Interacts with:22K

//

MESH1 mesh:D000544

NAME1 Alzheimer Disease

CLUSTER1 clust2

MESH2 mesh:D006528

NAME2 Carcinoma, Hepatocellular

CLUSTER2 clust2

AC1 O43521

PFAM1 PF08945

PDB1 3FDL:B

AC2 Q07817

PFAM2 PF00452

PDB2 3FDL:B

INT semi-edgetic

AA1 143R;Interacts with:111Q

MUT1 R143H;dbSNP:rs141962978;dbSNP:rs141962978;dbSNP:rs141962978;dbSNP:rs141962978;dbSNP:rs141962978;

AA1 144P;Interacts with:112L,111Q

AA1 145E;Interacts with:126V,113H,125Q,122S,112L

AA1 146I;Interacts with:125Q

AA1 147W;Interacts with:107D,108L

AA1 148I;Interacts with:126V,108L,146F,111Q,112L,104A

AA1 149A;Interacts with:126V,129E,130L

AA1 150Q;Interacts with:129E

AA1 151E;Interacts with:108L

AA1 152L;Interacts with:142A,130L,97F,146F,105F,108L,126V,145S,104A

AA1 153R;Interacts with:130L,133D,139R,129E

MUT1 R153W;dbSNP:rs146318804;dbSNP:rs146318804;dbSNP:rs146318804;dbSNP:rs146318804;dbSNP:rs146318804;

AA1 155I;Interacts with:97F,101Y,105F,108L,104A

AA1 156G;Interacts with:136N,97F,142A,139R,138G,101Y

AA1 157D;Interacts with:138G,139R,136N

AA1 158E;Interacts with:100R,101Y

AA1 159F;Interacts with:97F,96E,93A,141V,138G,101Y,100R

AA1 160N;Interacts with:137W,136N,138G,139R

AA1 162Y;Interacts with:100R

AA1 163Y;Interacts with:100R

AA1 1M;Interacts with:113H,111Q,112L

AA2 100R;Interacts with:158E,159F,162Y,163Y

AA2 101Y;Interacts with:155I,159F,158E,156G

AA2 104A;Interacts with:155I,152L,148I

AA2 105F;Interacts with:155I,152L

AA2 107D;Interacts with:147W

AA2 108L;Interacts with:155I,147W,148I,152L,151E

MUT2 L108R;dbSNP:rs139457299;dbSNP:rs139457299;

AA2 111Q;Interacts with:143R,148I,1M,144P

AA2 112L;Interacts with:148I,1M,145E,144P

AA2 113H;Interacts with:1M,145E

AA2 122S;Interacts with:145E

AA2 125Q;Interacts with:145E,146I

AA2 126V;Interacts with:145E,149A,148I,152L

AA2 129E;Interacts with:149A,150Q,153R

AA2 130L;Interacts with:153R,152L,149A

AA2 133D;Interacts with:153R

AA2 136N;Interacts with:156G,160N,157D

AA2 137W;Interacts with:160N

AA2 138G;Interacts with:157D,160N,159F,156G

MUT2 G138A;827592;

AA2 139R;Interacts with:157D,156G,153R,160N

AA2 141V;Interacts with:159F

AA2 142A;Interacts with:156G,152L

AA2 145S;Interacts with:152L

AA2 146F;Interacts with:152L,148I

AA2 93A;Interacts with:159F

AA2 96E;Interacts with:159F

AA2 97F;Interacts with:155I,159F,156G,152L

//

MESH1 mesh:D000544

NAME1 Alzheimer Disease

CLUSTER1 clust2

MESH2 mesh:D006528

NAME2 Carcinoma, Hepatocellular

CLUSTER2 clust2

AC1 Q92843

PFAM1 PF00452

PDB1 1ZY3:A

AC2 P55957

PFAM2 PF06393

PDB2 1ZY3:A

INT semi-edgetic

AA1 102F;Interacts with:90L

AA1 52E;Interacts with:99R

AA1 53F;Interacts with:98D

AA1 56R;Interacts with:98D,100S,97M

AA1 57F;Interacts with:97M,93V,94G

AA1 64L;Interacts with:90L,93V

AA1 67Q;Interacts with:90L,89H

AA1 68L;Interacts with:89H,90L,86I

AA1 71T;Interacts with:83I,86I

AA1 82V;Interacts with:86I,83I,90L,87A

AA1 83S;Interacts with:90L

AA1 85E;Interacts with:82I,87A,83I,84R

AA1 86L;Interacts with:84R,88R,91A,87A

AA1 88Q;Interacts with:84R

AA1 92N;Interacts with:95D

AA1 93W;Interacts with:95D

AA1 94G;Interacts with:95D

AA1 95R;Interacts with:91A,87A,95D,90L

AA1 98A;Interacts with:90L

AA2 100S;Interacts with:56R

AA2 82I;Interacts with:85E

AA2 83I;Interacts with:71T,82V,85E

AA2 84R;Interacts with:88Q,86L,85E

MUT2 R84W;dbSNP:rs148107209;dbSNP:rs148107209;

AA2 86I;Interacts with:82V,71T,68L

AA2 87A;Interacts with:95R,85E,82V,86L

AA2 88R;Interacts with:86L

AA2 89H;Interacts with:68L,67Q

AA2 90L;Interacts with:67Q,64L,98A,102F,83S,68L,82V,95R

AA2 91A;Interacts with:95R,86L

AA2 93V;Interacts with:57F,64L

AA2 94G;Interacts with:57F

MUT2 G94E;PMID:11121101;

AA2 95D;Interacts with:92N,93W,95R,94G

AA2 97M;Interacts with:57F,56R

AA2 98D;Interacts with:53F,56R

AA2 99R;Interacts with:52E

//

MESH1 mesh:D000544

NAME1 Alzheimer Disease

CLUSTER1 clust2

MESH2 mesh:D006528

NAME2 Carcinoma, Hepatocellular

CLUSTER2 clust2

AC1 O43521

PFAM1 PF08945

PDB1 2VM6:B

AC2 Q16548

PFAM2 PF00452

PDB2 2VM6:B

INT semi-edgetic

AA1 142M;Interacts with:55C,58N

MUT1 M142R;dbSNP:rs114585494;dbSNP:rs114585494;dbSNP:rs114585494;dbSNP:rs114585494;dbSNP:rs114585494;

AA1 144P;Interacts with:70L,74V

AA1 145E;Interacts with:70L,77K,73Q,74V

AA1 147W;Interacts with:54S,55C,52L,51N

AA1 148I;Interacts with:70L,74V,52L,95F,56L,59V

AA1 149A;Interacts with:77K,78E,74V

AA1 151E;Interacts with:51N,48V

AA1 152L;Interacts with:78E,48V,95F,91T,74V

AA1 153R;Interacts with:79F,88R,80E,78E,81D,91T,77K

MUT1 R153W;dbSNP:rs146318804;dbSNP:rs146318804;dbSNP:rs146318804;dbSNP:rs146318804;dbSNP:rs146318804;

AA1 154R;Interacts with:47E,51N

AA1 155I;Interacts with:48V,91T,44V,47E

AA1 156G;Interacts with:87G,91T,85N,88R

AA1 157D;Interacts with:88R,87G,85N

AA1 159F;Interacts with:40V,43S,44V,87G

AA1 160N;Interacts with:86W,87G,85N

AA1 163Y;Interacts with:40V

AA2 40V;Interacts with:163Y,159F

AA2 43S;Interacts with:159F

AA2 44V;Interacts with:155I,159F

AA2 47E;Interacts with:154R,155I

AA2 48V;Interacts with:155I,152L,151E

AA2 51N;Interacts with:147W,154R,151E

AA2 52L;Interacts with:148I,147W

AA2 54S;Interacts with:147W

AA2 55C;Interacts with:147W,142M

AA2 56L;Interacts with:148I

AA2 58N;Interacts with:142M

MUT2 N58S;dbSNP:rs141166047;dbSNP:rs141166047;

AA2 59V;Interacts with:148I

AA2 70L;Interacts with:145E,148I,144P

AA2 73Q;Interacts with:145E

AA2 74V;Interacts with:148I,144P,152L,149A,145E

AA2 77K;Interacts with:149A,145E,153R

AA2 78E;Interacts with:152L,153R,149A

AA2 79F;Interacts with:153R

AA2 80E;Interacts with:153R

AA2 81D;Interacts with:153R

AA2 85N;Interacts with:157D,156G,160N

AA2 86W;Interacts with:160N

AA2 87G;Interacts with:157D,156G,160N,159F

AA2 88R;Interacts with:157D,153R,156G

AA2 91T;Interacts with:152L,155I,156G,153R

AA2 95F;Interacts with:152L,148I

//

MESH1 mesh:D000544

NAME1 Alzheimer Disease

CLUSTER1 clust2

MESH2 mesh:D006528

NAME2 Carcinoma, Hepatocellular

CLUSTER2 clust2

AC1 Q07812

PFAM1 PF00452

PDB1 4BD2:A

AC2 P55957

PFAM2 PF06393

PDB2 4BD2:A

INT edgetic

AA1 106N;Interacts with:98D,94G,95D

AA1 108G;Interacts with:94G,97M

MUT1 G108V;828239;humsavar:VAR_013576, phenotype=A Burkitt lymphoma;

AA1 109R;Interacts with:91A,95D,94G

AA1 111V;Interacts with:97M

AA1 112A;Interacts with:93V,94G,90L

AA1 115Y;Interacts with:90L

AA1 116F;Interacts with:90L,86I

AA1 65R;Interacts with:103P,102P

MUT1 R65L;dbSNP:rs142278713;dbSNP:rs142278713;dbSNP:rs142278713;

AA1 66I;Interacts with:104G,97M,105L,103P

AA1 69E;Interacts with:105L,104G

AA1 70L;Interacts with:105L,93V

AA1 73N;Interacts with:89H

AA1 75E;Interacts with:89H

AA1 76L;Interacts with:89H,90L

AA1 79M;Interacts with:89H,86I,82I

AA1 80I;Interacts with:86I

AA1 82A;Interacts with:79Q,82I

AA1 83V;Interacts with:82I,86I,79Q,83I

AA1 84D;Interacts with:79Q

AA1 91V;Interacts with:83I

AA1 94R;Interacts with:83I,80E

AA1 95V;Interacts with:83I,90L,87A,86I

AA1 98D;Interacts with:87A,84R

AA1 99M;Interacts with:91A,90L,87A

AA2 102P;Interacts with:65R

AA2 103P;Interacts with:65R,66I

AA2 104G;Interacts with:66I,69E

AA2 105L;Interacts with:70L,69E,66I

MUT2 L105P;dbSNP:rs143734092;dbSNP:rs143734092;

AA2 79Q;Interacts with:82A,83V,84D

AA2 80E;Interacts with:94R

AA2 82I;Interacts with:83V,82A,79M

AA2 83I;Interacts with:91V,94R,83V,95V

AA2 84R;Interacts with:98D

MUT2 R84W;dbSNP:rs148107209;dbSNP:rs148107209;

AA2 86I;Interacts with:83V,116F,79M,80I,95V

AA2 87A;Interacts with:98D,99M,95V

AA2 89H;Interacts with:76L,75E,79M,73N

AA2 90L;Interacts with:116F,76L,95V,112A,99M,115Y

AA2 91A;Interacts with:109R,99M

AA2 93V;Interacts with:70L,112A

AA2 94G;Interacts with:108G,106N,112A,109R

MUT2 G94E;PMID:11121101;

AA2 95D;Interacts with:109R,106N

AA2 97M;Interacts with:66I,111V,108G

AA2 98D;Interacts with:106N

//

MESH1 mesh:D000544

NAME1 Alzheimer Disease

CLUSTER1 clust2

MESH2 mesh:D006528

NAME2 Carcinoma, Hepatocellular

CLUSTER2 clust2

AC1 Q07820

PFAM1 PF00452

PDB1 2KBW:A

AC2 P55957

PFAM2 PF06393

PDB2 2KBW:A

INT edgetic

AA1 216V;Interacts with:97M

AA1 220V;Interacts with:97M

AA1 224H;Interacts with:93V

AA1 227A;Interacts with:93V

MUT1 A227V;169059;dbSNP:rs11580946;humsavar:VAR_024022;rs11580946;

AA1 231M;Interacts with:93V,90L,89H,86I

MUT1 M231L;898693;dbSNP:rs140449444;humsavar:VAR_054157;

AA1 234K;Interacts with:82I,86I

MUT1 K234R;898704;

AA1 235L;Interacts with:86I,82I,83I,79Q

AA1 236D;Interacts with:79Q

AA1 242D;Interacts with:79Q

AA1 245S;Interacts with:83I,79Q

AA1 248R;Interacts with:83I

AA1 249V;Interacts with:86I,83I,90L,87A

AA1 252H;Interacts with:84R,87A

AA1 253V;Interacts with:88R,90L,91A,87A

AA1 260N;Interacts with:95D,98D,94G

AA1 261W;Interacts with:98D

AA1 262G;Interacts with:95D,97M,94G,98D

AA1 263R;Interacts with:95D,98D,91A,94G

AA1 265V;Interacts with:97M

AA1 266T;Interacts with:93V,97M,90L,95D,94G

AA1 267L;Interacts with:90L

AA1 270F;Interacts with:90L

AA2 79Q;Interacts with:242D,236D,245S,235L

AA2 82I;Interacts with:234K,235L

AA2 83I;Interacts with:245S,249V,235L,248R

AA2 84R;Interacts with:252H

MUT2 R84W;dbSNP:rs148107209;dbSNP:rs148107209;

AA2 86I;Interacts with:235L,249V,234K,231M

AA2 87A;Interacts with:252H,253V,249V

AA2 88R;Interacts with:253V

AA2 89H;Interacts with:231M

AA2 90L;Interacts with:253V,231M,249V,266T,270F,267L

AA2 91A;Interacts with:253V,263R

AA2 93V;Interacts with:266T,231M,227A,224H

AA2 94G;Interacts with:263R,262G,266T,260N

MUT2 G94E;PMID:11121101;

AA2 95D;Interacts with:263R,266T,262G,260N

AA2 97M;Interacts with:266T,265V,216V,262G,220V

AA2 98D;Interacts with:261W,263R,262G,260N

//

MESH1 mesh:D000544

NAME1 Alzheimer Disease

CLUSTER1 clust2

MESH2 mesh:D008175

NAME2 Lung Neoplasms

CLUSTER2 clust2

AC1 P10415

PFAM1 PF00452

PDB1 4AQ3:A

AC2 Q07817

PFAM2 PF00452

PDB2 4AQ3:C

INT non-edgetic

AA1 102D;Interacts with:100R

AA1 103D;Interacts with:100R,99L,97F

AA1 104F;Interacts with:96E

AA1 106R;Interacts with:96E

AA1 107R;Interacts with:95D,96E,92E,91R

AA1 108Y;Interacts with:92E

AA1 115M;Interacts with:117G

AA1 118Q;Interacts with:116P,115T,117G

AA1 119L;Interacts with:117G

AA1 120H;Interacts with:115T

AA1 129R;Interacts with:117G

MUT1 R129C;828636;

AA1 132T;Interacts with:117G

AA1 133V;Interacts with:117G

AA1 135E;Interacts with:120Y

AA1 136E;Interacts with:120Y

MUT1 E136R;PMID:11461956;

AA1 98R;Interacts with:100R

AA1 99Q;Interacts with:100R,101Y

AA2 100R;Interacts with:103D,102D,99Q,98R

AA2 101Y;Interacts with:99Q

AA2 115T;Interacts with:118Q,120H

AA2 116P;Interacts with:118Q

AA2 117G;Interacts with:132T,129R,115M,133V,119L,118Q

AA2 120Y;Interacts with:135E,136E

AA2 91R;Interacts with:107R

AA2 92E;Interacts with:108Y,107R

AA2 95D;Interacts with:107R

AA2 96E;Interacts with:107R,104F,106R

AA2 97F;Interacts with:103D

AA2 99L;Interacts with:103D

//

MESH1 mesh:D000544

NAME1 Alzheimer Disease

CLUSTER1 clust2

MESH2 mesh:D008175

NAME2 Lung Neoplasms

CLUSTER2 clust2

AC1 P10415

PFAM1 PF02180

PDB1 4AQ3:A

AC2 Q07817

PFAM2 PF00452

PDB2 4AQ3:A

INT non-edgetic

AA1 11N;Interacts with:188W,171A,175N,174L,179E

AA1 12R;Interacts with:171A,175N

MUT1 R12Q;PMID:9463381;

AA1 14I;Interacts with:90L,188W,144F

AA1 15V;Interacts with:170M,174L,167A,144F,171A

AA1 16M;Interacts with:167A

AA1 17K;Interacts with:91R

AA1 18Y;Interacts with:145S,144F,90L,91R,148G,170M,95D

AA1 19I;Interacts with:163V,167A,170M

AA1 21Y;Interacts with:91R

AA1 22K;Interacts with:95D,152V,145S,98E,91R

AA1 23L;Interacts with:155V,163V,152V,151C

AA1 25Q;Interacts with:95D,183Q

AA1 26R;Interacts with:179E,184E,180P,183Q

AA1 27G;Interacts with:183Q

AA1 28Y;Interacts with:155V

AA1 30W;Interacts with:167A,163V,164S,160Q

AA1 92V;Interacts with:90L

AA1 9Y;Interacts with:188W

AA2 144F;Interacts with:18Y,15V,14I

AA2 145S;Interacts with:18Y,22K

AA2 148G;Interacts with:18Y

MUT2 G148E;827593;

AA2 151C;Interacts with:23L

AA2 152V;Interacts with:23L,22K

AA2 155V;Interacts with:23L,28Y

AA2 160Q;Interacts with:30W

AA2 163V;Interacts with:19I,23L,30W

AA2 164S;Interacts with:30W

AA2 167A;Interacts with:15V,19I,30W,16M

MUT2 A167V;1KG:1359392;

AA2 170M;Interacts with:15V,19I,18Y

AA2 171A;Interacts with:11N,15V,12R

AA2 174L;Interacts with:15V,11N

AA2 175N;Interacts with:11N,12R

AA2 179E;Interacts with:26R,11N

AA2 180P;Interacts with:26R

AA2 183Q;Interacts with:25Q,27G,26R

AA2 184E;Interacts with:26R

AA2 188W;Interacts with:11N,14I,9Y

AA2 90L;Interacts with:14I,18Y,92V

AA2 91R;Interacts with:17K,21Y,18Y,22K

AA2 95D;Interacts with:22K,25Q,18Y

AA2 98E;Interacts with:22K

//

MESH1 mesh:D000544

NAME1 Alzheimer Disease

CLUSTER1 clust2

MESH2 mesh:D008175

NAME2 Lung Neoplasms

CLUSTER2 clust2

AC1 O43521

PFAM1 PF08945

PDB1 3FDL:B

AC2 Q07817

PFAM2 PF00452

PDB2 3FDL:B

INT non-edgetic

AA1 143R;Interacts with:111Q

MUT1 R143H;dbSNP:rs141962978;dbSNP:rs141962978;dbSNP:rs141962978;dbSNP:rs141962978;dbSNP:rs141962978;

AA1 144P;Interacts with:112L,111Q

AA1 145E;Interacts with:126V,113H,125Q,122S,112L

AA1 146I;Interacts with:125Q

AA1 147W;Interacts with:107D,108L

AA1 148I;Interacts with:126V,108L,146F,111Q,112L,104A

AA1 149A;Interacts with:126V,129E,130L

AA1 150Q;Interacts with:129E

AA1 151E;Interacts with:108L

AA1 152L;Interacts with:142A,130L,97F,146F,105F,108L,126V,145S,104A

AA1 153R;Interacts with:130L,133D,139R,129E

MUT1 R153W;dbSNP:rs146318804;dbSNP:rs146318804;dbSNP:rs146318804;dbSNP:rs146318804;dbSNP:rs146318804;

AA1 155I;Interacts with:97F,101Y,105F,108L,104A

AA1 156G;Interacts with:136N,97F,142A,139R,138G,101Y

AA1 157D;Interacts with:138G,139R,136N

AA1 158E;Interacts with:100R,101Y

AA1 159F;Interacts with:97F,96E,93A,141V,138G,101Y,100R

AA1 160N;Interacts with:137W,136N,138G,139R

AA1 162Y;Interacts with:100R

AA1 163Y;Interacts with:100R

AA1 1M;Interacts with:113H,111Q,112L

AA2 100R;Interacts with:158E,159F,162Y,163Y

AA2 101Y;Interacts with:155I,159F,158E,156G

AA2 104A;Interacts with:155I,152L,148I

AA2 105F;Interacts with:155I,152L

AA2 107D;Interacts with:147W

AA2 108L;Interacts with:155I,147W,148I,152L,151E

MUT2 L108R;dbSNP:rs139457299;dbSNP:rs139457299;

AA2 111Q;Interacts with:143R,148I,1M,144P

AA2 112L;Interacts with:148I,1M,145E,144P

AA2 113H;Interacts with:1M,145E

AA2 122S;Interacts with:145E

AA2 125Q;Interacts with:145E,146I

AA2 126V;Interacts with:145E,149A,148I,152L

AA2 129E;Interacts with:149A,150Q,153R

AA2 130L;Interacts with:153R,152L,149A

AA2 133D;Interacts with:153R

AA2 136N;Interacts with:156G,160N,157D

AA2 137W;Interacts with:160N

AA2 138G;Interacts with:157D,160N,159F,156G

MUT2 G138A;827592;

AA2 139R;Interacts with:157D,156G,153R,160N

AA2 141V;Interacts with:159F

AA2 142A;Interacts with:156G,152L

AA2 145S;Interacts with:152L

AA2 146F;Interacts with:152L,148I

AA2 93A;Interacts with:159F

AA2 96E;Interacts with:159F

AA2 97F;Interacts with:155I,159F,156G,152L

//

MESH1 mesh:D000544

NAME1 Alzheimer Disease

CLUSTER1 clust2

MESH2 mesh:D008175

NAME2 Lung Neoplasms

CLUSTER2 clust2

AC1 Q13794

PFAM1 PF15150

PDB1 3MQP:B

AC2 Q16548

PFAM2 PF00452

PDB2 3MQP:B

INT non-edgetic

AA1 21L;Interacts with:55C,59V,58N

AA1 22E;Interacts with:74V,73Q

MUT1 E22K;dbSNP:rs1126436;

AA1 24E;Interacts with:52L

AA1 25C;Interacts with:74V,52L

AA1 26A;Interacts with:77K,78E,74V

AA1 28Q;Interacts with:51N

AA1 29L;Interacts with:91T,48V,74V,78E,95F

MUT1 L29A;823237;

MUT1 L29E;823238;

AA1 30R;Interacts with:78E,81D,77K,80E,88R,91T

MUT1 R30delR;COSMIC:166155;

AA1 32F;Interacts with:47E,91T,48V

MUT1 F32I;823240;

MUT1 F32E;823239;

AA1 33G;Interacts with:91T,88R,85N,87G,78E

AA1 34D;Interacts with:87G,85N,88R

AA1 36L;Interacts with:87G,44V,40V,90V

MUT1 L36E;823242;

AA1 37N;Interacts with:86W,87G,88R,85N

AA2 40V;Interacts with:36L

AA2 44V;Interacts with:36L

AA2 47E;Interacts with:32F

AA2 48V;Interacts with:32F,29L

AA2 51N;Interacts with:28Q

AA2 52L;Interacts with:25C,24E

AA2 55C;Interacts with:21L

AA2 58N;Interacts with:21L

MUT2 N58S;dbSNP:rs141166047;dbSNP:rs141166047;

AA2 59V;Interacts with:21L

AA2 73Q;Interacts with:22E

AA2 74V;Interacts with:25C,29L,26A,22E

AA2 77K;Interacts with:26A,30R

AA2 78E;Interacts with:26A,30R,29L,33G

AA2 80E;Interacts with:30R

AA2 81D;Interacts with:30R

AA2 85N;Interacts with:34D,33G,37N

AA2 86W;Interacts with:37N

AA2 87G;Interacts with:36L,34D,37N,33G

AA2 88R;Interacts with:33G,37N,30R,34D

AA2 90V;Interacts with:36L

AA2 91T;Interacts with:29L,32F,33G,30R

AA2 95F;Interacts with:29L

//

MESH1 mesh:D000544

NAME1 Alzheimer Disease

CLUSTER1 clust2

MESH2 mesh:D008175

NAME2 Lung Neoplasms

CLUSTER2 clust2

AC1 Q969J5

PFAM1 PF09294

PDB1 3G9V:C

AC2 Q9GZX6

PFAM2 PF14565

PDB2 3G9V:C

INT non-edgetic

AA1 200E;Interacts with:175R

AA1 246P;Interacts with:50P

AA1 247M;Interacts with:54N,50P,51Y,53T,48Q

AA1 248L;Interacts with:54N,53T,50P

AA1 249D;Interacts with:54N,50P

AA1 250R;Interacts with:54N

AA2 175R;Interacts with:200E

AA2 48Q;Interacts with:247M

AA2 50P;Interacts with:247M,249D,248L,246P

AA2 51Y;Interacts with:247M

AA2 53T;Interacts with:247M,248L

AA2 54N;Interacts with:247M,248L,250R,249D

//

MESH1 mesh:D000544

NAME1 Alzheimer Disease

CLUSTER1 clust2

MESH2 mesh:D008175

NAME2 Lung Neoplasms

CLUSTER2 clust2

AC1 Q969J5

PFAM1 PF01108

PDB1 3G9V:A

AC2 Q9GZX6

PFAM2 PF14565

PDB2 3G9V:A

INT non-edgetic

AA1 100G;Interacts with:70T,71D,72V,73R

AA1 101Q;Interacts with:71D,72V,70T

AA1 102R;Interacts with:71D

AA1 103Q;Interacts with:71D

AA1 104W;Interacts with:70T,71D

AA1 128Q;Interacts with:175R,73R,176N

AA1 129E;Interacts with:73R

AA1 130P;Interacts with:73R

AA1 132Y;Interacts with:70T

AA1 65K;Interacts with:70T,71D

AA1 66I;Interacts with:73R

AA1 99Y;Interacts with:70T,169L,72V,69N,73R

AA2 169L;Interacts with:99Y

AA2 175R;Interacts with:128Q

AA2 176N;Interacts with:128Q

AA2 69N;Interacts with:99Y

AA2 70T;Interacts with:104W,99Y,132Y,100G,65K,101Q

AA2 71D;Interacts with:101Q,103Q,100G,104W,65K,102R

AA2 72V;Interacts with:99Y,100G,101Q

AA2 73R;Interacts with:129E,130P,128Q,100G,99Y,66I

MUT2 R73H;dbSNP:rs149366319;

//

MESH1 mesh:D000544

NAME1 Alzheimer Disease

CLUSTER1 clust2

MESH2 mesh:D008175

NAME2 Lung Neoplasms

CLUSTER2 clust2

AC1 Q8N6P7

PFAM1 PF09294

PDB1 3DGC:R

AC2 Q9GZX6

PFAM2 PF14565

PDB2 3DGC:R

INT non-edgetic

AA1 133R;Interacts with:116Q,117E

AA1 134S;Interacts with:124R

AA1 136Q;Interacts with:124R

AA1 162D;Interacts with:175R,48Q,44K

AA1 175Y;Interacts with:51Y

AA1 179L;Interacts with:45S

AA1 180G;Interacts with:48Q

AA1 183Q;Interacts with:45S,43D,44K

AA1 185E;Interacts with:128R,124R,43D

AA1 186Y;Interacts with:128R

AA1 187E;Interacts with:124R,49Q,128R,45S

AA1 189F;Interacts with:46N,52I,49Q,121F,124R

AA1 190G;Interacts with:51Y,52I,49Q

AA1 191L;Interacts with:117E

AA1 206P;Interacts with:48Q

AA1 207T;Interacts with:172M,175R,48Q,44K

AA1 208W;Interacts with:57F,172M

AA2 116Q;Interacts with:133R

AA2 117E;Interacts with:191L,133R

AA2 121F;Interacts with:189F

AA2 124R;Interacts with:187E,185E,189F,134S,136Q

AA2 128R;Interacts with:187E,185E,186Y

AA2 172M;Interacts with:207T,208W

MUT2 M172V;dbSNP:rs147812317;

AA2 175R;Interacts with:162D,207T

AA2 43D;Interacts with:183Q,185E

AA2 44K;Interacts with:162D,207T,183Q

AA2 45S;Interacts with:179L,183Q,187E

AA2 46N;Interacts with:189F

AA2 48Q;Interacts with:206P,162D,207T,180G

AA2 49Q;Interacts with:187E,189F,190G

AA2 51Y;Interacts with:190G,175Y

AA2 52I;Interacts with:189F,190G

AA2 57F;Interacts with:208W

//

MESH1 mesh:D000544

NAME1 Alzheimer Disease

CLUSTER1 clust2

MESH2 mesh:D008175

NAME2 Lung Neoplasms

CLUSTER2 clust2

AC1 Q8N6P7

PFAM1 PF01108

PDB1 3DGC:R

AC2 Q9GZX6

PFAM2 PF14565

PDB2 3DGC:R

INT non-edgetic

AA1 58K;Interacts with:71D,70T

MUT1 K58A;882419;

AA1 60Y;Interacts with:72V,70T,169L,73R,69N,74L

MUT1 Y60A;1368880;

MUT1 Y60R;1368881;

AA1 61G;Interacts with:73R,72V,70T,71D

AA1 62E;Interacts with:72V,70T,71D

AA1 63R;Interacts with:71D

AA1 64D;Interacts with:71D

AA1 65W;Interacts with:70T

AA1 89T;Interacts with:77E,73R

AA1 90E;Interacts with:73R

AA1 91L;Interacts with:73R,169L

MUT1 L91F;dbSNP:rs144035143;

AA1 93Y;Interacts with:70T

AA2 169L;Interacts with:60Y,91L

AA2 69N;Interacts with:60Y

AA2 70T;Interacts with:58K,61G,60Y,62E,93Y,65W

AA2 71D;Interacts with:58K,61G,63R,64D,62E

AA2 72V;Interacts with:62E,60Y,61G

AA2 73R;Interacts with:61G,90E,89T,60Y,91L

MUT2 R73H;dbSNP:rs149366319;

AA2 74L;Interacts with:60Y

AA2 77E;Interacts with:89T

//

MESH1 mesh:D000544

NAME1 Alzheimer Disease

CLUSTER1 clust2

MESH2 mesh:D008175

NAME2 Lung Neoplasms

CLUSTER2 clust2

AC1 P22301

PFAM1 PF00726

PDB1 1J7V:L

AC2 Q13651

PFAM2 PF09294

PDB2 1J7V:R

INT non-edgetic

AA1 38P;Interacts with:212R,211S,164F,210A

AA1 39N;Interacts with:210A

AA1 41L;Interacts with:211S

AA1 42R;Interacts with:211S,214N,208S,212R,166E,210A,213S

AA1 43D;Interacts with:211S

AA1 45R;Interacts with:211S

AA1 46D;Interacts with:213S

AA2 164F;Interacts with:38P

AA2 166E;Interacts with:42R

AA2 208S;Interacts with:42R

AA2 210A;Interacts with:39N,42R,38P

AA2 211S;Interacts with:42R,38P,41L,45R,43D

AA2 212R;Interacts with:38P,42R

AA2 213S;Interacts with:46D,42R

AA2 214N;Interacts with:42R

//

MESH1 mesh:D000544

NAME1 Alzheimer Disease

CLUSTER1 clust2

MESH2 mesh:D008175

NAME2 Lung Neoplasms

CLUSTER2 clust2

AC1 P22301

PFAM1 PF00726

PDB1 1J7V:L

AC2 Q13651

PFAM2 PF01108

PDB2 1J7V:R

INT non-edgetic

AA1 56Q;Interacts with:97R

AA1 59D;Interacts with:97R

AA1 60Q;Interacts with:97R

MUT1 Q60E;PMID:10637267;

AA1 61L;Interacts with:97R,64Y

AA1 62D;Interacts with:69W,66I,65G,67E,97R,64Y

AA1 63N;Interacts with:67E,65G,64Y

AA1 64L;Interacts with:64Y,67E,65G

AA1 65L;Interacts with:64Y

AA1 66L;Interacts with:94N

AA2 64Y;Interacts with:65L,64L,61L,62D,63N

AA2 65G;Interacts with:62D,63N,64L

AA2 66I;Interacts with:62D

AA2 67E;Interacts with:63N,62D,64L

AA2 69W;Interacts with:62D

AA2 94N;Interacts with:66L

AA2 97R;Interacts with:60Q,61L,59D,62D,56Q

//

MESH1 mesh:D000544

NAME1 Alzheimer Disease

CLUSTER1 clust2

MESH2 mesh:D008175

NAME2 Lung Neoplasms

CLUSTER2 clust2

AC1 O43521

PFAM1 PF08945

PDB1 2VM6:B

AC2 Q16548

PFAM2 PF00452

PDB2 2VM6:B

INT non-edgetic

AA1 142M;Interacts with:55C,58N

MUT1 M142R;dbSNP:rs114585494;dbSNP:rs114585494;dbSNP:rs114585494;dbSNP:rs114585494;dbSNP:rs114585494;

AA1 144P;Interacts with:70L,74V

AA1 145E;Interacts with:70L,77K,73Q,74V

AA1 147W;Interacts with:54S,55C,52L,51N

AA1 148I;Interacts with:70L,74V,52L,95F,56L,59V

AA1 149A;Interacts with:77K,78E,74V

AA1 151E;Interacts with:51N,48V

AA1 152L;Interacts with:78E,48V,95F,91T,74V

AA1 153R;Interacts with:79F,88R,80E,78E,81D,91T,77K

MUT1 R153W;dbSNP:rs146318804;dbSNP:rs146318804;dbSNP:rs146318804;dbSNP:rs146318804;dbSNP:rs146318804;

AA1 154R;Interacts with:47E,51N

AA1 155I;Interacts with:48V,91T,44V,47E

AA1 156G;Interacts with:87G,91T,85N,88R

AA1 157D;Interacts with:88R,87G,85N

AA1 159F;Interacts with:40V,43S,44V,87G

AA1 160N;Interacts with:86W,87G,85N

AA1 163Y;Interacts with:40V

AA2 40V;Interacts with:163Y,159F

AA2 43S;Interacts with:159F

AA2 44V;Interacts with:155I,159F

AA2 47E;Interacts with:154R,155I

AA2 48V;Interacts with:155I,152L,151E

AA2 51N;Interacts with:147W,154R,151E

AA2 52L;Interacts with:148I,147W

AA2 54S;Interacts with:147W

AA2 55C;Interacts with:147W,142M

AA2 56L;Interacts with:148I

AA2 58N;Interacts with:142M

MUT2 N58S;dbSNP:rs141166047;dbSNP:rs141166047;

AA2 59V;Interacts with:148I

AA2 70L;Interacts with:145E,148I,144P

AA2 73Q;Interacts with:145E

AA2 74V;Interacts with:148I,144P,152L,149A,145E

AA2 77K;Interacts with:149A,145E,153R

AA2 78E;Interacts with:152L,153R,149A

AA2 79F;Interacts with:153R

AA2 80E;Interacts with:153R

AA2 81D;Interacts with:153R

AA2 85N;Interacts with:157D,156G,160N

AA2 86W;Interacts with:160N

AA2 87G;Interacts with:157D,156G,160N,159F

AA2 88R;Interacts with:157D,153R,156G

AA2 91T;Interacts with:152L,155I,156G,153R

AA2 95F;Interacts with:152L,148I

//

MESH1 mesh:D000544

NAME1 Alzheimer Disease

CLUSTER1 clust2

MESH2 mesh:D008175

NAME2 Lung Neoplasms

CLUSTER2 clust2

AC1 O43521

PFAM1 PF08945

PDB1 2NL9:B

AC2 Q07820

PFAM2 PF00452

PDB2 2NL9:B

INT semi-edgetic

AA1 143R;Interacts with:234K

MUT1 R143H;dbSNP:rs141962978;dbSNP:rs141962978;dbSNP:rs141962978;dbSNP:rs141962978;dbSNP:rs141962978;

AA1 144P;Interacts with:234K,235L

AA1 145E;Interacts with:248R,249V,245S,252H,235L

AA1 146I;Interacts with:252H

AA1 147W;Interacts with:230G,231M

AA1 148I;Interacts with:270F,235L,234K,249V,231M

AA1 149A;Interacts with:249V,252H,253V

AA1 151E;Interacts with:231M

AA1 152L;Interacts with:228F,270F,253V,249V,266T,267L,231M

AA1 153R;Interacts with:252H,253V,254F,255S,256D,263R

MUT1 R153W;dbSNP:rs146318804;dbSNP:rs146318804;dbSNP:rs146318804;dbSNP:rs146318804;dbSNP:rs146318804;

AA1 155I;Interacts with:266T,220V,228F,227A,224H,231M

AA1 156G;Interacts with:262G,260N,266T,263R

AA1 157D;Interacts with:260N,263R,262G

AA1 158E;Interacts with:224H

AA1 159F;Interacts with:265V,220V,262G,216V,219G,224H

AA1 160N;Interacts with:261W,262G,260N,263R

AA1 163Y;Interacts with:215R,216V

AA2 215R;Interacts with:163Y

AA2 216V;Interacts with:159F,163Y

AA2 219G;Interacts with:159F

AA2 220V;Interacts with:159F,155I

AA2 224H;Interacts with:158E,155I,159F

AA2 227A;Interacts with:155I

MUT2 A227V;169059;dbSNP:rs11580946;humsavar:VAR_024022;rs11580946;

AA2 228F;Interacts with:152L,155I

AA2 230G;Interacts with:147W

AA2 231M;Interacts with:147W,151E,148I,152L,155I

MUT2 M231L;898693;dbSNP:rs140449444;humsavar:VAR_054157;

AA2 234K;Interacts with:148I,144P,143R

MUT2 K234R;898704;

AA2 235L;Interacts with:148I,145E,144P

AA2 245S;Interacts with:145E

AA2 248R;Interacts with:145E

AA2 249V;Interacts with:149A,152L,145E,148I

AA2 252H;Interacts with:153R,149A,146I,145E

AA2 253V;Interacts with:152L,153R,149A

AA2 254F;Interacts with:153R

AA2 255S;Interacts with:153R

AA2 256D;Interacts with:153R

AA2 260N;Interacts with:156G,157D,160N

AA2 261W;Interacts with:160N

AA2 262G;Interacts with:159F,156G,160N,157D

AA2 263R;Interacts with:160N,156G,153R,157D

AA2 265V;Interacts with:159F

AA2 266T;Interacts with:155I,152L,156G

AA2 267L;Interacts with:152L

AA2 270F;Interacts with:148I,152L

//

MESH1 mesh:D000544

NAME1 Alzheimer Disease

CLUSTER1 clust2

MESH2 mesh:D008175

NAME2 Lung Neoplasms

CLUSTER2 clust2

AC1 O43521

PFAM1 PF08945

PDB1 2K7W:B

AC2 Q07812

PFAM2 PF00452

PDB2 2K7W:B

INT semi-edgetic

AA1 145E;Interacts with:146E,142D,89R

AA1 146I;Interacts with:142D

AA1 148I;Interacts with:142D

AA1 149A;Interacts with:138G,142D,139W

AA1 152L;Interacts with:142D,141L,145R,138G

AA1 153R;Interacts with:138G,131E,135T,134R

MUT1 R153W;dbSNP:rs146318804;dbSNP:rs146318804;dbSNP:rs146318804;dbSNP:rs146318804;dbSNP:rs146318804;

AA1 156G;Interacts with:141L,137M,134R

AA1 157D;Interacts with:134R

AA1 160N;Interacts with:134R

AA2 131E;Interacts with:153R

AA2 134R;Interacts with:157D,153R,156G,160N

AA2 135T;Interacts with:153R

AA2 137M;Interacts with:156G

AA2 138G;Interacts with:149A,153R,152L

AA2 139W;Interacts with:149A

MUT2 W139C;COSMIC:267934;

AA2 141L;Interacts with:156G,152L

AA2 142D;Interacts with:145E,149A,152L,148I,146I

AA2 145R;Interacts with:152L

AA2 146E;Interacts with:145E

AA2 89R;Interacts with:145E

MUT2 R89Q;1KG:1191554;1KG:1224249;1KG:1224241;

//

MESH1 mesh:D000544

NAME1 Alzheimer Disease

CLUSTER1 clust2

MESH2 mesh:D008175

NAME2 Lung Neoplasms

CLUSTER2 clust2

AC1 Q13324

PFAM1 PF02793

PDB1 3N95:C

AC2 Q96RP3

PFAM2 PF11613

PDB2 3N95:E

INT semi-edgetic

AA1 47I;Interacts with:105I,106L,109V

AA1 68F;Interacts with:105I,106L,102N

AA1 69N;Interacts with:98Q,102N

AA1 71V;Interacts with:99A,102N,98Q

AA1 73Y;Interacts with:106L,103A,102N

AA1 92K;Interacts with:107A,109V

MUT1 K92N;dbSNP:rs148406223;dbSNP:rs148406223;

AA1 93I;Interacts with:109V

MUT1 I93S;dbSNP:rs144584262;dbSNP:rs144584262;

AA1 94N;Interacts with:109V

AA1 95Y;Interacts with:106L,109V

AA1 96S;Interacts with:106L

AA2 102N;Interacts with:71V,69N,73Y,68F

AA2 103A;Interacts with:73Y

AA2 105I;Interacts with:47I,68F

AA2 106L;Interacts with:96S,47I,73Y,95Y,68F

AA2 107A;Interacts with:92K

AA2 109V;Interacts with:47I,94N,95Y,92K,93I

AA2 98Q;Interacts with:69N,71V

AA2 99A;Interacts with:71V

//

MESH1 mesh:D000544

NAME1 Alzheimer Disease

CLUSTER1 clust2

MESH2 mesh:D008175

NAME2 Lung Neoplasms

CLUSTER2 clust2

AC1 P23560

PFAM1 PF00243

PDB1 1BND:A

AC2 P20783

PFAM2 PF00243

PDB2 1BND:B

INT edgetic

AA1 136G;Interacts with:252R,251S

AA1 137E;Interacts with:251S,250L,216Q

AA1 138L;Interacts with:250L,249A,213W,251S

AA1 139S;Interacts with:250L,248C

AA1 140V;Interacts with:208I,248C,151V,250L

AA1 141C;Interacts with:248C

AA1 147W;Interacts with:223Y,168I,239W

AA1 159M;Interacts with:158W

AA1 172V;Interacts with:237W

AA1 173S;Interacts with:181T

AA1 177L;Interacts with:237W

AA1 178K;Interacts with:225R

AA1 180Y;Interacts with:225R,239W

AA1 181F;Interacts with:239W

AA1 182Y;Interacts with:223Y,222T

AA1 184T;Interacts with:222T

AA1 197R;Interacts with:250L

AA1 198G;Interacts with:209D,213W,250L,208I

AA1 199I;Interacts with:207G,209D,151V,208I

AA1 200D;Interacts with:207G,209D,208I

AA1 202R;Interacts with:209D

AA1 204W;Interacts with:207G,149Y

AA1 209R;Interacts with:148E

AA1 213S;Interacts with:191Y

AA1 214Y;Interacts with:158W,191Y

AA1 215V;Interacts with:224V

AA1 216R;Interacts with:187K,189Y

AA1 228W;Interacts with:237W,182G,186V

AA1 230F;Interacts with:189Y,158W

AA1 235T;Interacts with:222T,244T

AA1 237C;Interacts with:247V

AA1 238V;Interacts with:247V

AA1 239C;Interacts with:151V,150S,152C

AA1 240T;Interacts with:149Y,150S

AA1 241L;Interacts with:151V,207G,150S,149Y,148E,206R

AA1 242T;Interacts with:148E,149Y,147G

AA1 243I;Interacts with:149Y,147G

AA2 147G;Interacts with:242T,243I

AA2 148E;Interacts with:209R,242T,241L

AA2 149Y;Interacts with:240T,242T,204W,243I,241L

AA2 150S;Interacts with:239C,241L,240T

AA2 151V;Interacts with:239C,140V,199I,241L

AA2 152C;Interacts with:239C

AA2 158W;Interacts with:214Y,159M,230F

AA2 168I;Interacts with:147W

AA2 181T;Interacts with:173S

AA2 182G;Interacts with:228W

AA2 186V;Interacts with:228W

AA2 187K;Interacts with:216R

AA2 189Y;Interacts with:230F,216R

AA2 191Y;Interacts with:213S,214Y

AA2 206R;Interacts with:241L

AA2 207G;Interacts with:200D,204W,199I,241L

AA2 208I;Interacts with:140V,200D,199I,198G

AA2 209D;Interacts with:198G,200D,199I,202R

AA2 213W;Interacts with:198G,138L

MUT2 W213L;dbSNP:rs73036423;dbSNP:rs73036423;

AA2 216Q;Interacts with:137E

AA2 222T;Interacts with:235T,184T,182Y

AA2 223Y;Interacts with:182Y,147W

AA2 224V;Interacts with:215V

AA2 225R;Interacts with:180Y,178K

AA2 237W;Interacts with:172V,228W,177L

AA2 239W;Interacts with:180Y,181F,147W

AA2 244T;Interacts with:235T

AA2 247V;Interacts with:237C,238V

AA2 248C;Interacts with:140V,141C,139S

AA2 249A;Interacts with:138L

AA2 250L;Interacts with:139S,138L,197R,137E,140V,198G

AA2 251S;Interacts with:137E,138L,136G

AA2 252R;Interacts with:136G

//

MESH1 mesh:D000544

NAME1 Alzheimer Disease

CLUSTER1 clust2

MESH2 mesh:D008175

NAME2 Lung Neoplasms

CLUSTER2 clust2

AC1 Q30201

PFAM1 PF00129

PDB1 1DE4:A

AC2 P02786

PFAM2 PF04253

PDB2 1DE4:C

INT edgetic

AA1 100V;Interacts with:643Y

MUT1 V100A;PMID:10369785;

AA1 104T;Interacts with:640Q

AA1 168E;Interacts with:640Q,639L

MUT1 E168Q;dbSNP:rs146519482;

AA1 171R;Interacts with:641W

AA1 172H;Interacts with:644S,641W,640Q

AA1 174I;Interacts with:641W

AA1 175R;Interacts with:640Q

AA1 178Q;Interacts with:651R,648D,644S

AA1 82Q;Interacts with:662N

AA1 85L;Interacts with:658T,661G,657T

AA1 86Q;Interacts with:658T

AA1 89Q;Interacts with:650F,657T,653T,654S,658T,655R

AA1 90S;Interacts with:654S

AA1 92K;Interacts with:650F

AA1 93G;Interacts with:646R,650F

MUT1 G93R;156025;dbSNP:rs28934597;dbSNP:rs28934597;dbSNP:rs28934597;humsavar:VAR_008729;rs28934597, phenotype=Hemochromatosis 1 (HFE1);clinvar:15052, phenotype=HFE hemochromatosis, modifier of;HEMOCHROMATOSIS, TYPE 1;

AA1 96H;Interacts with:646R,650F

AA1 97M;Interacts with:646R

AA2 639L;Interacts with:168E

AA2 640Q;Interacts with:175R,172H,168E,104T

MUT2 Q640A;960798;

AA2 641W;Interacts with:174I,172H,171R

MUT2 W641A;960799;

AA2 643Y;Interacts with:100V

MUT2 Y643A;960800;

MUT2 Y643D;dbSNP:rs79915860;

AA2 644S;Interacts with:172H,178Q

MUT2 S644A;960801;

AA2 646R;Interacts with:93G,96H,97M

MUT2 R646K;960802;

MUT2 R646H;1073985;

MUT2 R646A;1073984;

AA2 648D;Interacts with:178Q

MUT2 D648A;960804;

MUT2 D648E;960805;

AA2 650F;Interacts with:89Q,93G,92K,96H

MUT2 F650Q;960806;

AA2 651R;Interacts with:178Q

AA2 653T;Interacts with:89Q

AA2 654S;Interacts with:89Q,90S

AA2 655R;Interacts with:89Q

AA2 657T;Interacts with:85L,89Q

AA2 658T;Interacts with:86Q,85L,89Q

MUT2 T658R;COSMIC:72959;

AA2 661G;Interacts with:85L

AA2 662N;Interacts with:82Q

//

MESH1 mesh:D000544

NAME1 Alzheimer Disease

CLUSTER1 clust2

MESH2 mesh:D015179

NAME2 Colorectal Neoplasms

CLUSTER2 clust2

AC1 P10415

PFAM1 PF00452

PDB1 4AQ3:A

AC2 Q07817

PFAM2 PF00452

PDB2 4AQ3:C

INT semi-edgetic

AA1 102D;Interacts with:100R

AA1 103D;Interacts with:100R,99L,97F

AA1 104F;Interacts with:96E

AA1 106R;Interacts with:96E

AA1 107R;Interacts with:95D,96E,92E,91R

AA1 108Y;Interacts with:92E

AA1 115M;Interacts with:117G

AA1 118Q;Interacts with:116P,115T,117G

AA1 119L;Interacts with:117G

AA1 120H;Interacts with:115T

AA1 129R;Interacts with:117G

MUT1 R129C;828636;

AA1 132T;Interacts with:117G

AA1 133V;Interacts with:117G

AA1 135E;Interacts with:120Y

AA1 136E;Interacts with:120Y

MUT1 E136R;PMID:11461956;

AA1 98R;Interacts with:100R

AA1 99Q;Interacts with:100R,101Y

AA2 100R;Interacts with:103D,102D,99Q,98R

AA2 101Y;Interacts with:99Q

AA2 115T;Interacts with:118Q,120H

AA2 116P;Interacts with:118Q

AA2 117G;Interacts with:132T,129R,115M,133V,119L,118Q

AA2 120Y;Interacts with:135E,136E

AA2 91R;Interacts with:107R

AA2 92E;Interacts with:108Y,107R

AA2 95D;Interacts with:107R

AA2 96E;Interacts with:107R,104F,106R

AA2 97F;Interacts with:103D

AA2 99L;Interacts with:103D

//

MESH1 mesh:D000544

NAME1 Alzheimer Disease

CLUSTER1 clust2

MESH2 mesh:D015179

NAME2 Colorectal Neoplasms

CLUSTER2 clust2

AC1 P10415

PFAM1 PF02180

PDB1 4AQ3:A

AC2 Q07817

PFAM2 PF00452

PDB2 4AQ3:A

INT semi-edgetic

AA1 11N;Interacts with:188W,171A,175N,174L,179E

AA1 12R;Interacts with:171A,175N

MUT1 R12Q;PMID:9463381;

AA1 14I;Interacts with:90L,188W,144F

AA1 15V;Interacts with:170M,174L,167A,144F,171A

AA1 16M;Interacts with:167A

AA1 17K;Interacts with:91R

AA1 18Y;Interacts with:145S,144F,90L,91R,148G,170M,95D

AA1 19I;Interacts with:163V,167A,170M

AA1 21Y;Interacts with:91R

AA1 22K;Interacts with:95D,152V,145S,98E,91R

AA1 23L;Interacts with:155V,163V,152V,151C

AA1 25Q;Interacts with:95D,183Q

AA1 26R;Interacts with:179E,184E,180P,183Q

AA1 27G;Interacts with:183Q

AA1 28Y;Interacts with:155V

AA1 30W;Interacts with:167A,163V,164S,160Q

AA1 92V;Interacts with:90L

AA1 9Y;Interacts with:188W

AA2 144F;Interacts with:18Y,15V,14I

AA2 145S;Interacts with:18Y,22K

AA2 148G;Interacts with:18Y

MUT2 G148E;827593;

AA2 151C;Interacts with:23L

AA2 152V;Interacts with:23L,22K

AA2 155V;Interacts with:23L,28Y

AA2 160Q;Interacts with:30W

AA2 163V;Interacts with:19I,23L,30W

AA2 164S;Interacts with:30W

AA2 167A;Interacts with:15V,19I,30W,16M

MUT2 A167V;1KG:1359392;

AA2 170M;Interacts with:15V,19I,18Y

AA2 171A;Interacts with:11N,15V,12R

AA2 174L;Interacts with:15V,11N

AA2 175N;Interacts with:11N,12R

AA2 179E;Interacts with:26R,11N

AA2 180P;Interacts with:26R

AA2 183Q;Interacts with:25Q,27G,26R

AA2 184E;Interacts with:26R

AA2 188W;Interacts with:11N,14I,9Y

AA2 90L;Interacts with:14I,18Y,92V

AA2 91R;Interacts with:17K,21Y,18Y,22K

AA2 95D;Interacts with:22K,25Q,18Y

AA2 98E;Interacts with:22K

//

MESH1 mesh:D000544

NAME1 Alzheimer Disease

CLUSTER1 clust2

MESH2 mesh:D015179

NAME2 Colorectal Neoplasms

CLUSTER2 clust2

AC1 Q13794

PFAM1 PF15150

PDB1 3MQP:B

AC2 Q16548

PFAM2 PF00452

PDB2 3MQP:B

INT semi-edgetic

AA1 21L;Interacts with:55C,59V,58N

AA1 22E;Interacts with:74V,73Q

MUT1 E22K;dbSNP:rs1126436;

AA1 24E;Interacts with:52L

AA1 25C;Interacts with:74V,52L

AA1 26A;Interacts with:77K,78E,74V

AA1 28Q;Interacts with:51N

AA1 29L;Interacts with:91T,48V,74V,78E,95F

MUT1 L29A;823237;

MUT1 L29E;823238;

AA1 30R;Interacts with:78E,81D,77K,80E,88R,91T

MUT1 R30delR;COSMIC:166155;

AA1 32F;Interacts with:47E,91T,48V

MUT1 F32I;823240;

MUT1 F32E;823239;

AA1 33G;Interacts with:91T,88R,85N,87G,78E

AA1 34D;Interacts with:87G,85N,88R

AA1 36L;Interacts with:87G,44V,40V,90V

MUT1 L36E;823242;

AA1 37N;Interacts with:86W,87G,88R,85N

AA2 40V;Interacts with:36L

AA2 44V;Interacts with:36L

AA2 47E;Interacts with:32F

AA2 48V;Interacts with:32F,29L

AA2 51N;Interacts with:28Q

AA2 52L;Interacts with:25C,24E

AA2 55C;Interacts with:21L

AA2 58N;Interacts with:21L

MUT2 N58S;dbSNP:rs141166047;dbSNP:rs141166047;

AA2 59V;Interacts with:21L

AA2 73Q;Interacts with:22E

AA2 74V;Interacts with:25C,29L,26A,22E

AA2 77K;Interacts with:26A,30R

AA2 78E;Interacts with:26A,30R,29L,33G

AA2 80E;Interacts with:30R

AA2 81D;Interacts with:30R

AA2 85N;Interacts with:34D,33G,37N

AA2 86W;Interacts with:37N

AA2 87G;Interacts with:36L,34D,37N,33G

AA2 88R;Interacts with:33G,37N,30R,34D

AA2 90V;Interacts with:36L

AA2 91T;Interacts with:29L,32F,33G,30R

AA2 95F;Interacts with:29L

//

MESH1 mesh:D000544

NAME1 Alzheimer Disease

CLUSTER1 clust2

MESH2 mesh:D015179

NAME2 Colorectal Neoplasms

CLUSTER2 clust2

AC1 Q30201

PFAM1 PF00129

PDB1 1DE4:A

AC2 P02786

PFAM2 PF04253

PDB2 1DE4:C

INT edgetic

AA1 100V;Interacts with:643Y

MUT1 V100A;PMID:10369785;

AA1 104T;Interacts with:640Q

AA1 168E;Interacts with:640Q,639L

MUT1 E168Q;dbSNP:rs146519482;

AA1 171R;Interacts with:641W

AA1 172H;Interacts with:644S,641W,640Q

AA1 174I;Interacts with:641W

AA1 175R;Interacts with:640Q

AA1 178Q;Interacts with:651R,648D,644S

AA1 82Q;Interacts with:662N

AA1 85L;Interacts with:658T,661G,657T

AA1 86Q;Interacts with:658T

AA1 89Q;Interacts with:650F,657T,653T,654S,658T,655R

AA1 90S;Interacts with:654S

AA1 92K;Interacts with:650F

AA1 93G;Interacts with:646R,650F

MUT1 G93R;156025;dbSNP:rs28934597;dbSNP:rs28934597;dbSNP:rs28934597;humsavar:VAR_008729;rs28934597, phenotype=Hemochromatosis 1 (HFE1);clinvar:15052, phenotype=HFE hemochromatosis, modifier of;HEMOCHROMATOSIS, TYPE 1;

AA1 96H;Interacts with:646R,650F

AA1 97M;Interacts with:646R

AA2 639L;Interacts with:168E

AA2 640Q;Interacts with:175R,172H,168E,104T

MUT2 Q640A;960798;

AA2 641W;Interacts with:174I,172H,171R

MUT2 W641A;960799;

AA2 643Y;Interacts with:100V

MUT2 Y643A;960800;

MUT2 Y643D;dbSNP:rs79915860;

AA2 644S;Interacts with:172H,178Q

MUT2 S644A;960801;

AA2 646R;Interacts with:93G,96H,97M

MUT2 R646K;960802;

MUT2 R646H;1073985;

MUT2 R646A;1073984;

AA2 648D;Interacts with:178Q

MUT2 D648A;960804;

MUT2 D648E;960805;

AA2 650F;Interacts with:89Q,93G,92K,96H

MUT2 F650Q;960806;

AA2 651R;Interacts with:178Q

AA2 653T;Interacts with:89Q

AA2 654S;Interacts with:89Q,90S

AA2 655R;Interacts with:89Q

AA2 657T;Interacts with:85L,89Q

AA2 658T;Interacts with:86Q,85L,89Q

MUT2 T658R;COSMIC:72959;

AA2 661G;Interacts with:85L

AA2 662N;Interacts with:82Q

//

MESH1 mesh:D001172

NAME1 Arthritis, Rheumatoid

CLUSTER1 clust1

MESH2 mesh:D001249

NAME2 Asthma

CLUSTER2 clust1

AC1 P29459

PFAM1 PF03039

PDB1 3HMX:B

AC2 P29460

PFAM2 PF10420

PDB2 3HMX:A

INT non-edgetic

AA1 205R;Interacts with:203E

AA1 208T;Interacts with:203E,202A

AA1 211R;Interacts with:136Y

AA1 71H;Interacts with:205S

AA1 76K;Interacts with:205S

AA1 82V;Interacts with:201A,202A,203E

AA1 83E;Interacts with:201A,202A

AA1 85C;Interacts with:202A

AA1 86L;Interacts with:202A,200P,201A

AA2 136Y;Interacts with:211R

AA2 200P;Interacts with:86L

AA2 201A;Interacts with:82V,83E,86L

AA2 202A;Interacts with:85C,82V,86L,83E,208T

AA2 203E;Interacts with:208T,82V,205R

AA2 205S;Interacts with:71H,76K

//

MESH1 mesh:D001172

NAME1 Arthritis, Rheumatoid

CLUSTER1 clust1

MESH2 mesh:D001249

NAME2 Asthma

CLUSTER2 clust1

AC1 O14786

PFAM1 PF00754

PDB1 4DEQ:A

AC2 P15692

PFAM2 PF00754

PDB2 4DEQ:B

INT non-edgetic

AA1 319E;Interacts with:138R

AA1 323R;Interacts with:92L,45D,90E,91G

AA1 324E;Interacts with:138R

AA2 138R;Interacts with:324E,319E

AA2 45D;Interacts with:323R

AA2 90E;Interacts with:323R

AA2 91G;Interacts with:323R

AA2 92L;Interacts with:323R

//

MESH1 mesh:D001172

NAME1 Arthritis, Rheumatoid

CLUSTER1 clust1

MESH2 mesh:D001249

NAME2 Asthma

CLUSTER2 clust1

AC1 O14786

PFAM1 PF00754

PDB1 4DEQ:B

AC2 P15692

PFAM2 PF14554

PDB2 4DEQ:A

INT non-edgetic

AA1 297Y;Interacts with:231R,232R,230P

AA1 298S;Interacts with:219E,222E

AA1 299T;Interacts with:222E,219E

AA1 300N;Interacts with:210D,219E,231R

AA1 301W;Interacts with:219E,232R

AA1 316T;Interacts with:232R

AA1 320D;Interacts with:232R

AA1 348E;Interacts with:214K,231R

AA1 349T;Interacts with:231R,232R

AA1 353Y;Interacts with:232R

AA1 413T;Interacts with:232R

AA2 210D;Interacts with:300N

MUT2 D210H;971365;

AA2 214K;Interacts with:348E

AA2 219E;Interacts with:298S,301W,300N,299T

AA2 222E;Interacts with:298S,299T

AA2 230P;Interacts with:297Y

AA2 231R;Interacts with:297Y,349T,300N,348E

AA2 232R;Interacts with:320D,297Y,316T,301W,413T,349T,353Y

//

MESH1 mesh:D001172

NAME1 Arthritis, Rheumatoid

CLUSTER1 clust1

MESH2 mesh:D001249

NAME2 Asthma

CLUSTER2 clust1

AC1 P28068

PFAM1 PF07654

PDB1 4I0P:B

AC2 Q6ICR9

PFAM2 PF00993

PDB2 4I0P:A

INT non-edgetic

AA1 141Y;Interacts with:63Y

AA1 168P;Interacts with:65E

AA1 169N;Interacts with:66D,81R

AA1 170G;Interacts with:66D,81R

AA1 171D;Interacts with:66D,82L

AA1 172W;Interacts with:66D,68L,63Y,85F,67Q,82L

AA1 173T;Interacts with:66D

AA1 174Y;Interacts with:65E,66D

AA2 63Y;Interacts with:172W,141Y

AA2 65E;Interacts with:174Y,168P

AA2 66D;Interacts with:172W,173T,174Y,169N,170G,171D

AA2 67Q;Interacts with:172W

AA2 68L;Interacts with:172W

AA2 81R;Interacts with:170G,169N

AA2 82L;Interacts with:171D,172W

AA2 85F;Interacts with:172W

//

MESH1 mesh:D001172

NAME1 Arthritis, Rheumatoid

CLUSTER1 clust1

MESH2 mesh:D001249

NAME2 Asthma

CLUSTER2 clust1

AC1 P04440

PFAM1 PF07654

PDB1 3LQZ:B

AC2 P20036

PFAM2 PF00993

PDB2 3LQZ:A

INT non-edgetic

AA1 176R;Interacts with:59E,58D,60D

AA1 178G;Interacts with:60D,75H

AA1 179D;Interacts with:60D,75H

AA1 180W;Interacts with:62M,60D,61E,75H,57F,79F,76L

AA1 181T;Interacts with:60D

AA2 57F;Interacts with:180W

AA2 58D;Interacts with:176R

AA2 59E;Interacts with:176R

MUT2 E59D;157591;humsavar:VAR_058835;rs2308910;

AA2 60D;Interacts with:180W,179D,178G,176R,181T

AA2 61E;Interacts with:180W

AA2 62M;Interacts with:180W

MUT2 M62L;157592;humsavar:VAR_047685;rs2308911;

MUT2 M62K;157593;humsavar:VAR_058836;rs2308912;

MUT2 M62Q;157599;humsavar:VAR_058850;rs36013091;

AA2 75H;Interacts with:180W,179D,178G

AA2 76L;Interacts with:180W

AA2 79F;Interacts with:180W

//

MESH1 mesh:D001172

NAME1 Arthritis, Rheumatoid

CLUSTER1 clust1

MESH2 mesh:D001249

NAME2 Asthma

CLUSTER2 clust1

AC1 P01375

PFAM1 PF00229

PDB1 3ALQ:A

AC2 P20333

PFAM2 PF00020

PDB2 3ALQ:R

INT semi-edgetic

AA1 107R;Interacts with:93C,92E

MUT1 R107P;PMID:1331108;

MUT1 R107K;PMID:8253759;

MUT1 R107H;PMID:2217144,PMID:1331108;

AA1 108R;Interacts with:95S,93C,81S,92E,80D,94L,82T,79E

MUT1 R108V;PMID;

MUT1 R108W;963560;clinvar:27425, phenotype=TNF RECEPTOR BINDING, ALTERED;

MUT1 R108E;PMID:1715560;

MUT1 R108Q;PMID:2217144;

AA1 109A;Interacts with:94L,92E,90V,93C

MUT1 A109T;PMID:2217144,PMID:1715560,PMID:8253759;

AA1 143Q;Interacts with:102S,101S,103D

MUT1 Q143E;PMID:MEDLINE;

AA1 161V;Interacts with:89W

AA1 162S;Interacts with:89W,88N,90V

MUT1 S162L;PMID:2009860;

MUT1 S162F;963563;

AA1 163Y;Interacts with:85Q,94L,90V,84T,86L,88N,87W

MUT1 Y163H;PMID:2217144,PMID:1715560,PMID:1331108;

AA1 164Q;Interacts with:86L,87W

AA1 165T;Interacts with:86L

AA1 167V;Interacts with:94L

MUT1 V167I;PMID:2217144,PMID:1715560;

MUT1 V167A;1026230;

MUT1 V167D;1026231;

AA1 203E;Interacts with:89W

AA1 220F;Interacts with:98S

MUT1 F220Y;PMID:2217144,PMID:8253759;

AA1 221A;Interacts with:98S,97G

AA1 222E;Interacts with:98S

MUT1 E222K;963564;

AA2 101S;Interacts with:143Q

AA2 102S;Interacts with:143Q

MUT2 S102P;963767;

AA2 103D;Interacts with:143Q

AA2 79E;Interacts with:108R

AA2 80D;Interacts with:108R

AA2 81S;Interacts with:108R

AA2 82T;Interacts with:108R

AA2 84T;Interacts with:163Y

AA2 85Q;Interacts with:163Y

AA2 86L;Interacts with:165T,164Q,163Y

AA2 87W;Interacts with:163Y,164Q

AA2 88N;Interacts with:162S,163Y

AA2 89W;Interacts with:161V,162S,203E

AA2 90V;Interacts with:109A,162S,163Y

AA2 92E;Interacts with:108R,109A,107R

AA2 93C;Interacts with:108R,107R,109A

AA2 94L;Interacts with:109A,108R,163Y,167V

AA2 95S;Interacts with:108R

AA2 97G;Interacts with:221A

AA2 98S;Interacts with:221A,220F,222E

MUT2 S98P;963766;

//

MESH1 mesh:D001172

NAME1 Arthritis, Rheumatoid

CLUSTER1 clust1

MESH2 mesh:D001249

NAME2 Asthma

CLUSTER2 clust1

AC1 P01903

PFAM1 PF07654

PDB1 1A6A:A

AC2 P01912

PFAM2 PF07654

PDB2 1A6A:B

INT semi-edgetic

AA1 118T;Interacts with:179N,185Q

AA1 119N;Interacts with:185Q,179N,183T,181D,150G

AA1 120S;Interacts with:149S,185Q

AA1 121P;Interacts with:147S

MUT1 P121S;PMID:8294865,PMID:9590215;

AA1 131I;Interacts with:179N

AA1 173F;Interacts with:180G,178H,179N

AA1 175Y;Interacts with:180G,179N,181D

AA2 147S;Interacts with:121P

AA2 149S;Interacts with:120S

AA2 150G;Interacts with:119N

AA2 178H;Interacts with:173F

AA2 179N;Interacts with:119N,131I,118T,173F,175Y

AA2 180G;Interacts with:175Y,173F

AA2 181D;Interacts with:119N,175Y

AA2 183T;Interacts with:119N

AA2 185Q;Interacts with:119N,120S,118T

//

MESH1 mesh:D001172

NAME1 Arthritis, Rheumatoid

CLUSTER1 clust1

MESH2 mesh:D001249

NAME2 Asthma

CLUSTER2 clust1

AC1 P01903

PFAM1 PF00993

PDB1 1A6A:A

AC2 P01912

PFAM2 PF00969

PDB2 1A6A:B

INT semi-edgetic

AA1 101R;Interacts with:86D,82L

AA1 102S;Interacts with:61H,82L

AA1 105T;Interacts with:61H,62N

AA1 106P;Interacts with:62N,63Q

AA1 107I;Interacts with:62N,63Q

AA1 108T;Interacts with:63Q

AA1 30H;Interacts with:112Y,45H,46F,47F

AA1 31V;Interacts with:45H,44C,46F

AA1 32I;Interacts with:111N,45H,44C,112Y,46F,115V,43E

MUT1 I32S;PMID:7528190,PMID:7869051,PMID:7636246;

AA1 33I;Interacts with:43E,42S

MUT1 I33T;856205;

AA1 34Q;Interacts with:42S,107Y

AA1 35A;Interacts with:42S

AA1 49F;Interacts with:107Y

MUT1 F49H;PMID:7528190,PMID:7869051;

AA1 56I;Interacts with:115V

AA1 77A;Interacts with:114V

AA1 95L;Interacts with:61H

AA1 98M;Interacts with:61H,82L

MUT1 M98I;COSMIC:71078;

AA1 99T;Interacts with:61H

AA2 107Y;Interacts with:49F,34Q

AA2 111N;Interacts with:32I

AA2 112Y;Interacts with:30H,32I

AA2 114V;Interacts with:77A

AA2 115V;Interacts with:56I,32I

MUT2 V115G;PMID:7805754;

AA2 42S;Interacts with:33I,34Q,35A

MUT2 S42C;dbSNP:rs17424090;

MUT2 S42A;PMID:7805754;

AA2 43E;Interacts with:33I,32I

AA2 44C;Interacts with:31V,32I

AA2 45H;Interacts with:31V,30H,32I

AA2 46F;Interacts with:30H,31V,32I

AA2 47F;Interacts with:30H

AA2 61H;Interacts with:102S,105T,99T,95L,98M

AA2 62N;Interacts with:107I,106P,105T

AA2 63Q;Interacts with:108T,107I,106P

AA2 82L;Interacts with:102S,101R,98M

AA2 86D;Interacts with:101R

MUT2 D86V;PMID:7805754;

//

MESH1 mesh:D001172

NAME1 Arthritis, Rheumatoid

CLUSTER1 clust1

MESH2 mesh:D001249

NAME2 Asthma

CLUSTER2 clust1

AC1 P01920

PFAM1 PF07654

PDB1 1JK8:B

AC2 P01909

PFAM2 PF00993

PDB2 1JK8:A

INT semi-edgetic

AA1 181R;Interacts with:54G,55D,53D,52F

MUT1 R181G;dbSNP:rs9273967;

MUT1 R181S;dbSNP:rs9273966;

AA1 183G;Interacts with:56E,55D,71L

MUT1 G183A;dbSNP:rs9273964;

AA1 184D;Interacts with:55D,71L

MUT1 D184E;dbSNP:rs9273963;

AA1 185W;Interacts with:52F,74L,56E,71L,70C,57Q,55D

MUT1 W185L;dbSNP:rs9273961;

MUT1 W185R;dbSNP:rs9273962;

AA1 186T;Interacts with:55D

AA2 52F;Interacts with:185W,181R

AA2 53D;Interacts with:181R

AA2 54G;Interacts with:181R

MUT2 G54E;dbSNP:rs112029808;

AA2 55D;Interacts with:184D,181R,183G,186T,185W

AA2 56E;Interacts with:183G,185W

AA2 57Q;Interacts with:185W

MUT2 Q57E;856046;dbSNP:rs10093;humsavar:VAR_014604;rs10093;dbSNP:rs10093;

AA2 70C;Interacts with:185W

MUT2 C70W;dbSNP:rs1142326;

MUT2 C70Q;856052;dbSNP:rs3207983;humsavar:VAR_060500;

MUT2 C70R;856053;humsavar:VAR_060501;

MUT2 C70Y;856054;humsavar:VAR_033404;rs3207983;

MUT2 C70K;856051;humsavar:VAR_060499;

AA2 71L;Interacts with:185W,184D,183G

MUT2 L71W;856055;humsavar:VAR_060502;rs1142328;

AA2 74L;Interacts with:185W

MUT2 L74F;856059;humsavar:VAR_060504;rs9272698;

//

MESH1 mesh:D001172

NAME1 Arthritis, Rheumatoid

CLUSTER1 clust1

MESH2 mesh:D001249

NAME2 Asthma

CLUSTER2 clust1

AC1 Q969J5

PFAM1 PF09294

PDB1 3G9V:C

AC2 Q9GZX6

PFAM2 PF14565

PDB2 3G9V:C

INT semi-edgetic

AA1 200E;Interacts with:175R

AA1 246P;Interacts with:50P

AA1 247M;Interacts with:54N,50P,51Y,53T,48Q

AA1 248L;Interacts with:54N,53T,50P

AA1 249D;Interacts with:54N,50P

AA1 250R;Interacts with:54N

AA2 175R;Interacts with:200E

AA2 48Q;Interacts with:247M

AA2 50P;Interacts with:247M,249D,248L,246P

AA2 51Y;Interacts with:247M

AA2 53T;Interacts with:247M,248L

AA2 54N;Interacts with:247M,248L,250R,249D

//

MESH1 mesh:D001172

NAME1 Arthritis, Rheumatoid

CLUSTER1 clust1

MESH2 mesh:D001249

NAME2 Asthma

CLUSTER2 clust1

AC1 Q969J5

PFAM1 PF01108

PDB1 3G9V:A

AC2 Q9GZX6

PFAM2 PF14565

PDB2 3G9V:A

INT semi-edgetic

AA1 100G;Interacts with:70T,71D,72V,73R

AA1 101Q;Interacts with:71D,72V,70T

AA1 102R;Interacts with:71D

AA1 103Q;Interacts with:71D

AA1 104W;Interacts with:70T,71D

AA1 128Q;Interacts with:175R,73R,176N

AA1 129E;Interacts with:73R

AA1 130P;Interacts with:73R

AA1 132Y;Interacts with:70T

AA1 65K;Interacts with:70T,71D

AA1 66I;Interacts with:73R

AA1 99Y;Interacts with:70T,169L,72V,69N,73R

AA2 169L;Interacts with:99Y

AA2 175R;Interacts with:128Q

AA2 176N;Interacts with:128Q

AA2 69N;Interacts with:99Y

AA2 70T;Interacts with:104W,99Y,132Y,100G,65K,101Q

AA2 71D;Interacts with:101Q,103Q,100G,104W,65K,102R

AA2 72V;Interacts with:99Y,100G,101Q

AA2 73R;Interacts with:129E,130P,128Q,100G,99Y,66I

MUT2 R73H;dbSNP:rs149366319;

//

MESH1 mesh:D001172

NAME1 Arthritis, Rheumatoid

CLUSTER1 clust1

MESH2 mesh:D001249

NAME2 Asthma

CLUSTER2 clust1

AC1 P01903

PFAM1 PF07654

PDB1 1BX2:A

AC2 P01911

PFAM2 PF07654

PDB2 1BX2:B

INT semi-edgetic

AA1 118T;Interacts with:185Q

AA1 119N;Interacts with:185Q,179N,181D,183T,150G

AA1 120S;Interacts with:149S,185Q

AA1 121P;Interacts with:147S,149S

MUT1 P121S;PMID:8294865,PMID:9590215;

AA1 131I;Interacts with:179N

AA1 133F;Interacts with:177I,179N,178Q

AA1 160T;Interacts with:180G

AA1 171R;Interacts with:178Q

AA1 173F;Interacts with:180G,178Q,179N

AA1 175Y;Interacts with:180G,179N,181D

AA2 147S;Interacts with:121P

AA2 149S;Interacts with:121P,120S

MUT2 S149N;dbSNP:rs74626234,dbSNP:rs116633902;

AA2 150G;Interacts with:119N

AA2 177I;Interacts with:133F

AA2 178Q;Interacts with:173F,171R,133F

MUT2 Q178H;dbSNP:rs77637983,dbSNP:rs113627837;

AA2 179N;Interacts with:119N,131I,133F,173F,175Y

AA2 180G;Interacts with:175Y,173F,160T

MUT2 G180E;dbSNP:rs1059353;

AA2 181D;Interacts with:119N,175Y

AA2 183T;Interacts with:119N

AA2 185Q;Interacts with:119N,118T,120S

//

MESH1 mesh:D001172

NAME1 Arthritis, Rheumatoid

CLUSTER1 clust1

MESH2 mesh:D001249

NAME2 Asthma

CLUSTER2 clust1

AC1 P01903

PFAM1 PF07654

PDB1 1BX2:D

AC2 P01911

PFAM2 PF00969

PDB2 1BX2:E

INT semi-edgetic

AA1 138T;Interacts with:63Q

AA1 166E;Interacts with:58R

AA1 167D;Interacts with:63Q

AA1 168H;Interacts with:63Q,65E,60F

AA1 169L;Interacts with:63Q

AA2 58R;Interacts with:166E

MUT2 R58S;dbSNP:rs17878437;

AA2 60F;Interacts with:168H

MUT2 F60V;dbSNP:rs17882300;

MUT2 F60I;dbSNP:rs1059346,dbSNP:rs17882300;

AA2 63Q;Interacts with:168H,167D,138T,169L

MUT2 Q63R;dbSNP:rs17879476;

AA2 65E;Interacts with:168H

//

MESH1 mesh:D001172

NAME1 Arthritis, Rheumatoid

CLUSTER1 clust1

MESH2 mesh:D001249

NAME2 Asthma

CLUSTER2 clust1

AC1 P01903

PFAM1 PF00993

PDB1 1BX2:A

AC2 P01911

PFAM2 PF00969

PDB2 1BX2:B

INT semi-edgetic

AA1 100K;Interacts with:61Y

MUT1 K100E;PMID:7869051,PMID:7722459,PMID:7636246;

AA1 101R;Interacts with:86D,82L

AA1 102S;Interacts with:61Y,82L

AA1 104Y;Interacts with:61Y

AA1 105T;Interacts with:61Y,62N

AA1 106P;Interacts with:62N,61Y

AA1 107I;Interacts with:62N

AA1 108T;Interacts with:63Q,62N

AA1 29E;Interacts with:49G,46F,48N,47F,45H

AA1 30H;Interacts with:112Y,45H,46F,47F

AA1 31V;Interacts with:45H,44C,46F

AA1 32I;Interacts with:111N,45H,44C,112Y,46F,115V,43E

MUT1 I32S;PMID:7528190,PMID:7869051,PMID:7636246;

AA1 33I;Interacts with:43E,42R,44C

MUT1 I33T;856205;

AA1 34Q;Interacts with:107Y,42R,43E

AA1 35A;Interacts with:42R

AA1 36E;Interacts with:42R

AA1 49F;Interacts with:107Y

MUT1 F49H;PMID:7528190,PMID:7869051;

AA1 51F;Interacts with:115V

AA1 56I;Interacts with:115V

AA1 77A;Interacts with:114V

AA1 87N;Interacts with:42R

AA1 91D;Interacts with:42R

MUT1 D91L;PMID:7869051;

MUT1 D91R;PMID:7869051,PMID:7636246;

AA1 95L;Interacts with:61Y

AA1 98M;Interacts with:82L,61Y

MUT1 M98I;COSMIC:71078;

AA1 99T;Interacts with:61Y

AA2 107Y;Interacts with:34Q,49F

MUT2 Y107H;dbSNP:rs17884043;

MUT2 Y107D;dbSNP:rs17884043;

MUT2 Y107F;dbSNP:rs17879620;

AA2 111N;Interacts with:32I

AA2 112Y;Interacts with:30H,32I

AA2 114V;Interacts with:77A

MUT2 V114A;dbSNP:rs17424145;

AA2 115V;Interacts with:56I,51F,32I

MUT2 V115E;dbSNP:rs17885482;

MUT2 V115M;dbSNP:rs61759935;

MUT2 V115G;158129;humsavar:VAR_038164;rs17885482;

AA2 42R;Interacts with:35A,34Q,36E,33I,87N,91D

MUT2 R42T;dbSNP:rs1136759;

MUT2 R42K;dbSNP:rs17424104,dbSNP:rs1136759;

MUT2 R42G;dbSNP:rs1136758;

MUT2 R42S;dbSNP:rs17424111,dbSNP:rs9269951;

MUT2 R42W;dbSNP:rs1136758;

MUT2 R42M;dbSNP:rs17424104,dbSNP:rs1136759;

AA2 43E;Interacts with:33I,32I,34Q

MUT2 E43K;dbSNP:rs17405212,dbSNP:rs17882014;

AA2 44C;Interacts with:31V,32I,33I

AA2 45H;Interacts with:31V,30H,32I,29E

MUT2 H45Q;dbSNP:rs17879981;

MUT2 H45Y;dbSNP:rs17879702;

AA2 46F;Interacts with:30H,31V,32I,29E

AA2 47F;Interacts with:29E,30H

MUT2 F47L;dbSNP:rs114435757;

AA2 48N;Interacts with:29E

AA2 49G;Interacts with:29E

MUT2 G49A;dbSNP:rs17879469;

MUT2 G49R;dbSNP:rs61759931;

AA2 61Y;Interacts with:102S,105T,95L,106P,98M,99T,104Y,100K

MUT2 Y61D;dbSNP:rs1064664;

MUT2 Y61H;dbSNP:rs1064664;

AA2 62N;Interacts with:107I,106P,105T,108T

MUT2 N62H;dbSNP:rs17879995;

MUT2 N62K;dbSNP:rs17879242;

AA2 63Q;Interacts with:108T

MUT2 Q63R;dbSNP:rs17879476;

AA2 82L;Interacts with:102S,101R,98M

MUT2 L82M;dbSNP:rs72558166;

AA2 86D;Interacts with:101R

MUT2 D86G;dbSNP:rs17885129;

MUT2 D86V;dbSNP:rs17885129;

MUT2 D86A;dbSNP:rs17885129;

MUT2 D86E;dbSNP:rs17853228;

MUT2 D86N;dbSNP:rs17880292;

//

MESH1 mesh:D001172

NAME1 Arthritis, Rheumatoid

CLUSTER1 clust1

MESH2 mesh:D001249

NAME2 Asthma

CLUSTER2 clust1

AC1 Q8N6P7

PFAM1 PF09294

PDB1 3DGC:R

AC2 Q9GZX6

PFAM2 PF14565

PDB2 3DGC:R

INT semi-edgetic

AA1 133R;Interacts with:116Q,117E

AA1 134S;Interacts with:124R

AA1 136Q;Interacts with:124R

AA1 162D;Interacts with:175R,48Q,44K

AA1 175Y;Interacts with:51Y

AA1 179L;Interacts with:45S

AA1 180G;Interacts with:48Q

AA1 183Q;Interacts with:45S,43D,44K

AA1 185E;Interacts with:128R,124R,43D

AA1 186Y;Interacts with:128R

AA1 187E;Interacts with:124R,49Q,128R,45S

AA1 189F;Interacts with:46N,52I,49Q,121F,124R

AA1 190G;Interacts with:51Y,52I,49Q

AA1 191L;Interacts with:117E

AA1 206P;Interacts with:48Q

AA1 207T;Interacts with:172M,175R,48Q,44K

AA1 208W;Interacts with:57F,172M

AA2 116Q;Interacts with:133R

AA2 117E;Interacts with:191L,133R

AA2 121F;Interacts with:189F

AA2 124R;Interacts with:187E,185E,189F,134S,136Q

AA2 128R;Interacts with:187E,185E,186Y

AA2 172M;Interacts with:207T,208W

MUT2 M172V;dbSNP:rs147812317;

AA2 175R;Interacts with:162D,207T

AA2 43D;Interacts with:183Q,185E

AA2 44K;Interacts with:162D,207T,183Q

AA2 45S;Interacts with:179L,183Q,187E

AA2 46N;Interacts with:189F

AA2 48Q;Interacts with:206P,162D,207T,180G

AA2 49Q;Interacts with:187E,189F,190G

AA2 51Y;Interacts with:190G,175Y

AA2 52I;Interacts with:189F,190G

AA2 57F;Interacts with:208W

//

MESH1 mesh:D001172

NAME1 Arthritis, Rheumatoid

CLUSTER1 clust1

MESH2 mesh:D001249

NAME2 Asthma

CLUSTER2 clust1

AC1 Q8N6P7

PFAM1 PF01108

PDB1 3DGC:R

AC2 Q9GZX6

PFAM2 PF14565

PDB2 3DGC:R

INT semi-edgetic

AA1 58K;Interacts with:71D,70T

MUT1 K58A;882419;

AA1 60Y;Interacts with:72V,70T,169L,73R,69N,74L

MUT1 Y60A;1368880;

MUT1 Y60R;1368881;

AA1 61G;Interacts with:73R,72V,70T,71D

AA1 62E;Interacts with:72V,70T,71D

AA1 63R;Interacts with:71D

AA1 64D;Interacts with:71D

AA1 65W;Interacts with:70T

AA1 89T;Interacts with:77E,73R

AA1 90E;Interacts with:73R

AA1 91L;Interacts with:73R,169L

MUT1 L91F;dbSNP:rs144035143;

AA1 93Y;Interacts with:70T

AA2 169L;Interacts with:60Y,91L

AA2 69N;Interacts with:60Y

AA2 70T;Interacts with:58K,61G,60Y,62E,93Y,65W

AA2 71D;Interacts with:58K,61G,63R,64D,62E

AA2 72V;Interacts with:62E,60Y,61G

AA2 73R;Interacts with:61G,90E,89T,60Y,91L

MUT2 R73H;dbSNP:rs149366319;

AA2 74L;Interacts with:60Y

AA2 77E;Interacts with:89T

//

MESH1 mesh:D001172

NAME1 Arthritis, Rheumatoid

CLUSTER1 clust1

MESH2 mesh:D001249

NAME2 Asthma

CLUSTER2 clust1

AC1 P01903

PFAM1 PF07654

PDB1 4AH2:A

AC2 P04233

PFAM2 PF07654

PDB2 4AH2:B

INT semi-edgetic

AA1 118T;Interacts with:252C

AA1 119N;Interacts with:252C,246S,250C,248G,222A

AA1 120S;Interacts with:252C

AA1 121P;Interacts with:219H,205A,221P

MUT1 P121S;PMID:8294865,PMID:9590215;

AA1 131I;Interacts with:246S

AA1 171R;Interacts with:245G

AA1 173F;Interacts with:247I,245G,246S

AA1 175Y;Interacts with:247I,246S,248G

AA2 205A;Interacts with:121P

AA2 219H;Interacts with:121P

AA2 221P;Interacts with:121P

AA2 222A;Interacts with:119N

AA2 245G;Interacts with:173F,171R

AA2 246S;Interacts with:119N,131I,173F,175Y

AA2 247I;Interacts with:175Y,173F

AA2 248G;Interacts with:119N,175Y

AA2 250C;Interacts with:119N

AA2 252C;Interacts with:119N,120S,118T

//

MESH1 mesh:D001172

NAME1 Arthritis, Rheumatoid

CLUSTER1 clust1

MESH2 mesh:D001249

NAME2 Asthma

CLUSTER2 clust1

AC1 P01903

PFAM1 PF07654

PDB1 4AH2:A

AC2 P04233

PFAM2 PF00969

PDB2 4AH2:B

INT semi-edgetic

AA1 166E;Interacts with:69Y

AA1 167D;Interacts with:74Q

AA1 168H;Interacts with:74Q,91E,71L

AA2 69Y;Interacts with:166E

AA2 71L;Interacts with:168H

AA2 74Q;Interacts with:168H,167D

AA2 91E;Interacts with:168H

//

MESH1 mesh:D001172

NAME1 Arthritis, Rheumatoid

CLUSTER1 clust1

MESH2 mesh:D001249

NAME2 Asthma

CLUSTER2 clust1

AC1 P01903

PFAM1 PF00993

PDB1 4AH2:A

AC2 P04233

PFAM2 PF00969

PDB2 4AH2:B

INT semi-edgetic

AA1 101R;Interacts with:125Q,113L

AA1 102S;Interacts with:113L

AA1 104Y;Interacts with:72Y

AA1 105T;Interacts with:72Y,73Q

AA1 106P;Interacts with:73Q,72Y

AA1 107I;Interacts with:73Q

AA1 29E;Interacts with:50Y,49L,45S

AA1 30H;Interacts with:178W,45S,49L,50Y

AA1 31V;Interacts with:45S,44C,49L

AA1 32I;Interacts with:177D,45S,44C,178W,49L,43K

MUT1 I32S;PMID:7528190,PMID:7869051,PMID:7636246;

AA1 33I;Interacts with:43K,32M

MUT1 I33T;856205;

AA1 34Q;Interacts with:155Y,32M

AA1 35A;Interacts with:32M

AA1 49F;Interacts with:155Y

MUT1 F49H;PMID:7528190,PMID:7869051;

AA1 77A;Interacts with:180V

AA1 95L;Interacts with:72Y

AA1 98M;Interacts with:113L,72Y

MUT1 M98I;COSMIC:71078;

AA1 99T;Interacts with:72Y

AA2 113L;Interacts with:102S,101R,98M

AA2 125Q;Interacts with:101R

AA2 155Y;Interacts with:49F,34Q

AA2 177D;Interacts with:32I

AA2 178W;Interacts with:30H,32I

AA2 180V;Interacts with:77A

AA2 32M;Interacts with:35A,33I,34Q

AA2 43K;Interacts with:33I,32I

AA2 44C;Interacts with:31V,32I

AA2 45S;Interacts with:31V,30H,32I,29E

AA2 49L;Interacts with:30H,31V,32I,29E

AA2 50Y;Interacts with:29E,30H

AA2 72Y;Interacts with:105T,95L,106P,98M,99T,104Y

AA2 73Q;Interacts with:107I,106P,105T

//

MESH1 mesh:D001172

NAME1 Arthritis, Rheumatoid

CLUSTER1 clust1

MESH2 mesh:D001249

NAME2 Asthma

CLUSTER2 clust1

AC1 P22301

PFAM1 PF00726

PDB1 1J7V:L

AC2 Q13651

PFAM2 PF09294

PDB2 1J7V:R

INT semi-edgetic

AA1 38P;Interacts with:212R,211S,164F,210A

AA1 39N;Interacts with:210A

AA1 41L;Interacts with:211S

AA1 42R;Interacts with:211S,214N,208S,212R,166E,210A,213S

AA1 43D;Interacts with:211S

AA1 45R;Interacts with:211S

AA1 46D;Interacts with:213S

AA2 164F;Interacts with:38P

AA2 166E;Interacts with:42R

AA2 208S;Interacts with:42R

AA2 210A;Interacts with:39N,42R,38P

AA2 211S;Interacts with:42R,38P,41L,45R,43D

AA2 212R;Interacts with:38P,42R

AA2 213S;Interacts with:46D,42R

AA2 214N;Interacts with:42R

//

MESH1 mesh:D001172

NAME1 Arthritis, Rheumatoid

CLUSTER1 clust1

MESH2 mesh:D001249

NAME2 Asthma

CLUSTER2 clust1

AC1 P22301

PFAM1 PF00726

PDB1 1J7V:L

AC2 Q13651

PFAM2 PF01108

PDB2 1J7V:R

INT semi-edgetic

AA1 56Q;Interacts with:97R

AA1 59D;Interacts with:97R

AA1 60Q;Interacts with:97R

MUT1 Q60E;PMID:10637267;

AA1 61L;Interacts with:97R,64Y

AA1 62D;Interacts with:69W,66I,65G,67E,97R,64Y

AA1 63N;Interacts with:67E,65G,64Y

AA1 64L;Interacts with:64Y,67E,65G

AA1 65L;Interacts with:64Y

AA1 66L;Interacts with:94N

AA2 64Y;Interacts with:65L,64L,61L,62D,63N

AA2 65G;Interacts with:62D,63N,64L

AA2 66I;Interacts with:62D

AA2 67E;Interacts with:63N,62D,64L

AA2 69W;Interacts with:62D

AA2 94N;Interacts with:66L

AA2 97R;Interacts with:60Q,61L,59D,62D,56Q

//

MESH1 mesh:D001172

NAME1 Arthritis, Rheumatoid

CLUSTER1 clust1

MESH2 mesh:D001249

NAME2 Asthma

CLUSTER2 clust1

AC1 P01903

PFAM1 PF07654

PDB1 1ZGL:A

AC2 Q30154

PFAM2 PF07654

PDB2 1ZGL:B

INT semi-edgetic

AA1 118T;Interacts with:185Q,179N

AA1 119N;Interacts with:185Q,179N,149N,150G,181D

AA1 120S;Interacts with:149N,185Q

AA1 121P;Interacts with:147S,149N,131Y

MUT1 P121S;PMID:8294865,PMID:9590215;

AA1 131I;Interacts with:179N

AA1 160T;Interacts with:180G

AA1 171R;Interacts with:178Q

AA1 173F;Interacts with:180G,178Q,179N

AA1 175Y;Interacts with:180G,181D,179N

AA2 131Y;Interacts with:121P

AA2 147S;Interacts with:121P

MUT2 S147C;dbSNP:rs184544272;

AA2 149N;Interacts with:119N,121P,120S

MUT2 N149S;dbSNP:rs114293611;

AA2 150G;Interacts with:119N

AA2 178Q;Interacts with:173F,171R

MUT2 Q178H;dbSNP:rs139485758;

AA2 179N;Interacts with:119N,131I,173F,118T,175Y

AA2 180G;Interacts with:175Y,173F,160T

MUT2 G180E;dbSNP:rs1059353;

AA2 181D;Interacts with:175Y,119N

AA2 185Q;Interacts with:119N,118T,120S

//

MESH1 mesh:D001172

NAME1 Arthritis, Rheumatoid

CLUSTER1 clust1

MESH2 mesh:D001249

NAME2 Asthma

CLUSTER2 clust1

AC1 P01903

PFAM1 PF07654

PDB1 1HQR:A

AC2 Q30154

PFAM2 PF00969

PDB2 1HQR:B

INT semi-edgetic

AA1 166E;Interacts with:58R

AA1 167D;Interacts with:63Q

AA1 168H;Interacts with:60I,64E,65E,63Q

AA2 58R;Interacts with:166E

AA2 60I;Interacts with:168H

MUT2 I60F;dbSNP:rs1059346,dbSNP:rs140781802;

AA2 63Q;Interacts with:168H,167D

AA2 64E;Interacts with:168H

AA2 65E;Interacts with:168H

//

MESH1 mesh:D001172

NAME1 Arthritis, Rheumatoid

CLUSTER1 clust1

MESH2 mesh:D001249

NAME2 Asthma

CLUSTER2 clust1

AC1 P01903

PFAM1 PF00993

PDB1 1ZGL:A

AC2 Q30154

PFAM2 PF00969

PDB2 1ZGL:B

INT semi-edgetic

AA1 100K;Interacts with:61Y

MUT1 K100E;PMID:7869051,PMID:7722459,PMID:7636246;

AA1 101R;Interacts with:86D,82L,66D,85P

AA1 102S;Interacts with:61Y,82L,62N

AA1 104Y;Interacts with:61Y

AA1 105T;Interacts with:61Y,62N

AA1 106P;Interacts with:62N,61Y

AA1 107I;Interacts with:62N

AA1 108T;Interacts with:63Q

AA1 29E;Interacts with:46F,49G,47F,48N

AA1 30H;Interacts with:45H,46F,112Y,47F

AA1 31V;Interacts with:45H,44C,43E,46F

AA1 32I;Interacts with:45H,44C,46F,43E,111N,112Y

MUT1 I32S;PMID:7528190,PMID:7869051,PMID:7636246;

AA1 33I;Interacts with:43E,42Y,44C

MUT1 I33T;856205;

AA1 34Q;Interacts with:107Y,42Y,43E,111N

AA1 35A;Interacts with:42Y

AA1 49F;Interacts with:107Y

MUT1 F49H;PMID:7528190,PMID:7869051;

AA1 77A;Interacts with:114V

AA1 87N;Interacts with:42Y

AA1 94N;Interacts with:59D,86D

AA1 95L;Interacts with:61Y

AA1 98M;Interacts with:82L,61Y

MUT1 M98I;COSMIC:71078;

AA1 99T;Interacts with:61Y

AA2 107Y;Interacts with:49F,34Q

MUT2 Y107V;856275;humsavar:VAR_060976;

MUT2 Y107S;dbSNP:rs1064677;

MUT2 Y107D;dbSNP:rs1064595;

MUT2 Y107F;dbSNP:rs1064677;

AA2 111N;Interacts with:34Q,32I

AA2 112Y;Interacts with:30H,32I

AA2 114V;Interacts with:77A

MUT2 V114A;158255;humsavar:VAR_060977;rs1136778;

AA2 42Y;Interacts with:33I,34Q,87N,35A

MUT2 Y42F;dbSNP:rs17424104,dbSNP:rs1136760,dbSNP:rs79937656;

MUT2 Y42S;dbSNP:rs79937656;

MUT2 Y42H;dbSNP:rs17433877,dbSNP:rs1136757;

AA2 43E;Interacts with:33I,31V,32I,34Q

MUT2 E43K;dbSNP:rs17405212,dbSNP:rs149574893;

AA2 44C;Interacts with:31V,32I,33I

AA2 45H;Interacts with:31V,30H,32I

AA2 46F;Interacts with:30H,31V,32I,29E

AA2 47F;Interacts with:29E,30H

AA2 48N;Interacts with:29E

AA2 49G;Interacts with:29E

AA2 59D;Interacts with:94N

MUT2 D59Y;dbSNP:rs138849995;

MUT2 D59H;856268;humsavar:VAR_060957;rs707955;

MUT2 D59G;158267;humsavar:VAR_060956;rs41546317;

AA2 61Y;Interacts with:102S,105T,95L,106P,100K,98M,99T,104Y

MUT2 Y61H;dbSNP:rs137863146;

AA2 62N;Interacts with:107I,106P,105T,102S

MUT2 N62H;158238;humsavar:VAR_050358;rs1059576;

AA2 63Q;Interacts with:108T

AA2 66D;Interacts with:101R

MUT2 D66N;158286;humsavar:VAR_060958;rs707956;

MUT2 D66Y;856269;humsavar:VAR_060959;rs77853982;

AA2 82L;Interacts with:102S,101R,98M

AA2 85P;Interacts with:101R

AA2 86D;Interacts with:101R,94N

MUT2 D86V;dbSNP:rs144532965;

MUT2 D86N;dbSNP:rs145586726;

//

MESH1 mesh:D001172

NAME1 Arthritis, Rheumatoid

CLUSTER1 clust1

MESH2 mesh:D002446

NAME2 Celiac Disease

CLUSTER2 clust7

AC1 P28068

PFAM1 PF07654

PDB1 4I0P:B

AC2 Q6ICR9

PFAM2 PF00993

PDB2 4I0P:A

INT non-edgetic

AA1 141Y;Interacts with:63Y

AA1 168P;Interacts with:65E

AA1 169N;Interacts with:66D,81R

AA1 170G;Interacts with:66D,81R

AA1 171D;Interacts with:66D,82L

AA1 172W;Interacts with:66D,68L,63Y,85F,67Q,82L

AA1 173T;Interacts with:66D

AA1 174Y;Interacts with:65E,66D

AA2 63Y;Interacts with:172W,141Y

AA2 65E;Interacts with:174Y,168P

AA2 66D;Interacts with:172W,173T,174Y,169N,170G,171D

AA2 67Q;Interacts with:172W

AA2 68L;Interacts with:172W

AA2 81R;Interacts with:170G,169N

AA2 82L;Interacts with:171D,172W

AA2 85F;Interacts with:172W

//

MESH1 mesh:D001172

NAME1 Arthritis, Rheumatoid

CLUSTER1 clust1

MESH2 mesh:D002446

NAME2 Celiac Disease

CLUSTER2 clust7

AC1 P04440

PFAM1 PF07654

PDB1 3LQZ:B

AC2 P20036

PFAM2 PF00993

PDB2 3LQZ:A

INT non-edgetic

AA1 176R;Interacts with:59E,58D,60D

AA1 178G;Interacts with:60D,75H

AA1 179D;Interacts with:60D,75H

AA1 180W;Interacts with:62M,60D,61E,75H,57F,79F,76L

AA1 181T;Interacts with:60D

AA2 57F;Interacts with:180W

AA2 58D;Interacts with:176R

AA2 59E;Interacts with:176R

MUT2 E59D;157591;humsavar:VAR_058835;rs2308910;

AA2 60D;Interacts with:180W,179D,178G,176R,181T

AA2 61E;Interacts with:180W

AA2 62M;Interacts with:180W

MUT2 M62L;157592;humsavar:VAR_047685;rs2308911;

MUT2 M62K;157593;humsavar:VAR_058836;rs2308912;

MUT2 M62Q;157599;humsavar:VAR_058850;rs36013091;

AA2 75H;Interacts with:180W,179D,178G

AA2 76L;Interacts with:180W

AA2 79F;Interacts with:180W

//

MESH1 mesh:D001172

NAME1 Arthritis, Rheumatoid

CLUSTER1 clust1

MESH2 mesh:D002446

NAME2 Celiac Disease

CLUSTER2 clust7

AC1 P01903

PFAM1 PF07654

PDB1 1A6A:A

AC2 P01912

PFAM2 PF07654

PDB2 1A6A:B

INT semi-edgetic

AA1 118T;Interacts with:179N,185Q

AA1 119N;Interacts with:185Q,179N,183T,181D,150G

AA1 120S;Interacts with:149S,185Q

AA1 121P;Interacts with:147S

MUT1 P121S;PMID:8294865,PMID:9590215;

AA1 131I;Interacts with:179N

AA1 173F;Interacts with:180G,178H,179N

AA1 175Y;Interacts with:180G,179N,181D

AA2 147S;Interacts with:121P

AA2 149S;Interacts with:120S

AA2 150G;Interacts with:119N

AA2 178H;Interacts with:173F

AA2 179N;Interacts with:119N,131I,118T,173F,175Y

AA2 180G;Interacts with:175Y,173F

AA2 181D;Interacts with:119N,175Y

AA2 183T;Interacts with:119N

AA2 185Q;Interacts with:119N,120S,118T

//

MESH1 mesh:D001172

NAME1 Arthritis, Rheumatoid

CLUSTER1 clust1

MESH2 mesh:D002446

NAME2 Celiac Disease

CLUSTER2 clust7

AC1 P01903

PFAM1 PF00993

PDB1 1A6A:A

AC2 P01912

PFAM2 PF00969

PDB2 1A6A:B

INT semi-edgetic

AA1 101R;Interacts with:86D,82L

AA1 102S;Interacts with:61H,82L

AA1 105T;Interacts with:61H,62N

AA1 106P;Interacts with:62N,63Q

AA1 107I;Interacts with:62N,63Q

AA1 108T;Interacts with:63Q

AA1 30H;Interacts with:112Y,45H,46F,47F

AA1 31V;Interacts with:45H,44C,46F

AA1 32I;Interacts with:111N,45H,44C,112Y,46F,115V,43E

MUT1 I32S;PMID:7528190,PMID:7869051,PMID:7636246;

AA1 33I;Interacts with:43E,42S

MUT1 I33T;856205;

AA1 34Q;Interacts with:42S,107Y

AA1 35A;Interacts with:42S

AA1 49F;Interacts with:107Y

MUT1 F49H;PMID:7528190,PMID:7869051;

AA1 56I;Interacts with:115V

AA1 77A;Interacts with:114V

AA1 95L;Interacts with:61H

AA1 98M;Interacts with:61H,82L

MUT1 M98I;COSMIC:71078;

AA1 99T;Interacts with:61H

AA2 107Y;Interacts with:49F,34Q

AA2 111N;Interacts with:32I

AA2 112Y;Interacts with:30H,32I

AA2 114V;Interacts with:77A

AA2 115V;Interacts with:56I,32I

MUT2 V115G;PMID:7805754;

AA2 42S;Interacts with:33I,34Q,35A

MUT2 S42C;dbSNP:rs17424090;

MUT2 S42A;PMID:7805754;

AA2 43E;Interacts with:33I,32I

AA2 44C;Interacts with:31V,32I

AA2 45H;Interacts with:31V,30H,32I

AA2 46F;Interacts with:30H,31V,32I

AA2 47F;Interacts with:30H

AA2 61H;Interacts with:102S,105T,99T,95L,98M

AA2 62N;Interacts with:107I,106P,105T

AA2 63Q;Interacts with:108T,107I,106P

AA2 82L;Interacts with:102S,101R,98M

AA2 86D;Interacts with:101R

MUT2 D86V;PMID:7805754;

//

MESH1 mesh:D001172

NAME1 Arthritis, Rheumatoid

CLUSTER1 clust1

MESH2 mesh:D002446

NAME2 Celiac Disease

CLUSTER2 clust7

AC1 P01920

PFAM1 PF07654

PDB1 1JK8:B

AC2 P01909

PFAM2 PF00993

PDB2 1JK8:A

INT semi-edgetic

AA1 181R;Interacts with:54G,55D,53D,52F

MUT1 R181G;dbSNP:rs9273967;

MUT1 R181S;dbSNP:rs9273966;

AA1 183G;Interacts with:56E,55D,71L

MUT1 G183A;dbSNP:rs9273964;

AA1 184D;Interacts with:55D,71L

MUT1 D184E;dbSNP:rs9273963;

AA1 185W;Interacts with:52F,74L,56E,71L,70C,57Q,55D

MUT1 W185L;dbSNP:rs9273961;

MUT1 W185R;dbSNP:rs9273962;

AA1 186T;Interacts with:55D

AA2 52F;Interacts with:185W,181R

AA2 53D;Interacts with:181R

AA2 54G;Interacts with:181R

MUT2 G54E;dbSNP:rs112029808;

AA2 55D;Interacts with:184D,181R,183G,186T,185W

AA2 56E;Interacts with:183G,185W

AA2 57Q;Interacts with:185W

MUT2 Q57E;856046;dbSNP:rs10093;humsavar:VAR_014604;rs10093;dbSNP:rs10093;

AA2 70C;Interacts with:185W

MUT2 C70W;dbSNP:rs1142326;

MUT2 C70Q;856052;dbSNP:rs3207983;humsavar:VAR_060500;

MUT2 C70R;856053;humsavar:VAR_060501;

MUT2 C70Y;856054;humsavar:VAR_033404;rs3207983;

MUT2 C70K;856051;humsavar:VAR_060499;

AA2 71L;Interacts with:185W,184D,183G

MUT2 L71W;856055;humsavar:VAR_060502;rs1142328;

AA2 74L;Interacts with:185W

MUT2 L74F;856059;humsavar:VAR_060504;rs9272698;

//

MESH1 mesh:D001172

NAME1 Arthritis, Rheumatoid

CLUSTER1 clust1

MESH2 mesh:D002446

NAME2 Celiac Disease

CLUSTER2 clust7

AC1 P01903

PFAM1 PF07654

PDB1 4AH2:A

AC2 P04233

PFAM2 PF07654

PDB2 4AH2:B

INT semi-edgetic

AA1 118T;Interacts with:252C

AA1 119N;Interacts with:252C,246S,250C,248G,222A

AA1 120S;Interacts with:252C

AA1 121P;Interacts with:219H,205A,221P

MUT1 P121S;PMID:8294865,PMID:9590215;

AA1 131I;Interacts with:246S

AA1 171R;Interacts with:245G

AA1 173F;Interacts with:247I,245G,246S

AA1 175Y;Interacts with:247I,246S,248G

AA2 205A;Interacts with:121P

AA2 219H;Interacts with:121P

AA2 221P;Interacts with:121P

AA2 222A;Interacts with:119N

AA2 245G;Interacts with:173F,171R

AA2 246S;Interacts with:119N,131I,173F,175Y

AA2 247I;Interacts with:175Y,173F

AA2 248G;Interacts with:119N,175Y

AA2 250C;Interacts with:119N

AA2 252C;Interacts with:119N,120S,118T

//

MESH1 mesh:D001172

NAME1 Arthritis, Rheumatoid

CLUSTER1 clust1

MESH2 mesh:D002446

NAME2 Celiac Disease

CLUSTER2 clust7

AC1 P01903

PFAM1 PF07654

PDB1 4AH2:A

AC2 P04233

PFAM2 PF00969

PDB2 4AH2:B

INT semi-edgetic

AA1 166E;Interacts with:69Y

AA1 167D;Interacts with:74Q

AA1 168H;Interacts with:74Q,91E,71L

AA2 69Y;Interacts with:166E

AA2 71L;Interacts with:168H

AA2 74Q;Interacts with:168H,167D

AA2 91E;Interacts with:168H

//

MESH1 mesh:D001172

NAME1 Arthritis, Rheumatoid

CLUSTER1 clust1

MESH2 mesh:D002446

NAME2 Celiac Disease

CLUSTER2 clust7

AC1 P01903

PFAM1 PF00993

PDB1 4AH2:A

AC2 P04233

PFAM2 PF00969

PDB2 4AH2:B

INT semi-edgetic

AA1 101R;Interacts with:125Q,113L

AA1 102S;Interacts with:113L

AA1 104Y;Interacts with:72Y

AA1 105T;Interacts with:72Y,73Q

AA1 106P;Interacts with:73Q,72Y

AA1 107I;Interacts with:73Q

AA1 29E;Interacts with:50Y,49L,45S

AA1 30H;Interacts with:178W,45S,49L,50Y

AA1 31V;Interacts with:45S,44C,49L

AA1 32I;Interacts with:177D,45S,44C,178W,49L,43K

MUT1 I32S;PMID:7528190,PMID:7869051,PMID:7636246;

AA1 33I;Interacts with:43K,32M

MUT1 I33T;856205;

AA1 34Q;Interacts with:155Y,32M

AA1 35A;Interacts with:32M

AA1 49F;Interacts with:155Y

MUT1 F49H;PMID:7528190,PMID:7869051;

AA1 77A;Interacts with:180V

AA1 95L;Interacts with:72Y

AA1 98M;Interacts with:113L,72Y

MUT1 M98I;COSMIC:71078;

AA1 99T;Interacts with:72Y

AA2 113L;Interacts with:102S,101R,98M

AA2 125Q;Interacts with:101R

AA2 155Y;Interacts with:49F,34Q

AA2 177D;Interacts with:32I

AA2 178W;Interacts with:30H,32I

AA2 180V;Interacts with:77A

AA2 32M;Interacts with:35A,33I,34Q

AA2 43K;Interacts with:33I,32I

AA2 44C;Interacts with:31V,32I

AA2 45S;Interacts with:31V,30H,32I,29E

AA2 49L;Interacts with:30H,31V,32I,29E

AA2 50Y;Interacts with:29E,30H

AA2 72Y;Interacts with:105T,95L,106P,98M,99T,104Y

AA2 73Q;Interacts with:107I,106P,105T

//

MESH1 mesh:D001172

NAME1 Arthritis, Rheumatoid

CLUSTER1 clust1

MESH2 mesh:D002446

NAME2 Celiac Disease

CLUSTER2 clust7

AC1 P01903

PFAM1 PF07654

PDB1 1BX2:A

AC2 P01911

PFAM2 PF07654

PDB2 1BX2:B

INT semi-edgetic

AA1 118T;Interacts with:185Q

AA1 119N;Interacts with:185Q,179N,181D,183T,150G

AA1 120S;Interacts with:149S,185Q

AA1 121P;Interacts with:147S,149S

MUT1 P121S;PMID:8294865,PMID:9590215;

AA1 131I;Interacts with:179N

AA1 133F;Interacts with:177I,179N,178Q

AA1 160T;Interacts with:180G

AA1 171R;Interacts with:178Q

AA1 173F;Interacts with:180G,178Q,179N

AA1 175Y;Interacts with:180G,179N,181D

AA2 147S;Interacts with:121P

AA2 149S;Interacts with:121P,120S

MUT2 S149N;dbSNP:rs74626234,dbSNP:rs116633902;

AA2 150G;Interacts with:119N

AA2 177I;Interacts with:133F

AA2 178Q;Interacts with:173F,171R,133F

MUT2 Q178H;dbSNP:rs77637983,dbSNP:rs113627837;

AA2 179N;Interacts with:119N,131I,133F,173F,175Y

AA2 180G;Interacts with:175Y,173F,160T

MUT2 G180E;dbSNP:rs1059353;

AA2 181D;Interacts with:119N,175Y

AA2 183T;Interacts with:119N

AA2 185Q;Interacts with:119N,118T,120S

//

MESH1 mesh:D001172

NAME1 Arthritis, Rheumatoid

CLUSTER1 clust1

MESH2 mesh:D002446

NAME2 Celiac Disease

CLUSTER2 clust7

AC1 P01903

PFAM1 PF07654

PDB1 1BX2:D

AC2 P01911

PFAM2 PF00969

PDB2 1BX2:E

INT semi-edgetic

AA1 138T;Interacts with:63Q

AA1 166E;Interacts with:58R

AA1 167D;Interacts with:63Q

AA1 168H;Interacts with:63Q,65E,60F

AA1 169L;Interacts with:63Q

AA2 58R;Interacts with:166E

MUT2 R58S;dbSNP:rs17878437;

AA2 60F;Interacts with:168H

MUT2 F60V;dbSNP:rs17882300;

MUT2 F60I;dbSNP:rs1059346,dbSNP:rs17882300;

AA2 63Q;Interacts with:168H,167D,138T,169L

MUT2 Q63R;dbSNP:rs17879476;

AA2 65E;Interacts with:168H

//

MESH1 mesh:D001172

NAME1 Arthritis, Rheumatoid

CLUSTER1 clust1

MESH2 mesh:D002446

NAME2 Celiac Disease

CLUSTER2 clust7

AC1 P01903

PFAM1 PF00993

PDB1 1BX2:A

AC2 P01911

PFAM2 PF00969

PDB2 1BX2:B

INT semi-edgetic

AA1 100K;Interacts with:61Y

MUT1 K100E;PMID:7869051,PMID:7722459,PMID:7636246;

AA1 101R;Interacts with:86D,82L

AA1 102S;Interacts with:61Y,82L

AA1 104Y;Interacts with:61Y

AA1 105T;Interacts with:61Y,62N

AA1 106P;Interacts with:62N,61Y

AA1 107I;Interacts with:62N

AA1 108T;Interacts with:63Q,62N

AA1 29E;Interacts with:49G,46F,48N,47F,45H

AA1 30H;Interacts with:112Y,45H,46F,47F

AA1 31V;Interacts with:45H,44C,46F

AA1 32I;Interacts with:111N,45H,44C,112Y,46F,115V,43E

MUT1 I32S;PMID:7528190,PMID:7869051,PMID:7636246;

AA1 33I;Interacts with:43E,42R,44C

MUT1 I33T;856205;

AA1 34Q;Interacts with:107Y,42R,43E

AA1 35A;Interacts with:42R

AA1 36E;Interacts with:42R

AA1 49F;Interacts with:107Y

MUT1 F49H;PMID:7528190,PMID:7869051;

AA1 51F;Interacts with:115V

AA1 56I;Interacts with:115V

AA1 77A;Interacts with:114V

AA1 87N;Interacts with:42R

AA1 91D;Interacts with:42R

MUT1 D91L;PMID:7869051;

MUT1 D91R;PMID:7869051,PMID:7636246;

AA1 95L;Interacts with:61Y

AA1 98M;Interacts with:82L,61Y

MUT1 M98I;COSMIC:71078;

AA1 99T;Interacts with:61Y

AA2 107Y;Interacts with:34Q,49F

MUT2 Y107H;dbSNP:rs17884043;

MUT2 Y107D;dbSNP:rs17884043;

MUT2 Y107F;dbSNP:rs17879620;

AA2 111N;Interacts with:32I

AA2 112Y;Interacts with:30H,32I

AA2 114V;Interacts with:77A

MUT2 V114A;dbSNP:rs17424145;

AA2 115V;Interacts with:56I,51F,32I

MUT2 V115E;dbSNP:rs17885482;

MUT2 V115M;dbSNP:rs61759935;

MUT2 V115G;158129;humsavar:VAR_038164;rs17885482;

AA2 42R;Interacts with:35A,34Q,36E,33I,87N,91D

MUT2 R42T;dbSNP:rs1136759;

MUT2 R42K;dbSNP:rs17424104,dbSNP:rs1136759;

MUT2 R42G;dbSNP:rs1136758;

MUT2 R42S;dbSNP:rs17424111,dbSNP:rs9269951;

MUT2 R42W;dbSNP:rs1136758;

MUT2 R42M;dbSNP:rs17424104,dbSNP:rs1136759;

AA2 43E;Interacts with:33I,32I,34Q

MUT2 E43K;dbSNP:rs17405212,dbSNP:rs17882014;

AA2 44C;Interacts with:31V,32I,33I

AA2 45H;Interacts with:31V,30H,32I,29E

MUT2 H45Q;dbSNP:rs17879981;

MUT2 H45Y;dbSNP:rs17879702;

AA2 46F;Interacts with:30H,31V,32I,29E

AA2 47F;Interacts with:29E,30H

MUT2 F47L;dbSNP:rs114435757;

AA2 48N;Interacts with:29E

AA2 49G;Interacts with:29E

MUT2 G49A;dbSNP:rs17879469;

MUT2 G49R;dbSNP:rs61759931;

AA2 61Y;Interacts with:102S,105T,95L,106P,98M,99T,104Y,100K

MUT2 Y61D;dbSNP:rs1064664;

MUT2 Y61H;dbSNP:rs1064664;

AA2 62N;Interacts with:107I,106P,105T,108T

MUT2 N62H;dbSNP:rs17879995;

MUT2 N62K;dbSNP:rs17879242;

AA2 63Q;Interacts with:108T

MUT2 Q63R;dbSNP:rs17879476;

AA2 82L;Interacts with:102S,101R,98M

MUT2 L82M;dbSNP:rs72558166;

AA2 86D;Interacts with:101R

MUT2 D86G;dbSNP:rs17885129;

MUT2 D86V;dbSNP:rs17885129;

MUT2 D86A;dbSNP:rs17885129;

MUT2 D86E;dbSNP:rs17853228;

MUT2 D86N;dbSNP:rs17880292;

//

MESH1 mesh:D001172

NAME1 Arthritis, Rheumatoid

CLUSTER1 clust1

MESH2 mesh:D002446

NAME2 Celiac Disease

CLUSTER2 clust7

AC1 P01903

PFAM1 PF07654

PDB1 1ZGL:A

AC2 Q30154

PFAM2 PF07654

PDB2 1ZGL:B

INT semi-edgetic

AA1 118T;Interacts with:185Q,179N

AA1 119N;Interacts with:185Q,179N,149N,150G,181D

AA1 120S;Interacts with:149N,185Q

AA1 121P;Interacts with:147S,149N,131Y

MUT1 P121S;PMID:8294865,PMID:9590215;

AA1 131I;Interacts with:179N

AA1 160T;Interacts with:180G

AA1 171R;Interacts with:178Q

AA1 173F;Interacts with:180G,178Q,179N

AA1 175Y;Interacts with:180G,181D,179N

AA2 131Y;Interacts with:121P

AA2 147S;Interacts with:121P

MUT2 S147C;dbSNP:rs184544272;

AA2 149N;Interacts with:119N,121P,120S

MUT2 N149S;dbSNP:rs114293611;

AA2 150G;Interacts with:119N

AA2 178Q;Interacts with:173F,171R

MUT2 Q178H;dbSNP:rs139485758;

AA2 179N;Interacts with:119N,131I,173F,118T,175Y

AA2 180G;Interacts with:175Y,173F,160T

MUT2 G180E;dbSNP:rs1059353;

AA2 181D;Interacts with:175Y,119N

AA2 185Q;Interacts with:119N,118T,120S

//

MESH1 mesh:D001172

NAME1 Arthritis, Rheumatoid

CLUSTER1 clust1

MESH2 mesh:D002446

NAME2 Celiac Disease

CLUSTER2 clust7

AC1 P01903

PFAM1 PF07654

PDB1 1HQR:A

AC2 Q30154

PFAM2 PF00969

PDB2 1HQR:B

INT semi-edgetic

AA1 166E;Interacts with:58R

AA1 167D;Interacts with:63Q

AA1 168H;Interacts with:60I,64E,65E,63Q

AA2 58R;Interacts with:166E

AA2 60I;Interacts with:168H

MUT2 I60F;dbSNP:rs1059346,dbSNP:rs140781802;

AA2 63Q;Interacts with:168H,167D

AA2 64E;Interacts with:168H

AA2 65E;Interacts with:168H

//

MESH1 mesh:D001172

NAME1 Arthritis, Rheumatoid

CLUSTER1 clust1

MESH2 mesh:D002446

NAME2 Celiac Disease

CLUSTER2 clust7

AC1 P01903

PFAM1 PF00993

PDB1 1ZGL:A

AC2 Q30154

PFAM2 PF00969

PDB2 1ZGL:B

INT semi-edgetic

AA1 100K;Interacts with:61Y

MUT1 K100E;PMID:7869051,PMID:7722459,PMID:7636246;

AA1 101R;Interacts with:86D,82L,66D,85P

AA1 102S;Interacts with:61Y,82L,62N

AA1 104Y;Interacts with:61Y

AA1 105T;Interacts with:61Y,62N

AA1 106P;Interacts with:62N,61Y

AA1 107I;Interacts with:62N

AA1 108T;Interacts with:63Q

AA1 29E;Interacts with:46F,49G,47F,48N

AA1 30H;Interacts with:45H,46F,112Y,47F

AA1 31V;Interacts with:45H,44C,43E,46F

AA1 32I;Interacts with:45H,44C,46F,43E,111N,112Y

MUT1 I32S;PMID:7528190,PMID:7869051,PMID:7636246;

AA1 33I;Interacts with:43E,42Y,44C

MUT1 I33T;856205;

AA1 34Q;Interacts with:107Y,42Y,43E,111N

AA1 35A;Interacts with:42Y

AA1 49F;Interacts with:107Y

MUT1 F49H;PMID:7528190,PMID:7869051;

AA1 77A;Interacts with:114V

AA1 87N;Interacts with:42Y

AA1 94N;Interacts with:59D,86D

AA1 95L;Interacts with:61Y

AA1 98M;Interacts with:82L,61Y

MUT1 M98I;COSMIC:71078;

AA1 99T;Interacts with:61Y

AA2 107Y;Interacts with:49F,34Q

MUT2 Y107V;856275;humsavar:VAR_060976;

MUT2 Y107S;dbSNP:rs1064677;

MUT2 Y107D;dbSNP:rs1064595;

MUT2 Y107F;dbSNP:rs1064677;

AA2 111N;Interacts with:34Q,32I

AA2 112Y;Interacts with:30H,32I

AA2 114V;Interacts with:77A

MUT2 V114A;158255;humsavar:VAR_060977;rs1136778;

AA2 42Y;Interacts with:33I,34Q,87N,35A

MUT2 Y42F;dbSNP:rs17424104,dbSNP:rs1136760,dbSNP:rs79937656;

MUT2 Y42S;dbSNP:rs79937656;

MUT2 Y42H;dbSNP:rs17433877,dbSNP:rs1136757;

AA2 43E;Interacts with:33I,31V,32I,34Q

MUT2 E43K;dbSNP:rs17405212,dbSNP:rs149574893;

AA2 44C;Interacts with:31V,32I,33I

AA2 45H;Interacts with:31V,30H,32I

AA2 46F;Interacts with:30H,31V,32I,29E

AA2 47F;Interacts with:29E,30H

AA2 48N;Interacts with:29E

AA2 49G;Interacts with:29E

AA2 59D;Interacts with:94N

MUT2 D59Y;dbSNP:rs138849995;

MUT2 D59H;856268;humsavar:VAR_060957;rs707955;

MUT2 D59G;158267;humsavar:VAR_060956;rs41546317;

AA2 61Y;Interacts with:102S,105T,95L,106P,100K,98M,99T,104Y

MUT2 Y61H;dbSNP:rs137863146;

AA2 62N;Interacts with:107I,106P,105T,102S

MUT2 N62H;158238;humsavar:VAR_050358;rs1059576;

AA2 63Q;Interacts with:108T

AA2 66D;Interacts with:101R

MUT2 D66N;158286;humsavar:VAR_060958;rs707956;

MUT2 D66Y;856269;humsavar:VAR_060959;rs77853982;

AA2 82L;Interacts with:102S,101R,98M

AA2 85P;Interacts with:101R

AA2 86D;Interacts with:101R,94N

MUT2 D86V;dbSNP:rs144532965;

MUT2 D86N;dbSNP:rs145586726;

//

MESH1 mesh:D001172

NAME1 Arthritis, Rheumatoid

CLUSTER1 clust1

MESH2 mesh:D003093

NAME2 Colitis, Ulcerative

CLUSTER2 clust1

AC1 P29459

PFAM1 PF03039

PDB1 3HMX:B

AC2 P29460

PFAM2 PF10420

PDB2 3HMX:A

INT non-edgetic

AA1 205R;Interacts with:203E

AA1 208T;Interacts with:203E,202A

AA1 211R;Interacts with:136Y

AA1 71H;Interacts with:205S

AA1 76K;Interacts with:205S

AA1 82V;Interacts with:201A,202A,203E

AA1 83E;Interacts with:201A,202A

AA1 85C;Interacts with:202A

AA1 86L;Interacts with:202A,200P,201A

AA2 136Y;Interacts with:211R

AA2 200P;Interacts with:86L

AA2 201A;Interacts with:82V,83E,86L

AA2 202A;Interacts with:85C,82V,86L,83E,208T

AA2 203E;Interacts with:208T,82V,205R

AA2 205S;Interacts with:71H,76K

//

MESH1 mesh:D001172

NAME1 Arthritis, Rheumatoid

CLUSTER1 clust1

MESH2 mesh:D003093

NAME2 Colitis, Ulcerative

CLUSTER2 clust1

AC1 Q13324

PFAM1 PF02793

PDB1 3N95:C

AC2 Q96RP3

PFAM2 PF11613

PDB2 3N95:E

INT non-edgetic

AA1 47I;Interacts with:105I,106L,109V

AA1 68F;Interacts with:105I,106L,102N

AA1 69N;Interacts with:98Q,102N

AA1 71V;Interacts with:99A,102N,98Q

AA1 73Y;Interacts with:106L,103A,102N

AA1 92K;Interacts with:107A,109V

MUT1 K92N;dbSNP:rs148406223;dbSNP:rs148406223;

AA1 93I;Interacts with:109V

MUT1 I93S;dbSNP:rs144584262;dbSNP:rs144584262;

AA1 94N;Interacts with:109V

AA1 95Y;Interacts with:106L,109V

AA1 96S;Interacts with:106L

AA2 102N;Interacts with:71V,69N,73Y,68F

AA2 103A;Interacts with:73Y

AA2 105I;Interacts with:47I,68F

AA2 106L;Interacts with:96S,47I,73Y,95Y,68F

AA2 107A;Interacts with:92K

AA2 109V;Interacts with:47I,94N,95Y,92K,93I

AA2 98Q;Interacts with:69N,71V

AA2 99A;Interacts with:71V

//

MESH1 mesh:D001172

NAME1 Arthritis, Rheumatoid

CLUSTER1 clust1

MESH2 mesh:D003093

NAME2 Colitis, Ulcerative

CLUSTER2 clust1

AC1 Q8IU54

PFAM1 PF15177

PDB1 3OG4:A

AC2 Q8IU57

PFAM2 PF01108

PDB2 3OG4:B

INT non-edgetic

AA1 168S;Interacts with:64T

AA1 171F;Interacts with:63P,64T,94N

AA1 174F;Interacts with:93Y

AA1 175R;Interacts with:93Y,91D

AA1 178T;Interacts with:93Y

AA1 179R;Interacts with:93Y

AA1 54R;Interacts with:93Y,94N

AA1 55D;Interacts with:95K

AA1 58E;Interacts with:95K,66R

AA1 59E;Interacts with:95K

MUT1 E59D;dbSNP:rs142971889;

AA1 66W;Interacts with:64T,63P

AA1 71P;Interacts with:65R

AA1 73F;Interacts with:65R

AA1 74P;Interacts with:65R

AA1 75G;Interacts with:65R,89K

MUT1 G75R;dbSNP:rs185252878;

AA2 63P;Interacts with:171F,66W

AA2 64T;Interacts with:66W,171F,168S

AA2 65R;Interacts with:74P,75G,71P,73F

AA2 66R;Interacts with:58E

AA2 89K;Interacts with:75G

AA2 91D;Interacts with:175R

AA2 93Y;Interacts with:179R,54R,174F,175R,178T

AA2 94N;Interacts with:54R,171F

AA2 95K;Interacts with:59E,58E,55D

//

MESH1 mesh:D001172

NAME1 Arthritis, Rheumatoid

CLUSTER1 clust1

MESH2 mesh:D003093

NAME2 Colitis, Ulcerative

CLUSTER2 clust1

AC1 Q13324

PFAM1 PF02793

PDB1 3N93:B

AC2 Q969E3

PFAM2 PF11613

PDB2 3N93:C

INT non-edgetic

AA1 47I;Interacts with:157I,154M,153L

AA1 68F;Interacts with:154M,153L,150N

AA1 69N;Interacts with:146Q,150N

AA1 70G;Interacts with:146Q,143L

AA1 71V;Interacts with:147A,150N,143L,146Q

AA1 73Y;Interacts with:151A,147A,154M,150N

AA1 92K;Interacts with:157I,156Q,155A

MUT1 K92N;dbSNP:rs148406223;dbSNP:rs148406223;

AA1 93I;Interacts with:157I

MUT1 I93S;dbSNP:rs144584262;dbSNP:rs144584262;

AA1 95Y;Interacts with:154M,157I

AA2 143L;Interacts with:71V,70G

AA2 146Q;Interacts with:70G,69N,71V

AA2 147A;Interacts with:71V,73Y

MUT2 A147V;1KG:1127728;

AA2 150N;Interacts with:71V,69N,73Y,68F

AA2 151A;Interacts with:73Y

AA2 153L;Interacts with:68F,47I

AA2 154M;Interacts with:68F,95Y,73Y,47I

AA2 155A;Interacts with:92K

AA2 156Q;Interacts with:92K

AA2 157I;Interacts with:47I,92K,95Y,93I

//

MESH1 mesh:D001172

NAME1 Arthritis, Rheumatoid

CLUSTER1 clust1

MESH2 mesh:D003093

NAME2 Colitis, Ulcerative

CLUSTER2 clust1

AC1 Q969J5

PFAM1 PF09294

PDB1 3G9V:C

AC2 Q9GZX6

PFAM2 PF14565

PDB2 3G9V:C

INT non-edgetic

AA1 200E;Interacts with:175R

AA1 246P;Interacts with:50P

AA1 247M;Interacts with:54N,50P,51Y,53T,48Q

AA1 248L;Interacts with:54N,53T,50P

AA1 249D;Interacts with:54N,50P

AA1 250R;Interacts with:54N

AA2 175R;Interacts with:200E

AA2 48Q;Interacts with:247M

AA2 50P;Interacts with:247M,249D,248L,246P

AA2 51Y;Interacts with:247M

AA2 53T;Interacts with:247M,248L

AA2 54N;Interacts with:247M,248L,250R,249D

//

MESH1 mesh:D001172

NAME1 Arthritis, Rheumatoid

CLUSTER1 clust1

MESH2 mesh:D003093

NAME2 Colitis, Ulcerative

CLUSTER2 clust1

AC1 Q969J5

PFAM1 PF01108

PDB1 3G9V:A

AC2 Q9GZX6

PFAM2 PF14565

PDB2 3G9V:A

INT non-edgetic

AA1 100G;Interacts with:70T,71D,72V,73R

AA1 101Q;Interacts with:71D,72V,70T

AA1 102R;Interacts with:71D

AA1 103Q;Interacts with:71D

AA1 104W;Interacts with:70T,71D

AA1 128Q;Interacts with:175R,73R,176N

AA1 129E;Interacts with:73R

AA1 130P;Interacts with:73R

AA1 132Y;Interacts with:70T

AA1 65K;Interacts with:70T,71D

AA1 66I;Interacts with:73R

AA1 99Y;Interacts with:70T,169L,72V,69N,73R

AA2 169L;Interacts with:99Y

AA2 175R;Interacts with:128Q

AA2 176N;Interacts with:128Q

AA2 69N;Interacts with:99Y

AA2 70T;Interacts with:104W,99Y,132Y,100G,65K,101Q

AA2 71D;Interacts with:101Q,103Q,100G,104W,65K,102R

AA2 72V;Interacts with:99Y,100G,101Q

AA2 73R;Interacts with:129E,130P,128Q,100G,99Y,66I

MUT2 R73H;dbSNP:rs149366319;

//

MESH1 mesh:D001172

NAME1 Arthritis, Rheumatoid

CLUSTER1 clust1

MESH2 mesh:D003093

NAME2 Colitis, Ulcerative

CLUSTER2 clust1

AC1 Q8N6P7

PFAM1 PF09294

PDB1 3DGC:R

AC2 Q9GZX6

PFAM2 PF14565

PDB2 3DGC:R

INT non-edgetic

AA1 133R;Interacts with:116Q,117E

AA1 134S;Interacts with:124R

AA1 136Q;Interacts with:124R

AA1 162D;Interacts with:175R,48Q,44K

AA1 175Y;Interacts with:51Y

AA1 179L;Interacts with:45S

AA1 180G;Interacts with:48Q

AA1 183Q;Interacts with:45S,43D,44K

AA1 185E;Interacts with:128R,124R,43D

AA1 186Y;Interacts with:128R

AA1 187E;Interacts with:124R,49Q,128R,45S

AA1 189F;Interacts with:46N,52I,49Q,121F,124R

AA1 190G;Interacts with:51Y,52I,49Q

AA1 191L;Interacts with:117E

AA1 206P;Interacts with:48Q

AA1 207T;Interacts with:172M,175R,48Q,44K

AA1 208W;Interacts with:57F,172M

AA2 116Q;Interacts with:133R

AA2 117E;Interacts with:191L,133R

AA2 121F;Interacts with:189F

AA2 124R;Interacts with:187E,185E,189F,134S,136Q

AA2 128R;Interacts with:187E,185E,186Y

AA2 172M;Interacts with:207T,208W

MUT2 M172V;dbSNP:rs147812317;

AA2 175R;Interacts with:162D,207T

AA2 43D;Interacts with:183Q,185E

AA2 44K;Interacts with:162D,207T,183Q

AA2 45S;Interacts with:179L,183Q,187E

AA2 46N;Interacts with:189F

AA2 48Q;Interacts with:206P,162D,207T,180G

AA2 49Q;Interacts with:187E,189F,190G

AA2 51Y;Interacts with:190G,175Y

AA2 52I;Interacts with:189F,190G

AA2 57F;Interacts with:208W

//

MESH1 mesh:D001172

NAME1 Arthritis, Rheumatoid

CLUSTER1 clust1

MESH2 mesh:D003093

NAME2 Colitis, Ulcerative

CLUSTER2 clust1

AC1 Q8N6P7

PFAM1 PF01108

PDB1 3DGC:R

AC2 Q9GZX6

PFAM2 PF14565

PDB2 3DGC:R

INT non-edgetic

AA1 58K;Interacts with:71D,70T

MUT1 K58A;882419;

AA1 60Y;Interacts with:72V,70T,169L,73R,69N,74L

MUT1 Y60A;1368880;

MUT1 Y60R;1368881;

AA1 61G;Interacts with:73R,72V,70T,71D

AA1 62E;Interacts with:72V,70T,71D

AA1 63R;Interacts with:71D

AA1 64D;Interacts with:71D

AA1 65W;Interacts with:70T

AA1 89T;Interacts with:77E,73R

AA1 90E;Interacts with:73R

AA1 91L;Interacts with:73R,169L

MUT1 L91F;dbSNP:rs144035143;

AA1 93Y;Interacts with:70T

AA2 169L;Interacts with:60Y,91L

AA2 69N;Interacts with:60Y

AA2 70T;Interacts with:58K,61G,60Y,62E,93Y,65W

AA2 71D;Interacts with:58K,61G,63R,64D,62E

AA2 72V;Interacts with:62E,60Y,61G

AA2 73R;Interacts with:61G,90E,89T,60Y,91L

MUT2 R73H;dbSNP:rs149366319;

AA2 74L;Interacts with:60Y

AA2 77E;Interacts with:89T

//

MESH1 mesh:D001172

NAME1 Arthritis, Rheumatoid

CLUSTER1 clust1

MESH2 mesh:D003093

NAME2 Colitis, Ulcerative

CLUSTER2 clust1

AC1 P22301

PFAM1 PF00726

PDB1 1J7V:L

AC2 Q13651

PFAM2 PF09294

PDB2 1J7V:R

INT non-edgetic

AA1 38P;Interacts with:212R,211S,164F,210A

AA1 39N;Interacts with:210A

AA1 41L;Interacts with:211S

AA1 42R;Interacts with:211S,214N,208S,212R,166E,210A,213S

AA1 43D;Interacts with:211S

AA1 45R;Interacts with:211S

AA1 46D;Interacts with:213S

AA2 164F;Interacts with:38P

AA2 166E;Interacts with:42R

AA2 208S;Interacts with:42R

AA2 210A;Interacts with:39N,42R,38P

AA2 211S;Interacts with:42R,38P,41L,45R,43D

AA2 212R;Interacts with:38P,42R

AA2 213S;Interacts with:46D,42R

AA2 214N;Interacts with:42R

//

MESH1 mesh:D001172

NAME1 Arthritis, Rheumatoid

CLUSTER1 clust1

MESH2 mesh:D003093

NAME2 Colitis, Ulcerative

CLUSTER2 clust1

AC1 P22301

PFAM1 PF00726

PDB1 1J7V:L

AC2 Q13651

PFAM2 PF01108

PDB2 1J7V:R

INT non-edgetic

AA1 56Q;Interacts with:97R

AA1 59D;Interacts with:97R

AA1 60Q;Interacts with:97R

MUT1 Q60E;PMID:10637267;

AA1 61L;Interacts with:97R,64Y

AA1 62D;Interacts with:69W,66I,65G,67E,97R,64Y

AA1 63N;Interacts with:67E,65G,64Y

AA1 64L;Interacts with:64Y,67E,65G

AA1 65L;Interacts with:64Y

AA1 66L;Interacts with:94N

AA2 64Y;Interacts with:65L,64L,61L,62D,63N

AA2 65G;Interacts with:62D,63N,64L

AA2 66I;Interacts with:62D

AA2 67E;Interacts with:63N,62D,64L

AA2 69W;Interacts with:62D

AA2 94N;Interacts with:66L

AA2 97R;Interacts with:60Q,61L,59D,62D,56Q

//

MESH1 mesh:D001172

NAME1 Arthritis, Rheumatoid

CLUSTER1 clust1

MESH2 mesh:D003093

NAME2 Colitis, Ulcerative

CLUSTER2 clust1

AC1 P18510

PFAM1 PF00340

PDB1 1IRA:X

AC2 P14778

PFAM2 PF13895

PDB2 1IRA:Y

INT semi-edgetic

AA1 151E;Interacts with:146E

MUT1 E151G;PMID:7744786;

AA1 152A;Interacts with:146E,144Y

AA1 172Y;Interacts with:139G,132L

AA1 60L;Interacts with:128F,129K,144Y

AA1 61Q;Interacts with:127I,126A,125Q,128F

AA1 62G;Interacts with:125Q,127I

AA1 63P;Interacts with:125Q

AA1 68E;Interacts with:131K

AA2 125Q;Interacts with:63P,62G,61Q

MUT2 Q125K;dbSNP:rs76949245;

AA2 126A;Interacts with:61Q

AA2 127I;Interacts with:61Q,62G

AA2 128F;Interacts with:60L,61Q

AA2 129K;Interacts with:60L

AA2 131K;Interacts with:68E

AA2 132L;Interacts with:172Y

AA2 139G;Interacts with:172Y

AA2 144Y;Interacts with:152A,60L

AA2 146E;Interacts with:152A,151E

//

MESH1 mesh:D001172

NAME1 Arthritis, Rheumatoid

CLUSTER1 clust1

MESH2 mesh:D003093

NAME2 Colitis, Ulcerative

CLUSTER2 clust1

AC1 P01579

PFAM1 PF00714

PDB1 1FYH:A

AC2 P15260

PFAM2 PF07140

PDB2 1FYH:E

INT edgetic

AA1 146A;Interacts with:186K,203A,205P,185Y

AA1 147A;Interacts with:183I,185Y

MUT1 A147L;PMID:1662603;

AA1 149T;Interacts with:185Y

AA1 150G;Interacts with:208S,205P,207S

AA1 151K;Interacts with:209L,207S,208S

MUT1 K151R;PMID:2127102;

MUT1 K151E;PMID:1830392;

AA2 183I;Interacts with:147A

AA2 185Y;Interacts with:146A,147A,149T

AA2 186K;Interacts with:146A

AA2 203A;Interacts with:146A

AA2 205P;Interacts with:150G,146A

AA2 207S;Interacts with:151K,150G

AA2 208S;Interacts with:150G,151K

AA2 209L;Interacts with:151K

//

MESH1 mesh:D001172

NAME1 Arthritis, Rheumatoid

CLUSTER1 clust1

MESH2 mesh:D003093

NAME2 Colitis, Ulcerative

CLUSTER2 clust1

AC1 P01579

PFAM1 PF00714

PDB1 1FYH:A

AC2 P15260

PFAM2 PF01108

PDB2 1FYH:B

INT edgetic

AA1 163R;Interacts with:66Y

AA2 66Y;Interacts with:163R

MUT2 Y66A;PMID:9878445;

MUT2 Y66F;PMID:9878445;

//

MESH1 mesh:D001172

NAME1 Arthritis, Rheumatoid

CLUSTER1 clust1

MESH2 mesh:D003424

NAME2 Crohn Disease

CLUSTER2 clust1

AC1 P29459

PFAM1 PF03039

PDB1 3HMX:B

AC2 P29460

PFAM2 PF10420

PDB2 3HMX:A

INT non-edgetic

AA1 205R;Interacts with:203E

AA1 208T;Interacts with:203E,202A

AA1 211R;Interacts with:136Y

AA1 71H;Interacts with:205S

AA1 76K;Interacts with:205S

AA1 82V;Interacts with:201A,202A,203E

AA1 83E;Interacts with:201A,202A

AA1 85C;Interacts with:202A

AA1 86L;Interacts with:202A,200P,201A

AA2 136Y;Interacts with:211R

AA2 200P;Interacts with:86L

AA2 201A;Interacts with:82V,83E,86L

AA2 202A;Interacts with:85C,82V,86L,83E,208T

AA2 203E;Interacts with:208T,82V,205R

AA2 205S;Interacts with:71H,76K

//

MESH1 mesh:D001172

NAME1 Arthritis, Rheumatoid

CLUSTER1 clust1

MESH2 mesh:D003424

NAME2 Crohn Disease

CLUSTER2 clust1

AC1 Q13324

PFAM1 PF02793

PDB1 3N95:C

AC2 Q96RP3

PFAM2 PF11613

PDB2 3N95:E

INT non-edgetic

AA1 47I;Interacts with:105I,106L,109V

AA1 68F;Interacts with:105I,106L,102N

AA1 69N;Interacts with:98Q,102N

AA1 71V;Interacts with:99A,102N,98Q

AA1 73Y;Interacts with:106L,103A,102N

AA1 92K;Interacts with:107A,109V

MUT1 K92N;dbSNP:rs148406223;dbSNP:rs148406223;

AA1 93I;Interacts with:109V

MUT1 I93S;dbSNP:rs144584262;dbSNP:rs144584262;

AA1 94N;Interacts with:109V

AA1 95Y;Interacts with:106L,109V

AA1 96S;Interacts with:106L

AA2 102N;Interacts with:71V,69N,73Y,68F

AA2 103A;Interacts with:73Y

AA2 105I;Interacts with:47I,68F

AA2 106L;Interacts with:96S,47I,73Y,95Y,68F

AA2 107A;Interacts with:92K

AA2 109V;Interacts with:47I,94N,95Y,92K,93I

AA2 98Q;Interacts with:69N,71V

AA2 99A;Interacts with:71V

//

MESH1 mesh:D001172

NAME1 Arthritis, Rheumatoid

CLUSTER1 clust1

MESH2 mesh:D003424

NAME2 Crohn Disease

CLUSTER2 clust1

AC1 Q8IU54

PFAM1 PF15177

PDB1 3OG4:A

AC2 Q8IU57

PFAM2 PF01108

PDB2 3OG4:B

INT non-edgetic

AA1 168S;Interacts with:64T

AA1 171F;Interacts with:63P,64T,94N

AA1 174F;Interacts with:93Y

AA1 175R;Interacts with:93Y,91D

AA1 178T;Interacts with:93Y

AA1 179R;Interacts with:93Y

AA1 54R;Interacts with:93Y,94N

AA1 55D;Interacts with:95K

AA1 58E;Interacts with:95K,66R

AA1 59E;Interacts with:95K

MUT1 E59D;dbSNP:rs142971889;

AA1 66W;Interacts with:64T,63P

AA1 71P;Interacts with:65R

AA1 73F;Interacts with:65R

AA1 74P;Interacts with:65R

AA1 75G;Interacts with:65R,89K

MUT1 G75R;dbSNP:rs185252878;

AA2 63P;Interacts with:171F,66W

AA2 64T;Interacts with:66W,171F,168S

AA2 65R;Interacts with:74P,75G,71P,73F

AA2 66R;Interacts with:58E

AA2 89K;Interacts with:75G

AA2 91D;Interacts with:175R

AA2 93Y;Interacts with:179R,54R,174F,175R,178T

AA2 94N;Interacts with:54R,171F

AA2 95K;Interacts with:59E,58E,55D

//

MESH1 mesh:D001172

NAME1 Arthritis, Rheumatoid

CLUSTER1 clust1

MESH2 mesh:D003424

NAME2 Crohn Disease

CLUSTER2 clust1

AC1 Q13324

PFAM1 PF02793

PDB1 3N93:B

AC2 Q969E3

PFAM2 PF11613

PDB2 3N93:C

INT non-edgetic

AA1 47I;Interacts with:157I,154M,153L

AA1 68F;Interacts with:154M,153L,150N

AA1 69N;Interacts with:146Q,150N

AA1 70G;Interacts with:146Q,143L

AA1 71V;Interacts with:147A,150N,143L,146Q

AA1 73Y;Interacts with:151A,147A,154M,150N

AA1 92K;Interacts with:157I,156Q,155A

MUT1 K92N;dbSNP:rs148406223;dbSNP:rs148406223;

AA1 93I;Interacts with:157I

MUT1 I93S;dbSNP:rs144584262;dbSNP:rs144584262;

AA1 95Y;Interacts with:154M,157I

AA2 143L;Interacts with:71V,70G

AA2 146Q;Interacts with:70G,69N,71V

AA2 147A;Interacts with:71V,73Y

MUT2 A147V;1KG:1127728;

AA2 150N;Interacts with:71V,69N,73Y,68F

AA2 151A;Interacts with:73Y

AA2 153L;Interacts with:68F,47I

AA2 154M;Interacts with:68F,95Y,73Y,47I

AA2 155A;Interacts with:92K

AA2 156Q;Interacts with:92K

AA2 157I;Interacts with:47I,92K,95Y,93I

//

MESH1 mesh:D001172

NAME1 Arthritis, Rheumatoid

CLUSTER1 clust1

MESH2 mesh:D003424

NAME2 Crohn Disease

CLUSTER2 clust1

AC1 Q969J5

PFAM1 PF09294

PDB1 3G9V:C

AC2 Q9GZX6

PFAM2 PF14565

PDB2 3G9V:C

INT non-edgetic

AA1 200E;Interacts with:175R

AA1 246P;Interacts with:50P

AA1 247M;Interacts with:54N,50P,51Y,53T,48Q

AA1 248L;Interacts with:54N,53T,50P

AA1 249D;Interacts with:54N,50P

AA1 250R;Interacts with:54N

AA2 175R;Interacts with:200E

AA2 48Q;Interacts with:247M

AA2 50P;Interacts with:247M,249D,248L,246P

AA2 51Y;Interacts with:247M

AA2 53T;Interacts with:247M,248L

AA2 54N;Interacts with:247M,248L,250R,249D

//

MESH1 mesh:D001172

NAME1 Arthritis, Rheumatoid

CLUSTER1 clust1

MESH2 mesh:D003424

NAME2 Crohn Disease

CLUSTER2 clust1

AC1 Q969J5

PFAM1 PF01108

PDB1 3G9V:A

AC2 Q9GZX6

PFAM2 PF14565

PDB2 3G9V:A

INT non-edgetic

AA1 100G;Interacts with:70T,71D,72V,73R

AA1 101Q;Interacts with:71D,72V,70T

AA1 102R;Interacts with:71D

AA1 103Q;Interacts with:71D

AA1 104W;Interacts with:70T,71D

AA1 128Q;Interacts with:175R,73R,176N

AA1 129E;Interacts with:73R

AA1 130P;Interacts with:73R

AA1 132Y;Interacts with:70T

AA1 65K;Interacts with:70T,71D

AA1 66I;Interacts with:73R

AA1 99Y;Interacts with:70T,169L,72V,69N,73R

AA2 169L;Interacts with:99Y

AA2 175R;Interacts with:128Q

AA2 176N;Interacts with:128Q

AA2 69N;Interacts with:99Y

AA2 70T;Interacts with:104W,99Y,132Y,100G,65K,101Q

AA2 71D;Interacts with:101Q,103Q,100G,104W,65K,102R

AA2 72V;Interacts with:99Y,100G,101Q

AA2 73R;Interacts with:129E,130P,128Q,100G,99Y,66I

MUT2 R73H;dbSNP:rs149366319;

//

MESH1 mesh:D001172

NAME1 Arthritis, Rheumatoid

CLUSTER1 clust1

MESH2 mesh:D003424

NAME2 Crohn Disease

CLUSTER2 clust1

AC1 Q8N6P7

PFAM1 PF09294

PDB1 3DGC:R

AC2 Q9GZX6

PFAM2 PF14565

PDB2 3DGC:R

INT non-edgetic

AA1 133R;Interacts with:116Q,117E

AA1 134S;Interacts with:124R

AA1 136Q;Interacts with:124R

AA1 162D;Interacts with:175R,48Q,44K

AA1 175Y;Interacts with:51Y

AA1 179L;Interacts with:45S

AA1 180G;Interacts with:48Q

AA1 183Q;Interacts with:45S,43D,44K

AA1 185E;Interacts with:128R,124R,43D

AA1 186Y;Interacts with:128R

AA1 187E;Interacts with:124R,49Q,128R,45S

AA1 189F;Interacts with:46N,52I,49Q,121F,124R

AA1 190G;Interacts with:51Y,52I,49Q

AA1 191L;Interacts with:117E

AA1 206P;Interacts with:48Q

AA1 207T;Interacts with:172M,175R,48Q,44K

AA1 208W;Interacts with:57F,172M

AA2 116Q;Interacts with:133R

AA2 117E;Interacts with:191L,133R

AA2 121F;Interacts with:189F

AA2 124R;Interacts with:187E,185E,189F,134S,136Q

AA2 128R;Interacts with:187E,185E,186Y

AA2 172M;Interacts with:207T,208W

MUT2 M172V;dbSNP:rs147812317;

AA2 175R;Interacts with:162D,207T

AA2 43D;Interacts with:183Q,185E

AA2 44K;Interacts with:162D,207T,183Q

AA2 45S;Interacts with:179L,183Q,187E

AA2 46N;Interacts with:189F

AA2 48Q;Interacts with:206P,162D,207T,180G

AA2 49Q;Interacts with:187E,189F,190G

AA2 51Y;Interacts with:190G,175Y

AA2 52I;Interacts with:189F,190G

AA2 57F;Interacts with:208W

//

MESH1 mesh:D001172

NAME1 Arthritis, Rheumatoid

CLUSTER1 clust1

MESH2 mesh:D003424

NAME2 Crohn Disease

CLUSTER2 clust1

AC1 Q8N6P7

PFAM1 PF01108

PDB1 3DGC:R

AC2 Q9GZX6

PFAM2 PF14565

PDB2 3DGC:R

INT non-edgetic

AA1 58K;Interacts with:71D,70T

MUT1 K58A;882419;

AA1 60Y;Interacts with:72V,70T,169L,73R,69N,74L

MUT1 Y60A;1368880;

MUT1 Y60R;1368881;

AA1 61G;Interacts with:73R,72V,70T,71D

AA1 62E;Interacts with:72V,70T,71D

AA1 63R;Interacts with:71D

AA1 64D;Interacts with:71D

AA1 65W;Interacts with:70T

AA1 89T;Interacts with:77E,73R

AA1 90E;Interacts with:73R

AA1 91L;Interacts with:73R,169L

MUT1 L91F;dbSNP:rs144035143;

AA1 93Y;Interacts with:70T

AA2 169L;Interacts with:60Y,91L

AA2 69N;Interacts with:60Y

AA2 70T;Interacts with:58K,61G,60Y,62E,93Y,65W

AA2 71D;Interacts with:58K,61G,63R,64D,62E

AA2 72V;Interacts with:62E,60Y,61G

AA2 73R;Interacts with:61G,90E,89T,60Y,91L

MUT2 R73H;dbSNP:rs149366319;

AA2 74L;Interacts with:60Y

AA2 77E;Interacts with:89T

//

MESH1 mesh:D001172

NAME1 Arthritis, Rheumatoid

CLUSTER1 clust1

MESH2 mesh:D003424

NAME2 Crohn Disease

CLUSTER2 clust1

AC1 P22301

PFAM1 PF00726

PDB1 1J7V:L

AC2 Q13651

PFAM2 PF09294

PDB2 1J7V:R

INT non-edgetic

AA1 38P;Interacts with:212R,211S,164F,210A

AA1 39N;Interacts with:210A

AA1 41L;Interacts with:211S

AA1 42R;Interacts with:211S,214N,208S,212R,166E,210A,213S

AA1 43D;Interacts with:211S

AA1 45R;Interacts with:211S

AA1 46D;Interacts with:213S

AA2 164F;Interacts with:38P

AA2 166E;Interacts with:42R

AA2 208S;Interacts with:42R

AA2 210A;Interacts with:39N,42R,38P

AA2 211S;Interacts with:42R,38P,41L,45R,43D

AA2 212R;Interacts with:38P,42R

AA2 213S;Interacts with:46D,42R

AA2 214N;Interacts with:42R

//

MESH1 mesh:D001172

NAME1 Arthritis, Rheumatoid

CLUSTER1 clust1

MESH2 mesh:D003424

NAME2 Crohn Disease

CLUSTER2 clust1

AC1 P22301

PFAM1 PF00726

PDB1 1J7V:L

AC2 Q13651

PFAM2 PF01108

PDB2 1J7V:R

INT non-edgetic

AA1 56Q;Interacts with:97R

AA1 59D;Interacts with:97R

AA1 60Q;Interacts with:97R

MUT1 Q60E;PMID:10637267;

AA1 61L;Interacts with:97R,64Y

AA1 62D;Interacts with:69W,66I,65G,67E,97R,64Y

AA1 63N;Interacts with:67E,65G,64Y

AA1 64L;Interacts with:64Y,67E,65G

AA1 65L;Interacts with:64Y

AA1 66L;Interacts with:94N

AA2 64Y;Interacts with:65L,64L,61L,62D,63N

AA2 65G;Interacts with:62D,63N,64L

AA2 66I;Interacts with:62D

AA2 67E;Interacts with:63N,62D,64L

AA2 69W;Interacts with:62D

AA2 94N;Interacts with:66L

AA2 97R;Interacts with:60Q,61L,59D,62D,56Q

//

MESH1 mesh:D001172

NAME1 Arthritis, Rheumatoid

CLUSTER1 clust1

MESH2 mesh:D003424

NAME2 Crohn Disease

CLUSTER2 clust1

AC1 P18510

PFAM1 PF00340

PDB1 1IRA:X

AC2 P14778

PFAM2 PF13895

PDB2 1IRA:Y

INT semi-edgetic

AA1 151E;Interacts with:146E

MUT1 E151G;PMID:7744786;

AA1 152A;Interacts with:146E,144Y

AA1 172Y;Interacts with:139G,132L

AA1 60L;Interacts with:128F,129K,144Y

AA1 61Q;Interacts with:127I,126A,125Q,128F

AA1 62G;Interacts with:125Q,127I

AA1 63P;Interacts with:125Q

AA1 68E;Interacts with:131K

AA2 125Q;Interacts with:63P,62G,61Q

MUT2 Q125K;dbSNP:rs76949245;

AA2 126A;Interacts with:61Q

AA2 127I;Interacts with:61Q,62G

AA2 128F;Interacts with:60L,61Q

AA2 129K;Interacts with:60L

AA2 131K;Interacts with:68E

AA2 132L;Interacts with:172Y

AA2 139G;Interacts with:172Y

AA2 144Y;Interacts with:152A,60L

AA2 146E;Interacts with:152A,151E

//

MESH1 mesh:D001172

NAME1 Arthritis, Rheumatoid

CLUSTER1 clust1

MESH2 mesh:D003424

NAME2 Crohn Disease

CLUSTER2 clust1

AC1 P01579

PFAM1 PF00714

PDB1 1FYH:A

AC2 P15260

PFAM2 PF07140

PDB2 1FYH:E

INT edgetic

AA1 146A;Interacts with:186K,203A,205P,185Y

AA1 147A;Interacts with:183I,185Y

MUT1 A147L;PMID:1662603;

AA1 149T;Interacts with:185Y

AA1 150G;Interacts with:208S,205P,207S

AA1 151K;Interacts with:209L,207S,208S

MUT1 K151R;PMID:2127102;

MUT1 K151E;PMID:1830392;

AA2 183I;Interacts with:147A

AA2 185Y;Interacts with:146A,147A,149T

AA2 186K;Interacts with:146A

AA2 203A;Interacts with:146A

AA2 205P;Interacts with:150G,146A

AA2 207S;Interacts with:151K,150G

AA2 208S;Interacts with:150G,151K

AA2 209L;Interacts with:151K

//

MESH1 mesh:D001172

NAME1 Arthritis, Rheumatoid

CLUSTER1 clust1

MESH2 mesh:D003424

NAME2 Crohn Disease

CLUSTER2 clust1

AC1 P01579

PFAM1 PF00714

PDB1 1FYH:A

AC2 P15260

PFAM2 PF01108

PDB2 1FYH:B

INT edgetic

AA1 163R;Interacts with:66Y

AA2 66Y;Interacts with:163R

MUT2 Y66A;PMID:9878445;

MUT2 Y66F;PMID:9878445;

//

MESH1 mesh:D001172

NAME1 Arthritis, Rheumatoid

CLUSTER1 clust1

MESH2 mesh:D006526

NAME2 Hepatitis C

CLUSTER2 clust1

AC1 P29459

PFAM1 PF03039

PDB1 3HMX:B

AC2 P29460

PFAM2 PF10420

PDB2 3HMX:A

INT non-edgetic

AA1 205R;Interacts with:203E

AA1 208T;Interacts with:203E,202A

AA1 211R;Interacts with:136Y

AA1 71H;Interacts with:205S

AA1 76K;Interacts with:205S

AA1 82V;Interacts with:201A,202A,203E

AA1 83E;Interacts with:201A,202A

AA1 85C;Interacts with:202A

AA1 86L;Interacts with:202A,200P,201A

AA2 136Y;Interacts with:211R

AA2 200P;Interacts with:86L

AA2 201A;Interacts with:82V,83E,86L

AA2 202A;Interacts with:85C,82V,86L,83E,208T

AA2 203E;Interacts with:208T,82V,205R

AA2 205S;Interacts with:71H,76K

//

MESH1 mesh:D001172

NAME1 Arthritis, Rheumatoid

CLUSTER1 clust1

MESH2 mesh:D006526

NAME2 Hepatitis C

CLUSTER2 clust1

AC1 P04440

PFAM1 PF07654

PDB1 3LQZ:B

AC2 P20036

PFAM2 PF00993

PDB2 3LQZ:A

INT semi-edgetic

AA1 176R;Interacts with:59E,58D,60D

AA1 178G;Interacts with:60D,75H

AA1 179D;Interacts with:60D,75H

AA1 180W;Interacts with:62M,60D,61E,75H,57F,79F,76L

AA1 181T;Interacts with:60D

AA2 57F;Interacts with:180W

AA2 58D;Interacts with:176R

AA2 59E;Interacts with:176R

MUT2 E59D;157591;humsavar:VAR_058835;rs2308910;

AA2 60D;Interacts with:180W,179D,178G,176R,181T

AA2 61E;Interacts with:180W

AA2 62M;Interacts with:180W

MUT2 M62L;157592;humsavar:VAR_047685;rs2308911;

MUT2 M62K;157593;humsavar:VAR_058836;rs2308912;

MUT2 M62Q;157599;humsavar:VAR_058850;rs36013091;

AA2 75H;Interacts with:180W,179D,178G

AA2 76L;Interacts with:180W

AA2 79F;Interacts with:180W

//

MESH1 mesh:D001172

NAME1 Arthritis, Rheumatoid

CLUSTER1 clust1

MESH2 mesh:D006526

NAME2 Hepatitis C

CLUSTER2 clust1

AC1 P01579

PFAM1 PF00714

PDB1 1FYH:A

AC2 P15260

PFAM2 PF07140

PDB2 1FYH:E

INT semi-edgetic

AA1 146A;Interacts with:186K,203A,205P,185Y

AA1 147A;Interacts with:183I,185Y

MUT1 A147L;PMID:1662603;

AA1 149T;Interacts with:185Y

AA1 150G;Interacts with:208S,205P,207S

AA1 151K;Interacts with:209L,207S,208S

MUT1 K151R;PMID:2127102;

MUT1 K151E;PMID:1830392;

AA2 183I;Interacts with:147A

AA2 185Y;Interacts with:146A,147A,149T

AA2 186K;Interacts with:146A

AA2 203A;Interacts with:146A

AA2 205P;Interacts with:150G,146A

AA2 207S;Interacts with:151K,150G

AA2 208S;Interacts with:150G,151K

AA2 209L;Interacts with:151K

//

MESH1 mesh:D001172

NAME1 Arthritis, Rheumatoid

CLUSTER1 clust1

MESH2 mesh:D006526

NAME2 Hepatitis C

CLUSTER2 clust1

AC1 P01579

PFAM1 PF00714

PDB1 1FYH:A

AC2 P15260

PFAM2 PF01108

PDB2 1FYH:B

INT semi-edgetic

AA1 163R;Interacts with:66Y

AA2 66Y;Interacts with:163R

MUT2 Y66A;PMID:9878445;

MUT2 Y66F;PMID:9878445;

//

MESH1 mesh:D001172

NAME1 Arthritis, Rheumatoid

CLUSTER1 clust1

MESH2 mesh:D008180

NAME2 Systemic lupus erythematosus

CLUSTER2 clust1

AC1 P29459

PFAM1 PF03039

PDB1 3HMX:B

AC2 P29460

PFAM2 PF10420

PDB2 3HMX:A

INT non-edgetic

AA1 205R;Interacts with:203E

AA1 208T;Interacts with:203E,202A

AA1 211R;Interacts with:136Y

AA1 71H;Interacts with:205S

AA1 76K;Interacts with:205S

AA1 82V;Interacts with:201A,202A,203E

AA1 83E;Interacts with:201A,202A

AA1 85C;Interacts with:202A

AA1 86L;Interacts with:202A,200P,201A

AA2 136Y;Interacts with:211R

AA2 200P;Interacts with:86L

AA2 201A;Interacts with:82V,83E,86L

AA2 202A;Interacts with:85C,82V,86L,83E,208T

AA2 203E;Interacts with:208T,82V,205R

AA2 205S;Interacts with:71H,76K

//

MESH1 mesh:D001172

NAME1 Arthritis, Rheumatoid

CLUSTER1 clust1

MESH2 mesh:D008180

NAME2 Systemic lupus erythematosus

CLUSTER2 clust1

AC1 Q13324

PFAM1 PF02793

PDB1 3N95:C

AC2 Q96RP3

PFAM2 PF11613

PDB2 3N95:E

INT semi-edgetic

AA1 47I;Interacts with:105I,106L,109V

AA1 68F;Interacts with:105I,106L,102N

AA1 69N;Interacts with:98Q,102N

AA1 71V;Interacts with:99A,102N,98Q

AA1 73Y;Interacts with:106L,103A,102N

AA1 92K;Interacts with:107A,109V

MUT1 K92N;dbSNP:rs148406223;dbSNP:rs148406223;

AA1 93I;Interacts with:109V

MUT1 I93S;dbSNP:rs144584262;dbSNP:rs144584262;

AA1 94N;Interacts with:109V

AA1 95Y;Interacts with:106L,109V

AA1 96S;Interacts with:106L

AA2 102N;Interacts with:71V,69N,73Y,68F

AA2 103A;Interacts with:73Y

AA2 105I;Interacts with:47I,68F

AA2 106L;Interacts with:96S,47I,73Y,95Y,68F

AA2 107A;Interacts with:92K

AA2 109V;Interacts with:47I,94N,95Y,92K,93I

AA2 98Q;Interacts with:69N,71V

AA2 99A;Interacts with:71V

//

MESH1 mesh:D001172

NAME1 Arthritis, Rheumatoid

CLUSTER1 clust1

MESH2 mesh:D008180

NAME2 Systemic lupus erythematosus

CLUSTER2 clust1

AC1 Q13324

PFAM1 PF02793

PDB1 3N93:B

AC2 Q969E3

PFAM2 PF11613

PDB2 3N93:C

INT semi-edgetic

AA1 47I;Interacts with:157I,154M,153L

AA1 68F;Interacts with:154M,153L,150N

AA1 69N;Interacts with:146Q,150N

AA1 70G;Interacts with:146Q,143L

AA1 71V;Interacts with:147A,150N,143L,146Q

AA1 73Y;Interacts with:151A,147A,154M,150N

AA1 92K;Interacts with:157I,156Q,155A

MUT1 K92N;dbSNP:rs148406223;dbSNP:rs148406223;

AA1 93I;Interacts with:157I

MUT1 I93S;dbSNP:rs144584262;dbSNP:rs144584262;

AA1 95Y;Interacts with:154M,157I

AA2 143L;Interacts with:71V,70G

AA2 146Q;Interacts with:70G,69N,71V

AA2 147A;Interacts with:71V,73Y

MUT2 A147V;1KG:1127728;

AA2 150N;Interacts with:71V,69N,73Y,68F

AA2 151A;Interacts with:73Y

AA2 153L;Interacts with:68F,47I

AA2 154M;Interacts with:68F,95Y,73Y,47I

AA2 155A;Interacts with:92K

AA2 156Q;Interacts with:92K

AA2 157I;Interacts with:47I,92K,95Y,93I

//

MESH1 mesh:D001172

NAME1 Arthritis, Rheumatoid

CLUSTER1 clust1

MESH2 mesh:D008180

NAME2 Systemic lupus erythematosus

CLUSTER2 clust1

AC1 O95150

PFAM1 PF00229

PDB1 3K51:A

AC2 O95407

PFAM2 PF00020

PDB2 3K51:B

INT semi-edgetic

AA1 186D;Interacts with:84Y

AA1 187S;Interacts with:83N,84Y,85L

AA1 188Y;Interacts with:81F,85L,83N,89R,79T,80Q

AA1 189P;Interacts with:82W

AA1 190E;Interacts with:89R

AA1 191P;Interacts with:89R

AA1 192T;Interacts with:89R

AA1 221Q;Interacts with:86E

AA2 79T;Interacts with:188Y

AA2 80Q;Interacts with:188Y

AA2 81F;Interacts with:188Y

AA2 82W;Interacts with:189P

AA2 83N;Interacts with:187S,188Y

AA2 84Y;Interacts with:187S,186D

AA2 85L;Interacts with:188Y,187S

AA2 86E;Interacts with:221Q

AA2 89R;Interacts with:190E,192T,188Y,191P

//

MESH1 mesh:D001172

NAME1 Arthritis, Rheumatoid

CLUSTER1 clust1

MESH2 mesh:D008180

NAME2 Systemic lupus erythematosus

CLUSTER2 clust1

AC1 O43557

PFAM1 PF00229

PDB1 4J6G:A

AC2 O95407

PFAM2 PF00020

PDB2 4J6G:C

INT semi-edgetic

AA1 115E;Interacts with:76R

AA1 118L;Interacts with:77H,90Y

AA1 119G;Interacts with:90Y

AA1 168K;Interacts with:89R

AA1 170T;Interacts with:85L

AA1 171P;Interacts with:84Y

AA1 172R;Interacts with:84Y,83N,86E,85L

AA1 173Y;Interacts with:85L,89R,81F,79T,80Q

AA1 174P;Interacts with:82W

AA1 175E;Interacts with:89R

AA1 176E;Interacts with:89R

AA1 178E;Interacts with:92N

AA1 227L;Interacts with:94L

AA1 228R;Interacts with:99E,93V,94L

AA1 229D;Interacts with:92N,93V,94L

AA1 230G;Interacts with:92N

AA2 76R;Interacts with:115E

AA2 77H;Interacts with:118L

AA2 79T;Interacts with:173Y

AA2 80Q;Interacts with:173Y

AA2 81F;Interacts with:173Y

AA2 82W;Interacts with:174P

AA2 83N;Interacts with:172R

AA2 84Y;Interacts with:172R,171P

AA2 85L;Interacts with:173Y,170T,172R

AA2 86E;Interacts with:172R

AA2 89R;Interacts with:175E,176E,168K,173Y

AA2 90Y;Interacts with:119G,118L

AA2 92N;Interacts with:178E,229D,230G

AA2 93V;Interacts with:228R,229D

MUT2 V93I;dbSNP:rs185771249;

AA2 94L;Interacts with:227L,228R,229D

AA2 99E;Interacts with:228R

//

MESH1 mesh:D001172

NAME1 Arthritis, Rheumatoid

CLUSTER1 clust1

MESH2 mesh:D008180

NAME2 Systemic lupus erythematosus

CLUSTER2 clust1

AC1 P23510

PFAM1 PF00229

PDB1 2HEV:F

AC2 P43489

PFAM2 PF00020

PDB2 2HEV:R

INT semi-edgetic

AA1 144T;Interacts with:55R

AA1 145Y;Interacts with:37P,36Y,55R

AA1 146K;Interacts with:34D,33G,45E,35T

AA1 180F;Interacts with:38S,37P,36Y

AA1 182V;Interacts with:37P

AA1 98D;Interacts with:55R

AA2 33G;Interacts with:146K

AA2 34D;Interacts with:146K

AA2 35T;Interacts with:146K

AA2 36Y;Interacts with:180F,145Y

AA2 37P;Interacts with:145Y,180F,182V

AA2 38S;Interacts with:180F

AA2 45E;Interacts with:146K

AA2 55R;Interacts with:98D,144T,145Y

//

MESH1 mesh:D001172

NAME1 Arthritis, Rheumatoid

CLUSTER1 clust1

MESH2 mesh:D009765

NAME2 Obesity

CLUSTER2 clust1

AC1 Q13324

PFAM1 PF02793

PDB1 3N95:C

AC2 Q96RP3

PFAM2 PF11613

PDB2 3N95:E

INT non-edgetic

AA1 47I;Interacts with:105I,106L,109V

AA1 68F;Interacts with:105I,106L,102N

AA1 69N;Interacts with:98Q,102N

AA1 71V;Interacts with:99A,102N,98Q

AA1 73Y;Interacts with:106L,103A,102N

AA1 92K;Interacts with:107A,109V

MUT1 K92N;dbSNP:rs148406223;dbSNP:rs148406223;

AA1 93I;Interacts with:109V

MUT1 I93S;dbSNP:rs144584262;dbSNP:rs144584262;

AA1 94N;Interacts with:109V

AA1 95Y;Interacts with:106L,109V

AA1 96S;Interacts with:106L

AA2 102N;Interacts with:71V,69N,73Y,68F

AA2 103A;Interacts with:73Y

AA2 105I;Interacts with:47I,68F

AA2 106L;Interacts with:96S,47I,73Y,95Y,68F

AA2 107A;Interacts with:92K

AA2 109V;Interacts with:47I,94N,95Y,92K,93I

AA2 98Q;Interacts with:69N,71V

AA2 99A;Interacts with:71V

//

MESH1 mesh:D001172

NAME1 Arthritis, Rheumatoid

CLUSTER1 clust1

MESH2 mesh:D009765

NAME2 Obesity

CLUSTER2 clust1

AC1 Q13324

PFAM1 PF02793

PDB1 3N93:B

AC2 Q969E3

PFAM2 PF11613

PDB2 3N93:C

INT non-edgetic

AA1 47I;Interacts with:157I,154M,153L

AA1 68F;Interacts with:154M,153L,150N

AA1 69N;Interacts with:146Q,150N

AA1 70G;Interacts with:146Q,143L

AA1 71V;Interacts with:147A,150N,143L,146Q

AA1 73Y;Interacts with:151A,147A,154M,150N

AA1 92K;Interacts with:157I,156Q,155A

MUT1 K92N;dbSNP:rs148406223;dbSNP:rs148406223;

AA1 93I;Interacts with:157I

MUT1 I93S;dbSNP:rs144584262;dbSNP:rs144584262;

AA1 95Y;Interacts with:154M,157I

AA2 143L;Interacts with:71V,70G

AA2 146Q;Interacts with:70G,69N,71V

AA2 147A;Interacts with:71V,73Y

MUT2 A147V;1KG:1127728;

AA2 150N;Interacts with:71V,69N,73Y,68F

AA2 151A;Interacts with:73Y

AA2 153L;Interacts with:68F,47I

AA2 154M;Interacts with:68F,95Y,73Y,47I

AA2 155A;Interacts with:92K

AA2 156Q;Interacts with:92K

AA2 157I;Interacts with:47I,92K,95Y,93I

//

MESH1 mesh:D001172

NAME1 Arthritis, Rheumatoid

CLUSTER1 clust1

MESH2 mesh:D009765

NAME2 Obesity

CLUSTER2 clust1

AC1 Q969J5

PFAM1 PF09294

PDB1 3G9V:C

AC2 Q9GZX6

PFAM2 PF14565

PDB2 3G9V:C

INT semi-edgetic

AA1 200E;Interacts with:175R

AA1 246P;Interacts with:50P

AA1 247M;Interacts with:54N,50P,51Y,53T,48Q

AA1 248L;Interacts with:54N,53T,50P

AA1 249D;Interacts with:54N,50P

AA1 250R;Interacts with:54N

AA2 175R;Interacts with:200E

AA2 48Q;Interacts with:247M

AA2 50P;Interacts with:247M,249D,248L,246P

AA2 51Y;Interacts with:247M

AA2 53T;Interacts with:247M,248L

AA2 54N;Interacts with:247M,248L,250R,249D

//

MESH1 mesh:D001172

NAME1 Arthritis, Rheumatoid

CLUSTER1 clust1

MESH2 mesh:D009765

NAME2 Obesity

CLUSTER2 clust1

AC1 Q969J5

PFAM1 PF01108

PDB1 3G9V:A

AC2 Q9GZX6

PFAM2 PF14565

PDB2 3G9V:A

INT semi-edgetic

AA1 100G;Interacts with:70T,71D,72V,73R

AA1 101Q;Interacts with:71D,72V,70T

AA1 102R;Interacts with:71D

AA1 103Q;Interacts with:71D

AA1 104W;Interacts with:70T,71D

AA1 128Q;Interacts with:175R,73R,176N

AA1 129E;Interacts with:73R

AA1 130P;Interacts with:73R

AA1 132Y;Interacts with:70T

AA1 65K;Interacts with:70T,71D

AA1 66I;Interacts with:73R

AA1 99Y;Interacts with:70T,169L,72V,69N,73R

AA2 169L;Interacts with:99Y

AA2 175R;Interacts with:128Q

AA2 176N;Interacts with:128Q

AA2 69N;Interacts with:99Y

AA2 70T;Interacts with:104W,99Y,132Y,100G,65K,101Q

AA2 71D;Interacts with:101Q,103Q,100G,104W,65K,102R

AA2 72V;Interacts with:99Y,100G,101Q

AA2 73R;Interacts with:129E,130P,128Q,100G,99Y,66I

MUT2 R73H;dbSNP:rs149366319;

//

MESH1 mesh:D001172

NAME1 Arthritis, Rheumatoid

CLUSTER1 clust1

MESH2 mesh:D009765

NAME2 Obesity

CLUSTER2 clust1

AC1 Q8N6P7

PFAM1 PF09294

PDB1 3DGC:R

AC2 Q9GZX6

PFAM2 PF14565

PDB2 3DGC:R

INT semi-edgetic

AA1 133R;Interacts with:116Q,117E

AA1 134S;Interacts with:124R

AA1 136Q;Interacts with:124R

AA1 162D;Interacts with:175R,48Q,44K

AA1 175Y;Interacts with:51Y

AA1 179L;Interacts with:45S

AA1 180G;Interacts with:48Q

AA1 183Q;Interacts with:45S,43D,44K

AA1 185E;Interacts with:128R,124R,43D

AA1 186Y;Interacts with:128R

AA1 187E;Interacts with:124R,49Q,128R,45S

AA1 189F;Interacts with:46N,52I,49Q,121F,124R

AA1 190G;Interacts with:51Y,52I,49Q

AA1 191L;Interacts with:117E

AA1 206P;Interacts with:48Q

AA1 207T;Interacts with:172M,175R,48Q,44K

AA1 208W;Interacts with:57F,172M

AA2 116Q;Interacts with:133R

AA2 117E;Interacts with:191L,133R

AA2 121F;Interacts with:189F

AA2 124R;Interacts with:187E,185E,189F,134S,136Q

AA2 128R;Interacts with:187E,185E,186Y

AA2 172M;Interacts with:207T,208W

MUT2 M172V;dbSNP:rs147812317;

AA2 175R;Interacts with:162D,207T

AA2 43D;Interacts with:183Q,185E

AA2 44K;Interacts with:162D,207T,183Q

AA2 45S;Interacts with:179L,183Q,187E

AA2 46N;Interacts with:189F

AA2 48Q;Interacts with:206P,162D,207T,180G

AA2 49Q;Interacts with:187E,189F,190G

AA2 51Y;Interacts with:190G,175Y

AA2 52I;Interacts with:189F,190G

AA2 57F;Interacts with:208W

//

MESH1 mesh:D001172

NAME1 Arthritis, Rheumatoid

CLUSTER1 clust1

MESH2 mesh:D009765

NAME2 Obesity

CLUSTER2 clust1

AC1 Q8N6P7

PFAM1 PF01108

PDB1 3DGC:R

AC2 Q9GZX6

PFAM2 PF14565

PDB2 3DGC:R

INT semi-edgetic

AA1 58K;Interacts with:71D,70T

MUT1 K58A;882419;

AA1 60Y;Interacts with:72V,70T,169L,73R,69N,74L

MUT1 Y60A;1368880;

MUT1 Y60R;1368881;

AA1 61G;Interacts with:73R,72V,70T,71D

AA1 62E;Interacts with:72V,70T,71D

AA1 63R;Interacts with:71D

AA1 64D;Interacts with:71D

AA1 65W;Interacts with:70T

AA1 89T;Interacts with:77E,73R

AA1 90E;Interacts with:73R

AA1 91L;Interacts with:73R,169L

MUT1 L91F;dbSNP:rs144035143;

AA1 93Y;Interacts with:70T

AA2 169L;Interacts with:60Y,91L

AA2 69N;Interacts with:60Y

AA2 70T;Interacts with:58K,61G,60Y,62E,93Y,65W

AA2 71D;Interacts with:58K,61G,63R,64D,62E

AA2 72V;Interacts with:62E,60Y,61G

AA2 73R;Interacts with:61G,90E,89T,60Y,91L

MUT2 R73H;dbSNP:rs149366319;

AA2 74L;Interacts with:60Y

AA2 77E;Interacts with:89T

//

MESH1 mesh:D001172

NAME1 Arthritis, Rheumatoid

CLUSTER1 clust1

MESH2 mesh:D011471

NAME2 Prostatic Neoplasms

CLUSTER2 clust2

AC1 O14786

PFAM1 PF00754

PDB1 4DEQ:A

AC2 P15692

PFAM2 PF00754

PDB2 4DEQ:B

INT non-edgetic

AA1 319E;Interacts with:138R

AA1 323R;Interacts with:92L,45D,90E,91G

AA1 324E;Interacts with:138R

AA2 138R;Interacts with:324E,319E

AA2 45D;Interacts with:323R

AA2 90E;Interacts with:323R

AA2 91G;Interacts with:323R

AA2 92L;Interacts with:323R

//

MESH1 mesh:D001172

NAME1 Arthritis, Rheumatoid

CLUSTER1 clust1

MESH2 mesh:D011471

NAME2 Prostatic Neoplasms

CLUSTER2 clust2

AC1 O14786

PFAM1 PF00754

PDB1 4DEQ:B

AC2 P15692

PFAM2 PF14554

PDB2 4DEQ:A

INT non-edgetic

AA1 297Y;Interacts with:231R,232R,230P

AA1 298S;Interacts with:219E,222E

AA1 299T;Interacts with:222E,219E

AA1 300N;Interacts with:210D,219E,231R

AA1 301W;Interacts with:219E,232R

AA1 316T;Interacts with:232R

AA1 320D;Interacts with:232R

AA1 348E;Interacts with:214K,231R

AA1 349T;Interacts with:231R,232R

AA1 353Y;Interacts with:232R

AA1 413T;Interacts with:232R

AA2 210D;Interacts with:300N

MUT2 D210H;971365;

AA2 214K;Interacts with:348E

AA2 219E;Interacts with:298S,301W,300N,299T

AA2 222E;Interacts with:298S,299T

AA2 230P;Interacts with:297Y

AA2 231R;Interacts with:297Y,349T,300N,348E

AA2 232R;Interacts with:320D,297Y,316T,301W,413T,349T,353Y

//

MESH1 mesh:D001172

NAME1 Arthritis, Rheumatoid

CLUSTER1 clust1

MESH2 mesh:D011471

NAME2 Prostatic Neoplasms

CLUSTER2 clust2

AC1 P29459

PFAM1 PF03039

PDB1 3HMX:B

AC2 P29460

PFAM2 PF10420

PDB2 3HMX:A

INT semi-edgetic

AA1 205R;Interacts with:203E

AA1 208T;Interacts with:203E,202A

AA1 211R;Interacts with:136Y

AA1 71H;Interacts with:205S

AA1 76K;Interacts with:205S

AA1 82V;Interacts with:201A,202A,203E

AA1 83E;Interacts with:201A,202A

AA1 85C;Interacts with:202A

AA1 86L;Interacts with:202A,200P,201A

AA2 136Y;Interacts with:211R

AA2 200P;Interacts with:86L

AA2 201A;Interacts with:82V,83E,86L

AA2 202A;Interacts with:85C,82V,86L,83E,208T

AA2 203E;Interacts with:208T,82V,205R

AA2 205S;Interacts with:71H,76K

//

MESH1 mesh:D001172

NAME1 Arthritis, Rheumatoid

CLUSTER1 clust1

MESH2 mesh:D011471

NAME2 Prostatic Neoplasms

CLUSTER2 clust2

AC1 P22301

PFAM1 PF00726

PDB1 1J7V:L

AC2 Q13651

PFAM2 PF09294

PDB2 1J7V:R

INT semi-edgetic

AA1 38P;Interacts with:212R,211S,164F,210A

AA1 39N;Interacts with:210A

AA1 41L;Interacts with:211S

AA1 42R;Interacts with:211S,214N,208S,212R,166E,210A,213S

AA1 43D;Interacts with:211S

AA1 45R;Interacts with:211S

AA1 46D;Interacts with:213S

AA2 164F;Interacts with:38P

AA2 166E;Interacts with:42R

AA2 208S;Interacts with:42R

AA2 210A;Interacts with:39N,42R,38P

AA2 211S;Interacts with:42R,38P,41L,45R,43D

AA2 212R;Interacts with:38P,42R

AA2 213S;Interacts with:46D,42R

AA2 214N;Interacts with:42R

//

MESH1 mesh:D001172

NAME1 Arthritis, Rheumatoid

CLUSTER1 clust1

MESH2 mesh:D011471

NAME2 Prostatic Neoplasms

CLUSTER2 clust2

AC1 P22301

PFAM1 PF00726

PDB1 1J7V:L

AC2 Q13651

PFAM2 PF01108

PDB2 1J7V:R

INT semi-edgetic

AA1 56Q;Interacts with:97R

AA1 59D;Interacts with:97R

AA1 60Q;Interacts with:97R

MUT1 Q60E;PMID:10637267;

AA1 61L;Interacts with:97R,64Y

AA1 62D;Interacts with:69W,66I,65G,67E,97R,64Y

AA1 63N;Interacts with:67E,65G,64Y

AA1 64L;Interacts with:64Y,67E,65G

AA1 65L;Interacts with:64Y

AA1 66L;Interacts with:94N

AA2 64Y;Interacts with:65L,64L,61L,62D,63N

AA2 65G;Interacts with:62D,63N,64L

AA2 66I;Interacts with:62D

AA2 67E;Interacts with:63N,62D,64L

AA2 69W;Interacts with:62D

AA2 94N;Interacts with:66L

AA2 97R;Interacts with:60Q,61L,59D,62D,56Q

//

MESH1 mesh:D001172

NAME1 Arthritis, Rheumatoid

CLUSTER1 clust1

MESH2 mesh:D011471

NAME2 Prostatic Neoplasms

CLUSTER2 clust2

AC1 P28068

PFAM1 PF07654

PDB1 4I0P:B

AC2 Q6ICR9

PFAM2 PF00993

PDB2 4I0P:A

INT semi-edgetic

AA1 141Y;Interacts with:63Y

AA1 168P;Interacts with:65E

AA1 169N;Interacts with:66D,81R

AA1 170G;Interacts with:66D,81R

AA1 171D;Interacts with:66D,82L

AA1 172W;Interacts with:66D,68L,63Y,85F,67Q,82L

AA1 173T;Interacts with:66D

AA1 174Y;Interacts with:65E,66D

AA2 63Y;Interacts with:172W,141Y

AA2 65E;Interacts with:174Y,168P

AA2 66D;Interacts with:172W,173T,174Y,169N,170G,171D

AA2 67Q;Interacts with:172W

AA2 68L;Interacts with:172W

AA2 81R;Interacts with:170G,169N

AA2 82L;Interacts with:171D,172W

AA2 85F;Interacts with:172W

//

MESH1 mesh:D001172

NAME1 Arthritis, Rheumatoid

CLUSTER1 clust1

MESH2 mesh:D011471

NAME2 Prostatic Neoplasms

CLUSTER2 clust2

AC1 P15813

PFAM1 PF07654

PDB1 1ZT4:A

AC2 P61769

PFAM2 PF07654

PDB2 1ZT4:B

INT edgetic

AA1 208W;Interacts with:34P,32R,33H

AA1 227S;Interacts with:32R,33H

AA1 228G;Interacts with:32R

AA1 254L;Interacts with:46Y

AA1 255P;Interacts with:44N,85L

AA1 256N;Interacts with:85L,32R,44N,31S

AA1 257A;Interacts with:44N,87Y,85L,42F

AA1 258D;Interacts with:32R

AA1 260T;Interacts with:32R

AA1 261W;Interacts with:32R

AA1 262Y;Interacts with:31S

AA2 31S;Interacts with:262Y,256N

AA2 32R;Interacts with:260T,258D,227S,256N,261W,228G,208W

MUT2 R32C;dbSNP:rs11553032;

AA2 33H;Interacts with:227S,208W

AA2 34P;Interacts with:208W

MUT2 P34S;dbSNP:rs11553035;

AA2 42F;Interacts with:257A

MUT2 F42L;dbSNP:rs11553038;

AA2 44N;Interacts with:257A,255P,256N

AA2 46Y;Interacts with:254L

AA2 85L;Interacts with:256N,255P,257A

AA2 87Y;Interacts with:257A

//

MESH1 mesh:D001172

NAME1 Arthritis, Rheumatoid

CLUSTER1 clust1

MESH2 mesh:D011658

NAME2 Pulmonary Fibrosis

CLUSTER2 clust1

AC1 P01375

PFAM1 PF00229

PDB1 3ALQ:A

AC2 P20333

PFAM2 PF00020

PDB2 3ALQ:R

INT semi-edgetic

AA1 107R;Interacts with:93C,92E

MUT1 R107P;PMID:1331108;

MUT1 R107K;PMID:8253759;

MUT1 R107H;PMID:2217144,PMID:1331108;

AA1 108R;Interacts with:95S,93C,81S,92E,80D,94L,82T,79E

MUT1 R108V;PMID;

MUT1 R108W;963560;clinvar:27425, phenotype=TNF RECEPTOR BINDING, ALTERED;

MUT1 R108E;PMID:1715560;

MUT1 R108Q;PMID:2217144;

AA1 109A;Interacts with:94L,92E,90V,93C

MUT1 A109T;PMID:2217144,PMID:1715560,PMID:8253759;

AA1 143Q;Interacts with:102S,101S,103D

MUT1 Q143E;PMID:MEDLINE;

AA1 161V;Interacts with:89W

AA1 162S;Interacts with:89W,88N,90V

MUT1 S162L;PMID:2009860;

MUT1 S162F;963563;

AA1 163Y;Interacts with:85Q,94L,90V,84T,86L,88N,87W

MUT1 Y163H;PMID:2217144,PMID:1715560,PMID:1331108;

AA1 164Q;Interacts with:86L,87W

AA1 165T;Interacts with:86L

AA1 167V;Interacts with:94L

MUT1 V167I;PMID:2217144,PMID:1715560;

MUT1 V167A;1026230;

MUT1 V167D;1026231;

AA1 203E;Interacts with:89W

AA1 220F;Interacts with:98S

MUT1 F220Y;PMID:2217144,PMID:8253759;

AA1 221A;Interacts with:98S,97G

AA1 222E;Interacts with:98S

MUT1 E222K;963564;

AA2 101S;Interacts with:143Q

AA2 102S;Interacts with:143Q

MUT2 S102P;963767;

AA2 103D;Interacts with:143Q

AA2 79E;Interacts with:108R

AA2 80D;Interacts with:108R

AA2 81S;Interacts with:108R

AA2 82T;Interacts with:108R

AA2 84T;Interacts with:163Y

AA2 85Q;Interacts with:163Y

AA2 86L;Interacts with:165T,164Q,163Y

AA2 87W;Interacts with:163Y,164Q

AA2 88N;Interacts with:162S,163Y

AA2 89W;Interacts with:161V,162S,203E

AA2 90V;Interacts with:109A,162S,163Y

AA2 92E;Interacts with:108R,109A,107R

AA2 93C;Interacts with:108R,107R,109A

AA2 94L;Interacts with:109A,108R,163Y,167V

AA2 95S;Interacts with:108R

AA2 97G;Interacts with:221A

AA2 98S;Interacts with:221A,220F,222E

MUT2 S98P;963766;

//

MESH1 mesh:D001172

NAME1 Arthritis, Rheumatoid

CLUSTER1 clust1

MESH2 mesh:D011658

NAME2 Pulmonary Fibrosis

CLUSTER2 clust1

AC1 O14786

PFAM1 PF00754

PDB1 4DEQ:A

AC2 P15692

PFAM2 PF00754

PDB2 4DEQ:B

INT semi-edgetic

AA1 319E;Interacts with:138R

AA1 323R;Interacts with:92L,45D,90E,91G

AA1 324E;Interacts with:138R

AA2 138R;Interacts with:324E,319E

AA2 45D;Interacts with:323R

AA2 90E;Interacts with:323R

AA2 91G;Interacts with:323R

AA2 92L;Interacts with:323R

//

MESH1 mesh:D001172

NAME1 Arthritis, Rheumatoid

CLUSTER1 clust1

MESH2 mesh:D011658

NAME2 Pulmonary Fibrosis

CLUSTER2 clust1

AC1 O14786

PFAM1 PF00754

PDB1 4DEQ:B

AC2 P15692

PFAM2 PF14554

PDB2 4DEQ:A

INT semi-edgetic

AA1 297Y;Interacts with:231R,232R,230P

AA1 298S;Interacts with:219E,222E

AA1 299T;Interacts with:222E,219E

AA1 300N;Interacts with:210D,219E,231R

AA1 301W;Interacts with:219E,232R

AA1 316T;Interacts with:232R

AA1 320D;Interacts with:232R

AA1 348E;Interacts with:214K,231R

AA1 349T;Interacts with:231R,232R

AA1 353Y;Interacts with:232R

AA1 413T;Interacts with:232R

AA2 210D;Interacts with:300N

MUT2 D210H;971365;

AA2 214K;Interacts with:348E

AA2 219E;Interacts with:298S,301W,300N,299T

AA2 222E;Interacts with:298S,299T

AA2 230P;Interacts with:297Y

AA2 231R;Interacts with:297Y,349T,300N,348E

AA2 232R;Interacts with:320D,297Y,316T,301W,413T,349T,353Y

//

MESH1 mesh:D001172

NAME1 Arthritis, Rheumatoid

CLUSTER1 clust1

MESH2 mesh:D012130

NAME2 Respiratory Hypersensitivity

CLUSTER2 clust1

AC1 P18510

PFAM1 PF00340

PDB1 1IRA:X

AC2 P14778

PFAM2 PF13895

PDB2 1IRA:Y

INT semi-edgetic

AA1 151E;Interacts with:146E

MUT1 E151G;PMID:7744786;

AA1 152A;Interacts with:146E,144Y

AA1 172Y;Interacts with:139G,132L

AA1 60L;Interacts with:128F,129K,144Y

AA1 61Q;Interacts with:127I,126A,125Q,128F

AA1 62G;Interacts with:125Q,127I

AA1 63P;Interacts with:125Q

AA1 68E;Interacts with:131K

AA2 125Q;Interacts with:63P,62G,61Q

MUT2 Q125K;dbSNP:rs76949245;

AA2 126A;Interacts with:61Q

AA2 127I;Interacts with:61Q,62G

AA2 128F;Interacts with:60L,61Q

AA2 129K;Interacts with:60L

AA2 131K;Interacts with:68E

AA2 132L;Interacts with:172Y

AA2 139G;Interacts with:172Y

AA2 144Y;Interacts with:152A,60L

AA2 146E;Interacts with:152A,151E

//

MESH1 mesh:D001172

NAME1 Arthritis, Rheumatoid

CLUSTER1 clust1

MESH2 mesh:D014376

NAME2 Tuberculosis

CLUSTER2 clust1

AC1 P29459

PFAM1 PF03039

PDB1 3HMX:B

AC2 P29460

PFAM2 PF10420

PDB2 3HMX:A

INT non-edgetic

AA1 205R;Interacts with:203E

AA1 208T;Interacts with:203E,202A

AA1 211R;Interacts with:136Y

AA1 71H;Interacts with:205S

AA1 76K;Interacts with:205S

AA1 82V;Interacts with:201A,202A,203E

AA1 83E;Interacts with:201A,202A

AA1 85C;Interacts with:202A

AA1 86L;Interacts with:202A,200P,201A

AA2 136Y;Interacts with:211R

AA2 200P;Interacts with:86L

AA2 201A;Interacts with:82V,83E,86L

AA2 202A;Interacts with:85C,82V,86L,83E,208T

AA2 203E;Interacts with:208T,82V,205R

AA2 205S;Interacts with:71H,76K

//

MESH1 mesh:D001172

NAME1 Arthritis, Rheumatoid

CLUSTER1 clust1

MESH2 mesh:D014376

NAME2 Tuberculosis

CLUSTER2 clust1

AC1 P01375

PFAM1 PF00229

PDB1 3ALQ:A

AC2 P20333

PFAM2 PF00020

PDB2 3ALQ:R

INT semi-edgetic

AA1 107R;Interacts with:93C,92E

MUT1 R107P;PMID:1331108;

MUT1 R107K;PMID:8253759;

MUT1 R107H;PMID:2217144,PMID:1331108;

AA1 108R;Interacts with:95S,93C,81S,92E,80D,94L,82T,79E

MUT1 R108V;PMID;

MUT1 R108W;963560;clinvar:27425, phenotype=TNF RECEPTOR BINDING, ALTERED;

MUT1 R108E;PMID:1715560;

MUT1 R108Q;PMID:2217144;

AA1 109A;Interacts with:94L,92E,90V,93C

MUT1 A109T;PMID:2217144,PMID:1715560,PMID:8253759;

AA1 143Q;Interacts with:102S,101S,103D

MUT1 Q143E;PMID:MEDLINE;

AA1 161V;Interacts with:89W

AA1 162S;Interacts with:89W,88N,90V

MUT1 S162L;PMID:2009860;

MUT1 S162F;963563;

AA1 163Y;Interacts with:85Q,94L,90V,84T,86L,88N,87W

MUT1 Y163H;PMID:2217144,PMID:1715560,PMID:1331108;

AA1 164Q;Interacts with:86L,87W

AA1 165T;Interacts with:86L

AA1 167V;Interacts with:94L

MUT1 V167I;PMID:2217144,PMID:1715560;

MUT1 V167A;1026230;

MUT1 V167D;1026231;

AA1 203E;Interacts with:89W

AA1 220F;Interacts with:98S

MUT1 F220Y;PMID:2217144,PMID:8253759;

AA1 221A;Interacts with:98S,97G

AA1 222E;Interacts with:98S

MUT1 E222K;963564;

AA2 101S;Interacts with:143Q

AA2 102S;Interacts with:143Q

MUT2 S102P;963767;

AA2 103D;Interacts with:143Q

AA2 79E;Interacts with:108R

AA2 80D;Interacts with:108R

AA2 81S;Interacts with:108R

AA2 82T;Interacts with:108R

AA2 84T;Interacts with:163Y

AA2 85Q;Interacts with:163Y

AA2 86L;Interacts with:165T,164Q,163Y

AA2 87W;Interacts with:163Y,164Q

AA2 88N;Interacts with:162S,163Y

AA2 89W;Interacts with:161V,162S,203E

AA2 90V;Interacts with:109A,162S,163Y

AA2 92E;Interacts with:108R,109A,107R

AA2 93C;Interacts with:108R,107R,109A

AA2 94L;Interacts with:109A,108R,163Y,167V

AA2 95S;Interacts with:108R

AA2 97G;Interacts with:221A

AA2 98S;Interacts with:221A,220F,222E

MUT2 S98P;963766;

//

MESH1 mesh:D001172

NAME1 Arthritis, Rheumatoid

CLUSTER1 clust1

MESH2 mesh:D014376

NAME2 Tuberculosis

CLUSTER2 clust1

AC1 P18510

PFAM1 PF00340

PDB1 1IRA:X

AC2 P14778

PFAM2 PF13895

PDB2 1IRA:Y

INT semi-edgetic

AA1 151E;Interacts with:146E

MUT1 E151G;PMID:7744786;

AA1 152A;Interacts with:146E,144Y

AA1 172Y;Interacts with:139G,132L

AA1 60L;Interacts with:128F,129K,144Y

AA1 61Q;Interacts with:127I,126A,125Q,128F

AA1 62G;Interacts with:125Q,127I

AA1 63P;Interacts with:125Q

AA1 68E;Interacts with:131K

AA2 125Q;Interacts with:63P,62G,61Q

MUT2 Q125K;dbSNP:rs76949245;

AA2 126A;Interacts with:61Q

AA2 127I;Interacts with:61Q,62G

AA2 128F;Interacts with:60L,61Q

AA2 129K;Interacts with:60L

AA2 131K;Interacts with:68E

AA2 132L;Interacts with:172Y

AA2 139G;Interacts with:172Y

AA2 144Y;Interacts with:152A,60L

AA2 146E;Interacts with:152A,151E

//

MESH1 mesh:D001172

NAME1 Arthritis, Rheumatoid

CLUSTER1 clust1

MESH2 mesh:D014376

NAME2 Tuberculosis

CLUSTER2 clust1

AC1 P01579

PFAM1 PF00714

PDB1 1FYH:A

AC2 P15260

PFAM2 PF07140

PDB2 1FYH:E

INT semi-edgetic

AA1 146A;Interacts with:186K,203A,205P,185Y

AA1 147A;Interacts with:183I,185Y

MUT1 A147L;PMID:1662603;

AA1 149T;Interacts with:185Y

AA1 150G;Interacts with:208S,205P,207S

AA1 151K;Interacts with:209L,207S,208S

MUT1 K151R;PMID:2127102;

MUT1 K151E;PMID:1830392;

AA2 183I;Interacts with:147A

AA2 185Y;Interacts with:146A,147A,149T

AA2 186K;Interacts with:146A

AA2 203A;Interacts with:146A

AA2 205P;Interacts with:150G,146A

AA2 207S;Interacts with:151K,150G

AA2 208S;Interacts with:150G,151K

AA2 209L;Interacts with:151K

//

MESH1 mesh:D001172

NAME1 Arthritis, Rheumatoid

CLUSTER1 clust1

MESH2 mesh:D014376

NAME2 Tuberculosis

CLUSTER2 clust1

AC1 P01579

PFAM1 PF00714

PDB1 1FYH:A

AC2 P15260

PFAM2 PF01108

PDB2 1FYH:B

INT semi-edgetic

AA1 163R;Interacts with:66Y

AA2 66Y;Interacts with:163R

MUT2 Y66A;PMID:9878445;

MUT2 Y66F;PMID:9878445;

//

MESH1 mesh:D001172

NAME1 Arthritis, Rheumatoid

CLUSTER1 clust1

MESH2 mesh:D016889

NAME2 Endometrial Neoplasms

CLUSTER2 clust5

AC1 P01375

PFAM1 PF00229

PDB1 3ALQ:A

AC2 P20333

PFAM2 PF00020

PDB2 3ALQ:R

INT semi-edgetic

AA1 107R;Interacts with:93C,92E

MUT1 R107P;PMID:1331108;

MUT1 R107K;PMID:8253759;

MUT1 R107H;PMID:2217144,PMID:1331108;

AA1 108R;Interacts with:95S,93C,81S,92E,80D,94L,82T,79E

MUT1 R108V;PMID;

MUT1 R108W;963560;clinvar:27425, phenotype=TNF RECEPTOR BINDING, ALTERED;

MUT1 R108E;PMID:1715560;

MUT1 R108Q;PMID:2217144;

AA1 109A;Interacts with:94L,92E,90V,93C

MUT1 A109T;PMID:2217144,PMID:1715560,PMID:8253759;

AA1 143Q;Interacts with:102S,101S,103D

MUT1 Q143E;PMID:MEDLINE;

AA1 161V;Interacts with:89W

AA1 162S;Interacts with:89W,88N,90V

MUT1 S162L;PMID:2009860;

MUT1 S162F;963563;

AA1 163Y;Interacts with:85Q,94L,90V,84T,86L,88N,87W

MUT1 Y163H;PMID:2217144,PMID:1715560,PMID:1331108;

AA1 164Q;Interacts with:86L,87W

AA1 165T;Interacts with:86L

AA1 167V;Interacts with:94L

MUT1 V167I;PMID:2217144,PMID:1715560;

MUT1 V167A;1026230;

MUT1 V167D;1026231;

AA1 203E;Interacts with:89W

AA1 220F;Interacts with:98S

MUT1 F220Y;PMID:2217144,PMID:8253759;

AA1 221A;Interacts with:98S,97G

AA1 222E;Interacts with:98S

MUT1 E222K;963564;

AA2 101S;Interacts with:143Q

AA2 102S;Interacts with:143Q

MUT2 S102P;963767;

AA2 103D;Interacts with:143Q

AA2 79E;Interacts with:108R

AA2 80D;Interacts with:108R

AA2 81S;Interacts with:108R

AA2 82T;Interacts with:108R

AA2 84T;Interacts with:163Y

AA2 85Q;Interacts with:163Y

AA2 86L;Interacts with:165T,164Q,163Y

AA2 87W;Interacts with:163Y,164Q

AA2 88N;Interacts with:162S,163Y

AA2 89W;Interacts with:161V,162S,203E

AA2 90V;Interacts with:109A,162S,163Y

AA2 92E;Interacts with:108R,109A,107R

AA2 93C;Interacts with:108R,107R,109A

AA2 94L;Interacts with:109A,108R,163Y,167V

AA2 95S;Interacts with:108R

AA2 97G;Interacts with:221A

AA2 98S;Interacts with:221A,220F,222E

MUT2 S98P;963766;

//

MESH1 mesh:D001172

NAME1 Arthritis, Rheumatoid

CLUSTER1 clust1

MESH2 mesh:D029424

NAME2 Pulmonary Disease, Chronic Obstructive

CLUSTER2 clust1

AC1 P03956

PFAM1 PF00413

PDB1 2J0T:A

AC2 P01033

PFAM2 PF00965

PDB2 2J0T:D

INT semi-edgetic

AA1 171N;Interacts with:58Y,56T

AA1 172S;Interacts with:58Y,92V

AA1 173P;Interacts with:58Y

AA1 175D;Interacts with:111K

AA1 178G;Interacts with:27V

AA1 179G;Interacts with:26C,27V

AA1 180N;Interacts with:25T,26C,120T,92V

AA1 181L;Interacts with:26C,25T,27V

AA1 182A;Interacts with:24C,25T

AA1 183H;Interacts with:24C,91S,92V

AA1 184A;Interacts with:92V,91S

AA1 185F;Interacts with:92V,91S,57L

AA1 186Q;Interacts with:91S

AA1 191I;Interacts with:57L

MUT1 I191V;171135;humsavar:VAR_021024;rs17879973;

AA1 210Y;Interacts with:27V

MUT1 Y210T;PMID:10871619;

AA1 214R;Interacts with:25T

MUT1 R214H;COSMIC:236794;

AA1 215V;Interacts with:25T

AA1 218H;Interacts with:25T,24C

MUT1 H218S;PMID:7929334;

AA1 219E;Interacts with:25T,24C

AA1 222H;Interacts with:24C,91S,90E

AA1 227S;Interacts with:90E

AA1 228H;Interacts with:24C,121T,90E

AA1 230T;Interacts with:98R

AA1 238P;Interacts with:25T,24C,26C

AA1 239S;Interacts with:24C,26C,25T,28P

AA1 240Y;Interacts with:26C,25T,28P

AA2 111K;Interacts with:175D

AA2 120T;Interacts with:180N

AA2 121T;Interacts with:228H

AA2 24C;Interacts with:222H,238P,183H,239S,182A,228H,218H,219E

MUT2 C24S;PMID:9774703;

AA2 25T;Interacts with:180N,238P,218H,219E,182A,214R,181L,215V,239S,240Y

MUT2 T25Q;1375658;

MUT2 T25K;1375657;

MUT2 T25V;961993;

MUT2 T25G;1375656;

MUT2 T25E;1375655;

MUT2 T25R;1375659;

AA2 26C;Interacts with:238P,179G,181L,239S,180N,240Y

AA2 27V;Interacts with:178G,179G,210Y,181L

AA2 28P;Interacts with:239S,240Y

MUT2 P28A;PMID:9774703;

AA2 56T;Interacts with:171N

AA2 57L;Interacts with:185F,191I

AA2 58Y;Interacts with:171N,172S,173P

AA2 90E;Interacts with:227S,222H,228H

AA2 91S;Interacts with:184A,222H,186Q,185F,183H

AA2 92V;Interacts with:184A,185F,183H,180N,172S

AA2 98R;Interacts with:230T

//

MESH1 mesh:D001172

NAME1 Arthritis, Rheumatoid

CLUSTER1 clust1

MESH2 mesh:D029424

NAME2 Pulmonary Disease, Chronic Obstructive

CLUSTER2 clust1

AC1 P08254

PFAM1 PF00413

PDB1 1OO9:A

AC2 P01033

PFAM2 PF00965

PDB2 1OO9:B

INT semi-edgetic

AA1 171F;Interacts with:57L

AA1 172Y;Interacts with:58Y,87P,92V,88A,57L,89M

AA1 178G;Interacts with:27V

AA1 179N;Interacts with:26C,29P,28P,27V,122C,120T

AA1 180V;Interacts with:120T,26C,27V,25T

AA1 181L;Interacts with:26C,27V,25T

AA1 182A;Interacts with:24C,25T

AA1 183H;Interacts with:24C,92V,91S

AA1 184A;Interacts with:91S,92V,24C

AA1 185Y;Interacts with:92V,91S

AA1 186A;Interacts with:89M,91S

AA1 207T;Interacts with:157S,158I,156L

AA1 208T;Interacts with:156L,157S

AA1 209G;Interacts with:156L,157S

AA1 210T;Interacts with:156L

MUT1 T210Y;PMID:10871619;

AA1 215V;Interacts with:25T

AA1 218H;Interacts with:25T,24C

AA1 219E;Interacts with:25T,24C

AA1 222H;Interacts with:90E,24C,91S

AA1 227F;Interacts with:89M

AA1 228H;Interacts with:91S,24C,121T,90E,94G

AA1 229S;Interacts with:90E

AA1 238P;Interacts with:27V,24C,25T,26C

AA1 239L;Interacts with:26C,121T,25T,28P

AA1 240Y;Interacts with:27V,25T,26C,156L,173Q

AA1 241H;Interacts with:173Q,179E,177G,176Q

AA1 242S;Interacts with:173Q

AA1 244T;Interacts with:176Q

AA1 245D;Interacts with:176Q

AA2 120T;Interacts with:180V,179N

AA2 121T;Interacts with:239L,228H

AA2 122C;Interacts with:179N

AA2 156L;Interacts with:240Y,209G,208T,210T,207T

AA2 157S;Interacts with:209G,207T,208T

AA2 158I;Interacts with:207T

MUT2 I158V;dbSNP:rs1803571;

AA2 173Q;Interacts with:241H,242S,240Y

AA2 176Q;Interacts with:241H,245D,244T

AA2 177G;Interacts with:241H

AA2 179E;Interacts with:241H

AA2 24C;Interacts with:222H,228H,238P,182A,183H,218H,219E,184A

MUT2 C24S;PMID:9774703;

AA2 25T;Interacts with:240Y,218H,181L,238P,215V,182A,239L,219E,180V

MUT2 T25Q;1375658;

MUT2 T25K;1375657;

MUT2 T25V;961993;

MUT2 T25G;1375656;

MUT2 T25E;1375655;

MUT2 T25R;1375659;

AA2 26C;Interacts with:179N,239L,181L,180V,238P,240Y

AA2 27V;Interacts with:240Y,238P,178G,181L,179N,180V

AA2 28P;Interacts with:179N,239L

MUT2 P28A;PMID:9774703;

AA2 29P;Interacts with:179N

AA2 57L;Interacts with:171F,172Y

AA2 58Y;Interacts with:172Y

AA2 87P;Interacts with:172Y

AA2 88A;Interacts with:172Y

AA2 89M;Interacts with:186A,172Y,227F

AA2 90E;Interacts with:222H,228H,229S

AA2 91S;Interacts with:228H,184A,222H,186A,185Y,183H

AA2 92V;Interacts with:185Y,172Y,183H,184A

AA2 94G;Interacts with:228H

//

MESH1 mesh:D001172

NAME1 Arthritis, Rheumatoid

CLUSTER1 clust1

MESH2 mesh:D029424

NAME2 Pulmonary Disease, Chronic Obstructive

CLUSTER2 clust1

AC1 P08253

PFAM1 PF00045

PDB1 1GXD:A

AC2 P16035

PFAM2 PF00965

PDB2 1GXD:C

INT semi-edgetic

AA1 607A;Interacts with:191F

AA1 609A;Interacts with:196R

AA1 611N;Interacts with:191F,196R,203W

AA1 612A;Interacts with:203W,186H,191F,175M

AA1 615D;Interacts with:186H

AA1 633K;Interacts with:179T

AA1 636Y;Interacts with:162I,179T,178V,181K

AA1 638L;Interacts with:173L,175M

AA1 644S;Interacts with:196R

MUT1 S644I;901990;humsavar:VAR_036138, phenotype=A colorectal cancer sample;

AA1 645L;Interacts with:196R

AA1 646K;Interacts with:169P,198D,200S,202A,201C,196R,166I

AA1 647S;Interacts with:173L,203W,166I

AA1 648V;Interacts with:173L,165Y,203W,166I

AA1 649K;Interacts with:173L,165Y,164C

AA1 650F;Interacts with:178V,175M,173L,164C,163P,162I

AA1 651G;Interacts with:164C,162I,163P

AA1 652S;Interacts with:162I

AA2 162I;Interacts with:652S,636Y,651G,650F

AA2 163P;Interacts with:651G,650F

AA2 164C;Interacts with:651G,650F,649K

AA2 165Y;Interacts with:649K,648V

AA2 166I;Interacts with:648V,646K,647S

AA2 169P;Interacts with:646K

AA2 173L;Interacts with:647S,648V,638L,650F,649K

AA2 175M;Interacts with:650F,638L,612A

MUT2 M175T;962024;

AA2 178V;Interacts with:650F,636Y

AA2 179T;Interacts with:636Y,633K

AA2 181K;Interacts with:636Y

AA2 186H;Interacts with:615D,612A

AA2 191F;Interacts with:611N,612A,607A

AA2 196R;Interacts with:611N,609A,645L,646K,644S

AA2 198D;Interacts with:646K

AA2 200S;Interacts with:646K

AA2 201C;Interacts with:646K

AA2 202A;Interacts with:646K

AA2 203W;Interacts with:612A,611N,647S,648V

//

MESH1 mesh:D001172

NAME1 Arthritis, Rheumatoid

CLUSTER1 clust1

MESH2 mesh:D029424

NAME2 Pulmonary Disease, Chronic Obstructive

CLUSTER2 clust1

AC1 P15813

PFAM1 PF07654

PDB1 1ZT4:A

AC2 P61769

PFAM2 PF07654

PDB2 1ZT4:B

INT edgetic

AA1 208W;Interacts with:34P,32R,33H

AA1 227S;Interacts with:32R,33H

AA1 228G;Interacts with:32R

AA1 254L;Interacts with:46Y

AA1 255P;Interacts with:44N,85L

AA1 256N;Interacts with:85L,32R,44N,31S

AA1 257A;Interacts with:44N,87Y,85L,42F

AA1 258D;Interacts with:32R

AA1 260T;Interacts with:32R

AA1 261W;Interacts with:32R

AA1 262Y;Interacts with:31S

AA2 31S;Interacts with:262Y,256N

AA2 32R;Interacts with:260T,258D,227S,256N,261W,228G,208W

MUT2 R32C;dbSNP:rs11553032;

AA2 33H;Interacts with:227S,208W

AA2 34P;Interacts with:208W

MUT2 P34S;dbSNP:rs11553035;

AA2 42F;Interacts with:257A

MUT2 F42L;dbSNP:rs11553038;

AA2 44N;Interacts with:257A,255P,256N

AA2 46Y;Interacts with:254L

AA2 85L;Interacts with:256N,255P,257A

AA2 87Y;Interacts with:257A

//

MESH1 mesh:D001249

NAME1 Asthma

CLUSTER1 clust1

MESH2 mesh:D001943

NAME2 Breast Neoplasms

CLUSTER2 clust8

AC1 P29459

PFAM1 PF03039

PDB1 3HMX:B

AC2 P29460

PFAM2 PF10420

PDB2 3HMX:A

INT semi-edgetic

AA1 205R;Interacts with:203E

AA1 208T;Interacts with:203E,202A

AA1 211R;Interacts with:136Y

AA1 71H;Interacts with:205S

AA1 76K;Interacts with:205S

AA1 82V;Interacts with:201A,202A,203E

AA1 83E;Interacts with:201A,202A

AA1 85C;Interacts with:202A

AA1 86L;Interacts with:202A,200P,201A

AA2 136Y;Interacts with:211R

AA2 200P;Interacts with:86L

AA2 201A;Interacts with:82V,83E,86L

AA2 202A;Interacts with:85C,82V,86L,83E,208T

AA2 203E;Interacts with:208T,82V,205R

AA2 205S;Interacts with:71H,76K

//

MESH1 mesh:D001249

NAME1 Asthma

CLUSTER1 clust1

MESH2 mesh:D001943

NAME2 Breast Neoplasms

CLUSTER2 clust8

AC1 P08887

PFAM1 PF09240

PDB1 1P9M:C

AC2 P05231

PFAM2 PF00489

PDB2 1P9M:C

INT edgetic

AA1 155N;Interacts with:89N,94K

AA1 182E;Interacts with:97E,102F,207R

AA1 183G;Interacts with:102F

AA1 185S;Interacts with:94K

AA1 187F;Interacts with:82K

AA1 209Q;Interacts with:82K

AA1 211C;Interacts with:82K

MUT1 C211A;884560;

AA1 212G;Interacts with:82K

AA2 102F;Interacts with:183G,182E

AA2 207R;Interacts with:182E

MUT2 R207S;PMID:8436132,PMID:8483922;

MUT2 R207A;PMID:8436132,PMID:8483922;

MUT2 R207P;PMID:8436132,PMID:8483922;

MUT2 R207G;PMID:8436132,PMID:8483922;

MUT2 R207H;PMID:8436132;

MUT2 R207K;PMID:1321818,PMID:8436132,PMID:8483922;

MUT2 R207N;PMID:8436132,PMID:8483922;

MUT2 R207W;PMID:8436132;

AA2 82K;Interacts with:212G,209Q,211C,187F

AA2 89N;Interacts with:155N

AA2 94K;Interacts with:185S,155N

AA2 97E;Interacts with:182E

//

MESH1 mesh:D001249

NAME1 Asthma

CLUSTER1 clust1

MESH2 mesh:D003093

NAME2 Colitis, Ulcerative

CLUSTER2 clust1

AC1 P29459

PFAM1 PF03039

PDB1 3HMX:B

AC2 P29460

PFAM2 PF10420

PDB2 3HMX:A

INT non-edgetic

AA1 205R;Interacts with:203E

AA1 208T;Interacts with:203E,202A

AA1 211R;Interacts with:136Y

AA1 71H;Interacts with:205S

AA1 76K;Interacts with:205S

AA1 82V;Interacts with:201A,202A,203E

AA1 83E;Interacts with:201A,202A

AA1 85C;Interacts with:202A

AA1 86L;Interacts with:202A,200P,201A

AA2 136Y;Interacts with:211R

AA2 200P;Interacts with:86L

AA2 201A;Interacts with:82V,83E,86L

AA2 202A;Interacts with:85C,82V,86L,83E,208T

AA2 203E;Interacts with:208T,82V,205R

AA2 205S;Interacts with:71H,76K

//

MESH1 mesh:D001249

NAME1 Asthma

CLUSTER1 clust1

MESH2 mesh:D003093

NAME2 Colitis, Ulcerative

CLUSTER2 clust1

AC1 P01563

PFAM1 PF00143

PDB1 2HYM:B

AC2 P48551

PFAM2 PF01108

PDB2 2HYM:A

INT non-edgetic

AA1 145Y;Interacts with:75K

AA1 164E;Interacts with:73M

AA1 165V;Interacts with:73M

AA1 168A;Interacts with:73M,74S,105A

MUT1 A168T;dbSNP:rs143535431;

MUT1 A168G;PMID:11698684;

MUT1 A168M;PMID:10984492;

AA1 169E;Interacts with:73M,75K,74S

AA1 171M;Interacts with:73M

MUT1 M171A;PMID:10984492;PMID:11698684;

MUT1 M171I;dbSNP:rs77047588;

AA1 172R;Interacts with:104E,103H,74S,101S

AA1 175S;Interacts with:103H,104E

MUT1 S175P;dbSNP:rs138547087;

AA1 176L;Interacts with:103H

AA1 178T;Interacts with:103H

AA1 179N;Interacts with:103H,102T

AA1 182E;Interacts with:103H

AA1 185R;Interacts with:96T,99W,102T,100R,97D

AA1 186S;Interacts with:100R

AA1 48S;Interacts with:107V

MUT1 S48A;PMID:10984492;PMID:10556041;

AA1 49L;Interacts with:76P,73M,74S,107V,105A

MUT1 L49A;PMID:10984492;PMID:11698684,PMID:10556041;

AA1 50F;Interacts with:107V,79L,109V,71T,108T,70Y

AA1 52C;Interacts with:76P,77E

AA1 53L;Interacts with:79L,76P,77E,107V,71T,73M

MUT1 L53I;PMID:3081003;

MUT1 L53S;dbSNP:rs145655120;

AA1 54K;Interacts with:77E

AA1 55D;Interacts with:77E

MUT1 D55A;PMID:10984492;PMID:3081003,PMID:10556041;

AA1 56R;Interacts with:77E,75K,78D,76P,73M,71T,72I

MUT1 R56K;PMID:10984492;PMID:3081003;

AA1 57H;Interacts with:75K,77E,73M,74S

MUT1 H57R;883731;dbSNP:rs73420190;humsavar:VAR_013001;

AA1 58D;Interacts with:74S,75K,77E

AA1 59F;Interacts with:74S

MUT1 F59S;dbSNP:rs147918701;

AA2 100R;Interacts with:186S,185R

AA2 101S;Interacts with:172R

AA2 102T;Interacts with:185R,179N

AA2 103H;Interacts with:176L,175S,172R,178T,179N,182E

AA2 104E;Interacts with:172R,175S

AA2 105A;Interacts with:49L,168A

AA2 107V;Interacts with:50F,49L,48S,53L

AA2 108T;Interacts with:50F

AA2 109V;Interacts with:50F

MUT2 V109I;dbSNP:rs140084698;dbSNP:rs140084698;

AA2 70Y;Interacts with:50F

AA2 71T;Interacts with:50F,56R,53L

MUT2 T71A;PMID:11698684,PMID:9737924;

AA2 72I;Interacts with:56R

AA2 73M;Interacts with:49L,168A,169E,171M,56R,57H,164E,165V,53L

MUT2 M73A;PMID:9737924;

MUT2 M73V;dbSNP:rs142850110;dbSNP:rs142850110;

AA2 74S;Interacts with:58D,49L,172R,169E,168A,59F,57H

AA2 75K;Interacts with:58D,56R,169E,57H,145Y

MUT2 K75A;PMID:11698684,PMID:9737924;

AA2 76P;Interacts with:49L,53L,52C,56R

AA2 77E;Interacts with:56R,52C,55D,53L,54K,57H,58D

AA2 78D;Interacts with:56R

AA2 79L;Interacts with:53L,50F

AA2 96T;Interacts with:185R

AA2 97D;Interacts with:185R

AA2 99W;Interacts with:185R

MUT2 W99A;PMID:9737924;

//

MESH1 mesh:D001249

NAME1 Asthma

CLUSTER1 clust1

MESH2 mesh:D003093

NAME2 Colitis, Ulcerative

CLUSTER2 clust1

AC1 P13232

PFAM1 PF01415

PDB1 3DI2:A

AC2 P16871

PFAM2 PF00041

PDB2 3DI2:B

INT non-edgetic

AA1 103H;Interacts with:159Y

AA1 106K;Interacts with:159Y

AA1 35K;Interacts with:212Y

AA1 36Q;Interacts with:212Y

AA1 39S;Interacts with:211H,159Y,212Y,213F

AA1 40V;Interacts with:213F,212Y,159Y

AA1 43V;Interacts with:159Y,158K

AA1 44S;Interacts with:159Y

AA1 47Q;Interacts with:158K

AA2 158K;Interacts with:43V,47Q

AA2 159Y;Interacts with:39S,103H,43V,106K,40V,44S

AA2 211H;Interacts with:39S

AA2 212Y;Interacts with:40V,36Q,39S,35K

AA2 213F;Interacts with:40V,39S

//

MESH1 mesh:D001249

NAME1 Asthma

CLUSTER1 clust1

MESH2 mesh:D003093

NAME2 Colitis, Ulcerative

CLUSTER2 clust1

AC1 Q13651

PFAM1 PF09294

PDB1 1J7V:R

AC2 P22301

PFAM2 PF00726

PDB2 1J7V:R

INT semi-edgetic

AA1 164F;Interacts with:38P

AA1 166E;Interacts with:42R

AA1 208S;Interacts with:42R

AA1 210A;Interacts with:39N,42R,38P

AA1 211S;Interacts with:42R,38P,41L,45R,43D

AA1 212R;Interacts with:38P,42R

AA1 213S;Interacts with:46D,42R

AA1 214N;Interacts with:42R

AA2 38P;Interacts with:212R,211S,164F,210A

AA2 39N;Interacts with:210A

AA2 41L;Interacts with:211S

AA2 42R;Interacts with:211S,214N,208S,212R,166E,210A,213S

AA2 43D;Interacts with:211S

AA2 45R;Interacts with:211S

AA2 46D;Interacts with:213S

//

MESH1 mesh:D001249

NAME1 Asthma

CLUSTER1 clust1

MESH2 mesh:D003093

NAME2 Colitis, Ulcerative

CLUSTER2 clust1

AC1 Q13651

PFAM1 PF01108

PDB1 1J7V:R

AC2 P22301

PFAM2 PF00726

PDB2 1J7V:R

INT semi-edgetic

AA1 64Y;Interacts with:65L,64L,61L,62D,63N

AA1 65G;Interacts with:62D,63N,64L

AA1 66I;Interacts with:62D

AA1 67E;Interacts with:63N,62D,64L

AA1 69W;Interacts with:62D

AA1 94N;Interacts with:66L

AA1 97R;Interacts with:60Q,61L,59D,62D,56Q

AA2 56Q;Interacts with:97R

AA2 59D;Interacts with:97R

AA2 60Q;Interacts with:97R

MUT2 Q60E;PMID:10637267;

AA2 61L;Interacts with:97R,64Y

MUT2 L61V;PMID:10637267;

AA2 62D;Interacts with:69W,66I,65G,67E,97R,64Y

AA2 63N;Interacts with:67E,65G,64Y

AA2 64L;Interacts with:64Y,67E,65G

AA2 65L;Interacts with:64Y

AA2 66L;Interacts with:94N

//

MESH1 mesh:D001249

NAME1 Asthma

CLUSTER1 clust1

MESH2 mesh:D003093

NAME2 Colitis, Ulcerative

CLUSTER2 clust1

AC1 Q9GZX6

PFAM1 PF14565

PDB1 3DGC:L

AC2 Q8N6P7

PFAM2 PF09294

PDB2 3DGC:R

INT semi-edgetic

AA1 116Q;Interacts with:133R

AA1 117E;Interacts with:191L,133R

AA1 121F;Interacts with:189F

AA1 124R;Interacts with:187E,185E,189F,134S,136Q

AA1 128R;Interacts with:187E,185E,186Y

AA1 172M;Interacts with:207T,208W

MUT1 M172V;dbSNP:rs147812317;

AA1 175R;Interacts with:162D,207T

AA1 43D;Interacts with:183Q,185E

AA1 44K;Interacts with:162D,207T,183Q

AA1 45S;Interacts with:179L,183Q,187E

AA1 46N;Interacts with:189F

AA1 48Q;Interacts with:206P,162D,207T,180G

AA1 49Q;Interacts with:187E,189F,190G

AA1 51Y;Interacts with:190G,175Y

AA1 52I;Interacts with:189F,190G

AA1 57F;Interacts with:208W

AA2 133R;Interacts with:116Q,117E

AA2 134S;Interacts with:124R

AA2 136Q;Interacts with:124R

AA2 162D;Interacts with:175R,48Q,44K

AA2 175Y;Interacts with:51Y

AA2 179L;Interacts with:45S

AA2 180G;Interacts with:48Q

AA2 183Q;Interacts with:45S,43D,44K

AA2 185E;Interacts with:128R,124R,43D

AA2 186Y;Interacts with:128R

AA2 187E;Interacts with:124R,49Q,128R,45S

AA2 189F;Interacts with:46N,52I,49Q,121F,124R

AA2 190G;Interacts with:51Y,52I,49Q

AA2 191L;Interacts with:117E

AA2 206P;Interacts with:48Q

AA2 207T;Interacts with:172M,175R,48Q,44K

AA2 208W;Interacts with:57F,172M

//

MESH1 mesh:D001249

NAME1 Asthma

CLUSTER1 clust1

MESH2 mesh:D003093

NAME2 Colitis, Ulcerative

CLUSTER2 clust1

AC1 Q9GZX6

PFAM1 PF14565

PDB1 3DGC:L

AC2 Q8N6P7

PFAM2 PF01108

PDB2 3DGC:R

INT semi-edgetic

AA1 169L;Interacts with:60Y,91L

AA1 69N;Interacts with:60Y

AA1 70T;Interacts with:58K,61G,60Y,62E,93Y,65W

AA1 71D;Interacts with:58K,61G,63R,64D,62E

AA1 72V;Interacts with:62E,60Y,61G

AA1 73R;Interacts with:61G,90E,89T,60Y,91L

MUT1 R73H;dbSNP:rs149366319;

AA1 74L;Interacts with:60Y

AA1 77E;Interacts with:89T

AA2 58K;Interacts with:71D,70T

MUT2 K58A;882419;

AA2 60Y;Interacts with:72V,70T,169L,73R,69N,74L

MUT2 Y60A;1368880;

MUT2 Y60R;1368881;

AA2 61G;Interacts with:73R,72V,70T,71D

AA2 62E;Interacts with:72V,70T,71D

AA2 63R;Interacts with:71D

AA2 64D;Interacts with:71D

AA2 65W;Interacts with:70T

AA2 89T;Interacts with:77E,73R

AA2 90E;Interacts with:73R

AA2 91L;Interacts with:73R,169L

MUT2 L91F;dbSNP:rs144035143;

AA2 93Y;Interacts with:70T

//

MESH1 mesh:D001249

NAME1 Asthma

CLUSTER1 clust1

MESH2 mesh:D003093

NAME2 Colitis, Ulcerative

CLUSTER2 clust1

AC1 Q9GZX6

PFAM1 PF14565

PDB1 3G9V:D

AC2 Q969J5

PFAM2 PF09294

PDB2 3G9V:C

INT semi-edgetic

AA1 175R;Interacts with:200E

AA1 48Q;Interacts with:247M

AA1 50P;Interacts with:247M,249D,248L,246P

AA1 51Y;Interacts with:247M

AA1 53T;Interacts with:247M,248L

AA1 54N;Interacts with:247M,248L,250R,249D

AA2 200E;Interacts with:175R

AA2 246P;Interacts with:50P

AA2 247M;Interacts with:54N,50P,51Y,53T,48Q

AA2 248L;Interacts with:54N,53T,50P

AA2 249D;Interacts with:54N,50P

AA2 250R;Interacts with:54N

//

MESH1 mesh:D001249

NAME1 Asthma

CLUSTER1 clust1

MESH2 mesh:D003093

NAME2 Colitis, Ulcerative

CLUSTER2 clust1

AC1 Q9GZX6

PFAM1 PF14565

PDB1 3G9V:B

AC2 Q969J5

PFAM2 PF01108

PDB2 3G9V:A

INT semi-edgetic

AA1 169L;Interacts with:99Y

AA1 175R;Interacts with:128Q

AA1 176N;Interacts with:128Q

AA1 69N;Interacts with:99Y

AA1 70T;Interacts with:104W,99Y,132Y,100G,65K,101Q

AA1 71D;Interacts with:101Q,103Q,100G,104W,65K,102R

AA1 72V;Interacts with:99Y,100G,101Q

AA1 73R;Interacts with:129E,130P,128Q,100G,99Y,66I

MUT1 R73H;dbSNP:rs149366319;

AA2 100G;Interacts with:70T,71D,72V,73R

AA2 101Q;Interacts with:71D,72V,70T

AA2 102R;Interacts with:71D

AA2 103Q;Interacts with:71D

AA2 104W;Interacts with:70T,71D

AA2 128Q;Interacts with:175R,73R,176N

AA2 129E;Interacts with:73R

AA2 130P;Interacts with:73R

AA2 132Y;Interacts with:70T

AA2 65K;Interacts with:70T,71D

AA2 66I;Interacts with:73R

AA2 99Y;Interacts with:70T,169L,72V,69N,73R

//

MESH1 mesh:D001249

NAME1 Asthma

CLUSTER1 clust1

MESH2 mesh:D003093

NAME2 Colitis, Ulcerative

CLUSTER2 clust1

AC1 P60568

PFAM1 PF00715

PDB1 1Z92:A

AC2 P01589

PFAM2 PF00084

PDB2 1Z92:B

INT edgetic

AA1 125C;Interacts with:56R,59K

AA1 126E;Interacts with:56R

AA1 127Y;Interacts with:60S,56R,59K

AA1 128A;Interacts with:56R

AA1 54P;Interacts with:25D

MUT1 P54S;1KG:1244597;

AA1 55K;Interacts with:25D,24C

AA1 58R;Interacts with:26D,25D,24C,27D

AA1 61T;Interacts with:48N

AA1 62F;Interacts with:48N,63L,64Y

AA1 63K;Interacts with:63L,50E,57R

AA1 65Y;Interacts with:56R,57R

AA1 81E;Interacts with:57R,59K,60S,61G

AA1 82E;Interacts with:60S,61G,57R,56R

AA1 84K;Interacts with:62S,61G,60S,78N

AA1 85P;Interacts with:63L,60S,61G

AA1 88E;Interacts with:64Y,78N,63L,62S

AA1 91N;Interacts with:64Y

AA1 92L;Interacts with:64Y,46M

AA2 24C;Interacts with:58R,55K

MUT2 C24A;PMID:2832473;

AA2 25D;Interacts with:54P,55K,58R

MUT2 D25K;PMID:3135551;

MUT2 D25N;1KG:1340011;

AA2 26D;Interacts with:58R

MUT2 D26H;dbSNP:rs55868253;

AA2 27D;Interacts with:58R

AA2 46M;Interacts with:92L

AA2 48N;Interacts with:62F,61T

MUT2 N48A;PMID:3135551;

AA2 50E;Interacts with:63K

MUT2 E50R;PMID:3135551;

MUT2 E50A;PMID:3135551;

AA2 56R;Interacts with:65Y,126E,128A,125C,127Y,82E

AA2 57R;Interacts with:81E,82E,65Y,63K

AA2 59K;Interacts with:81E,127Y,125C

MUT2 K59T;PMID:3135551;

AA2 60S;Interacts with:82E,127Y,81E,85P,84K

AA2 61G;Interacts with:82E,81E,84K,85P

MUT2 G61R;1KG:1340008;

AA2 62S;Interacts with:88E,84K

AA2 63L;Interacts with:63K,88E,85P,62F

AA2 64Y;Interacts with:92L,88E,91N,62F

AA2 78N;Interacts with:88E,84K

//

MESH1 mesh:D001249

NAME1 Asthma

CLUSTER1 clust1

MESH2 mesh:D003093

NAME2 Colitis, Ulcerative

CLUSTER2 clust1

AC1 P01579

PFAM1 PF00714

PDB1 1FYH:A

AC2 P15260

PFAM2 PF07140

PDB2 1FYH:E

INT edgetic

AA1 146A;Interacts with:186K,203A,205P,185Y

AA1 147A;Interacts with:183I,185Y

MUT1 A147L;PMID:1662603;

AA1 149T;Interacts with:185Y

AA1 150G;Interacts with:208S,205P,207S

AA1 151K;Interacts with:209L,207S,208S

MUT1 K151R;PMID:2127102;

MUT1 K151E;PMID:1830392;

AA2 183I;Interacts with:147A

AA2 185Y;Interacts with:146A,147A,149T

AA2 186K;Interacts with:146A

AA2 203A;Interacts with:146A

AA2 205P;Interacts with:150G,146A

AA2 207S;Interacts with:151K,150G

AA2 208S;Interacts with:150G,151K

AA2 209L;Interacts with:151K

//

MESH1 mesh:D001249

NAME1 Asthma

CLUSTER1 clust1

MESH2 mesh:D003093

NAME2 Colitis, Ulcerative

CLUSTER2 clust1

AC1 P01579

PFAM1 PF00714

PDB1 1FYH:A

AC2 P15260

PFAM2 PF01108

PDB2 1FYH:B

INT edgetic

AA1 163R;Interacts with:66Y

AA2 66Y;Interacts with:163R

MUT2 Y66A;PMID:9878445;

MUT2 Y66F;PMID:9878445;

//

MESH1 mesh:D001249

NAME1 Asthma

CLUSTER1 clust1

MESH2 mesh:D003424

NAME2 Crohn Disease

CLUSTER2 clust1

AC1 P29459

PFAM1 PF03039

PDB1 3HMX:B

AC2 P29460

PFAM2 PF10420

PDB2 3HMX:A

INT non-edgetic

AA1 205R;Interacts with:203E

AA1 208T;Interacts with:203E,202A

AA1 211R;Interacts with:136Y

AA1 71H;Interacts with:205S

AA1 76K;Interacts with:205S

AA1 82V;Interacts with:201A,202A,203E

AA1 83E;Interacts with:201A,202A

AA1 85C;Interacts with:202A

AA1 86L;Interacts with:202A,200P,201A

AA2 136Y;Interacts with:211R

AA2 200P;Interacts with:86L

AA2 201A;Interacts with:82V,83E,86L

AA2 202A;Interacts with:85C,82V,86L,83E,208T

AA2 203E;Interacts with:208T,82V,205R

AA2 205S;Interacts with:71H,76K

//

MESH1 mesh:D001249

NAME1 Asthma

CLUSTER1 clust1

MESH2 mesh:D003424

NAME2 Crohn Disease

CLUSTER2 clust1

AC1 P13232

PFAM1 PF01415

PDB1 3DI2:A

AC2 P16871

PFAM2 PF00041

PDB2 3DI2:B

INT non-edgetic

AA1 103H;Interacts with:159Y

AA1 106K;Interacts with:159Y

AA1 35K;Interacts with:212Y

AA1 36Q;Interacts with:212Y

AA1 39S;Interacts with:211H,159Y,212Y,213F

AA1 40V;Interacts with:213F,212Y,159Y

AA1 43V;Interacts with:159Y,158K

AA1 44S;Interacts with:159Y

AA1 47Q;Interacts with:158K

AA2 158K;Interacts with:43V,47Q

AA2 159Y;Interacts with:39S,103H,43V,106K,40V,44S

AA2 211H;Interacts with:39S

AA2 212Y;Interacts with:40V,36Q,39S,35K

AA2 213F;Interacts with:40V,39S

//

MESH1 mesh:D001249

NAME1 Asthma

CLUSTER1 clust1

MESH2 mesh:D003424

NAME2 Crohn Disease

CLUSTER2 clust1

AC1 P15018

PFAM1 PF01291

PDB1 1PVH:B

AC2 P40189

PFAM2 PF09240

PDB2 1PVH:B

INT semi-edgetic

AA1 142D;Interacts with:189V,187S

AA1 143I;Interacts with:189V

AA1 145R;Interacts with:164W

AA1 146G;Interacts with:192V,164W,189V

AA1 149S;Interacts with:165A,166T

AA1 150N;Interacts with:191F,193N,192V

AA1 152L;Interacts with:166T

AA1 47Q;Interacts with:191F,192V,215D,193N

AA1 50S;Interacts with:191F

AA1 51Q;Interacts with:191F,192V

AA1 54Q;Interacts with:190Y

AA2 164W;Interacts with:146G,145R

AA2 165A;Interacts with:149S

AA2 166T;Interacts with:149S,152L

AA2 187S;Interacts with:142D

AA2 189V;Interacts with:143I,142D,146G

AA2 190Y;Interacts with:54Q

AA2 191F;Interacts with:150N,47Q,51Q,50S

AA2 192V;Interacts with:146G,47Q,51Q,150N

AA2 193N;Interacts with:150N,47Q

MUT2 N193delN;COSMIC:251360;

AA2 215D;Interacts with:47Q

//

MESH1 mesh:D001249

NAME1 Asthma

CLUSTER1 clust1

MESH2 mesh:D003424

NAME2 Crohn Disease

CLUSTER2 clust1

AC1 P01563

PFAM1 PF00143

PDB1 2HYM:B

AC2 P48551

PFAM2 PF01108

PDB2 2HYM:A

INT semi-edgetic

AA1 145Y;Interacts with:75K

AA1 164E;Interacts with:73M

AA1 165V;Interacts with:73M

AA1 168A;Interacts with:73M,74S,105A

MUT1 A168T;dbSNP:rs143535431;

MUT1 A168G;PMID:11698684;

MUT1 A168M;PMID:10984492;

AA1 169E;Interacts with:73M,75K,74S

AA1 171M;Interacts with:73M

MUT1 M171A;PMID:10984492;PMID:11698684;

MUT1 M171I;dbSNP:rs77047588;

AA1 172R;Interacts with:104E,103H,74S,101S

AA1 175S;Interacts with:103H,104E

MUT1 S175P;dbSNP:rs138547087;

AA1 176L;Interacts with:103H

AA1 178T;Interacts with:103H

AA1 179N;Interacts with:103H,102T

AA1 182E;Interacts with:103H

AA1 185R;Interacts with:96T,99W,102T,100R,97D

AA1 186S;Interacts with:100R

AA1 48S;Interacts with:107V

MUT1 S48A;PMID:10984492;PMID:10556041;

AA1 49L;Interacts with:76P,73M,74S,107V,105A

MUT1 L49A;PMID:10984492;PMID:11698684,PMID:10556041;

AA1 50F;Interacts with:107V,79L,109V,71T,108T,70Y

AA1 52C;Interacts with:76P,77E

AA1 53L;Interacts with:79L,76P,77E,107V,71T,73M

MUT1 L53I;PMID:3081003;

MUT1 L53S;dbSNP:rs145655120;

AA1 54K;Interacts with:77E

AA1 55D;Interacts with:77E

MUT1 D55A;PMID:10984492;PMID:3081003,PMID:10556041;

AA1 56R;Interacts with:77E,75K,78D,76P,73M,71T,72I

MUT1 R56K;PMID:10984492;PMID:3081003;

AA1 57H;Interacts with:75K,77E,73M,74S

MUT1 H57R;883731;dbSNP:rs73420190;humsavar:VAR_013001;

AA1 58D;Interacts with:74S,75K,77E

AA1 59F;Interacts with:74S

MUT1 F59S;dbSNP:rs147918701;

AA2 100R;Interacts with:186S,185R

AA2 101S;Interacts with:172R

AA2 102T;Interacts with:185R,179N

AA2 103H;Interacts with:176L,175S,172R,178T,179N,182E

AA2 104E;Interacts with:172R,175S

AA2 105A;Interacts with:49L,168A

AA2 107V;Interacts with:50F,49L,48S,53L

AA2 108T;Interacts with:50F

AA2 109V;Interacts with:50F

MUT2 V109I;dbSNP:rs140084698;dbSNP:rs140084698;

AA2 70Y;Interacts with:50F

AA2 71T;Interacts with:50F,56R,53L

MUT2 T71A;PMID:11698684,PMID:9737924;

AA2 72I;Interacts with:56R

AA2 73M;Interacts with:49L,168A,169E,171M,56R,57H,164E,165V,53L

MUT2 M73A;PMID:9737924;

MUT2 M73V;dbSNP:rs142850110;dbSNP:rs142850110;

AA2 74S;Interacts with:58D,49L,172R,169E,168A,59F,57H

AA2 75K;Interacts with:58D,56R,169E,57H,145Y

MUT2 K75A;PMID:11698684,PMID:9737924;

AA2 76P;Interacts with:49L,53L,52C,56R

AA2 77E;Interacts with:56R,52C,55D,53L,54K,57H,58D

AA2 78D;Interacts with:56R

AA2 79L;Interacts with:53L,50F

AA2 96T;Interacts with:185R

AA2 97D;Interacts with:185R

AA2 99W;Interacts with:185R

MUT2 W99A;PMID:9737924;

//

MESH1 mesh:D001249

NAME1 Asthma

CLUSTER1 clust1

MESH2 mesh:D003424

NAME2 Crohn Disease

CLUSTER2 clust1

AC1 Q13651

PFAM1 PF09294

PDB1 1J7V:R

AC2 P22301

PFAM2 PF00726

PDB2 1J7V:R

INT semi-edgetic

AA1 164F;Interacts with:38P

AA1 166E;Interacts with:42R

AA1 208S;Interacts with:42R

AA1 210A;Interacts with:39N,42R,38P

AA1 211S;Interacts with:42R,38P,41L,45R,43D

AA1 212R;Interacts with:38P,42R

AA1 213S;Interacts with:46D,42R

AA1 214N;Interacts with:42R

AA2 38P;Interacts with:212R,211S,164F,210A

AA2 39N;Interacts with:210A

AA2 41L;Interacts with:211S

AA2 42R;Interacts with:211S,214N,208S,212R,166E,210A,213S

AA2 43D;Interacts with:211S

AA2 45R;Interacts with:211S

AA2 46D;Interacts with:213S

//

MESH1 mesh:D001249

NAME1 Asthma

CLUSTER1 clust1

MESH2 mesh:D003424

NAME2 Crohn Disease

CLUSTER2 clust1

AC1 Q13651

PFAM1 PF01108

PDB1 1J7V:R

AC2 P22301

PFAM2 PF00726

PDB2 1J7V:R

INT semi-edgetic

AA1 64Y;Interacts with:65L,64L,61L,62D,63N

AA1 65G;Interacts with:62D,63N,64L

AA1 66I;Interacts with:62D

AA1 67E;Interacts with:63N,62D,64L

AA1 69W;Interacts with:62D

AA1 94N;Interacts with:66L

AA1 97R;Interacts with:60Q,61L,59D,62D,56Q

AA2 56Q;Interacts with:97R

AA2 59D;Interacts with:97R

AA2 60Q;Interacts with:97R

MUT2 Q60E;PMID:10637267;

AA2 61L;Interacts with:97R,64Y

MUT2 L61V;PMID:10637267;

AA2 62D;Interacts with:69W,66I,65G,67E,97R,64Y

AA2 63N;Interacts with:67E,65G,64Y

AA2 64L;Interacts with:64Y,67E,65G

AA2 65L;Interacts with:64Y

AA2 66L;Interacts with:94N

//

MESH1 mesh:D001249

NAME1 Asthma

CLUSTER1 clust1

MESH2 mesh:D003424

NAME2 Crohn Disease

CLUSTER2 clust1

AC1 Q9GZX6

PFAM1 PF14565

PDB1 3DGC:L

AC2 Q8N6P7

PFAM2 PF09294

PDB2 3DGC:R

INT semi-edgetic

AA1 116Q;Interacts with:133R

AA1 117E;Interacts with:191L,133R

AA1 121F;Interacts with:189F

AA1 124R;Interacts with:187E,185E,189F,134S,136Q

AA1 128R;Interacts with:187E,185E,186Y

AA1 172M;Interacts with:207T,208W

MUT1 M172V;dbSNP:rs147812317;

AA1 175R;Interacts with:162D,207T

AA1 43D;Interacts with:183Q,185E

AA1 44K;Interacts with:162D,207T,183Q

AA1 45S;Interacts with:179L,183Q,187E

AA1 46N;Interacts with:189F

AA1 48Q;Interacts with:206P,162D,207T,180G

AA1 49Q;Interacts with:187E,189F,190G

AA1 51Y;Interacts with:190G,175Y

AA1 52I;Interacts with:189F,190G

AA1 57F;Interacts with:208W

AA2 133R;Interacts with:116Q,117E

AA2 134S;Interacts with:124R

AA2 136Q;Interacts with:124R

AA2 162D;Interacts with:175R,48Q,44K

AA2 175Y;Interacts with:51Y

AA2 179L;Interacts with:45S

AA2 180G;Interacts with:48Q

AA2 183Q;Interacts with:45S,43D,44K

AA2 185E;Interacts with:128R,124R,43D

AA2 186Y;Interacts with:128R

AA2 187E;Interacts with:124R,49Q,128R,45S

AA2 189F;Interacts with:46N,52I,49Q,121F,124R

AA2 190G;Interacts with:51Y,52I,49Q

AA2 191L;Interacts with:117E

AA2 206P;Interacts with:48Q

AA2 207T;Interacts with:172M,175R,48Q,44K

AA2 208W;Interacts with:57F,172M

//

MESH1 mesh:D001249

NAME1 Asthma

CLUSTER1 clust1

MESH2 mesh:D003424

NAME2 Crohn Disease

CLUSTER2 clust1

AC1 Q9GZX6

PFAM1 PF14565

PDB1 3DGC:L

AC2 Q8N6P7

PFAM2 PF01108

PDB2 3DGC:R

INT semi-edgetic

AA1 169L;Interacts with:60Y,91L

AA1 69N;Interacts with:60Y

AA1 70T;Interacts with:58K,61G,60Y,62E,93Y,65W

AA1 71D;Interacts with:58K,61G,63R,64D,62E

AA1 72V;Interacts with:62E,60Y,61G

AA1 73R;Interacts with:61G,90E,89T,60Y,91L

MUT1 R73H;dbSNP:rs149366319;

AA1 74L;Interacts with:60Y

AA1 77E;Interacts with:89T

AA2 58K;Interacts with:71D,70T

MUT2 K58A;882419;

AA2 60Y;Interacts with:72V,70T,169L,73R,69N,74L

MUT2 Y60A;1368880;

MUT2 Y60R;1368881;

AA2 61G;Interacts with:73R,72V,70T,71D

AA2 62E;Interacts with:72V,70T,71D

AA2 63R;Interacts with:71D

AA2 64D;Interacts with:71D

AA2 65W;Interacts with:70T

AA2 89T;Interacts with:77E,73R

AA2 90E;Interacts with:73R

AA2 91L;Interacts with:73R,169L

MUT2 L91F;dbSNP:rs144035143;

AA2 93Y;Interacts with:70T

//

MESH1 mesh:D001249

NAME1 Asthma

CLUSTER1 clust1

MESH2 mesh:D003424

NAME2 Crohn Disease

CLUSTER2 clust1

AC1 P08887

PFAM1 PF09240

PDB1 1P9M:C

AC2 P05231

PFAM2 PF00489

PDB2 1P9M:C

INT semi-edgetic

AA1 155N;Interacts with:89N,94K

AA1 182E;Interacts with:97E,102F,207R

AA1 183G;Interacts with:102F

AA1 185S;Interacts with:94K

AA1 187F;Interacts with:82K

AA1 209Q;Interacts with:82K

AA1 211C;Interacts with:82K

MUT1 C211A;884560;

AA1 212G;Interacts with:82K

AA2 102F;Interacts with:183G,182E

AA2 207R;Interacts with:182E

MUT2 R207S;PMID:8436132,PMID:8483922;

MUT2 R207A;PMID:8436132,PMID:8483922;

MUT2 R207P;PMID:8436132,PMID:8483922;

MUT2 R207G;PMID:8436132,PMID:8483922;

MUT2 R207H;PMID:8436132;

MUT2 R207K;PMID:1321818,PMID:8436132,PMID:8483922;

MUT2 R207N;PMID:8436132,PMID:8483922;

MUT2 R207W;PMID:8436132;

AA2 82K;Interacts with:212G,209Q,211C,187F

AA2 89N;Interacts with:155N

AA2 94K;Interacts with:185S,155N

AA2 97E;Interacts with:182E

//

MESH1 mesh:D001249

NAME1 Asthma

CLUSTER1 clust1

MESH2 mesh:D003424

NAME2 Crohn Disease

CLUSTER2 clust1

AC1 Q9GZX6

PFAM1 PF14565

PDB1 3G9V:D

AC2 Q969J5

PFAM2 PF09294

PDB2 3G9V:C

INT semi-edgetic

AA1 175R;Interacts with:200E

AA1 48Q;Interacts with:247M

AA1 50P;Interacts with:247M,249D,248L,246P

AA1 51Y;Interacts with:247M

AA1 53T;Interacts with:247M,248L

AA1 54N;Interacts with:247M,248L,250R,249D

AA2 200E;Interacts with:175R

AA2 246P;Interacts with:50P

AA2 247M;Interacts with:54N,50P,51Y,53T,48Q

AA2 248L;Interacts with:54N,53T,50P

AA2 249D;Interacts with:54N,50P

AA2 250R;Interacts with:54N

//

MESH1 mesh:D001249

NAME1 Asthma

CLUSTER1 clust1

MESH2 mesh:D003424

NAME2 Crohn Disease

CLUSTER2 clust1

AC1 Q9GZX6

PFAM1 PF14565

PDB1 3G9V:B

AC2 Q969J5

PFAM2 PF01108

PDB2 3G9V:A

INT semi-edgetic

AA1 169L;Interacts with:99Y

AA1 175R;Interacts with:128Q

AA1 176N;Interacts with:128Q

AA1 69N;Interacts with:99Y

AA1 70T;Interacts with:104W,99Y,132Y,100G,65K,101Q

AA1 71D;Interacts with:101Q,103Q,100G,104W,65K,102R

AA1 72V;Interacts with:99Y,100G,101Q

AA1 73R;Interacts with:129E,130P,128Q,100G,99Y,66I

MUT1 R73H;dbSNP:rs149366319;

AA2 100G;Interacts with:70T,71D,72V,73R

AA2 101Q;Interacts with:71D,72V,70T

AA2 102R;Interacts with:71D

AA2 103Q;Interacts with:71D

AA2 104W;Interacts with:70T,71D

AA2 128Q;Interacts with:175R,73R,176N

AA2 129E;Interacts with:73R

AA2 130P;Interacts with:73R

AA2 132Y;Interacts with:70T

AA2 65K;Interacts with:70T,71D

AA2 66I;Interacts with:73R

AA2 99Y;Interacts with:70T,169L,72V,69N,73R

//

MESH1 mesh:D001249

NAME1 Asthma

CLUSTER1 clust1

MESH2 mesh:D003424

NAME2 Crohn Disease

CLUSTER2 clust1

AC1 P05113

PFAM1 PF02025

PDB1 3QT2:C

AC2 Q01344

PFAM2 PF09240

PDB2 3QT2:A

INT semi-edgetic

AA1 121E;Interacts with:209D,208R

MUT1 E121A;PMID:7761472,PMID:7797578;

AA1 124G;Interacts with:208R

MUT1 G124A;PMID:7479902;

AA1 125V;Interacts with:208R

AA1 128T;Interacts with:208R,206K

MUT1 T128A;PMID:7761472,PMID:7479902;

AA1 129E;Interacts with:206K,204L,208R

MUT1 E129R;PMID:7761472;

AA1 131I;Interacts with:175Y,176G,204L,178W

AA1 132I;Interacts with:177S

AA1 133E;Interacts with:177S

AA1 58K;Interacts with:208R,209D

AA2 175Y;Interacts with:131I

AA2 176G;Interacts with:131I

AA2 177S;Interacts with:132I,133E

AA2 178W;Interacts with:131I

AA2 204L;Interacts with:129E,131I

AA2 206K;Interacts with:129E,128T

AA2 208R;Interacts with:121E,128T,124G,58K,125V,129E

MUT2 R208H;1KG:1179373;1KG:1229851;1KG:1229780;

AA2 209D;Interacts with:121E,58K

//

MESH1 mesh:D001249

NAME1 Asthma

CLUSTER1 clust1

MESH2 mesh:D003424

NAME2 Crohn Disease

CLUSTER2 clust1

AC1 P01579

PFAM1 PF00714

PDB1 1FYH:A

AC2 P15260

PFAM2 PF07140

PDB2 1FYH:E

INT edgetic

AA1 146A;Interacts with:186K,203A,205P,185Y

AA1 147A;Interacts with:183I,185Y

MUT1 A147L;PMID:1662603;

AA1 149T;Interacts with:185Y

AA1 150G;Interacts with:208S,205P,207S

AA1 151K;Interacts with:209L,207S,208S

MUT1 K151R;PMID:2127102;

MUT1 K151E;PMID:1830392;

AA2 183I;Interacts with:147A

AA2 185Y;Interacts with:146A,147A,149T

AA2 186K;Interacts with:146A

AA2 203A;Interacts with:146A

AA2 205P;Interacts with:150G,146A

AA2 207S;Interacts with:151K,150G

AA2 208S;Interacts with:150G,151K

AA2 209L;Interacts with:151K

//

MESH1 mesh:D001249

NAME1 Asthma

CLUSTER1 clust1

MESH2 mesh:D003424

NAME2 Crohn Disease

CLUSTER2 clust1

AC1 P01579

PFAM1 PF00714

PDB1 1FYH:A

AC2 P15260

PFAM2 PF01108

PDB2 1FYH:B

INT edgetic

AA1 163R;Interacts with:66Y

AA2 66Y;Interacts with:163R

MUT2 Y66A;PMID:9878445;

MUT2 Y66F;PMID:9878445;

//

MESH1 mesh:D001249

NAME1 Asthma

CLUSTER1 clust1

MESH2 mesh:D003876

NAME2 Dermatitis, Atopic

CLUSTER2 clust1

AC1 P13232

PFAM1 PF01415

PDB1 3DI2:A

AC2 P16871

PFAM2 PF00041

PDB2 3DI2:B

INT non-edgetic

AA1 103H;Interacts with:159Y

AA1 106K;Interacts with:159Y

AA1 35K;Interacts with:212Y

AA1 36Q;Interacts with:212Y

AA1 39S;Interacts with:211H,159Y,212Y,213F

AA1 40V;Interacts with:213F,212Y,159Y

AA1 43V;Interacts with:159Y,158K

AA1 44S;Interacts with:159Y

AA1 47Q;Interacts with:158K

AA2 158K;Interacts with:43V,47Q

AA2 159Y;Interacts with:39S,103H,43V,106K,40V,44S

AA2 211H;Interacts with:39S

AA2 212Y;Interacts with:40V,36Q,39S,35K

AA2 213F;Interacts with:40V,39S

//

MESH1 mesh:D001249

NAME1 Asthma

CLUSTER1 clust1

MESH2 mesh:D003876

NAME2 Dermatitis, Atopic

CLUSTER2 clust1

AC1 P15018

PFAM1 PF01291

PDB1 1PVH:B

AC2 P40189

PFAM2 PF09240

PDB2 1PVH:B

INT semi-edgetic

AA1 142D;Interacts with:189V,187S

AA1 143I;Interacts with:189V

AA1 145R;Interacts with:164W

AA1 146G;Interacts with:192V,164W,189V

AA1 149S;Interacts with:165A,166T

AA1 150N;Interacts with:191F,193N,192V

AA1 152L;Interacts with:166T

AA1 47Q;Interacts with:191F,192V,215D,193N

AA1 50S;Interacts with:191F

AA1 51Q;Interacts with:191F,192V

AA1 54Q;Interacts with:190Y

AA2 164W;Interacts with:146G,145R

AA2 165A;Interacts with:149S

AA2 166T;Interacts with:149S,152L

AA2 187S;Interacts with:142D

AA2 189V;Interacts with:143I,142D,146G

AA2 190Y;Interacts with:54Q

AA2 191F;Interacts with:150N,47Q,51Q,50S

AA2 192V;Interacts with:146G,47Q,51Q,150N

AA2 193N;Interacts with:150N,47Q

MUT2 N193delN;COSMIC:251360;

AA2 215D;Interacts with:47Q

//

MESH1 mesh:D001249

NAME1 Asthma

CLUSTER1 clust1

MESH2 mesh:D003876

NAME2 Dermatitis, Atopic

CLUSTER2 clust1

AC1 P31785

PFAM1 PF00041

PDB1 2B5I:C

AC2 P60568

PFAM2 PF00715

PDB2 2B5I:C

INT semi-edgetic

AA1 181H;Interacts with:31Q,150S,149I,35E

AA1 190R;Interacts with:125C,127Y,81E

AA1 195H;Interacts with:65Y,127Y

AA1 197W;Interacts with:125C,126E,124M

AA1 220T;Interacts with:124M

AA1 222R;Interacts with:124M,126E

MUT1 R222C;160917;humsavar:VAR_002688, phenotype=X-linked combined immunodeficiency (XCID);

AA1 228N;Interacts with:146Q,42Q

AA1 229P;Interacts with:38L,146Q,42Q

AA1 230L;Interacts with:38L,35E,150S,149I,146Q,147S,39L,42Q

MUT1 L230P;884416;humsavar:VAR_002693, phenotype=Severe combined immunodeficiency X-linked T-cell-negative/B-cell-positive/NK-cell-negative (XSCID);

AA1 231C;Interacts with:147S,146Q,150S

AA1 232G;Interacts with:42Q,146Q

AA2 124M;Interacts with:222R,197W,220T

AA2 125C;Interacts with:190R,197W

AA2 126E;Interacts with:197W,222R

AA2 127Y;Interacts with:195H,190R

AA2 146Q;Interacts with:232G,229P,230L,231C,228N

AA2 147S;Interacts with:231C,230L

AA2 149I;Interacts with:230L,181H

AA2 150S;Interacts with:230L,181H,231C

AA2 31Q;Interacts with:181H

AA2 35E;Interacts with:230L,181H

AA2 38L;Interacts with:230L,229P

MUT2 L38R;dbSNP:rs3087209;

AA2 39L;Interacts with:230L

AA2 42Q;Interacts with:232G,229P,230L,228N

AA2 65Y;Interacts with:195H

AA2 81E;Interacts with:190R

//

MESH1 mesh:D001249

NAME1 Asthma

CLUSTER1 clust1

MESH2 mesh:D003876

NAME2 Dermatitis, Atopic

CLUSTER2 clust1

AC1 P31785

PFAM1 PF09240

PDB1 2B5I:C

AC2 P60568

PFAM2 PF00715

PDB2 2B5I:C

INT semi-edgetic

AA1 125Y;Interacts with:143T,146Q,147S,150S,140R

MUT1 Y125N;884404;humsavar:VAR_002679, phenotype=Severe combined immunodeficiency X-linked T-cell-negative/B-cell-positive/NK-cell-negative (XSCID);

AA1 147K;Interacts with:139N

AA1 149Q;Interacts with:143T,146Q,139N

AA1 150N;Interacts with:146Q,139N

AA1 92K;Interacts with:130E

AA1 93N;Interacts with:130E,68K

AA1 95D;Interacts with:69K

AA2 130E;Interacts with:93N,92K

AA2 139N;Interacts with:147K,149Q,150N

AA2 140R;Interacts with:125Y

AA2 143T;Interacts with:125Y,149Q

AA2 146Q;Interacts with:150N,125Y,149Q

AA2 147S;Interacts with:125Y

AA2 150S;Interacts with:125Y

AA2 68K;Interacts with:93N

AA2 69K;Interacts with:95D

//

MESH1 mesh:D001249

NAME1 Asthma

CLUSTER1 clust1

MESH2 mesh:D003876

NAME2 Dermatitis, Atopic

CLUSTER2 clust1

AC1 P05112

PFAM1 PF00727

PDB1 3BPL:A

AC2 P31785

PFAM2 PF00041

PDB2 3BPL:C

INT semi-edgetic

AA1 141K;Interacts with:229P

MUT1 K141D;PMID:9799097;

MUT1 K141Q;PMID:8262056,PMID:7517357;

AA1 145R;Interacts with:228N,230L,231C,232G,229P

MUT1 R145D;PMID:8262056,PMID:7517357,PMID:9799097;

AA1 146E;Interacts with:231C

AA1 148Y;Interacts with:230L,181H,231C,204Y

MUT1 Y148H;PMID:1387082,PMID:7517357;

MUT1 Y148D;PMID:1864379,PMID:1387082,PMID:8262056,PMID:,PMID:7957181,PMID:7517357,PMID:8151703,PMID:9799097;

MUT1 Y148G;PMID:1387082,PMID:,PMID:7517357,PMID:8151703;

AA1 149S;Interacts with:181H,227F

AA1 32Q;Interacts with:230L,204Y

AA1 35I;Interacts with:230L

AA1 39N;Interacts with:230L,229P

AA2 181H;Interacts with:148Y,149S

AA2 204Y;Interacts with:32Q,148Y

AA2 227F;Interacts with:149S

MUT2 F227C;884415;humsavar:VAR_002692, phenotype=Severe combined immunodeficiency X-linked T-cell-negative/B-cell-positive/NK-cell-negative (XSCID);

AA2 228N;Interacts with:145R

AA2 229P;Interacts with:39N,145R,141K

AA2 230L;Interacts with:39N,32Q,148Y,35I,145R

MUT2 L230P;884416;humsavar:VAR_002693, phenotype=Severe combined immunodeficiency X-linked T-cell-negative/B-cell-positive/NK-cell-negative (XSCID);

AA2 231C;Interacts with:146E,145R,148Y

AA2 232G;Interacts with:145R

//

MESH1 mesh:D001249

NAME1 Asthma

CLUSTER1 clust1

MESH2 mesh:D003876

NAME2 Dermatitis, Atopic

CLUSTER2 clust1

AC1 P05112

PFAM1 PF00727

PDB1 3BPL:A

AC2 P31785

PFAM2 PF09240

PDB2 3BPL:C

INT semi-edgetic

AA1 138E;Interacts with:147K,149Q

MUT1 E138R;PMID:9799097;

MUT1 E138Q;PMID:8262056,PMID:7517357;

AA1 139R;Interacts with:93N,125Y

AA1 141K;Interacts with:150N

AA1 142T;Interacts with:125Y,147K,149Q

MUT1 T142M;dbSNP:rs145068648;dbSNP:rs145068648;

AA1 145R;Interacts with:149Q,125Y,150N

MUT1 R145D;PMID:8262056,PMID:7517357,PMID:9799097;

AA1 146E;Interacts with:126Q,125Y

AA1 149S;Interacts with:125Y,67V,124L

AA2 124L;Interacts with:149S

AA2 125Y;Interacts with:142T,149S,145R,146E,139R

MUT2 Y125N;884404;humsavar:VAR_002679, phenotype=Severe combined immunodeficiency X-linked T-cell-negative/B-cell-positive/NK-cell-negative (XSCID);

AA2 126Q;Interacts with:146E

AA2 147K;Interacts with:142T,138E

AA2 149Q;Interacts with:145R,142T,138E

AA2 150N;Interacts with:145R,141K

AA2 67V;Interacts with:149S

AA2 93N;Interacts with:139R

//

MESH1 mesh:D001249

NAME1 Asthma

CLUSTER1 clust1

MESH2 mesh:D006526

NAME2 Hepatitis C

CLUSTER2 clust1

AC1 P29459

PFAM1 PF03039

PDB1 3HMX:B

AC2 P29460

PFAM2 PF10420

PDB2 3HMX:A

INT non-edgetic

AA1 205R;Interacts with:203E

AA1 208T;Interacts with:203E,202A

AA1 211R;Interacts with:136Y

AA1 71H;Interacts with:205S

AA1 76K;Interacts with:205S

AA1 82V;Interacts with:201A,202A,203E

AA1 83E;Interacts with:201A,202A

AA1 85C;Interacts with:202A

AA1 86L;Interacts with:202A,200P,201A

AA2 136Y;Interacts with:211R

AA2 200P;Interacts with:86L

AA2 201A;Interacts with:82V,83E,86L

AA2 202A;Interacts with:85C,82V,86L,83E,208T

AA2 203E;Interacts with:208T,82V,205R

AA2 205S;Interacts with:71H,76K

//

MESH1 mesh:D001249

NAME1 Asthma

CLUSTER1 clust1

MESH2 mesh:D006526

NAME2 Hepatitis C

CLUSTER2 clust1

AC1 P01563

PFAM1 PF00143

PDB1 2HYM:B

AC2 P48551

PFAM2 PF01108

PDB2 2HYM:A

INT semi-edgetic

AA1 145Y;Interacts with:75K

AA1 164E;Interacts with:73M

AA1 165V;Interacts with:73M

AA1 168A;Interacts with:73M,74S,105A

MUT1 A168T;dbSNP:rs143535431;

MUT1 A168G;PMID:11698684;

MUT1 A168M;PMID:10984492;

AA1 169E;Interacts with:73M,75K,74S

AA1 171M;Interacts with:73M

MUT1 M171A;PMID:10984492;PMID:11698684;

MUT1 M171I;dbSNP:rs77047588;

AA1 172R;Interacts with:104E,103H,74S,101S

AA1 175S;Interacts with:103H,104E

MUT1 S175P;dbSNP:rs138547087;

AA1 176L;Interacts with:103H

AA1 178T;Interacts with:103H

AA1 179N;Interacts with:103H,102T

AA1 182E;Interacts with:103H

AA1 185R;Interacts with:96T,99W,102T,100R,97D

AA1 186S;Interacts with:100R

AA1 48S;Interacts with:107V

MUT1 S48A;PMID:10984492;PMID:10556041;

AA1 49L;Interacts with:76P,73M,74S,107V,105A

MUT1 L49A;PMID:10984492;PMID:11698684,PMID:10556041;

AA1 50F;Interacts with:107V,79L,109V,71T,108T,70Y

AA1 52C;Interacts with:76P,77E

AA1 53L;Interacts with:79L,76P,77E,107V,71T,73M

MUT1 L53I;PMID:3081003;

MUT1 L53S;dbSNP:rs145655120;

AA1 54K;Interacts with:77E

AA1 55D;Interacts with:77E

MUT1 D55A;PMID:10984492;PMID:3081003,PMID:10556041;

AA1 56R;Interacts with:77E,75K,78D,76P,73M,71T,72I

MUT1 R56K;PMID:10984492;PMID:3081003;

AA1 57H;Interacts with:75K,77E,73M,74S

MUT1 H57R;883731;dbSNP:rs73420190;humsavar:VAR_013001;

AA1 58D;Interacts with:74S,75K,77E

AA1 59F;Interacts with:74S

MUT1 F59S;dbSNP:rs147918701;

AA2 100R;Interacts with:186S,185R

AA2 101S;Interacts with:172R

AA2 102T;Interacts with:185R,179N

AA2 103H;Interacts with:176L,175S,172R,178T,179N,182E

AA2 104E;Interacts with:172R,175S

AA2 105A;Interacts with:49L,168A

AA2 107V;Interacts with:50F,49L,48S,53L

AA2 108T;Interacts with:50F

AA2 109V;Interacts with:50F

MUT2 V109I;dbSNP:rs140084698;dbSNP:rs140084698;

AA2 70Y;Interacts with:50F

AA2 71T;Interacts with:50F,56R,53L

MUT2 T71A;PMID:11698684,PMID:9737924;

AA2 72I;Interacts with:56R

AA2 73M;Interacts with:49L,168A,169E,171M,56R,57H,164E,165V,53L

MUT2 M73A;PMID:9737924;

MUT2 M73V;dbSNP:rs142850110;dbSNP:rs142850110;

AA2 74S;Interacts with:58D,49L,172R,169E,168A,59F,57H

AA2 75K;Interacts with:58D,56R,169E,57H,145Y

MUT2 K75A;PMID:11698684,PMID:9737924;

AA2 76P;Interacts with:49L,53L,52C,56R

AA2 77E;Interacts with:56R,52C,55D,53L,54K,57H,58D

AA2 78D;Interacts with:56R

AA2 79L;Interacts with:53L,50F

AA2 96T;Interacts with:185R

AA2 97D;Interacts with:185R

AA2 99W;Interacts with:185R

MUT2 W99A;PMID:9737924;

//

MESH1 mesh:D001249

NAME1 Asthma

CLUSTER1 clust1

MESH2 mesh:D006526

NAME2 Hepatitis C

CLUSTER2 clust1

AC1 P04440

PFAM1 PF07654

PDB1 3LQZ:B

AC2 P20036

PFAM2 PF00993

PDB2 3LQZ:A

INT semi-edgetic

AA1 176R;Interacts with:59E,58D,60D

AA1 178G;Interacts with:60D,75H

AA1 179D;Interacts with:60D,75H

AA1 180W;Interacts with:62M,60D,61E,75H,57F,79F,76L

AA1 181T;Interacts with:60D

AA2 57F;Interacts with:180W

AA2 58D;Interacts with:176R

AA2 59E;Interacts with:176R

MUT2 E59D;157591;humsavar:VAR_058835;rs2308910;

AA2 60D;Interacts with:180W,179D,178G,176R,181T

AA2 61E;Interacts with:180W

AA2 62M;Interacts with:180W

MUT2 M62L;157592;humsavar:VAR_047685;rs2308911;

MUT2 M62K;157593;humsavar:VAR_058836;rs2308912;

MUT2 M62Q;157599;humsavar:VAR_058850;rs36013091;

AA2 75H;Interacts with:180W,179D,178G

AA2 76L;Interacts with:180W

AA2 79F;Interacts with:180W

//

MESH1 mesh:D001249

NAME1 Asthma

CLUSTER1 clust1

MESH2 mesh:D006526

NAME2 Hepatitis C

CLUSTER2 clust1

AC1 P01579

PFAM1 PF00714

PDB1 1FYH:A

AC2 P15260

PFAM2 PF07140

PDB2 1FYH:E

INT semi-edgetic

AA1 146A;Interacts with:186K,203A,205P,185Y

AA1 147A;Interacts with:183I,185Y

MUT1 A147L;PMID:1662603;

AA1 149T;Interacts with:185Y

AA1 150G;Interacts with:208S,205P,207S

AA1 151K;Interacts with:209L,207S,208S

MUT1 K151R;PMID:2127102;

MUT1 K151E;PMID:1830392;

AA2 183I;Interacts with:147A

AA2 185Y;Interacts with:146A,147A,149T

AA2 186K;Interacts with:146A

AA2 203A;Interacts with:146A

AA2 205P;Interacts with:150G,146A

AA2 207S;Interacts with:151K,150G

AA2 208S;Interacts with:150G,151K

AA2 209L;Interacts with:151K

//

MESH1 mesh:D001249

NAME1 Asthma

CLUSTER1 clust1

MESH2 mesh:D006526

NAME2 Hepatitis C

CLUSTER2 clust1

AC1 P01579

PFAM1 PF00714

PDB1 1FYH:A

AC2 P15260

PFAM2 PF01108

PDB2 1FYH:B

INT semi-edgetic

AA1 163R;Interacts with:66Y

AA2 66Y;Interacts with:163R

MUT2 Y66A;PMID:9878445;

MUT2 Y66F;PMID:9878445;

//

MESH1 mesh:D001249

NAME1 Asthma

CLUSTER1 clust1

MESH2 mesh:D006526

NAME2 Hepatitis C

CLUSTER2 clust1

AC1 P05362

PFAM1 PF03921

PDB1 1MQ8:A

AC2 P20701

PFAM2 PF00092

PDB2 1MQ8:B

INT edgetic

AA1 100Q;Interacts with:232N,268T,269D

MUT1 Q100T;PMID:1970514,PMID:1346257;

MUT1 Q100N;PMID:9452454;

MUT1 Q100H;PMID:1970514;

AA1 104K;Interacts with:228L

AA1 56K;Interacts with:270S

MUT1 K56E;PMID:1716769;

MUT1 K56M;159879;humsavar:VAR_010204;rs5491;clinvar:29700, phenotype=MALARIA, CEREBRAL, SUSCEPTIBILITY TO;dbSNP:rs5491, phenotype=MALARIA_CEREBRAL_SUSCEPTIBILITY_TO;

AA1 57L;Interacts with:268T,269D,270S

AA1 61E;Interacts with:166S,165M,230L,266E

MUT1 E61A;PMID:1970514,PMID:1346257,PMID:9452454;

AA1 62T;Interacts with:289H,166S,165M,266E

AA1 63P;Interacts with:168Q,289H,165M,166S,288K

AA1 64L;Interacts with:289H

MUT1 L64S;PMID:1346257;

AA1 65P;Interacts with:289H

AA1 66K;Interacts with:296Q,266E,289H

AA1 89Q;Interacts with:165M

AA1 91M;Interacts with:229L,228L,230L,165M

MUT1 M91I;dbSNP:rs140559142;

MUT1 M91T;COSMIC:272793;

AA1 93Y;Interacts with:230L,268T,269D

AA1 95N;Interacts with:268T,269D,270S,232N

AA2 165M;Interacts with:61E,62T,63P,89Q,91M

MUT2 M165A;PMID:9786897;

AA2 166S;Interacts with:61E,62T,63P

AA2 168Q;Interacts with:63P

MUT2 Q168A;PMID:9786897;

AA2 228L;Interacts with:91M,104K

AA2 229L;Interacts with:91M

AA2 230L;Interacts with:93Y,61E,91M

MUT2 L230A;PMID:9786897;

AA2 232N;Interacts with:100Q,95N

AA2 266E;Interacts with:62T,66K,61E

MUT2 E266A;PMID:9786897;

AA2 268T;Interacts with:93Y,57L,95N,100Q

MUT2 T268A;PMID:9786897;

AA2 269D;Interacts with:100Q,95N,93Y,57L

AA2 270S;Interacts with:56K,95N,57L

MUT2 S270A;PMID:9786897;

AA2 288K;Interacts with:63P

MUT2 K288A;PMID:9786897;

AA2 289H;Interacts with:62T,63P,64L,65P,66K

MUT2 H289A;PMID:9786897;

AA2 296Q;Interacts with:66K

//

MESH1 mesh:D001249

NAME1 Asthma

CLUSTER1 clust1

MESH2 mesh:D006969

NAME2 Hypersensitivity, Immediate

CLUSTER2 clust1

AC1 P13232

PFAM1 PF01415

PDB1 3DI2:A

AC2 P16871

PFAM2 PF00041

PDB2 3DI2:B

INT non-edgetic

AA1 103H;Interacts with:159Y

AA1 106K;Interacts with:159Y

AA1 35K;Interacts with:212Y

AA1 36Q;Interacts with:212Y

AA1 39S;Interacts with:211H,159Y,212Y,213F

AA1 40V;Interacts with:213F,212Y,159Y

AA1 43V;Interacts with:159Y,158K

AA1 44S;Interacts with:159Y

AA1 47Q;Interacts with:158K

AA2 158K;Interacts with:43V,47Q

AA2 159Y;Interacts with:39S,103H,43V,106K,40V,44S

AA2 211H;Interacts with:39S

AA2 212Y;Interacts with:40V,36Q,39S,35K

AA2 213F;Interacts with:40V,39S

//

MESH1 mesh:D001249

NAME1 Asthma

CLUSTER1 clust1

MESH2 mesh:D006969

NAME2 Hypersensitivity, Immediate

CLUSTER2 clust1

AC1 P31785

PFAM1 PF00041

PDB1 2B5I:C

AC2 P60568

PFAM2 PF00715

PDB2 2B5I:C

INT non-edgetic

AA1 181H;Interacts with:31Q,150S,149I,35E

AA1 190R;Interacts with:125C,127Y,81E

AA1 195H;Interacts with:65Y,127Y

AA1 197W;Interacts with:125C,126E,124M

AA1 220T;Interacts with:124M

AA1 222R;Interacts with:124M,126E

MUT1 R222C;160917;humsavar:VAR_002688, phenotype=X-linked combined immunodeficiency (XCID);

AA1 228N;Interacts with:146Q,42Q

AA1 229P;Interacts with:38L,146Q,42Q

AA1 230L;Interacts with:38L,35E,150S,149I,146Q,147S,39L,42Q

MUT1 L230P;884416;humsavar:VAR_002693, phenotype=Severe combined immunodeficiency X-linked T-cell-negative/B-cell-positive/NK-cell-negative (XSCID);

AA1 231C;Interacts with:147S,146Q,150S

AA1 232G;Interacts with:42Q,146Q

AA2 124M;Interacts with:222R,197W,220T

AA2 125C;Interacts with:190R,197W

AA2 126E;Interacts with:197W,222R

AA2 127Y;Interacts with:195H,190R

AA2 146Q;Interacts with:232G,229P,230L,231C,228N

AA2 147S;Interacts with:231C,230L

AA2 149I;Interacts with:230L,181H

AA2 150S;Interacts with:230L,181H,231C

AA2 31Q;Interacts with:181H

AA2 35E;Interacts with:230L,181H

AA2 38L;Interacts with:230L,229P

MUT2 L38R;dbSNP:rs3087209;

AA2 39L;Interacts with:230L

AA2 42Q;Interacts with:232G,229P,230L,228N

AA2 65Y;Interacts with:195H

AA2 81E;Interacts with:190R

//

MESH1 mesh:D001249

NAME1 Asthma

CLUSTER1 clust1

MESH2 mesh:D006969

NAME2 Hypersensitivity, Immediate

CLUSTER2 clust1

AC1 P31785

PFAM1 PF09240

PDB1 2B5I:C

AC2 P60568

PFAM2 PF00715

PDB2 2B5I:C

INT non-edgetic

AA1 125Y;Interacts with:143T,146Q,147S,150S,140R

MUT1 Y125N;884404;humsavar:VAR_002679, phenotype=Severe combined immunodeficiency X-linked T-cell-negative/B-cell-positive/NK-cell-negative (XSCID);

AA1 147K;Interacts with:139N

AA1 149Q;Interacts with:143T,146Q,139N

AA1 150N;Interacts with:146Q,139N

AA1 92K;Interacts with:130E

AA1 93N;Interacts with:130E,68K

AA1 95D;Interacts with:69K

AA2 130E;Interacts with:93N,92K

AA2 139N;Interacts with:147K,149Q,150N

AA2 140R;Interacts with:125Y

AA2 143T;Interacts with:125Y,149Q

AA2 146Q;Interacts with:150N,125Y,149Q

AA2 147S;Interacts with:125Y

AA2 150S;Interacts with:125Y

AA2 68K;Interacts with:93N

AA2 69K;Interacts with:95D

//

MESH1 mesh:D001249

NAME1 Asthma

CLUSTER1 clust1

MESH2 mesh:D006969

NAME2 Hypersensitivity, Immediate

CLUSTER2 clust1

AC1 P05112

PFAM1 PF00727

PDB1 3BPL:A

AC2 P31785

PFAM2 PF00041

PDB2 3BPL:C

INT non-edgetic

AA1 141K;Interacts with:229P

MUT1 K141D;PMID:9799097;

MUT1 K141Q;PMID:8262056,PMID:7517357;

AA1 145R;Interacts with:228N,230L,231C,232G,229P

MUT1 R145D;PMID:8262056,PMID:7517357,PMID:9799097;

AA1 146E;Interacts with:231C

AA1 148Y;Interacts with:230L,181H,231C,204Y

MUT1 Y148H;PMID:1387082,PMID:7517357;

MUT1 Y148D;PMID:1864379,PMID:1387082,PMID:8262056,PMID:,PMID:7957181,PMID:7517357,PMID:8151703,PMID:9799097;

MUT1 Y148G;PMID:1387082,PMID:,PMID:7517357,PMID:8151703;

AA1 149S;Interacts with:181H,227F

AA1 32Q;Interacts with:230L,204Y

AA1 35I;Interacts with:230L

AA1 39N;Interacts with:230L,229P

AA2 181H;Interacts with:148Y,149S

AA2 204Y;Interacts with:32Q,148Y

AA2 227F;Interacts with:149S

MUT2 F227C;884415;humsavar:VAR_002692, phenotype=Severe combined immunodeficiency X-linked T-cell-negative/B-cell-positive/NK-cell-negative (XSCID);

AA2 228N;Interacts with:145R

AA2 229P;Interacts with:39N,145R,141K

AA2 230L;Interacts with:39N,32Q,148Y,35I,145R

MUT2 L230P;884416;humsavar:VAR_002693, phenotype=Severe combined immunodeficiency X-linked T-cell-negative/B-cell-positive/NK-cell-negative (XSCID);

AA2 231C;Interacts with:146E,145R,148Y

AA2 232G;Interacts with:145R

//

MESH1 mesh:D001249

NAME1 Asthma

CLUSTER1 clust1

MESH2 mesh:D006969

NAME2 Hypersensitivity, Immediate

CLUSTER2 clust1

AC1 P05112

PFAM1 PF00727

PDB1 3BPL:A

AC2 P31785

PFAM2 PF09240

PDB2 3BPL:C

INT non-edgetic

AA1 138E;Interacts with:147K,149Q

MUT1 E138R;PMID:9799097;

MUT1 E138Q;PMID:8262056,PMID:7517357;

AA1 139R;Interacts with:93N,125Y

AA1 141K;Interacts with:150N

AA1 142T;Interacts with:125Y,147K,149Q

MUT1 T142M;dbSNP:rs145068648;dbSNP:rs145068648;

AA1 145R;Interacts with:149Q,125Y,150N

MUT1 R145D;PMID:8262056,PMID:7517357,PMID:9799097;

AA1 146E;Interacts with:126Q,125Y

AA1 149S;Interacts with:125Y,67V,124L

AA2 124L;Interacts with:149S

AA2 125Y;Interacts with:142T,149S,145R,146E,139R

MUT2 Y125N;884404;humsavar:VAR_002679, phenotype=Severe combined immunodeficiency X-linked T-cell-negative/B-cell-positive/NK-cell-negative (XSCID);

AA2 126Q;Interacts with:146E

AA2 147K;Interacts with:142T,138E

AA2 149Q;Interacts with:145R,142T,138E

AA2 150N;Interacts with:145R,141K

AA2 67V;Interacts with:149S

AA2 93N;Interacts with:139R

//

MESH1 mesh:D001249

NAME1 Asthma

CLUSTER1 clust1

MESH2 mesh:D006969

NAME2 Hypersensitivity, Immediate

CLUSTER2 clust1

AC1 P05112

PFAM1 PF00727

PDB1 3BPL:A

AC2 P24394

PFAM2 PF09238

PDB2 3BPL:B

INT semi-edgetic

AA1 105R;Interacts with:92D,91D

MUT1 R105E;PMID:7517357;

AA1 109R;Interacts with:94V,93V,92D

MUT1 R109Q;dbSNP:rs79908535;dbSNP:rs79908535;

AA1 112R;Interacts with:96A,97D,66F,94V,92D

MUT1 R112Q;PMID:7682108,PMID:8262056,PMID:7517357,PMID:7485389;

MUT1 R112T;PMID:7682108;

MUT1 R112D;PMID:8262056,PMID:7517357;

AA1 113N;Interacts with:96A,97D,94V,95S

AA1 115W;Interacts with:66F,67L,65V

MUT1 W115R;PMID:1864379,PMID:7517357;

AA1 116G;Interacts with:96A

AA1 29I;Interacts with:95S

MUT1 I29A;PMID:9799097;

AA1 30T;Interacts with:95S,96A

AA1 33E;Interacts with:94V,95S,96A

MUT1 E33A;PMID:11526337;

AA1 80Y;Interacts with:68L

AA2 65V;Interacts with:115W

AA2 66F;Interacts with:115W,112R

MUT2 F66A;884477;

AA2 67L;Interacts with:115W

MUT2 L67A;884478;

AA2 68L;Interacts with:80Y

MUT2 L68A;884479;

AA2 91D;Interacts with:105R

MUT2 D91A;884480;

AA2 92D;Interacts with:105R,112R,109R

MUT2 D92A;884481;

AA2 93V;Interacts with:109R

MUT2 V93A;884482;

AA2 94V;Interacts with:33E,109R,113N,112R

MUT2 V94A;884483;

AA2 95S;Interacts with:33E,30T,113N,29I

MUT2 S95A;884484;

AA2 96A;Interacts with:113N,112R,116G,30T,33E

AA2 97D;Interacts with:113N,112R

MUT2 D97N;1369005;

MUT2 D97A;1369004;

//

MESH1 mesh:D001249

NAME1 Asthma

CLUSTER1 clust1

MESH2 mesh:D008180

NAME2 Systemic lupus erythematosus

CLUSTER2 clust1

AC1 P29459

PFAM1 PF03039

PDB1 3HMX:B

AC2 P29460

PFAM2 PF10420

PDB2 3HMX:A

INT non-edgetic

AA1 205R;Interacts with:203E

AA1 208T;Interacts with:203E,202A

AA1 211R;Interacts with:136Y

AA1 71H;Interacts with:205S

AA1 76K;Interacts with:205S

AA1 82V;Interacts with:201A,202A,203E

AA1 83E;Interacts with:201A,202A

AA1 85C;Interacts with:202A

AA1 86L;Interacts with:202A,200P,201A

AA2 136Y;Interacts with:211R

AA2 200P;Interacts with:86L

AA2 201A;Interacts with:82V,83E,86L

AA2 202A;Interacts with:85C,82V,86L,83E,208T

AA2 203E;Interacts with:208T,82V,205R

AA2 205S;Interacts with:71H,76K

//

MESH1 mesh:D001249

NAME1 Asthma

CLUSTER1 clust1

MESH2 mesh:D008180

NAME2 Systemic lupus erythematosus

CLUSTER2 clust1

AC1 O95150

PFAM1 PF00229

PDB1 3K51:A

AC2 O95407

PFAM2 PF00020

PDB2 3K51:B

INT semi-edgetic

AA1 186D;Interacts with:84Y

AA1 187S;Interacts with:83N,84Y,85L

AA1 188Y;Interacts with:81F,85L,83N,89R,79T,80Q

AA1 189P;Interacts with:82W

AA1 190E;Interacts with:89R

AA1 191P;Interacts with:89R

AA1 192T;Interacts with:89R

AA1 221Q;Interacts with:86E

AA2 79T;Interacts with:188Y

AA2 80Q;Interacts with:188Y

AA2 81F;Interacts with:188Y

AA2 82W;Interacts with:189P

AA2 83N;Interacts with:187S,188Y

AA2 84Y;Interacts with:187S,186D

AA2 85L;Interacts with:188Y,187S

AA2 86E;Interacts with:221Q

AA2 89R;Interacts with:190E,192T,188Y,191P

//

MESH1 mesh:D001249

NAME1 Asthma

CLUSTER1 clust1

MESH2 mesh:D011658

NAME2 Pulmonary Fibrosis

CLUSTER2 clust1

AC1 O14786

PFAM1 PF00754

PDB1 4DEQ:A

AC2 P15692

PFAM2 PF00754

PDB2 4DEQ:B

INT semi-edgetic

AA1 319E;Interacts with:138R

AA1 323R;Interacts with:92L,45D,90E,91G

AA1 324E;Interacts with:138R

AA2 138R;Interacts with:324E,319E

AA2 45D;Interacts with:323R

AA2 90E;Interacts with:323R

AA2 91G;Interacts with:323R

AA2 92L;Interacts with:323R

//

MESH1 mesh:D001249

NAME1 Asthma

CLUSTER1 clust1

MESH2 mesh:D011658

NAME2 Pulmonary Fibrosis

CLUSTER2 clust1

AC1 O14786

PFAM1 PF00754

PDB1 4DEQ:B

AC2 P15692

PFAM2 PF14554

PDB2 4DEQ:A

INT semi-edgetic

AA1 297Y;Interacts with:231R,232R,230P

AA1 298S;Interacts with:219E,222E

AA1 299T;Interacts with:222E,219E

AA1 300N;Interacts with:210D,219E,231R

AA1 301W;Interacts with:219E,232R

AA1 316T;Interacts with:232R

AA1 320D;Interacts with:232R

AA1 348E;Interacts with:214K,231R

AA1 349T;Interacts with:231R,232R

AA1 353Y;Interacts with:232R

AA1 413T;Interacts with:232R

AA2 210D;Interacts with:300N

MUT2 D210H;971365;

AA2 214K;Interacts with:348E

AA2 219E;Interacts with:298S,301W,300N,299T

AA2 222E;Interacts with:298S,299T

AA2 230P;Interacts with:297Y

AA2 231R;Interacts with:297Y,349T,300N,348E

AA2 232R;Interacts with:320D,297Y,316T,301W,413T,349T,353Y

//

MESH1 mesh:D001249

NAME1 Asthma

CLUSTER1 clust1

MESH2 mesh:D014376

NAME2 Tuberculosis

CLUSTER2 clust1

AC1 P29459

PFAM1 PF03039

PDB1 3HMX:B

AC2 P29460

PFAM2 PF10420

PDB2 3HMX:A

INT non-edgetic

AA1 205R;Interacts with:203E

AA1 208T;Interacts with:203E,202A

AA1 211R;Interacts with:136Y

AA1 71H;Interacts with:205S

AA1 76K;Interacts with:205S

AA1 82V;Interacts with:201A,202A,203E

AA1 83E;Interacts with:201A,202A

AA1 85C;Interacts with:202A

AA1 86L;Interacts with:202A,200P,201A

AA2 136Y;Interacts with:211R

AA2 200P;Interacts with:86L

AA2 201A;Interacts with:82V,83E,86L

AA2 202A;Interacts with:85C,82V,86L,83E,208T

AA2 203E;Interacts with:208T,82V,205R

AA2 205S;Interacts with:71H,76K

//

MESH1 mesh:D001249

NAME1 Asthma

CLUSTER1 clust1

MESH2 mesh:D014376

NAME2 Tuberculosis

CLUSTER2 clust1

AC1 P01579

PFAM1 PF00714

PDB1 1FYH:A

AC2 P15260

PFAM2 PF07140

PDB2 1FYH:E

INT semi-edgetic

AA1 146A;Interacts with:186K,203A,205P,185Y

AA1 147A;Interacts with:183I,185Y

MUT1 A147L;PMID:1662603;

AA1 149T;Interacts with:185Y

AA1 150G;Interacts with:208S,205P,207S

AA1 151K;Interacts with:209L,207S,208S

MUT1 K151R;PMID:2127102;

MUT1 K151E;PMID:1830392;

AA2 183I;Interacts with:147A

AA2 185Y;Interacts with:146A,147A,149T

AA2 186K;Interacts with:146A

AA2 203A;Interacts with:146A

AA2 205P;Interacts with:150G,146A

AA2 207S;Interacts with:151K,150G

AA2 208S;Interacts with:150G,151K

AA2 209L;Interacts with:151K

//

MESH1 mesh:D001249

NAME1 Asthma

CLUSTER1 clust1

MESH2 mesh:D014376

NAME2 Tuberculosis

CLUSTER2 clust1

AC1 P01579

PFAM1 PF00714

PDB1 1FYH:A

AC2 P15260

PFAM2 PF01108

PDB2 1FYH:B

INT semi-edgetic

AA1 163R;Interacts with:66Y

AA2 66Y;Interacts with:163R

MUT2 Y66A;PMID:9878445;

MUT2 Y66F;PMID:9878445;

//

MESH1 mesh:D001249

NAME1 Asthma

CLUSTER1 clust1

MESH2 mesh:D014376

NAME2 Tuberculosis

CLUSTER2 clust1

AC1 P05362

PFAM1 PF03921

PDB1 1MQ8:A

AC2 P20701

PFAM2 PF00092

PDB2 1MQ8:B

INT edgetic

AA1 100Q;Interacts with:232N,268T,269D

MUT1 Q100T;PMID:1970514,PMID:1346257;

MUT1 Q100N;PMID:9452454;

MUT1 Q100H;PMID:1970514;

AA1 104K;Interacts with:228L

AA1 56K;Interacts with:270S

MUT1 K56E;PMID:1716769;

MUT1 K56M;159879;humsavar:VAR_010204;rs5491;clinvar:29700, phenotype=MALARIA, CEREBRAL, SUSCEPTIBILITY TO;dbSNP:rs5491, phenotype=MALARIA_CEREBRAL_SUSCEPTIBILITY_TO;

AA1 57L;Interacts with:268T,269D,270S

AA1 61E;Interacts with:166S,165M,230L,266E

MUT1 E61A;PMID:1970514,PMID:1346257,PMID:9452454;

AA1 62T;Interacts with:289H,166S,165M,266E

AA1 63P;Interacts with:168Q,289H,165M,166S,288K

AA1 64L;Interacts with:289H

MUT1 L64S;PMID:1346257;

AA1 65P;Interacts with:289H

AA1 66K;Interacts with:296Q,266E,289H

AA1 89Q;Interacts with:165M

AA1 91M;Interacts with:229L,228L,230L,165M

MUT1 M91I;dbSNP:rs140559142;

MUT1 M91T;COSMIC:272793;

AA1 93Y;Interacts with:230L,268T,269D

AA1 95N;Interacts with:268T,269D,270S,232N

AA2 165M;Interacts with:61E,62T,63P,89Q,91M

MUT2 M165A;PMID:9786897;

AA2 166S;Interacts with:61E,62T,63P

AA2 168Q;Interacts with:63P

MUT2 Q168A;PMID:9786897;

AA2 228L;Interacts with:91M,104K

AA2 229L;Interacts with:91M

AA2 230L;Interacts with:93Y,61E,91M

MUT2 L230A;PMID:9786897;

AA2 232N;Interacts with:100Q,95N

AA2 266E;Interacts with:62T,66K,61E

MUT2 E266A;PMID:9786897;

AA2 268T;Interacts with:93Y,57L,95N,100Q

MUT2 T268A;PMID:9786897;

AA2 269D;Interacts with:100Q,95N,93Y,57L

AA2 270S;Interacts with:56K,95N,57L

MUT2 S270A;PMID:9786897;

AA2 288K;Interacts with:63P

MUT2 K288A;PMID:9786897;

AA2 289H;Interacts with:62T,63P,64L,65P,66K

MUT2 H289A;PMID:9786897;

AA2 296Q;Interacts with:66K

//

MESH1 mesh:D001249

NAME1 Asthma

CLUSTER1 clust1

MESH2 mesh:D029424

NAME2 Pulmonary Disease, Chronic Obstructive

CLUSTER2 clust1

AC1 P17693

PFAM1 PF07654

PDB1 2DYP:A

AC2 Q8N423

PFAM2 PF13895

PDB2 2DYP:D

INT non-edgetic

AA1 218V;Interacts with:71T

AA1 219F;Interacts with:70I,71T,78V

AA1 220D;Interacts with:59R

AA1 221Y;Interacts with:59R,60L,61Y

AA1 222E;Interacts with:67A,66S,62R

AA1 224T;Interacts with:66S

AA1 250Q;Interacts with:64K

AA1 251D;Interacts with:64K

AA1 253E;Interacts with:64K,65K

AA1 272V;Interacts with:65K,64K,66S

AA2 59R;Interacts with:221Y,220D

MUT2 R59H;dbSNP:rs141797988;dbSNP:rs141797988;

AA2 60L;Interacts with:221Y

AA2 61Y;Interacts with:221Y

AA2 62R;Interacts with:222E

AA2 64K;Interacts with:251D,253E,272V,250Q

AA2 65K;Interacts with:272V,253E

AA2 66S;Interacts with:222E,224T,272V

AA2 67A;Interacts with:222E

AA2 70I;Interacts with:219F

AA2 71T;Interacts with:218V,219F

AA2 78V;Interacts with:219F

//

MESH1 mesh:D001249

NAME1 Asthma

CLUSTER1 clust1

MESH2 mesh:D029424

NAME2 Pulmonary Disease, Chronic Obstructive

CLUSTER2 clust1

AC1 P01137

PFAM1 PF00019

PDB1 3KFD:A

AC2 P37173

PFAM2 PF08917

PDB2 3KFD:E

INT semi-edgetic

AA1 303R;Interacts with:74T,142E

AA1 309K;Interacts with:74T,141D,50L,142E

AA1 310W;Interacts with:50L,74T,76I

AA1 312H;Interacts with:72S,73I,74T

AA1 368Y;Interacts with:76I

AA1 369Y;Interacts with:76I,74T,75S,73I

AA1 370V;Interacts with:76I,75S,78E

AA1 371G;Interacts with:75S,76I,53F,78E

AA1 372R;Interacts with:78E,75S,108Y,55D,53F,98E

AA1 373K;Interacts with:78E

AA2 108Y;Interacts with:372R

AA2 141D;Interacts with:309K

AA2 142E;Interacts with:303R,309K

AA2 50L;Interacts with:310W,309K

MUT2 L50Y;PMID;

AA2 53F;Interacts with:371G,372R

AA2 55D;Interacts with:372R

AA2 72S;Interacts with:312H

AA2 73I;Interacts with:312H,369Y

MUT2 I73V;960937;humsavar:VAR_036070, phenotype=A colorectal cancer sample;

AA2 74T;Interacts with:303R,369Y,309K,310W,312H

AA2 75S;Interacts with:371G,372R,370V,369Y

AA2 76I;Interacts with:369Y,370V,368Y,371G,310W

AA2 78E;Interacts with:372R,373K,370V,371G

AA2 98E;Interacts with:372R

//

MESH1 mesh:D001249

NAME1 Asthma

CLUSTER1 clust1

MESH2 mesh:D029424

NAME2 Pulmonary Disease, Chronic Obstructive

CLUSTER2 clust1

AC1 P08254

PFAM1 PF00413

PDB1 1OO9:A

AC2 P01033

PFAM2 PF00965

PDB2 1OO9:B

INT semi-edgetic

AA1 171F;Interacts with:57L

AA1 172Y;Interacts with:58Y,87P,92V,88A,57L,89M

AA1 178G;Interacts with:27V

AA1 179N;Interacts with:26C,29P,28P,27V,122C,120T

AA1 180V;Interacts with:120T,26C,27V,25T

AA1 181L;Interacts with:26C,27V,25T

AA1 182A;Interacts with:24C,25T

AA1 183H;Interacts with:24C,92V,91S

AA1 184A;Interacts with:91S,92V,24C

AA1 185Y;Interacts with:92V,91S

AA1 186A;Interacts with:89M,91S

AA1 207T;Interacts with:157S,158I,156L

AA1 208T;Interacts with:156L,157S

AA1 209G;Interacts with:156L,157S

AA1 210T;Interacts with:156L

MUT1 T210Y;PMID:10871619;

AA1 215V;Interacts with:25T

AA1 218H;Interacts with:25T,24C

AA1 219E;Interacts with:25T,24C

AA1 222H;Interacts with:90E,24C,91S

AA1 227F;Interacts with:89M

AA1 228H;Interacts with:91S,24C,121T,90E,94G

AA1 229S;Interacts with:90E

AA1 238P;Interacts with:27V,24C,25T,26C

AA1 239L;Interacts with:26C,121T,25T,28P

AA1 240Y;Interacts with:27V,25T,26C,156L,173Q

AA1 241H;Interacts with:173Q,179E,177G,176Q

AA1 242S;Interacts with:173Q

AA1 244T;Interacts with:176Q

AA1 245D;Interacts with:176Q

AA2 120T;Interacts with:180V,179N

AA2 121T;Interacts with:239L,228H

AA2 122C;Interacts with:179N

AA2 156L;Interacts with:240Y,209G,208T,210T,207T

AA2 157S;Interacts with:209G,207T,208T

AA2 158I;Interacts with:207T

MUT2 I158V;dbSNP:rs1803571;

AA2 173Q;Interacts with:241H,242S,240Y

AA2 176Q;Interacts with:241H,245D,244T

AA2 177G;Interacts with:241H

AA2 179E;Interacts with:241H

AA2 24C;Interacts with:222H,228H,238P,182A,183H,218H,219E,184A

MUT2 C24S;PMID:9774703;

AA2 25T;Interacts with:240Y,218H,181L,238P,215V,182A,239L,219E,180V

MUT2 T25Q;1375658;

MUT2 T25K;1375657;

MUT2 T25V;961993;

MUT2 T25G;1375656;

MUT2 T25E;1375655;

MUT2 T25R;1375659;

AA2 26C;Interacts with:179N,239L,181L,180V,238P,240Y

AA2 27V;Interacts with:240Y,238P,178G,181L,179N,180V

AA2 28P;Interacts with:179N,239L

MUT2 P28A;PMID:9774703;

AA2 29P;Interacts with:179N

AA2 57L;Interacts with:171F,172Y

AA2 58Y;Interacts with:172Y

AA2 87P;Interacts with:172Y

AA2 88A;Interacts with:172Y

AA2 89M;Interacts with:186A,172Y,227F

AA2 90E;Interacts with:222H,228H,229S

AA2 91S;Interacts with:228H,184A,222H,186A,185Y,183H

AA2 92V;Interacts with:185Y,172Y,183H,184A

AA2 94G;Interacts with:228H

//

MESH1 mesh:D001249

NAME1 Asthma

CLUSTER1 clust1

MESH2 mesh:D029424

NAME2 Pulmonary Disease, Chronic Obstructive

CLUSTER2 clust1

AC1 Q13241

PFAM1 PF00059

PDB1 3CDG:E

AC2 P13747

PFAM2 PF00129

PDB2 3CDG:C

INT semi-edgetic

AA1 112Q;Interacts with:94I,173E

AA1 113Q;Interacts with:86R,90D,94I

AA1 114F;Interacts with:94I,90D,97V,93Q

AA1 143S;Interacts with:100R

MUT1 S143*;COSMIC:1235125;

AA1 146T;Interacts with:100R

AA1 160N;Interacts with:100R

AA1 161A;Interacts with:100R

AA1 162L;Interacts with:93Q,97V,96R

AA1 163D;Interacts with:96R,100R

AA1 164E;Interacts with:93Q,96R

AA1 169K;Interacts with:89R,93Q

AA1 170N;Interacts with:93Q

AA1 171R;Interacts with:86R,90D

AA1 78E;Interacts with:86R

AA1 79Q;Interacts with:86R,90D,89R

AA2 100R;Interacts with:161A,160N,143S,146T,163D

AA2 173E;Interacts with:112Q

AA2 86R;Interacts with:113Q,171R,79Q,78E

AA2 89R;Interacts with:79Q,169K

AA2 90D;Interacts with:114F,171R,113Q,79Q

AA2 93Q;Interacts with:164E,162L,170N,114F,169K

AA2 94I;Interacts with:114F,112Q,113Q

AA2 96R;Interacts with:164E,163D,162L

AA2 97V;Interacts with:114F,162L

//

MESH1 mesh:D001249

NAME1 Asthma

CLUSTER1 clust1

MESH2 mesh:D029424

NAME2 Pulmonary Disease, Chronic Obstructive

CLUSTER2 clust1

AC1 P17693

PFAM1 PF07654

PDB1 1YDP:A

AC2 P61769

PFAM2 PF07654

PDB2 1YDP:B

INT semi-edgetic

AA1 256E;Interacts with:49G,48S

AA1 257T;Interacts with:46Y

AA1 258R;Interacts with:46Y,31S

AA1 259P;Interacts with:44N,85L,46Y

AA1 260A;Interacts with:32R,85L,44N,46Y

AA1 261G;Interacts with:85L,32R,44N

AA1 262D;Interacts with:33H,32R

AA1 266Q;Interacts with:32R,31S,44N,33H,46Y

AA2 31S;Interacts with:266Q,258R

AA2 32R;Interacts with:260A,266Q,261G,262D

MUT2 R32C;dbSNP:rs11553032;

AA2 33H;Interacts with:262D,266Q

AA2 44N;Interacts with:259P,260A,266Q,261G

AA2 46Y;Interacts with:258R,257T,266Q,259P,260A

AA2 48S;Interacts with:256E

AA2 49G;Interacts with:256E

AA2 85L;Interacts with:260A,261G,259P

//

MESH1 mesh:D001249

NAME1 Asthma

CLUSTER1 clust1

MESH2 mesh:D029424

NAME2 Pulmonary Disease, Chronic Obstructive

CLUSTER2 clust1

AC1 P26715

PFAM1 PF00059

PDB1 3CDG:F

AC2 P13747

PFAM2 PF00129

PDB2 3CDG:C

INT edgetic

AA1 137R;Interacts with:172S,175E,169N,170D,152R

AA1 171P;Interacts with:176H

AA1 172S;Interacts with:176H

AA1 199K;Interacts with:183D,187E

AA1 212Q;Interacts with:183D,175E,176H,178R

AA1 213V;Interacts with:176H

AA1 217K;Interacts with:183D,175E,179A,178R,182E

AA1 218S;Interacts with:178R

AA1 223S;Interacts with:152R,178R

MUT1 S223T;dbSNP:rs144003798;

AA1 224S;Interacts with:152R,175E

AA1 226I;Interacts with:172S

AA2 152R;Interacts with:224S,223S,137R

AA2 169N;Interacts with:137R

AA2 170D;Interacts with:137R

AA2 172S;Interacts with:137R,226I

AA2 175E;Interacts with:137R,212Q,217K,224S

AA2 176H;Interacts with:171P,172S,213V,212Q

AA2 178R;Interacts with:223S,218S,212Q,217K

MUT2 R178G;879592;dbSNP:rs41562314;humsavar:VAR_016652;rs41562314;

AA2 179A;Interacts with:217K

AA2 182E;Interacts with:217K

AA2 183D;Interacts with:217K,212Q,199K

AA2 187E;Interacts with:199K

//

MESH1 mesh:D001249

NAME1 Asthma

CLUSTER1 clust1

MESH2 mesh:D050197

NAME2 Atherosclerosis

CLUSTER2 clust1

AC1 O14786

PFAM1 PF00754

PDB1 4DEQ:A

AC2 P15692

PFAM2 PF00754

PDB2 4DEQ:B

INT non-edgetic

AA1 319E;Interacts with:138R

AA1 323R;Interacts with:92L,45D,90E,91G

AA1 324E;Interacts with:138R

AA2 138R;Interacts with:324E,319E

AA2 45D;Interacts with:323R

AA2 90E;Interacts with:323R

AA2 91G;Interacts with:323R

AA2 92L;Interacts with:323R

//

MESH1 mesh:D001249

NAME1 Asthma

CLUSTER1 clust1

MESH2 mesh:D050197

NAME2 Atherosclerosis

CLUSTER2 clust1

AC1 O14786

PFAM1 PF00754

PDB1 4DEQ:B

AC2 P15692

PFAM2 PF14554

PDB2 4DEQ:A

INT non-edgetic

AA1 297Y;Interacts with:231R,232R,230P

AA1 298S;Interacts with:219E,222E

AA1 299T;Interacts with:222E,219E

AA1 300N;Interacts with:210D,219E,231R

AA1 301W;Interacts with:219E,232R

AA1 316T;Interacts with:232R

AA1 320D;Interacts with:232R

AA1 348E;Interacts with:214K,231R

AA1 349T;Interacts with:231R,232R

AA1 353Y;Interacts with:232R

AA1 413T;Interacts with:232R

AA2 210D;Interacts with:300N

MUT2 D210H;971365;

AA2 214K;Interacts with:348E

AA2 219E;Interacts with:298S,301W,300N,299T

AA2 222E;Interacts with:298S,299T

AA2 230P;Interacts with:297Y

AA2 231R;Interacts with:297Y,349T,300N,348E

AA2 232R;Interacts with:320D,297Y,316T,301W,413T,349T,353Y

//

MESH1 mesh:D002277

NAME1 Carcinoma

CLUSTER1 clust2

MESH2 mesh:D006528

NAME2 Carcinoma, Hepatocellular

CLUSTER2 clust2

AC1 Q13794

PFAM1 PF15150

PDB1 3MQP:B

AC2 Q16548

PFAM2 PF00452

PDB2 3MQP:B

INT non-edgetic

AA1 21L;Interacts with:55C,59V,58N

AA1 22E;Interacts with:74V,73Q

MUT1 E22K;dbSNP:rs1126436;

AA1 24E;Interacts with:52L

AA1 25C;Interacts with:74V,52L

AA1 26A;Interacts with:77K,78E,74V

AA1 28Q;Interacts with:51N

AA1 29L;Interacts with:91T,48V,74V,78E,95F

MUT1 L29A;823237;

MUT1 L29E;823238;

AA1 30R;Interacts with:78E,81D,77K,80E,88R,91T

MUT1 R30delR;COSMIC:166155;

AA1 32F;Interacts with:47E,91T,48V

MUT1 F32I;823240;

MUT1 F32E;823239;

AA1 33G;Interacts with:91T,88R,85N,87G,78E

AA1 34D;Interacts with:87G,85N,88R

AA1 36L;Interacts with:87G,44V,40V,90V

MUT1 L36E;823242;

AA1 37N;Interacts with:86W,87G,88R,85N

AA2 40V;Interacts with:36L

AA2 44V;Interacts with:36L

AA2 47E;Interacts with:32F

AA2 48V;Interacts with:32F,29L

AA2 51N;Interacts with:28Q

AA2 52L;Interacts with:25C,24E

AA2 55C;Interacts with:21L

AA2 58N;Interacts with:21L

MUT2 N58S;dbSNP:rs141166047;dbSNP:rs141166047;

AA2 59V;Interacts with:21L

AA2 73Q;Interacts with:22E

AA2 74V;Interacts with:25C,29L,26A,22E

AA2 77K;Interacts with:26A,30R

AA2 78E;Interacts with:26A,30R,29L,33G

AA2 80E;Interacts with:30R

AA2 81D;Interacts with:30R

AA2 85N;Interacts with:34D,33G,37N

AA2 86W;Interacts with:37N

AA2 87G;Interacts with:36L,34D,37N,33G

AA2 88R;Interacts with:33G,37N,30R,34D

AA2 90V;Interacts with:36L

AA2 91T;Interacts with:29L,32F,33G,30R

AA2 95F;Interacts with:29L

//

MESH1 mesh:D002277

NAME1 Carcinoma

CLUSTER1 clust2

MESH2 mesh:D006528

NAME2 Carcinoma, Hepatocellular

CLUSTER2 clust2

AC1 P55957

PFAM1 PF06393

PDB1 1ZY3:B

AC2 Q92843

PFAM2 PF00452

PDB2 1ZY3:A

INT non-edgetic

AA1 100S;Interacts with:56R

AA1 82I;Interacts with:85E

AA1 83I;Interacts with:71T,82V,85E

AA1 84R;Interacts with:88Q,86L,85E

MUT1 R84W;dbSNP:rs148107209;dbSNP:rs148107209;

AA1 86I;Interacts with:82V,71T,68L

AA1 87A;Interacts with:95R,85E,82V,86L

AA1 88R;Interacts with:86L

AA1 89H;Interacts with:68L,67Q

AA1 90L;Interacts with:67Q,64L,98A,102F,83S,68L,82V,95R

AA1 91A;Interacts with:95R,86L

AA1 93V;Interacts with:57F,64L

AA1 94G;Interacts with:57F

MUT1 G94E;PMID:11121101;

AA1 95D;Interacts with:92N,93W,95R,94G

AA1 97M;Interacts with:57F,56R

AA1 98D;Interacts with:53F,56R

AA1 99R;Interacts with:52E

AA2 102F;Interacts with:90L

AA2 52E;Interacts with:99R

AA2 53F;Interacts with:98D

AA2 56R;Interacts with:98D,100S,97M

AA2 57F;Interacts with:97M,93V,94G

AA2 64L;Interacts with:90L,93V

AA2 67Q;Interacts with:90L,89H

AA2 68L;Interacts with:89H,90L,86I

AA2 71T;Interacts with:83I,86I

AA2 82V;Interacts with:86I,83I,90L,87A

AA2 83S;Interacts with:90L

AA2 85E;Interacts with:82I,87A,83I,84R

AA2 86L;Interacts with:84R,88R,91A,87A

AA2 88Q;Interacts with:84R

AA2 92N;Interacts with:95D

AA2 93W;Interacts with:95D

AA2 94G;Interacts with:95D

AA2 95R;Interacts with:91A,87A,95D,90L

AA2 98A;Interacts with:90L

//

MESH1 mesh:D002277

NAME1 Carcinoma

CLUSTER1 clust2

MESH2 mesh:D006528

NAME2 Carcinoma, Hepatocellular

CLUSTER2 clust2

AC1 P10415

PFAM1 PF00452

PDB1 4AQ3:A

AC2 Q07817

PFAM2 PF00452

PDB2 4AQ3:C

INT semi-edgetic

AA1 102D;Interacts with:100R

AA1 103D;Interacts with:100R,99L,97F

AA1 104F;Interacts with:96E

AA1 106R;Interacts with:96E

AA1 107R;Interacts with:95D,96E,92E,91R

AA1 108Y;Interacts with:92E

AA1 115M;Interacts with:117G

AA1 118Q;Interacts with:116P,115T,117G

AA1 119L;Interacts with:117G

AA1 120H;Interacts with:115T

AA1 129R;Interacts with:117G

MUT1 R129C;828636;

AA1 132T;Interacts with:117G

AA1 133V;Interacts with:117G

AA1 135E;Interacts with:120Y

AA1 136E;Interacts with:120Y

MUT1 E136R;PMID:11461956;

AA1 98R;Interacts with:100R

AA1 99Q;Interacts with:100R,101Y

AA2 100R;Interacts with:103D,102D,99Q,98R

AA2 101Y;Interacts with:99Q

AA2 115T;Interacts with:118Q,120H

AA2 116P;Interacts with:118Q

AA2 117G;Interacts with:132T,129R,115M,133V,119L,118Q

AA2 120Y;Interacts with:135E,136E

AA2 91R;Interacts with:107R

AA2 92E;Interacts with:108Y,107R

AA2 95D;Interacts with:107R

AA2 96E;Interacts with:107R,104F,106R

AA2 97F;Interacts with:103D

AA2 99L;Interacts with:103D

//

MESH1 mesh:D002277

NAME1 Carcinoma

CLUSTER1 clust2

MESH2 mesh:D006528

NAME2 Carcinoma, Hepatocellular

CLUSTER2 clust2

AC1 P10415

PFAM1 PF02180

PDB1 4AQ3:A

AC2 Q07817

PFAM2 PF00452

PDB2 4AQ3:A

INT semi-edgetic

AA1 11N;Interacts with:188W,171A,175N,174L,179E

AA1 12R;Interacts with:171A,175N

MUT1 R12Q;PMID:9463381;

AA1 14I;Interacts with:90L,188W,144F

AA1 15V;Interacts with:170M,174L,167A,144F,171A

AA1 16M;Interacts with:167A

AA1 17K;Interacts with:91R

AA1 18Y;Interacts with:145S,144F,90L,91R,148G,170M,95D

AA1 19I;Interacts with:163V,167A,170M

AA1 21Y;Interacts with:91R

AA1 22K;Interacts with:95D,152V,145S,98E,91R

AA1 23L;Interacts with:155V,163V,152V,151C

AA1 25Q;Interacts with:95D,183Q

AA1 26R;Interacts with:179E,184E,180P,183Q

AA1 27G;Interacts with:183Q

AA1 28Y;Interacts with:155V

AA1 30W;Interacts with:167A,163V,164S,160Q

AA1 92V;Interacts with:90L

AA1 9Y;Interacts with:188W

AA2 144F;Interacts with:18Y,15V,14I

AA2 145S;Interacts with:18Y,22K

AA2 148G;Interacts with:18Y

MUT2 G148E;827593;

AA2 151C;Interacts with:23L

AA2 152V;Interacts with:23L,22K

AA2 155V;Interacts with:23L,28Y

AA2 160Q;Interacts with:30W

AA2 163V;Interacts with:19I,23L,30W

AA2 164S;Interacts with:30W

AA2 167A;Interacts with:15V,19I,30W,16M

MUT2 A167V;1KG:1359392;

AA2 170M;Interacts with:15V,19I,18Y

AA2 171A;Interacts with:11N,15V,12R

AA2 174L;Interacts with:15V,11N

AA2 175N;Interacts with:11N,12R

AA2 179E;Interacts with:26R,11N

AA2 180P;Interacts with:26R

AA2 183Q;Interacts with:25Q,27G,26R

AA2 184E;Interacts with:26R

AA2 188W;Interacts with:11N,14I,9Y

AA2 90L;Interacts with:14I,18Y,92V

AA2 91R;Interacts with:17K,21Y,18Y,22K

AA2 95D;Interacts with:22K,25Q,18Y

AA2 98E;Interacts with:22K

//

MESH1 mesh:D002277

NAME1 Carcinoma

CLUSTER1 clust2

MESH2 mesh:D006528

NAME2 Carcinoma, Hepatocellular

CLUSTER2 clust2

AC1 Q07817

PFAM1 PF00452

PDB1 2P1L:A

AC2 Q14457

PFAM2 PF15285

PDB2 2P1L:A

INT semi-edgetic

AA1 100R;Interacts with:122L,119T,123F

AA1 101Y;Interacts with:116L,115R,119T,112L

AA1 105F;Interacts with:115R,118V

AA1 106S;Interacts with:115R

AA1 108L;Interacts with:116L,112L

MUT1 L108R;dbSNP:rs139457299;dbSNP:rs139457299;

AA1 111Q;Interacts with:115R,108T,111N,112L

AA1 112L;Interacts with:108T,109M,112L

AA1 113H;Interacts with:3G,108T,109M

AA1 122S;Interacts with:109M

AA1 125Q;Interacts with:4S,106G

AA1 126V;Interacts with:109M,112L,113S,116L

AA1 129E;Interacts with:113S,117K,114R

AA1 130L;Interacts with:117K,116L,113S

AA1 133D;Interacts with:117K

AA1 136N;Interacts with:120G,124D,121D,125I

AA1 137W;Interacts with:124D,125I,126M

AA1 138G;Interacts with:124D,123F,121D,120G

MUT1 G138A;827592;

AA1 139R;Interacts with:121D,120G,117K,124D

AA1 141V;Interacts with:123F

AA1 142A;Interacts with:116L,120G

AA1 146F;Interacts with:112L,116L

AA1 185N;Interacts with:126M

AA1 93A;Interacts with:123F

AA1 96E;Interacts with:123F

AA1 97F;Interacts with:119T,123F,120G,116L

AA2 106G;Interacts with:125Q

AA2 108T;Interacts with:112L,111Q,113H

AA2 109M;Interacts with:126V,122S,112L,113H

AA2 111N;Interacts with:111Q

AA2 112L;Interacts with:146F,126V,108L,112L,101Y,111Q

AA2 113S;Interacts with:129E,126V,130L

MUT2 S113R;dbSNP:rs80236238;

AA2 114R;Interacts with:129E

AA2 115R;Interacts with:111Q,105F,101Y,106S

AA2 116L;Interacts with:101Y,130L,142A,146F,108L,126V,97F

AA2 117K;Interacts with:130L,139R,133D,129E

AA2 118V;Interacts with:105F

AA2 119T;Interacts with:97F,100R,101Y

AA2 120G;Interacts with:139R,136N,97F,142A,138G

AA2 121D;Interacts with:139R,138G,136N

AA2 122L;Interacts with:100R

AA2 123F;Interacts with:97F,93A,141V,138G,96E,100R

AA2 124D;Interacts with:138G,136N,137W,139R

AA2 125I;Interacts with:137W,136N

AA2 126M;Interacts with:185N,137W

AA2 3G;Interacts with:113H

AA2 4S;Interacts with:125Q

//

MESH1 mesh:D002277

NAME1 Carcinoma

CLUSTER1 clust2

MESH2 mesh:D006528

NAME2 Carcinoma, Hepatocellular

CLUSTER2 clust2

AC1 O43521

PFAM1 PF08945

PDB1 3FDL:B

AC2 Q07817

PFAM2 PF00452

PDB2 3FDL:B

INT semi-edgetic

AA1 143R;Interacts with:111Q

MUT1 R143H;dbSNP:rs141962978;dbSNP:rs141962978;dbSNP:rs141962978;dbSNP:rs141962978;dbSNP:rs141962978;

AA1 144P;Interacts with:112L,111Q

AA1 145E;Interacts with:126V,113H,125Q,122S,112L

AA1 146I;Interacts with:125Q

AA1 147W;Interacts with:107D,108L

AA1 148I;Interacts with:126V,108L,146F,111Q,112L,104A

AA1 149A;Interacts with:126V,129E,130L

AA1 150Q;Interacts with:129E

AA1 151E;Interacts with:108L

AA1 152L;Interacts with:142A,130L,97F,146F,105F,108L,126V,145S,104A

AA1 153R;Interacts with:130L,133D,139R,129E

MUT1 R153W;dbSNP:rs146318804;dbSNP:rs146318804;dbSNP:rs146318804;dbSNP:rs146318804;dbSNP:rs146318804;

AA1 155I;Interacts with:97F,101Y,105F,108L,104A

AA1 156G;Interacts with:136N,97F,142A,139R,138G,101Y

AA1 157D;Interacts with:138G,139R,136N

AA1 158E;Interacts with:100R,101Y

AA1 159F;Interacts with:97F,96E,93A,141V,138G,101Y,100R

AA1 160N;Interacts with:137W,136N,138G,139R

AA1 162Y;Interacts with:100R

AA1 163Y;Interacts with:100R

AA1 1M;Interacts with:113H,111Q,112L

AA2 100R;Interacts with:158E,159F,162Y,163Y

AA2 101Y;Interacts with:155I,159F,158E,156G

AA2 104A;Interacts with:155I,152L,148I

AA2 105F;Interacts with:155I,152L

AA2 107D;Interacts with:147W

AA2 108L;Interacts with:155I,147W,148I,152L,151E

MUT2 L108R;dbSNP:rs139457299;dbSNP:rs139457299;

AA2 111Q;Interacts with:143R,148I,1M,144P

AA2 112L;Interacts with:148I,1M,145E,144P

AA2 113H;Interacts with:1M,145E

AA2 122S;Interacts with:145E

AA2 125Q;Interacts with:145E,146I

AA2 126V;Interacts with:145E,149A,148I,152L

AA2 129E;Interacts with:149A,150Q,153R

AA2 130L;Interacts with:153R,152L,149A

AA2 133D;Interacts with:153R

AA2 136N;Interacts with:156G,160N,157D

AA2 137W;Interacts with:160N

AA2 138G;Interacts with:157D,160N,159F,156G

MUT2 G138A;827592;

AA2 139R;Interacts with:157D,156G,153R,160N

AA2 141V;Interacts with:159F

AA2 142A;Interacts with:156G,152L

AA2 145S;Interacts with:152L

AA2 146F;Interacts with:152L,148I

AA2 93A;Interacts with:159F

AA2 96E;Interacts with:159F

AA2 97F;Interacts with:155I,159F,156G,152L

//

MESH1 mesh:D002277

NAME1 Carcinoma

CLUSTER1 clust2

MESH2 mesh:D006528

NAME2 Carcinoma, Hepatocellular

CLUSTER2 clust2

AC1 P55957

PFAM1 PF06393

PDB1 4BD2:C

AC2 Q07812

PFAM2 PF00452

PDB2 4BD2:A

INT semi-edgetic

AA1 102P;Interacts with:65R

AA1 103P;Interacts with:65R,66I

AA1 104G;Interacts with:66I,69E

AA1 105L;Interacts with:70L,69E,66I

MUT1 L105P;dbSNP:rs143734092;dbSNP:rs143734092;

AA1 79Q;Interacts with:82A,83V,84D

AA1 80E;Interacts with:94R

AA1 82I;Interacts with:83V,82A,79M

AA1 83I;Interacts with:91V,94R,83V,95V

AA1 84R;Interacts with:98D

MUT1 R84W;dbSNP:rs148107209;dbSNP:rs148107209;

AA1 86I;Interacts with:83V,116F,79M,80I,95V

AA1 87A;Interacts with:98D,99M,95V

AA1 89H;Interacts with:76L,75E,79M,73N

AA1 90L;Interacts with:116F,76L,95V,112A,99M,115Y

AA1 91A;Interacts with:109R,99M

AA1 93V;Interacts with:70L,112A

AA1 94G;Interacts with:108G,106N,112A,109R

MUT1 G94E;PMID:11121101;

AA1 95D;Interacts with:109R,106N

AA1 97M;Interacts with:66I,111V,108G

AA1 98D;Interacts with:106N

AA2 106N;Interacts with:98D,94G,95D

AA2 108G;Interacts with:94G,97M

MUT2 G108V;828239;humsavar:VAR_013576, phenotype=A Burkitt lymphoma;

AA2 109R;Interacts with:91A,95D,94G

AA2 111V;Interacts with:97M

AA2 112A;Interacts with:93V,94G,90L

AA2 115Y;Interacts with:90L

AA2 116F;Interacts with:90L,86I

AA2 65R;Interacts with:103P,102P

MUT2 R65L;dbSNP:rs142278713;dbSNP:rs142278713;dbSNP:rs142278713;

AA2 66I;Interacts with:104G,97M,105L,103P

AA2 69E;Interacts with:105L,104G

AA2 70L;Interacts with:105L,93V

AA2 73N;Interacts with:89H

AA2 75E;Interacts with:89H

AA2 76L;Interacts with:89H,90L

AA2 79M;Interacts with:89H,86I,82I

AA2 80I;Interacts with:86I

AA2 82A;Interacts with:79Q,82I

AA2 83V;Interacts with:82I,86I,79Q,83I

AA2 84D;Interacts with:79Q

AA2 91V;Interacts with:83I

AA2 94R;Interacts with:83I,80E

AA2 95V;Interacts with:83I,90L,87A,86I

AA2 98D;Interacts with:87A,84R

AA2 99M;Interacts with:91A,90L,87A

//

MESH1 mesh:D002277

NAME1 Carcinoma

CLUSTER1 clust2

MESH2 mesh:D006528

NAME2 Carcinoma, Hepatocellular

CLUSTER2 clust2

AC1 P55957

PFAM1 PF06393

PDB1 2KBW:B

AC2 Q07820

PFAM2 PF00452

PDB2 2KBW:A

INT semi-edgetic

AA1 79Q;Interacts with:242D,236D,245S,235L

AA1 82I;Interacts with:234K,235L

AA1 83I;Interacts with:245S,249V,235L,248R

AA1 84R;Interacts with:252H

MUT1 R84W;dbSNP:rs148107209;dbSNP:rs148107209;

AA1 86I;Interacts with:235L,249V,234K,231M

AA1 87A;Interacts with:252H,253V,249V

AA1 88R;Interacts with:253V

AA1 89H;Interacts with:231M

AA1 90L;Interacts with:253V,231M,249V,266T,270F,267L

AA1 91A;Interacts with:253V,263R

AA1 93V;Interacts with:266T,231M,227A,224H

AA1 94G;Interacts with:263R,262G,266T,260N

MUT1 G94E;PMID:11121101;

AA1 95D;Interacts with:263R,266T,262G,260N

AA1 97M;Interacts with:266T,265V,216V,262G,220V

AA1 98D;Interacts with:261W,263R,262G,260N

AA2 216V;Interacts with:97M

AA2 220V;Interacts with:97M

AA2 224H;Interacts with:93V

AA2 227A;Interacts with:93V

MUT2 A227V;169059;dbSNP:rs11580946;humsavar:VAR_024022;rs11580946;

AA2 231M;Interacts with:93V,90L,89H,86I

MUT2 M231L;898693;dbSNP:rs140449444;humsavar:VAR_054157;

AA2 234K;Interacts with:82I,86I

MUT2 K234R;898704;

AA2 235L;Interacts with:86I,82I,83I,79Q

AA2 236D;Interacts with:79Q

AA2 242D;Interacts with:79Q

AA2 245S;Interacts with:83I,79Q

AA2 248R;Interacts with:83I

AA2 249V;Interacts with:86I,83I,90L,87A

AA2 252H;Interacts with:84R,87A

AA2 253V;Interacts with:88R,90L,91A,87A

AA2 260N;Interacts with:95D,98D,94G

AA2 261W;Interacts with:98D

AA2 262G;Interacts with:95D,97M,94G,98D

AA2 263R;Interacts with:95D,98D,91A,94G

AA2 265V;Interacts with:97M

AA2 266T;Interacts with:93V,97M,90L,95D,94G

AA2 267L;Interacts with:90L

AA2 270F;Interacts with:90L

//

MESH1 mesh:D002277

NAME1 Carcinoma

CLUSTER1 clust2

MESH2 mesh:D006528

NAME2 Carcinoma, Hepatocellular

CLUSTER2 clust2

AC1 O43521

PFAM1 PF08945

PDB1 2VM6:B

AC2 Q16548

PFAM2 PF00452

PDB2 2VM6:B

INT semi-edgetic

AA1 142M;Interacts with:55C,58N

MUT1 M142R;dbSNP:rs114585494;dbSNP:rs114585494;dbSNP:rs114585494;dbSNP:rs114585494;dbSNP:rs114585494;

AA1 144P;Interacts with:70L,74V

AA1 145E;Interacts with:70L,77K,73Q,74V

AA1 147W;Interacts with:54S,55C,52L,51N

AA1 148I;Interacts with:70L,74V,52L,95F,56L,59V

AA1 149A;Interacts with:77K,78E,74V

AA1 151E;Interacts with:51N,48V

AA1 152L;Interacts with:78E,48V,95F,91T,74V

AA1 153R;Interacts with:79F,88R,80E,78E,81D,91T,77K

MUT1 R153W;dbSNP:rs146318804;dbSNP:rs146318804;dbSNP:rs146318804;dbSNP:rs146318804;dbSNP:rs146318804;

AA1 154R;Interacts with:47E,51N

AA1 155I;Interacts with:48V,91T,44V,47E

AA1 156G;Interacts with:87G,91T,85N,88R

AA1 157D;Interacts with:88R,87G,85N

AA1 159F;Interacts with:40V,43S,44V,87G

AA1 160N;Interacts with:86W,87G,85N

AA1 163Y;Interacts with:40V

AA2 40V;Interacts with:163Y,159F

AA2 43S;Interacts with:159F

AA2 44V;Interacts with:155I,159F

AA2 47E;Interacts with:154R,155I

AA2 48V;Interacts with:155I,152L,151E

AA2 51N;Interacts with:147W,154R,151E

AA2 52L;Interacts with:148I,147W

AA2 54S;Interacts with:147W

AA2 55C;Interacts with:147W,142M

AA2 56L;Interacts with:148I

AA2 58N;Interacts with:142M

MUT2 N58S;dbSNP:rs141166047;dbSNP:rs141166047;

AA2 59V;Interacts with:148I

AA2 70L;Interacts with:145E,148I,144P

AA2 73Q;Interacts with:145E

AA2 74V;Interacts with:148I,144P,152L,149A,145E

AA2 77K;Interacts with:149A,145E,153R

AA2 78E;Interacts with:152L,153R,149A

AA2 79F;Interacts with:153R

AA2 80E;Interacts with:153R

AA2 81D;Interacts with:153R

AA2 85N;Interacts with:157D,156G,160N

AA2 86W;Interacts with:160N

AA2 87G;Interacts with:157D,156G,160N,159F

AA2 88R;Interacts with:157D,153R,156G

AA2 91T;Interacts with:152L,155I,156G,153R

AA2 95F;Interacts with:152L,148I

//

MESH1 mesh:D002277

NAME1 Carcinoma

CLUSTER1 clust2

MESH2 mesh:D007938

NAME2 Leukemia

CLUSTER2 clust2

AC1 O43521

PFAM1 PF08945

PDB1 2NL9:B

AC2 Q07820

PFAM2 PF00452

PDB2 2NL9:B

INT non-edgetic

AA1 143R;Interacts with:234K

MUT1 R143H;dbSNP:rs141962978;dbSNP:rs141962978;dbSNP:rs141962978;dbSNP:rs141962978;dbSNP:rs141962978;

AA1 144P;Interacts with:234K,235L

AA1 145E;Interacts with:248R,249V,245S,252H,235L

AA1 146I;Interacts with:252H

AA1 147W;Interacts with:230G,231M

AA1 148I;Interacts with:270F,235L,234K,249V,231M

AA1 149A;Interacts with:249V,252H,253V

AA1 151E;Interacts with:231M

AA1 152L;Interacts with:228F,270F,253V,249V,266T,267L,231M

AA1 153R;Interacts with:252H,253V,254F,255S,256D,263R

MUT1 R153W;dbSNP:rs146318804;dbSNP:rs146318804;dbSNP:rs146318804;dbSNP:rs146318804;dbSNP:rs146318804;

AA1 155I;Interacts with:266T,220V,228F,227A,224H,231M

AA1 156G;Interacts with:262G,260N,266T,263R

AA1 157D;Interacts with:260N,263R,262G

AA1 158E;Interacts with:224H

AA1 159F;Interacts with:265V,220V,262G,216V,219G,224H

AA1 160N;Interacts with:261W,262G,260N,263R

AA1 163Y;Interacts with:215R,216V

AA2 215R;Interacts with:163Y

AA2 216V;Interacts with:159F,163Y

AA2 219G;Interacts with:159F

AA2 220V;Interacts with:159F,155I

AA2 224H;Interacts with:158E,155I,159F

AA2 227A;Interacts with:155I

MUT2 A227V;169059;dbSNP:rs11580946;humsavar:VAR_024022;rs11580946;

AA2 228F;Interacts with:152L,155I

AA2 230G;Interacts with:147W

AA2 231M;Interacts with:147W,151E,148I,152L,155I

MUT2 M231L;898693;dbSNP:rs140449444;humsavar:VAR_054157;

AA2 234K;Interacts with:148I,144P,143R

MUT2 K234R;898704;

AA2 235L;Interacts with:148I,145E,144P

AA2 245S;Interacts with:145E

AA2 248R;Interacts with:145E

AA2 249V;Interacts with:149A,152L,145E,148I

AA2 252H;Interacts with:153R,149A,146I,145E

AA2 253V;Interacts with:152L,153R,149A

AA2 254F;Interacts with:153R

AA2 255S;Interacts with:153R

AA2 256D;Interacts with:153R

AA2 260N;Interacts with:156G,157D,160N

AA2 261W;Interacts with:160N

AA2 262G;Interacts with:159F,156G,160N,157D

AA2 263R;Interacts with:160N,156G,153R,157D

AA2 265V;Interacts with:159F

AA2 266T;Interacts with:155I,152L,156G

AA2 267L;Interacts with:152L

AA2 270F;Interacts with:148I,152L

//

MESH1 mesh:D002277

NAME1 Carcinoma

CLUSTER1 clust2

MESH2 mesh:D007938

NAME2 Leukemia

CLUSTER2 clust2

AC1 O43521

PFAM1 PF08945

PDB1 2K7W:B

AC2 Q07812

PFAM2 PF00452

PDB2 2K7W:B

INT non-edgetic

AA1 145E;Interacts with:146E,142D,89R

AA1 146I;Interacts with:142D

AA1 148I;Interacts with:142D

AA1 149A;Interacts with:138G,142D,139W

AA1 152L;Interacts with:142D,141L,145R,138G

AA1 153R;Interacts with:138G,131E,135T,134R

MUT1 R153W;dbSNP:rs146318804;dbSNP:rs146318804;dbSNP:rs146318804;dbSNP:rs146318804;dbSNP:rs146318804;

AA1 156G;Interacts with:141L,137M,134R

AA1 157D;Interacts with:134R

AA1 160N;Interacts with:134R

AA2 131E;Interacts with:153R

AA2 134R;Interacts with:157D,153R,156G,160N

AA2 135T;Interacts with:153R

AA2 137M;Interacts with:156G

AA2 138G;Interacts with:149A,153R,152L

AA2 139W;Interacts with:149A

MUT2 W139C;COSMIC:267934;

AA2 141L;Interacts with:156G,152L

AA2 142D;Interacts with:145E,149A,152L,148I,146I

AA2 145R;Interacts with:152L

AA2 146E;Interacts with:145E

AA2 89R;Interacts with:145E

MUT2 R89Q;1KG:1191554;1KG:1224249;1KG:1224241;

//

MESH1 mesh:D002277

NAME1 Carcinoma

CLUSTER1 clust2

MESH2 mesh:D007938

NAME2 Leukemia

CLUSTER2 clust2

AC1 P10415

PFAM1 PF00452

PDB1 4AQ3:A

AC2 Q07817

PFAM2 PF00452

PDB2 4AQ3:C

INT non-edgetic

AA1 102D;Interacts with:100R

AA1 103D;Interacts with:100R,99L,97F

AA1 104F;Interacts with:96E

AA1 106R;Interacts with:96E

AA1 107R;Interacts with:95D,96E,92E,91R

AA1 108Y;Interacts with:92E

AA1 115M;Interacts with:117G

AA1 118Q;Interacts with:116P,115T,117G

AA1 119L;Interacts with:117G

AA1 120H;Interacts with:115T

AA1 129R;Interacts with:117G

MUT1 R129C;828636;

AA1 132T;Interacts with:117G

AA1 133V;Interacts with:117G

AA1 135E;Interacts with:120Y

AA1 136E;Interacts with:120Y

MUT1 E136R;PMID:11461956;

AA1 98R;Interacts with:100R

AA1 99Q;Interacts with:100R,101Y

AA2 100R;Interacts with:103D,102D,99Q,98R

AA2 101Y;Interacts with:99Q

AA2 115T;Interacts with:118Q,120H

AA2 116P;Interacts with:118Q

AA2 117G;Interacts with:132T,129R,115M,133V,119L,118Q

AA2 120Y;Interacts with:135E,136E

AA2 91R;Interacts with:107R

AA2 92E;Interacts with:108Y,107R

AA2 95D;Interacts with:107R

AA2 96E;Interacts with:107R,104F,106R

AA2 97F;Interacts with:103D

AA2 99L;Interacts with:103D

//

MESH1 mesh:D002277

NAME1 Carcinoma

CLUSTER1 clust2

MESH2 mesh:D007938

NAME2 Leukemia

CLUSTER2 clust2

AC1 P10415

PFAM1 PF02180

PDB1 4AQ3:A

AC2 Q07817

PFAM2 PF00452

PDB2 4AQ3:A

INT non-edgetic

AA1 11N;Interacts with:188W,171A,175N,174L,179E

AA1 12R;Interacts with:171A,175N

MUT1 R12Q;PMID:9463381;

AA1 14I;Interacts with:90L,188W,144F

AA1 15V;Interacts with:170M,174L,167A,144F,171A

AA1 16M;Interacts with:167A

AA1 17K;Interacts with:91R

AA1 18Y;Interacts with:145S,144F,90L,91R,148G,170M,95D

AA1 19I;Interacts with:163V,167A,170M

AA1 21Y;Interacts with:91R

AA1 22K;Interacts with:95D,152V,145S,98E,91R

AA1 23L;Interacts with:155V,163V,152V,151C

AA1 25Q;Interacts with:95D,183Q

AA1 26R;Interacts with:179E,184E,180P,183Q

AA1 27G;Interacts with:183Q

AA1 28Y;Interacts with:155V

AA1 30W;Interacts with:167A,163V,164S,160Q

AA1 92V;Interacts with:90L

AA1 9Y;Interacts with:188W

AA2 144F;Interacts with:18Y,15V,14I

AA2 145S;Interacts with:18Y,22K

AA2 148G;Interacts with:18Y

MUT2 G148E;827593;

AA2 151C;Interacts with:23L

AA2 152V;Interacts with:23L,22K

AA2 155V;Interacts with:23L,28Y

AA2 160Q;Interacts with:30W

AA2 163V;Interacts with:19I,23L,30W

AA2 164S;Interacts with:30W

AA2 167A;Interacts with:15V,19I,30W,16M

MUT2 A167V;1KG:1359392;

AA2 170M;Interacts with:15V,19I,18Y

AA2 171A;Interacts with:11N,15V,12R

AA2 174L;Interacts with:15V,11N

AA2 175N;Interacts with:11N,12R

AA2 179E;Interacts with:26R,11N

AA2 180P;Interacts with:26R

AA2 183Q;Interacts with:25Q,27G,26R

AA2 184E;Interacts with:26R

AA2 188W;Interacts with:11N,14I,9Y

AA2 90L;Interacts with:14I,18Y,92V

AA2 91R;Interacts with:17K,21Y,18Y,22K

AA2 95D;Interacts with:22K,25Q,18Y

AA2 98E;Interacts with:22K

//

MESH1 mesh:D002277

NAME1 Carcinoma

CLUSTER1 clust2

MESH2 mesh:D007938

NAME2 Leukemia

CLUSTER2 clust2

AC1 O43521

PFAM1 PF08945

PDB1 3FDL:B

AC2 Q07817

PFAM2 PF00452

PDB2 3FDL:B

INT non-edgetic

AA1 143R;Interacts with:111Q

MUT1 R143H;dbSNP:rs141962978;dbSNP:rs141962978;dbSNP:rs141962978;dbSNP:rs141962978;dbSNP:rs141962978;

AA1 144P;Interacts with:112L,111Q

AA1 145E;Interacts with:126V,113H,125Q,122S,112L

AA1 146I;Interacts with:125Q

AA1 147W;Interacts with:107D,108L

AA1 148I;Interacts with:126V,108L,146F,111Q,112L,104A

AA1 149A;Interacts with:126V,129E,130L

AA1 150Q;Interacts with:129E

AA1 151E;Interacts with:108L

AA1 152L;Interacts with:142A,130L,97F,146F,105F,108L,126V,145S,104A

AA1 153R;Interacts with:130L,133D,139R,129E

MUT1 R153W;dbSNP:rs146318804;dbSNP:rs146318804;dbSNP:rs146318804;dbSNP:rs146318804;dbSNP:rs146318804;

AA1 155I;Interacts with:97F,101Y,105F,108L,104A

AA1 156G;Interacts with:136N,97F,142A,139R,138G,101Y

AA1 157D;Interacts with:138G,139R,136N

AA1 158E;Interacts with:100R,101Y

AA1 159F;Interacts with:97F,96E,93A,141V,138G,101Y,100R

AA1 160N;Interacts with:137W,136N,138G,139R

AA1 162Y;Interacts with:100R

AA1 163Y;Interacts with:100R

AA1 1M;Interacts with:113H,111Q,112L

AA2 100R;Interacts with:158E,159F,162Y,163Y

AA2 101Y;Interacts with:155I,159F,158E,156G

AA2 104A;Interacts with:155I,152L,148I

AA2 105F;Interacts with:155I,152L

AA2 107D;Interacts with:147W

AA2 108L;Interacts with:155I,147W,148I,152L,151E

MUT2 L108R;dbSNP:rs139457299;dbSNP:rs139457299;

AA2 111Q;Interacts with:143R,148I,1M,144P

AA2 112L;Interacts with:148I,1M,145E,144P

AA2 113H;Interacts with:1M,145E

AA2 122S;Interacts with:145E

AA2 125Q;Interacts with:145E,146I

AA2 126V;Interacts with:145E,149A,148I,152L

AA2 129E;Interacts with:149A,150Q,153R

AA2 130L;Interacts with:153R,152L,149A

AA2 133D;Interacts with:153R

AA2 136N;Interacts with:156G,160N,157D

AA2 137W;Interacts with:160N

AA2 138G;Interacts with:157D,160N,159F,156G

MUT2 G138A;827592;

AA2 139R;Interacts with:157D,156G,153R,160N

AA2 141V;Interacts with:159F

AA2 142A;Interacts with:156G,152L

AA2 145S;Interacts with:152L

AA2 146F;Interacts with:152L,148I

AA2 93A;Interacts with:159F

AA2 96E;Interacts with:159F

AA2 97F;Interacts with:155I,159F,156G,152L

//

MESH1 mesh:D002277

NAME1 Carcinoma

CLUSTER1 clust2

MESH2 mesh:D007938

NAME2 Leukemia

CLUSTER2 clust2

AC1 Q13794

PFAM1 PF15150

PDB1 3MQP:B

AC2 Q16548

PFAM2 PF00452

PDB2 3MQP:B

INT non-edgetic

AA1 21L;Interacts with:55C,59V,58N

AA1 22E;Interacts with:74V,73Q

MUT1 E22K;dbSNP:rs1126436;

AA1 24E;Interacts with:52L

AA1 25C;Interacts with:74V,52L

AA1 26A;Interacts with:77K,78E,74V

AA1 28Q;Interacts with:51N

AA1 29L;Interacts with:91T,48V,74V,78E,95F

MUT1 L29A;823237;

MUT1 L29E;823238;

AA1 30R;Interacts with:78E,81D,77K,80E,88R,91T

MUT1 R30delR;COSMIC:166155;

AA1 32F;Interacts with:47E,91T,48V

MUT1 F32I;823240;

MUT1 F32E;823239;

AA1 33G;Interacts with:91T,88R,85N,87G,78E

AA1 34D;Interacts with:87G,85N,88R

AA1 36L;Interacts with:87G,44V,40V,90V

MUT1 L36E;823242;

AA1 37N;Interacts with:86W,87G,88R,85N

AA2 40V;Interacts with:36L

AA2 44V;Interacts with:36L

AA2 47E;Interacts with:32F

AA2 48V;Interacts with:32F,29L

AA2 51N;Interacts with:28Q

AA2 52L;Interacts with:25C,24E

AA2 55C;Interacts with:21L

AA2 58N;Interacts with:21L

MUT2 N58S;dbSNP:rs141166047;dbSNP:rs141166047;

AA2 59V;Interacts with:21L

AA2 73Q;Interacts with:22E

AA2 74V;Interacts with:25C,29L,26A,22E

AA2 77K;Interacts with:26A,30R

AA2 78E;Interacts with:26A,30R,29L,33G

AA2 80E;Interacts with:30R

AA2 81D;Interacts with:30R

AA2 85N;Interacts with:34D,33G,37N

AA2 86W;Interacts with:37N

AA2 87G;Interacts with:36L,34D,37N,33G

AA2 88R;Interacts with:33G,37N,30R,34D

AA2 90V;Interacts with:36L

AA2 91T;Interacts with:29L,32F,33G,30R

AA2 95F;Interacts with:29L

//

MESH1 mesh:D002277

NAME1 Carcinoma

CLUSTER1 clust2

MESH2 mesh:D007938

NAME2 Leukemia

CLUSTER2 clust2

AC1 P55957

PFAM1 PF06393

PDB1 4BD2:C

AC2 Q07812

PFAM2 PF00452

PDB2 4BD2:A

INT non-edgetic

AA1 102P;Interacts with:65R

AA1 103P;Interacts with:65R,66I

AA1 104G;Interacts with:66I,69E

AA1 105L;Interacts with:70L,69E,66I

MUT1 L105P;dbSNP:rs143734092;dbSNP:rs143734092;

AA1 79Q;Interacts with:82A,83V,84D

AA1 80E;Interacts with:94R

AA1 82I;Interacts with:83V,82A,79M

AA1 83I;Interacts with:91V,94R,83V,95V

AA1 84R;Interacts with:98D

MUT1 R84W;dbSNP:rs148107209;dbSNP:rs148107209;

AA1 86I;Interacts with:83V,116F,79M,80I,95V

AA1 87A;Interacts with:98D,99M,95V

AA1 89H;Interacts with:76L,75E,79M,73N

AA1 90L;Interacts with:116F,76L,95V,112A,99M,115Y

AA1 91A;Interacts with:109R,99M

AA1 93V;Interacts with:70L,112A

AA1 94G;Interacts with:108G,106N,112A,109R

MUT1 G94E;PMID:11121101;

AA1 95D;Interacts with:109R,106N

AA1 97M;Interacts with:66I,111V,108G

AA1 98D;Interacts with:106N

AA2 106N;Interacts with:98D,94G,95D

AA2 108G;Interacts with:94G,97M

MUT2 G108V;828239;humsavar:VAR_013576, phenotype=A Burkitt lymphoma;

AA2 109R;Interacts with:91A,95D,94G

AA2 111V;Interacts with:97M

AA2 112A;Interacts with:93V,94G,90L

AA2 115Y;Interacts with:90L

AA2 116F;Interacts with:90L,86I

AA2 65R;Interacts with:103P,102P

MUT2 R65L;dbSNP:rs142278713;dbSNP:rs142278713;dbSNP:rs142278713;

AA2 66I;Interacts with:104G,97M,105L,103P

AA2 69E;Interacts with:105L,104G

AA2 70L;Interacts with:105L,93V

AA2 73N;Interacts with:89H

AA2 75E;Interacts with:89H

AA2 76L;Interacts with:89H,90L

AA2 79M;Interacts with:89H,86I,82I

AA2 80I;Interacts with:86I

AA2 82A;Interacts with:79Q,82I

AA2 83V;Interacts with:82I,86I,79Q,83I

AA2 84D;Interacts with:79Q

AA2 91V;Interacts with:83I

AA2 94R;Interacts with:83I,80E

AA2 95V;Interacts with:83I,90L,87A,86I

AA2 98D;Interacts with:87A,84R

AA2 99M;Interacts with:91A,90L,87A

//

MESH1 mesh:D002277

NAME1 Carcinoma

CLUSTER1 clust2

MESH2 mesh:D007938

NAME2 Leukemia

CLUSTER2 clust2

AC1 P55957

PFAM1 PF06393

PDB1 2KBW:B

AC2 Q07820

PFAM2 PF00452

PDB2 2KBW:A

INT non-edgetic

AA1 79Q;Interacts with:242D,236D,245S,235L

AA1 82I;Interacts with:234K,235L

AA1 83I;Interacts with:245S,249V,235L,248R

AA1 84R;Interacts with:252H

MUT1 R84W;dbSNP:rs148107209;dbSNP:rs148107209;

AA1 86I;Interacts with:235L,249V,234K,231M

AA1 87A;Interacts with:252H,253V,249V

AA1 88R;Interacts with:253V

AA1 89H;Interacts with:231M

AA1 90L;Interacts with:253V,231M,249V,266T,270F,267L

AA1 91A;Interacts with:253V,263R

AA1 93V;Interacts with:266T,231M,227A,224H

AA1 94G;Interacts with:263R,262G,266T,260N

MUT1 G94E;PMID:11121101;

AA1 95D;Interacts with:263R,266T,262G,260N

AA1 97M;Interacts with:266T,265V,216V,262G,220V

AA1 98D;Interacts with:261W,263R,262G,260N

AA2 216V;Interacts with:97M

AA2 220V;Interacts with:97M

AA2 224H;Interacts with:93V

AA2 227A;Interacts with:93V

MUT2 A227V;169059;dbSNP:rs11580946;humsavar:VAR_024022;rs11580946;

AA2 231M;Interacts with:93V,90L,89H,86I

MUT2 M231L;898693;dbSNP:rs140449444;humsavar:VAR_054157;

AA2 234K;Interacts with:82I,86I

MUT2 K234R;898704;

AA2 235L;Interacts with:86I,82I,83I,79Q

AA2 236D;Interacts with:79Q

AA2 242D;Interacts with:79Q

AA2 245S;Interacts with:83I,79Q

AA2 248R;Interacts with:83I

AA2 249V;Interacts with:86I,83I,90L,87A

AA2 252H;Interacts with:84R,87A

AA2 253V;Interacts with:88R,90L,91A,87A

AA2 260N;Interacts with:95D,98D,94G

AA2 261W;Interacts with:98D

AA2 262G;Interacts with:95D,97M,94G,98D

AA2 263R;Interacts with:95D,98D,91A,94G

AA2 265V;Interacts with:97M

AA2 266T;Interacts with:93V,97M,90L,95D,94G

AA2 267L;Interacts with:90L

AA2 270F;Interacts with:90L

//

MESH1 mesh:D002277

NAME1 Carcinoma

CLUSTER1 clust2

MESH2 mesh:D007938

NAME2 Leukemia

CLUSTER2 clust2

AC1 P55957

PFAM1 PF06393

PDB1 1ZY3:B

AC2 Q92843

PFAM2 PF00452

PDB2 1ZY3:A

INT non-edgetic

AA1 100S;Interacts with:56R

AA1 82I;Interacts with:85E

AA1 83I;Interacts with:71T,82V,85E

AA1 84R;Interacts with:88Q,86L,85E

MUT1 R84W;dbSNP:rs148107209;dbSNP:rs148107209;

AA1 86I;Interacts with:82V,71T,68L

AA1 87A;Interacts with:95R,85E,82V,86L

AA1 88R;Interacts with:86L

AA1 89H;Interacts with:68L,67Q

AA1 90L;Interacts with:67Q,64L,98A,102F,83S,68L,82V,95R

AA1 91A;Interacts with:95R,86L

AA1 93V;Interacts with:57F,64L

AA1 94G;Interacts with:57F

MUT1 G94E;PMID:11121101;

AA1 95D;Interacts with:92N,93W,95R,94G

AA1 97M;Interacts with:57F,56R

AA1 98D;Interacts with:53F,56R

AA1 99R;Interacts with:52E

AA2 102F;Interacts with:90L

AA2 52E;Interacts with:99R

AA2 53F;Interacts with:98D

AA2 56R;Interacts with:98D,100S,97M

AA2 57F;Interacts with:97M,93V,94G

AA2 64L;Interacts with:90L,93V

AA2 67Q;Interacts with:90L,89H

AA2 68L;Interacts with:89H,90L,86I

AA2 71T;Interacts with:83I,86I

AA2 82V;Interacts with:86I,83I,90L,87A

AA2 83S;Interacts with:90L

AA2 85E;Interacts with:82I,87A,83I,84R

AA2 86L;Interacts with:84R,88R,91A,87A

AA2 88Q;Interacts with:84R

AA2 92N;Interacts with:95D

AA2 93W;Interacts with:95D

AA2 94G;Interacts with:95D

AA2 95R;Interacts with:91A,87A,95D,90L

AA2 98A;Interacts with:90L

//

MESH1 mesh:D002277

NAME1 Carcinoma

CLUSTER1 clust2

MESH2 mesh:D007938

NAME2 Leukemia

CLUSTER2 clust2

AC1 O43521

PFAM1 PF08945

PDB1 2VM6:B

AC2 Q16548

PFAM2 PF00452

PDB2 2VM6:B

INT non-edgetic

AA1 142M;Interacts with:55C,58N

MUT1 M142R;dbSNP:rs114585494;dbSNP:rs114585494;dbSNP:rs114585494;dbSNP:rs114585494;dbSNP:rs114585494;

AA1 144P;Interacts with:70L,74V

AA1 145E;Interacts with:70L,77K,73Q,74V

AA1 147W;Interacts with:54S,55C,52L,51N

AA1 148I;Interacts with:70L,74V,52L,95F,56L,59V

AA1 149A;Interacts with:77K,78E,74V

AA1 151E;Interacts with:51N,48V

AA1 152L;Interacts with:78E,48V,95F,91T,74V

AA1 153R;Interacts with:79F,88R,80E,78E,81D,91T,77K

MUT1 R153W;dbSNP:rs146318804;dbSNP:rs146318804;dbSNP:rs146318804;dbSNP:rs146318804;dbSNP:rs146318804;

AA1 154R;Interacts with:47E,51N

AA1 155I;Interacts with:48V,91T,44V,47E

AA1 156G;Interacts with:87G,91T,85N,88R

AA1 157D;Interacts with:88R,87G,85N

AA1 159F;Interacts with:40V,43S,44V,87G

AA1 160N;Interacts with:86W,87G,85N

AA1 163Y;Interacts with:40V

AA2 40V;Interacts with:163Y,159F

AA2 43S;Interacts with:159F

AA2 44V;Interacts with:155I,159F

AA2 47E;Interacts with:154R,155I

AA2 48V;Interacts with:155I,152L,151E

AA2 51N;Interacts with:147W,154R,151E

AA2 52L;Interacts with:148I,147W

AA2 54S;Interacts with:147W

AA2 55C;Interacts with:147W,142M

AA2 56L;Interacts with:148I

AA2 58N;Interacts with:142M

MUT2 N58S;dbSNP:rs141166047;dbSNP:rs141166047;

AA2 59V;Interacts with:148I

AA2 70L;Interacts with:145E,148I,144P

AA2 73Q;Interacts with:145E

AA2 74V;Interacts with:148I,144P,152L,149A,145E

AA2 77K;Interacts with:149A,145E,153R

AA2 78E;Interacts with:152L,153R,149A

AA2 79F;Interacts with:153R

AA2 80E;Interacts with:153R

AA2 81D;Interacts with:153R

AA2 85N;Interacts with:157D,156G,160N

AA2 86W;Interacts with:160N

AA2 87G;Interacts with:157D,156G,160N,159F

AA2 88R;Interacts with:157D,153R,156G

AA2 91T;Interacts with:152L,155I,156G,153R

AA2 95F;Interacts with:152L,148I

//

MESH1 mesh:D002277

NAME1 Carcinoma

CLUSTER1 clust2

MESH2 mesh:D007938

NAME2 Leukemia

CLUSTER2 clust2

AC1 Q07817

PFAM1 PF00452

PDB1 2P1L:A

AC2 Q14457

PFAM2 PF15285

PDB2 2P1L:A

INT semi-edgetic

AA1 100R;Interacts with:122L,119T,123F

AA1 101Y;Interacts with:116L,115R,119T,112L

AA1 105F;Interacts with:115R,118V

AA1 106S;Interacts with:115R

AA1 108L;Interacts with:116L,112L

MUT1 L108R;dbSNP:rs139457299;dbSNP:rs139457299;

AA1 111Q;Interacts with:115R,108T,111N,112L

AA1 112L;Interacts with:108T,109M,112L

AA1 113H;Interacts with:3G,108T,109M

AA1 122S;Interacts with:109M

AA1 125Q;Interacts with:4S,106G

AA1 126V;Interacts with:109M,112L,113S,116L

AA1 129E;Interacts with:113S,117K,114R

AA1 130L;Interacts with:117K,116L,113S

AA1 133D;Interacts with:117K

AA1 136N;Interacts with:120G,124D,121D,125I

AA1 137W;Interacts with:124D,125I,126M

AA1 138G;Interacts with:124D,123F,121D,120G

MUT1 G138A;827592;

AA1 139R;Interacts with:121D,120G,117K,124D

AA1 141V;Interacts with:123F

AA1 142A;Interacts with:116L,120G

AA1 146F;Interacts with:112L,116L

AA1 185N;Interacts with:126M

AA1 93A;Interacts with:123F

AA1 96E;Interacts with:123F

AA1 97F;Interacts with:119T,123F,120G,116L

AA2 106G;Interacts with:125Q

AA2 108T;Interacts with:112L,111Q,113H

AA2 109M;Interacts with:126V,122S,112L,113H

AA2 111N;Interacts with:111Q

AA2 112L;Interacts with:146F,126V,108L,112L,101Y,111Q

AA2 113S;Interacts with:129E,126V,130L

MUT2 S113R;dbSNP:rs80236238;

AA2 114R;Interacts with:129E

AA2 115R;Interacts with:111Q,105F,101Y,106S

AA2 116L;Interacts with:101Y,130L,142A,146F,108L,126V,97F

AA2 117K;Interacts with:130L,139R,133D,129E

AA2 118V;Interacts with:105F

AA2 119T;Interacts with:97F,100R,101Y

AA2 120G;Interacts with:139R,136N,97F,142A,138G

AA2 121D;Interacts with:139R,138G,136N

AA2 122L;Interacts with:100R

AA2 123F;Interacts with:97F,93A,141V,138G,96E,100R

AA2 124D;Interacts with:138G,136N,137W,139R

AA2 125I;Interacts with:137W,136N

AA2 126M;Interacts with:185N,137W

AA2 3G;Interacts with:113H

AA2 4S;Interacts with:125Q

//

MESH1 mesh:D002277

NAME1 Carcinoma

CLUSTER1 clust2

MESH2 mesh:D007938

NAME2 Leukemia

CLUSTER2 clust2

AC1 O14727

PFAM1 PF00619

PDB1 3YGS:C

AC2 P55211

PFAM2 PF00619

PDB2 3YGS:P

INT semi-edgetic

AA1 23S;Interacts with:56R

AA1 24Y;Interacts with:52R

MUT1 Y24C;dbSNP:rs150457288;dbSNP:rs150457288;dbSNP:rs150457288;dbSNP:rs150457288;dbSNP:rs150457288;

AA1 27D;Interacts with:52R,56R,13R,14L

AA1 28H;Interacts with:14L

AA1 30I;Interacts with:10R,11R

AA1 31S;Interacts with:12C,13R,10R,15R,14L,11R

AA1 32D;Interacts with:11R

AA1 37I;Interacts with:60I

AA1 40E;Interacts with:13R,56R

AA1 78E;Interacts with:52R

AA2 10R;Interacts with:31S,30I

AA2 11R;Interacts with:32D,30I,31S

AA2 12C;Interacts with:31S

AA2 13R;Interacts with:40E,31S,27D

MUT2 R13A;PMID:10376594;

AA2 14L;Interacts with:31S,27D,28H

AA2 15R;Interacts with:31S

AA2 52R;Interacts with:27D,24Y,78E

MUT2 R52A;PMID:10376594;

AA2 56R;Interacts with:27D,40E,23S

MUT2 R56A;PMID:10376594;

AA2 60I;Interacts with:37I

MUT2 I60L;1KG:1285113;

//

MESH1 mesh:D002277

NAME1 Carcinoma

CLUSTER1 clust2

MESH2 mesh:D007938

NAME2 Leukemia

CLUSTER2 clust2

AC1 O95150

PFAM1 PF00229

PDB1 3K51:A

AC2 O95407

PFAM2 PF00020

PDB2 3K51:B

INT semi-edgetic

AA1 186D;Interacts with:84Y

AA1 187S;Interacts with:83N,84Y,85L

AA1 188Y;Interacts with:81F,85L,83N,89R,79T,80Q

AA1 189P;Interacts with:82W

AA1 190E;Interacts with:89R

AA1 191P;Interacts with:89R

AA1 192T;Interacts with:89R

AA1 221Q;Interacts with:86E

AA2 79T;Interacts with:188Y

AA2 80Q;Interacts with:188Y

AA2 81F;Interacts with:188Y

AA2 82W;Interacts with:189P

AA2 83N;Interacts with:187S,188Y

AA2 84Y;Interacts with:187S,186D

AA2 85L;Interacts with:188Y,187S

AA2 86E;Interacts with:221Q

AA2 89R;Interacts with:190E,192T,188Y,191P

//

MESH1 mesh:D002277

NAME1 Carcinoma

CLUSTER1 clust2

MESH2 mesh:D007938

NAME2 Leukemia

CLUSTER2 clust2

AC1 P23510

PFAM1 PF00229

PDB1 2HEV:F

AC2 P43489

PFAM2 PF00020

PDB2 2HEV:R

INT semi-edgetic

AA1 144T;Interacts with:55R

AA1 145Y;Interacts with:37P,36Y,55R

AA1 146K;Interacts with:34D,33G,45E,35T

AA1 180F;Interacts with:38S,37P,36Y

AA1 182V;Interacts with:37P

AA1 98D;Interacts with:55R

AA2 33G;Interacts with:146K

AA2 34D;Interacts with:146K

AA2 35T;Interacts with:146K

AA2 36Y;Interacts with:180F,145Y

AA2 37P;Interacts with:145Y,180F,182V

AA2 38S;Interacts with:180F

AA2 45E;Interacts with:146K

AA2 55R;Interacts with:98D,144T,145Y

//

MESH1 mesh:D002277

NAME1 Carcinoma

CLUSTER1 clust2

MESH2 mesh:D007938

NAME2 Leukemia

CLUSTER2 clust2

AC1 Q07817

PFAM1 PF00452

PDB1 1G5J:A

AC2 Q92934

PFAM2 PF10514

PDB2 1G5J:B

INT semi-edgetic

AA1 100R;Interacts with:121F,120E

AA1 101Y;Interacts with:117M

AA1 104A;Interacts with:110Y,114L

AA1 105F;Interacts with:110Y

AA1 108L;Interacts with:110Y

MUT1 L108R;dbSNP:rs139457299;dbSNP:rs139457299;

AA1 111Q;Interacts with:105W,106A

AA1 112L;Interacts with:107A,106A,104L

AA1 113H;Interacts with:103N

AA1 121Q;Interacts with:104L

AA1 122S;Interacts with:107A,104L

AA1 125Q;Interacts with:107A,108Q

AA1 126V;Interacts with:107A,111G,110Y

AA1 129E;Interacts with:112R,111G,115R

AA1 130L;Interacts with:111G,115R,114L

AA1 133D;Interacts with:115R

AA1 138G;Interacts with:121F,122V

MUT1 G138A;827592;

AA1 139R;Interacts with:115R,119D,118S

AA1 141V;Interacts with:121F

AA1 142A;Interacts with:114L,121F

AA1 146F;Interacts with:111G,110Y,114L

AA1 93A;Interacts with:121F

AA1 96E;Interacts with:121F

AA1 97F;Interacts with:117M,121F,118S,114L

AA2 103N;Interacts with:113H

AA2 104L;Interacts with:122S,112L,121Q

AA2 105W;Interacts with:111Q

AA2 106A;Interacts with:112L,111Q

AA2 107A;Interacts with:112L,122S,126V,125Q

MUT2 A107S;119982;humsavar:VAR_015380;rs3729933;

AA2 108Q;Interacts with:125Q

AA2 110Y;Interacts with:104A,146F,108L,105F,126V

AA2 111G;Interacts with:130L,146F,129E,126V

AA2 112R;Interacts with:129E

AA2 114L;Interacts with:104A,142A,130L,146F,97F

AA2 115R;Interacts with:133D,139R,129E,130L

AA2 117M;Interacts with:97F,101Y

AA2 118S;Interacts with:97F,139R

AA2 119D;Interacts with:139R

AA2 120E;Interacts with:100R

AA2 121F;Interacts with:100R,138G,141V,97F,96E,93A,142A

AA2 122V;Interacts with:138G

//

MESH1 mesh:D002277

NAME1 Carcinoma

CLUSTER1 clust2

MESH2 mesh:D008175

NAME2 Lung Neoplasms

CLUSTER2 clust2

AC1 P10415

PFAM1 PF00452

PDB1 4AQ3:A

AC2 Q07817

PFAM2 PF00452

PDB2 4AQ3:C

INT non-edgetic

AA1 102D;Interacts with:100R

AA1 103D;Interacts with:100R,99L,97F

AA1 104F;Interacts with:96E

AA1 106R;Interacts with:96E

AA1 107R;Interacts with:95D,96E,92E,91R

AA1 108Y;Interacts with:92E

AA1 115M;Interacts with:117G

AA1 118Q;Interacts with:116P,115T,117G

AA1 119L;Interacts with:117G

AA1 120H;Interacts with:115T

AA1 129R;Interacts with:117G

MUT1 R129C;828636;

AA1 132T;Interacts with:117G

AA1 133V;Interacts with:117G

AA1 135E;Interacts with:120Y

AA1 136E;Interacts with:120Y

MUT1 E136R;PMID:11461956;

AA1 98R;Interacts with:100R

AA1 99Q;Interacts with:100R,101Y

AA2 100R;Interacts with:103D,102D,99Q,98R

AA2 101Y;Interacts with:99Q

AA2 115T;Interacts with:118Q,120H

AA2 116P;Interacts with:118Q

AA2 117G;Interacts with:132T,129R,115M,133V,119L,118Q

AA2 120Y;Interacts with:135E,136E

AA2 91R;Interacts with:107R

AA2 92E;Interacts with:108Y,107R

AA2 95D;Interacts with:107R

AA2 96E;Interacts with:107R,104F,106R

AA2 97F;Interacts with:103D

AA2 99L;Interacts with:103D

//

MESH1 mesh:D002277

NAME1 Carcinoma

CLUSTER1 clust2

MESH2 mesh:D008175

NAME2 Lung Neoplasms

CLUSTER2 clust2

AC1 P10415

PFAM1 PF02180

PDB1 4AQ3:A

AC2 Q07817

PFAM2 PF00452

PDB2 4AQ3:A

INT non-edgetic

AA1 11N;Interacts with:188W,171A,175N,174L,179E

AA1 12R;Interacts with:171A,175N

MUT1 R12Q;PMID:9463381;

AA1 14I;Interacts with:90L,188W,144F

AA1 15V;Interacts with:170M,174L,167A,144F,171A

AA1 16M;Interacts with:167A

AA1 17K;Interacts with:91R

AA1 18Y;Interacts with:145S,144F,90L,91R,148G,170M,95D

AA1 19I;Interacts with:163V,167A,170M

AA1 21Y;Interacts with:91R

AA1 22K;Interacts with:95D,152V,145S,98E,91R

AA1 23L;Interacts with:155V,163V,152V,151C

AA1 25Q;Interacts with:95D,183Q

AA1 26R;Interacts with:179E,184E,180P,183Q

AA1 27G;Interacts with:183Q

AA1 28Y;Interacts with:155V

AA1 30W;Interacts with:167A,163V,164S,160Q

AA1 92V;Interacts with:90L

AA1 9Y;Interacts with:188W

AA2 144F;Interacts with:18Y,15V,14I

AA2 145S;Interacts with:18Y,22K

AA2 148G;Interacts with:18Y

MUT2 G148E;827593;

AA2 151C;Interacts with:23L

AA2 152V;Interacts with:23L,22K

AA2 155V;Interacts with:23L,28Y

AA2 160Q;Interacts with:30W

AA2 163V;Interacts with:19I,23L,30W

AA2 164S;Interacts with:30W

AA2 167A;Interacts with:15V,19I,30W,16M

MUT2 A167V;1KG:1359392;

AA2 170M;Interacts with:15V,19I,18Y

AA2 171A;Interacts with:11N,15V,12R

AA2 174L;Interacts with:15V,11N

AA2 175N;Interacts with:11N,12R

AA2 179E;Interacts with:26R,11N

AA2 180P;Interacts with:26R

AA2 183Q;Interacts with:25Q,27G,26R

AA2 184E;Interacts with:26R

AA2 188W;Interacts with:11N,14I,9Y

AA2 90L;Interacts with:14I,18Y,92V

AA2 91R;Interacts with:17K,21Y,18Y,22K

AA2 95D;Interacts with:22K,25Q,18Y

AA2 98E;Interacts with:22K

//

MESH1 mesh:D002277

NAME1 Carcinoma

CLUSTER1 clust2

MESH2 mesh:D008175

NAME2 Lung Neoplasms

CLUSTER2 clust2

AC1 O43521

PFAM1 PF08945

PDB1 3FDL:B

AC2 Q07817

PFAM2 PF00452

PDB2 3FDL:B

INT non-edgetic

AA1 143R;Interacts with:111Q

MUT1 R143H;dbSNP:rs141962978;dbSNP:rs141962978;dbSNP:rs141962978;dbSNP:rs141962978;dbSNP:rs141962978;

AA1 144P;Interacts with:112L,111Q

AA1 145E;Interacts with:126V,113H,125Q,122S,112L

AA1 146I;Interacts with:125Q

AA1 147W;Interacts with:107D,108L

AA1 148I;Interacts with:126V,108L,146F,111Q,112L,104A

AA1 149A;Interacts with:126V,129E,130L

AA1 150Q;Interacts with:129E

AA1 151E;Interacts with:108L

AA1 152L;Interacts with:142A,130L,97F,146F,105F,108L,126V,145S,104A

AA1 153R;Interacts with:130L,133D,139R,129E

MUT1 R153W;dbSNP:rs146318804;dbSNP:rs146318804;dbSNP:rs146318804;dbSNP:rs146318804;dbSNP:rs146318804;

AA1 155I;Interacts with:97F,101Y,105F,108L,104A

AA1 156G;Interacts with:136N,97F,142A,139R,138G,101Y

AA1 157D;Interacts with:138G,139R,136N

AA1 158E;Interacts with:100R,101Y

AA1 159F;Interacts with:97F,96E,93A,141V,138G,101Y,100R

AA1 160N;Interacts with:137W,136N,138G,139R

AA1 162Y;Interacts with:100R

AA1 163Y;Interacts with:100R

AA1 1M;Interacts with:113H,111Q,112L

AA2 100R;Interacts with:158E,159F,162Y,163Y

AA2 101Y;Interacts with:155I,159F,158E,156G

AA2 104A;Interacts with:155I,152L,148I

AA2 105F;Interacts with:155I,152L

AA2 107D;Interacts with:147W

AA2 108L;Interacts with:155I,147W,148I,152L,151E

MUT2 L108R;dbSNP:rs139457299;dbSNP:rs139457299;

AA2 111Q;Interacts with:143R,148I,1M,144P

AA2 112L;Interacts with:148I,1M,145E,144P

AA2 113H;Interacts with:1M,145E

AA2 122S;Interacts with:145E

AA2 125Q;Interacts with:145E,146I

AA2 126V;Interacts with:145E,149A,148I,152L

AA2 129E;Interacts with:149A,150Q,153R

AA2 130L;Interacts with:153R,152L,149A

AA2 133D;Interacts with:153R

AA2 136N;Interacts with:156G,160N,157D

AA2 137W;Interacts with:160N

AA2 138G;Interacts with:157D,160N,159F,156G

MUT2 G138A;827592;

AA2 139R;Interacts with:157D,156G,153R,160N

AA2 141V;Interacts with:159F

AA2 142A;Interacts with:156G,152L

AA2 145S;Interacts with:152L

AA2 146F;Interacts with:152L,148I

AA2 93A;Interacts with:159F

AA2 96E;Interacts with:159F

AA2 97F;Interacts with:155I,159F,156G,152L

//

MESH1 mesh:D002277

NAME1 Carcinoma

CLUSTER1 clust2

MESH2 mesh:D008175

NAME2 Lung Neoplasms

CLUSTER2 clust2

AC1 Q13794

PFAM1 PF15150

PDB1 3MQP:B

AC2 Q16548

PFAM2 PF00452

PDB2 3MQP:B

INT non-edgetic

AA1 21L;Interacts with:55C,59V,58N

AA1 22E;Interacts with:74V,73Q

MUT1 E22K;dbSNP:rs1126436;

AA1 24E;Interacts with:52L

AA1 25C;Interacts with:74V,52L

AA1 26A;Interacts with:77K,78E,74V

AA1 28Q;Interacts with:51N

AA1 29L;Interacts with:91T,48V,74V,78E,95F

MUT1 L29A;823237;

MUT1 L29E;823238;

AA1 30R;Interacts with:78E,81D,77K,80E,88R,91T

MUT1 R30delR;COSMIC:166155;

AA1 32F;Interacts with:47E,91T,48V

MUT1 F32I;823240;

MUT1 F32E;823239;

AA1 33G;Interacts with:91T,88R,85N,87G,78E

AA1 34D;Interacts with:87G,85N,88R

AA1 36L;Interacts with:87G,44V,40V,90V

MUT1 L36E;823242;

AA1 37N;Interacts with:86W,87G,88R,85N

AA2 40V;Interacts with:36L

AA2 44V;Interacts with:36L

AA2 47E;Interacts with:32F

AA2 48V;Interacts with:32F,29L

AA2 51N;Interacts with:28Q

AA2 52L;Interacts with:25C,24E

AA2 55C;Interacts with:21L

AA2 58N;Interacts with:21L

MUT2 N58S;dbSNP:rs141166047;dbSNP:rs141166047;

AA2 59V;Interacts with:21L

AA2 73Q;Interacts with:22E

AA2 74V;Interacts with:25C,29L,26A,22E

AA2 77K;Interacts with:26A,30R

AA2 78E;Interacts with:26A,30R,29L,33G

AA2 80E;Interacts with:30R

AA2 81D;Interacts with:30R

AA2 85N;Interacts with:34D,33G,37N

AA2 86W;Interacts with:37N

AA2 87G;Interacts with:36L,34D,37N,33G

AA2 88R;Interacts with:33G,37N,30R,34D

AA2 90V;Interacts with:36L

AA2 91T;Interacts with:29L,32F,33G,30R

AA2 95F;Interacts with:29L

//

MESH1 mesh:D002277

NAME1 Carcinoma

CLUSTER1 clust2

MESH2 mesh:D008175

NAME2 Lung Neoplasms

CLUSTER2 clust2

AC1 O43521

PFAM1 PF08945

PDB1 2VM6:B

AC2 Q16548

PFAM2 PF00452

PDB2 2VM6:B

INT non-edgetic

AA1 142M;Interacts with:55C,58N

MUT1 M142R;dbSNP:rs114585494;dbSNP:rs114585494;dbSNP:rs114585494;dbSNP:rs114585494;dbSNP:rs114585494;

AA1 144P;Interacts with:70L,74V

AA1 145E;Interacts with:70L,77K,73Q,74V

AA1 147W;Interacts with:54S,55C,52L,51N

AA1 148I;Interacts with:70L,74V,52L,95F,56L,59V

AA1 149A;Interacts with:77K,78E,74V

AA1 151E;Interacts with:51N,48V

AA1 152L;Interacts with:78E,48V,95F,91T,74V

AA1 153R;Interacts with:79F,88R,80E,78E,81D,91T,77K

MUT1 R153W;dbSNP:rs146318804;dbSNP:rs146318804;dbSNP:rs146318804;dbSNP:rs146318804;dbSNP:rs146318804;

AA1 154R;Interacts with:47E,51N

AA1 155I;Interacts with:48V,91T,44V,47E

AA1 156G;Interacts with:87G,91T,85N,88R

AA1 157D;Interacts with:88R,87G,85N

AA1 159F;Interacts with:40V,43S,44V,87G

AA1 160N;Interacts with:86W,87G,85N

AA1 163Y;Interacts with:40V

AA2 40V;Interacts with:163Y,159F

AA2 43S;Interacts with:159F

AA2 44V;Interacts with:155I,159F

AA2 47E;Interacts with:154R,155I

AA2 48V;Interacts with:155I,152L,151E

AA2 51N;Interacts with:147W,154R,151E

AA2 52L;Interacts with:148I,147W

AA2 54S;Interacts with:147W

AA2 55C;Interacts with:147W,142M

AA2 56L;Interacts with:148I

AA2 58N;Interacts with:142M

MUT2 N58S;dbSNP:rs141166047;dbSNP:rs141166047;

AA2 59V;Interacts with:148I

AA2 70L;Interacts with:145E,148I,144P

AA2 73Q;Interacts with:145E

AA2 74V;Interacts with:148I,144P,152L,149A,145E

AA2 77K;Interacts with:149A,145E,153R

AA2 78E;Interacts with:152L,153R,149A

AA2 79F;Interacts with:153R

AA2 80E;Interacts with:153R

AA2 81D;Interacts with:153R

AA2 85N;Interacts with:157D,156G,160N

AA2 86W;Interacts with:160N

AA2 87G;Interacts with:157D,156G,160N,159F

AA2 88R;Interacts with:157D,153R,156G

AA2 91T;Interacts with:152L,155I,156G,153R

AA2 95F;Interacts with:152L,148I

//

MESH1 mesh:D002277

NAME1 Carcinoma

CLUSTER1 clust2

MESH2 mesh:D008175

NAME2 Lung Neoplasms

CLUSTER2 clust2

AC1 O43521

PFAM1 PF08945

PDB1 2NL9:B

AC2 Q07820

PFAM2 PF00452

PDB2 2NL9:B

INT semi-edgetic

AA1 143R;Interacts with:234K

MUT1 R143H;dbSNP:rs141962978;dbSNP:rs141962978;dbSNP:rs141962978;dbSNP:rs141962978;dbSNP:rs141962978;

AA1 144P;Interacts with:234K,235L

AA1 145E;Interacts with:248R,249V,245S,252H,235L

AA1 146I;Interacts with:252H

AA1 147W;Interacts with:230G,231M

AA1 148I;Interacts with:270F,235L,234K,249V,231M

AA1 149A;Interacts with:249V,252H,253V

AA1 151E;Interacts with:231M

AA1 152L;Interacts with:228F,270F,253V,249V,266T,267L,231M

AA1 153R;Interacts with:252H,253V,254F,255S,256D,263R

MUT1 R153W;dbSNP:rs146318804;dbSNP:rs146318804;dbSNP:rs146318804;dbSNP:rs146318804;dbSNP:rs146318804;

AA1 155I;Interacts with:266T,220V,228F,227A,224H,231M

AA1 156G;Interacts with:262G,260N,266T,263R

AA1 157D;Interacts with:260N,263R,262G

AA1 158E;Interacts with:224H

AA1 159F;Interacts with:265V,220V,262G,216V,219G,224H

AA1 160N;Interacts with:261W,262G,260N,263R

AA1 163Y;Interacts with:215R,216V

AA2 215R;Interacts with:163Y

AA2 216V;Interacts with:159F,163Y

AA2 219G;Interacts with:159F

AA2 220V;Interacts with:159F,155I

AA2 224H;Interacts with:158E,155I,159F

AA2 227A;Interacts with:155I

MUT2 A227V;169059;dbSNP:rs11580946;humsavar:VAR_024022;rs11580946;

AA2 228F;Interacts with:152L,155I

AA2 230G;Interacts with:147W

AA2 231M;Interacts with:147W,151E,148I,152L,155I

MUT2 M231L;898693;dbSNP:rs140449444;humsavar:VAR_054157;

AA2 234K;Interacts with:148I,144P,143R

MUT2 K234R;898704;

AA2 235L;Interacts with:148I,145E,144P

AA2 245S;Interacts with:145E

AA2 248R;Interacts with:145E

AA2 249V;Interacts with:149A,152L,145E,148I

AA2 252H;Interacts with:153R,149A,146I,145E

AA2 253V;Interacts with:152L,153R,149A

AA2 254F;Interacts with:153R

AA2 255S;Interacts with:153R

AA2 256D;Interacts with:153R

AA2 260N;Interacts with:156G,157D,160N

AA2 261W;Interacts with:160N

AA2 262G;Interacts with:159F,156G,160N,157D

AA2 263R;Interacts with:160N,156G,153R,157D

AA2 265V;Interacts with:159F

AA2 266T;Interacts with:155I,152L,156G

AA2 267L;Interacts with:152L

AA2 270F;Interacts with:148I,152L

//

MESH1 mesh:D002277

NAME1 Carcinoma

CLUSTER1 clust2

MESH2 mesh:D008175

NAME2 Lung Neoplasms

CLUSTER2 clust2

AC1 O43521

PFAM1 PF08945

PDB1 2K7W:B

AC2 Q07812

PFAM2 PF00452

PDB2 2K7W:B

INT semi-edgetic

AA1 145E;Interacts with:146E,142D,89R

AA1 146I;Interacts with:142D

AA1 148I;Interacts with:142D

AA1 149A;Interacts with:138G,142D,139W

AA1 152L;Interacts with:142D,141L,145R,138G

AA1 153R;Interacts with:138G,131E,135T,134R

MUT1 R153W;dbSNP:rs146318804;dbSNP:rs146318804;dbSNP:rs146318804;dbSNP:rs146318804;dbSNP:rs146318804;

AA1 156G;Interacts with:141L,137M,134R

AA1 157D;Interacts with:134R

AA1 160N;Interacts with:134R

AA2 131E;Interacts with:153R

AA2 134R;Interacts with:157D,153R,156G,160N

AA2 135T;Interacts with:153R

AA2 137M;Interacts with:156G

AA2 138G;Interacts with:149A,153R,152L

AA2 139W;Interacts with:149A

MUT2 W139C;COSMIC:267934;

AA2 141L;Interacts with:156G,152L

AA2 142D;Interacts with:145E,149A,152L,148I,146I

AA2 145R;Interacts with:152L

AA2 146E;Interacts with:145E

AA2 89R;Interacts with:145E

MUT2 R89Q;1KG:1191554;1KG:1224249;1KG:1224241;

//

MESH1 mesh:D002277

NAME1 Carcinoma

CLUSTER1 clust2

MESH2 mesh:D008175

NAME2 Lung Neoplasms

CLUSTER2 clust2

AC1 Q07817

PFAM1 PF00452

PDB1 2P1L:A

AC2 Q14457

PFAM2 PF15285

PDB2 2P1L:A

INT semi-edgetic

AA1 100R;Interacts with:122L,119T,123F

AA1 101Y;Interacts with:116L,115R,119T,112L

AA1 105F;Interacts with:115R,118V

AA1 106S;Interacts with:115R

AA1 108L;Interacts with:116L,112L

MUT1 L108R;dbSNP:rs139457299;dbSNP:rs139457299;

AA1 111Q;Interacts with:115R,108T,111N,112L

AA1 112L;Interacts with:108T,109M,112L

AA1 113H;Interacts with:3G,108T,109M

AA1 122S;Interacts with:109M

AA1 125Q;Interacts with:4S,106G

AA1 126V;Interacts with:109M,112L,113S,116L

AA1 129E;Interacts with:113S,117K,114R

AA1 130L;Interacts with:117K,116L,113S

AA1 133D;Interacts with:117K

AA1 136N;Interacts with:120G,124D,121D,125I

AA1 137W;Interacts with:124D,125I,126M

AA1 138G;Interacts with:124D,123F,121D,120G

MUT1 G138A;827592;

AA1 139R;Interacts with:121D,120G,117K,124D

AA1 141V;Interacts with:123F

AA1 142A;Interacts with:116L,120G

AA1 146F;Interacts with:112L,116L

AA1 185N;Interacts with:126M

AA1 93A;Interacts with:123F

AA1 96E;Interacts with:123F

AA1 97F;Interacts with:119T,123F,120G,116L

AA2 106G;Interacts with:125Q

AA2 108T;Interacts with:112L,111Q,113H

AA2 109M;Interacts with:126V,122S,112L,113H

AA2 111N;Interacts with:111Q

AA2 112L;Interacts with:146F,126V,108L,112L,101Y,111Q

AA2 113S;Interacts with:129E,126V,130L

MUT2 S113R;dbSNP:rs80236238;

AA2 114R;Interacts with:129E

AA2 115R;Interacts with:111Q,105F,101Y,106S

AA2 116L;Interacts with:101Y,130L,142A,146F,108L,126V,97F

AA2 117K;Interacts with:130L,139R,133D,129E

AA2 118V;Interacts with:105F

AA2 119T;Interacts with:97F,100R,101Y

AA2 120G;Interacts with:139R,136N,97F,142A,138G

AA2 121D;Interacts with:139R,138G,136N

AA2 122L;Interacts with:100R

AA2 123F;Interacts with:97F,93A,141V,138G,96E,100R

AA2 124D;Interacts with:138G,136N,137W,139R

AA2 125I;Interacts with:137W,136N

AA2 126M;Interacts with:185N,137W

AA2 3G;Interacts with:113H

AA2 4S;Interacts with:125Q

//

MESH1 mesh:D002277

NAME1 Carcinoma

CLUSTER1 clust2

MESH2 mesh:D009369

NAME2 Neoplasms

CLUSTER2 clust2

AC1 O43521

PFAM1 PF08945

PDB1 2NL9:B

AC2 Q07820

PFAM2 PF00452

PDB2 2NL9:B

INT semi-edgetic

AA1 143R;Interacts with:234K

MUT1 R143H;dbSNP:rs141962978;dbSNP:rs141962978;dbSNP:rs141962978;dbSNP:rs141962978;dbSNP:rs141962978;

AA1 144P;Interacts with:234K,235L

AA1 145E;Interacts with:248R,249V,245S,252H,235L

AA1 146I;Interacts with:252H

AA1 147W;Interacts with:230G,231M

AA1 148I;Interacts with:270F,235L,234K,249V,231M

AA1 149A;Interacts with:249V,252H,253V

AA1 151E;Interacts with:231M

AA1 152L;Interacts with:228F,270F,253V,249V,266T,267L,231M

AA1 153R;Interacts with:252H,253V,254F,255S,256D,263R

MUT1 R153W;dbSNP:rs146318804;dbSNP:rs146318804;dbSNP:rs146318804;dbSNP:rs146318804;dbSNP:rs146318804;

AA1 155I;Interacts with:266T,220V,228F,227A,224H,231M

AA1 156G;Interacts with:262G,260N,266T,263R

AA1 157D;Interacts with:260N,263R,262G

AA1 158E;Interacts with:224H

AA1 159F;Interacts with:265V,220V,262G,216V,219G,224H

AA1 160N;Interacts with:261W,262G,260N,263R

AA1 163Y;Interacts with:215R,216V

AA2 215R;Interacts with:163Y

AA2 216V;Interacts with:159F,163Y

AA2 219G;Interacts with:159F

AA2 220V;Interacts with:159F,155I

AA2 224H;Interacts with:158E,155I,159F

AA2 227A;Interacts with:155I

MUT2 A227V;169059;dbSNP:rs11580946;humsavar:VAR_024022;rs11580946;

AA2 228F;Interacts with:152L,155I

AA2 230G;Interacts with:147W

AA2 231M;Interacts with:147W,151E,148I,152L,155I

MUT2 M231L;898693;dbSNP:rs140449444;humsavar:VAR_054157;

AA2 234K;Interacts with:148I,144P,143R

MUT2 K234R;898704;

AA2 235L;Interacts with:148I,145E,144P

AA2 245S;Interacts with:145E

AA2 248R;Interacts with:145E

AA2 249V;Interacts with:149A,152L,145E,148I

AA2 252H;Interacts with:153R,149A,146I,145E

AA2 253V;Interacts with:152L,153R,149A

AA2 254F;Interacts with:153R

AA2 255S;Interacts with:153R

AA2 256D;Interacts with:153R

AA2 260N;Interacts with:156G,157D,160N

AA2 261W;Interacts with:160N

AA2 262G;Interacts with:159F,156G,160N,157D

AA2 263R;Interacts with:160N,156G,153R,157D

AA2 265V;Interacts with:159F

AA2 266T;Interacts with:155I,152L,156G

AA2 267L;Interacts with:152L

AA2 270F;Interacts with:148I,152L

//

MESH1 mesh:D002277

NAME1 Carcinoma

CLUSTER1 clust2

MESH2 mesh:D009369

NAME2 Neoplasms

CLUSTER2 clust2

AC1 Q13794

PFAM1 PF15150

PDB1 3MQP:B

AC2 Q16548

PFAM2 PF00452

PDB2 3MQP:B

INT semi-edgetic

AA1 21L;Interacts with:55C,59V,58N

AA1 22E;Interacts with:74V,73Q

MUT1 E22K;dbSNP:rs1126436;

AA1 24E;Interacts with:52L

AA1 25C;Interacts with:74V,52L

AA1 26A;Interacts with:77K,78E,74V

AA1 28Q;Interacts with:51N

AA1 29L;Interacts with:91T,48V,74V,78E,95F

MUT1 L29A;823237;

MUT1 L29E;823238;

AA1 30R;Interacts with:78E,81D,77K,80E,88R,91T

MUT1 R30delR;COSMIC:166155;

AA1 32F;Interacts with:47E,91T,48V

MUT1 F32I;823240;

MUT1 F32E;823239;

AA1 33G;Interacts with:91T,88R,85N,87G,78E

AA1 34D;Interacts with:87G,85N,88R

AA1 36L;Interacts with:87G,44V,40V,90V

MUT1 L36E;823242;

AA1 37N;Interacts with:86W,87G,88R,85N

AA2 40V;Interacts with:36L

AA2 44V;Interacts with:36L

AA2 47E;Interacts with:32F

AA2 48V;Interacts with:32F,29L

AA2 51N;Interacts with:28Q

AA2 52L;Interacts with:25C,24E

AA2 55C;Interacts with:21L

AA2 58N;Interacts with:21L

MUT2 N58S;dbSNP:rs141166047;dbSNP:rs141166047;

AA2 59V;Interacts with:21L

AA2 73Q;Interacts with:22E

AA2 74V;Interacts with:25C,29L,26A,22E

AA2 77K;Interacts with:26A,30R

AA2 78E;Interacts with:26A,30R,29L,33G

AA2 80E;Interacts with:30R

AA2 81D;Interacts with:30R

AA2 85N;Interacts with:34D,33G,37N

AA2 86W;Interacts with:37N

AA2 87G;Interacts with:36L,34D,37N,33G

AA2 88R;Interacts with:33G,37N,30R,34D

AA2 90V;Interacts with:36L

AA2 91T;Interacts with:29L,32F,33G,30R

AA2 95F;Interacts with:29L

//

MESH1 mesh:D002277

NAME1 Carcinoma

CLUSTER1 clust2

MESH2 mesh:D009369

NAME2 Neoplasms

CLUSTER2 clust2

AC1 P55957

PFAM1 PF06393

PDB1 2KBW:B

AC2 Q07820

PFAM2 PF00452

PDB2 2KBW:A

INT semi-edgetic

AA1 79Q;Interacts with:242D,236D,245S,235L

AA1 82I;Interacts with:234K,235L

AA1 83I;Interacts with:245S,249V,235L,248R

AA1 84R;Interacts with:252H

MUT1 R84W;dbSNP:rs148107209;dbSNP:rs148107209;

AA1 86I;Interacts with:235L,249V,234K,231M

AA1 87A;Interacts with:252H,253V,249V

AA1 88R;Interacts with:253V

AA1 89H;Interacts with:231M

AA1 90L;Interacts with:253V,231M,249V,266T,270F,267L

AA1 91A;Interacts with:253V,263R

AA1 93V;Interacts with:266T,231M,227A,224H

AA1 94G;Interacts with:263R,262G,266T,260N

MUT1 G94E;PMID:11121101;

AA1 95D;Interacts with:263R,266T,262G,260N

AA1 97M;Interacts with:266T,265V,216V,262G,220V

AA1 98D;Interacts with:261W,263R,262G,260N

AA2 216V;Interacts with:97M

AA2 220V;Interacts with:97M

AA2 224H;Interacts with:93V

AA2 227A;Interacts with:93V

MUT2 A227V;169059;dbSNP:rs11580946;humsavar:VAR_024022;rs11580946;

AA2 231M;Interacts with:93V,90L,89H,86I

MUT2 M231L;898693;dbSNP:rs140449444;humsavar:VAR_054157;

AA2 234K;Interacts with:82I,86I

MUT2 K234R;898704;

AA2 235L;Interacts with:86I,82I,83I,79Q

AA2 236D;Interacts with:79Q

AA2 242D;Interacts with:79Q

AA2 245S;Interacts with:83I,79Q

AA2 248R;Interacts with:83I

AA2 249V;Interacts with:86I,83I,90L,87A

AA2 252H;Interacts with:84R,87A

AA2 253V;Interacts with:88R,90L,91A,87A

AA2 260N;Interacts with:95D,98D,94G

AA2 261W;Interacts with:98D

AA2 262G;Interacts with:95D,97M,94G,98D

AA2 263R;Interacts with:95D,98D,91A,94G

AA2 265V;Interacts with:97M

AA2 266T;Interacts with:93V,97M,90L,95D,94G

AA2 267L;Interacts with:90L

AA2 270F;Interacts with:90L

//

MESH1 mesh:D002277

NAME1 Carcinoma

CLUSTER1 clust2

MESH2 mesh:D009369

NAME2 Neoplasms

CLUSTER2 clust2

AC1 P40337

PFAM1 PF01847

PDB1 1LM8:V

AC2 Q16665

PFAM2 PF11413

PDB2 1LM8:V

INT edgetic

AA1 103P;Interacts with:573Q

AA1 104G;Interacts with:573Q,574L

MUT1 G104A;971728;humsavar:VAR_005710;

AA1 105T;Interacts with:573Q,574L,572F

MUT1 T105P;971729;humsavar:VAR_005711, phenotype=Von Hippel-Lindau disease (VHLD);

AA1 106G;Interacts with:571D,572F,574L

MUT1 G106D;971730;humsavar:VAR_005712, phenotype=Lung cancer;

AA1 107R;Interacts with:572F,571D

MUT1 R107P;971732;COSMIC:422837;humsavar:VAR_005713, phenotype=Von Hippel-Lindau disease (VHLD);clinvar:45561, phenotype=von Hippel-Lindau syndrome, modifier of;

MUT1 R107fs*58;COSMIC:423293;

MUT1 R107G;971731;humsavar:VAR_034991, phenotype=Pheochromocytoma (PCC);clinvar:52768;

AA1 108R;Interacts with:567P,566I,569D,568M

AA1 109I;Interacts with:566I,565Y

MUT1 I109S;COSMIC:30224;

MUT1 I109N;COSMIC:30221;clinvar:99235;

MUT1 I109fs*56;COSMIC:423294;

AA1 110H;Interacts with:567P,565Y,566I

MUT1 H110P;COSMIC:30410;

MUT1 H110fs*49;COSMIC:423186;

MUT1 H110Y;211342;dbSNP:rs17855706;humsavar:VAR_055087;rs17855706;

AA1 112Y;Interacts with:562L,565Y,563A

MUT1 Y112H;211349;dbSNP:rs104893824;humsavar:VAR_005717, phenotype=Von Hippel-Lindau disease (VHLD);clinvar:17261, phenotype=von Hippel-Lindau syndrome, modifier of;VON HIPPEL-LINDAU SYNDROME;

MUT1 Y112*;COSMIC:30227;

MUT1 Y112N;211348;dbSNP:rs104893824;humsavar:VAR_034992, phenotype=Von Hippel-Lindau disease (VHLD);clinvar:17267, phenotype=von Hippel-Lindau syndrome, modifier of;VON HIPPEL-LINDAU SYNDROME;

AA1 115H;Interacts with:562L,563A

MUT1 H115R;211331;humsavar:VAR_008098;rs5030812, phenotype=Von Hippel-Lindau disease (VHLD);

MUT1 H115Q;971739;humsavar:VAR_005723, phenotype=Von Hippel-Lindau disease (VHLD);

MUT1 H115Y;211330;humsavar:VAR_005722;rs5030811, phenotype=Von Hippel-Lindau disease (VHLD);

MUT1 H115N;COSMIC:17752;

AA1 67N;Interacts with:562L,1M,560E,561M

MUT1 N67fs*65;COSMIC:423289;

AA1 69R;Interacts with:562L,560E

AA1 75I;Interacts with:572F

MUT1 I75fs*54;COSMIC:423178;

AA1 77C;Interacts with:574L

AA1 79R;Interacts with:574L,576S

MUT1 R79P;971712;humsavar:VAR_005685, phenotype=Von Hippel-Lindau disease (VHLD);

AA1 88W;Interacts with:563A

MUT1 W88C;COSMIC:97142;

MUT1 W88S;211358;dbSNP:rs119103277;COSMIC:18415;humsavar:VAR_005698, phenotype=Von Hippel-Lindau disease (VHLD);clinvar:17259, phenotype=von Hippel-Lindau syndrome, modifier of;HEMANGIOBLASTOMA, SPORADIC CEREBELLAR;

MUT1 W88R;971720;COSMIC:17953;humsavar:VAR_005697, phenotype=Von Hippel-Lindau disease (VHLD);

AA1 91F;Interacts with:1M,563A,562L,561M

MUT1 F91fs*68;COSMIC:423182;

MUT1 F91L;971722;humsavar:VAR_005701;

AA1 98Y;Interacts with:565Y,566I

MUT1 Y98fs*1;COSMIC:423292;

MUT1 Y98*;COSMIC:17958;

MUT1 Y98N;971788;

MUT1 Y98H;211329;dbSNP:rs5030809;humsavar:VAR_005707;rs5030809, phenotype=Pheochromocytoma (PCC);clinvar:17262, phenotype=von Hippel-Lindau syndrome, modifier of;VON HIPPEL-LINDAU SYNDROME;

AA1 99P;Interacts with:566I

MUT1 P99fs*32;COSMIC:423184;

MUT1 P99L;COSMIC:30238;

AA2 1M;Interacts with:91F,67N

AA2 560E;Interacts with:69R,67N

MUT2 E560Q;PMID:11292861;

AA2 561M;Interacts with:91F,67N

AA2 562L;Interacts with:115H,69R,112Y,67N,91F

MUT2 L562A;PMID:11292862;

AA2 563A;Interacts with:88W,91F,115H,112Y

AA2 565Y;Interacts with:98Y,110H,112Y,109I

MUT2 Y565A;PMID:11292861;

AA2 566I;Interacts with:109I,108R,98Y,99P,110H

AA2 567P;Interacts with:108R,110H

MUT2 P567G;PMID:11292861;

AA2 568M;Interacts with:108R

MUT2 M568R;PMID:11292861;

AA2 569D;Interacts with:108R

MUT2 D569N;PMID:11292861;

AA2 571D;Interacts with:106G,107R

AA2 572F;Interacts with:106G,75I,107R,105T

MUT2 F572L;879027;

AA2 573Q;Interacts with:104G,105T,103P

AA2 574L;Interacts with:104G,77C,105T,106G,79R

AA2 576S;Interacts with:79R

MUT2 S576A;1368548;

//

MESH1 mesh:D002277

NAME1 Carcinoma

CLUSTER1 clust2

MESH2 mesh:D011471

NAME2 Prostatic Neoplasms

CLUSTER2 clust2

AC1 O43521

PFAM1 PF08945

PDB1 2K7W:B

AC2 Q07812

PFAM2 PF00452

PDB2 2K7W:B

INT semi-edgetic

AA1 145E;Interacts with:146E,142D,89R

AA1 146I;Interacts with:142D

AA1 148I;Interacts with:142D

AA1 149A;Interacts with:138G,142D,139W

AA1 152L;Interacts with:142D,141L,145R,138G

AA1 153R;Interacts with:138G,131E,135T,134R

MUT1 R153W;dbSNP:rs146318804;dbSNP:rs146318804;dbSNP:rs146318804;dbSNP:rs146318804;dbSNP:rs146318804;

AA1 156G;Interacts with:141L,137M,134R

AA1 157D;Interacts with:134R

AA1 160N;Interacts with:134R

AA2 131E;Interacts with:153R

AA2 134R;Interacts with:157D,153R,156G,160N

AA2 135T;Interacts with:153R

AA2 137M;Interacts with:156G

AA2 138G;Interacts with:149A,153R,152L

AA2 139W;Interacts with:149A

MUT2 W139C;COSMIC:267934;

AA2 141L;Interacts with:156G,152L

AA2 142D;Interacts with:145E,149A,152L,148I,146I

AA2 145R;Interacts with:152L

AA2 146E;Interacts with:145E

AA2 89R;Interacts with:145E

MUT2 R89Q;1KG:1191554;1KG:1224249;1KG:1224241;

//

MESH1 mesh:D002277

NAME1 Carcinoma

CLUSTER1 clust2

MESH2 mesh:D011471

NAME2 Prostatic Neoplasms

CLUSTER2 clust2

AC1 P10415

PFAM1 PF00452

PDB1 4AQ3:A

AC2 Q07817

PFAM2 PF00452

PDB2 4AQ3:C

INT semi-edgetic

AA1 102D;Interacts with:100R

AA1 103D;Interacts with:100R,99L,97F

AA1 104F;Interacts with:96E

AA1 106R;Interacts with:96E

AA1 107R;Interacts with:95D,96E,92E,91R

AA1 108Y;Interacts with:92E

AA1 115M;Interacts with:117G

AA1 118Q;Interacts with:116P,115T,117G

AA1 119L;Interacts with:117G

AA1 120H;Interacts with:115T

AA1 129R;Interacts with:117G

MUT1 R129C;828636;

AA1 132T;Interacts with:117G

AA1 133V;Interacts with:117G

AA1 135E;Interacts with:120Y

AA1 136E;Interacts with:120Y

MUT1 E136R;PMID:11461956;

AA1 98R;Interacts with:100R

AA1 99Q;Interacts with:100R,101Y

AA2 100R;Interacts with:103D,102D,99Q,98R

AA2 101Y;Interacts with:99Q

AA2 115T;Interacts with:118Q,120H

AA2 116P;Interacts with:118Q

AA2 117G;Interacts with:132T,129R,115M,133V,119L,118Q

AA2 120Y;Interacts with:135E,136E

AA2 91R;Interacts with:107R

AA2 92E;Interacts with:108Y,107R

AA2 95D;Interacts with:107R

AA2 96E;Interacts with:107R,104F,106R

AA2 97F;Interacts with:103D

AA2 99L;Interacts with:103D

//

MESH1 mesh:D002277

NAME1 Carcinoma

CLUSTER1 clust2

MESH2 mesh:D011471

NAME2 Prostatic Neoplasms

CLUSTER2 clust2

AC1 P10415

PFAM1 PF02180

PDB1 4AQ3:A

AC2 Q07817

PFAM2 PF00452

PDB2 4AQ3:A

INT semi-edgetic

AA1 11N;Interacts with:188W,171A,175N,174L,179E

AA1 12R;Interacts with:171A,175N

MUT1 R12Q;PMID:9463381;

AA1 14I;Interacts with:90L,188W,144F

AA1 15V;Interacts with:170M,174L,167A,144F,171A

AA1 16M;Interacts with:167A

AA1 17K;Interacts with:91R

AA1 18Y;Interacts with:145S,144F,90L,91R,148G,170M,95D

AA1 19I;Interacts with:163V,167A,170M

AA1 21Y;Interacts with:91R

AA1 22K;Interacts with:95D,152V,145S,98E,91R

AA1 23L;Interacts with:155V,163V,152V,151C

AA1 25Q;Interacts with:95D,183Q

AA1 26R;Interacts with:179E,184E,180P,183Q

AA1 27G;Interacts with:183Q

AA1 28Y;Interacts with:155V

AA1 30W;Interacts with:167A,163V,164S,160Q

AA1 92V;Interacts with:90L

AA1 9Y;Interacts with:188W

AA2 144F;Interacts with:18Y,15V,14I

AA2 145S;Interacts with:18Y,22K

AA2 148G;Interacts with:18Y

MUT2 G148E;827593;

AA2 151C;Interacts with:23L

AA2 152V;Interacts with:23L,22K

AA2 155V;Interacts with:23L,28Y

AA2 160Q;Interacts with:30W

AA2 163V;Interacts with:19I,23L,30W

AA2 164S;Interacts with:30W

AA2 167A;Interacts with:15V,19I,30W,16M

MUT2 A167V;1KG:1359392;

AA2 170M;Interacts with:15V,19I,18Y

AA2 171A;Interacts with:11N,15V,12R

AA2 174L;Interacts with:15V,11N

AA2 175N;Interacts with:11N,12R

AA2 179E;Interacts with:26R,11N

AA2 180P;Interacts with:26R

AA2 183Q;Interacts with:25Q,27G,26R

AA2 184E;Interacts with:26R

AA2 188W;Interacts with:11N,14I,9Y

AA2 90L;Interacts with:14I,18Y,92V

AA2 91R;Interacts with:17K,21Y,18Y,22K

AA2 95D;Interacts with:22K,25Q,18Y

AA2 98E;Interacts with:22K

//

MESH1 mesh:D002277

NAME1 Carcinoma

CLUSTER1 clust2

MESH2 mesh:D011471

NAME2 Prostatic Neoplasms

CLUSTER2 clust2

AC1 O14727

PFAM1 PF00619

PDB1 3YGS:C

AC2 P55211

PFAM2 PF00619

PDB2 3YGS:P

INT semi-edgetic

AA1 23S;Interacts with:56R

AA1 24Y;Interacts with:52R

MUT1 Y24C;dbSNP:rs150457288;dbSNP:rs150457288;dbSNP:rs150457288;dbSNP:rs150457288;dbSNP:rs150457288;

AA1 27D;Interacts with:52R,56R,13R,14L

AA1 28H;Interacts with:14L

AA1 30I;Interacts with:10R,11R

AA1 31S;Interacts with:12C,13R,10R,15R,14L,11R

AA1 32D;Interacts with:11R

AA1 37I;Interacts with:60I

AA1 40E;Interacts with:13R,56R

AA1 78E;Interacts with:52R

AA2 10R;Interacts with:31S,30I

AA2 11R;Interacts with:32D,30I,31S

AA2 12C;Interacts with:31S

AA2 13R;Interacts with:40E,31S,27D

MUT2 R13A;PMID:10376594;

AA2 14L;Interacts with:31S,27D,28H

AA2 15R;Interacts with:31S

AA2 52R;Interacts with:27D,24Y,78E

MUT2 R52A;PMID:10376594;

AA2 56R;Interacts with:27D,40E,23S

MUT2 R56A;PMID:10376594;

AA2 60I;Interacts with:37I

MUT2 I60L;1KG:1285113;

//

MESH1 mesh:D002277

NAME1 Carcinoma

CLUSTER1 clust2

MESH2 mesh:D011471

NAME2 Prostatic Neoplasms

CLUSTER2 clust2

AC1 Q13794

PFAM1 PF15150

PDB1 3MQP:B

AC2 Q16548

PFAM2 PF00452

PDB2 3MQP:B

INT semi-edgetic

AA1 21L;Interacts with:55C,59V,58N

AA1 22E;Interacts with:74V,73Q

MUT1 E22K;dbSNP:rs1126436;

AA1 24E;Interacts with:52L

AA1 25C;Interacts with:74V,52L

AA1 26A;Interacts with:77K,78E,74V

AA1 28Q;Interacts with:51N

AA1 29L;Interacts with:91T,48V,74V,78E,95F

MUT1 L29A;823237;

MUT1 L29E;823238;

AA1 30R;Interacts with:78E,81D,77K,80E,88R,91T

MUT1 R30delR;COSMIC:166155;

AA1 32F;Interacts with:47E,91T,48V

MUT1 F32I;823240;

MUT1 F32E;823239;

AA1 33G;Interacts with:91T,88R,85N,87G,78E

AA1 34D;Interacts with:87G,85N,88R

AA1 36L;Interacts with:87G,44V,40V,90V

MUT1 L36E;823242;

AA1 37N;Interacts with:86W,87G,88R,85N

AA2 40V;Interacts with:36L

AA2 44V;Interacts with:36L

AA2 47E;Interacts with:32F

AA2 48V;Interacts with:32F,29L

AA2 51N;Interacts with:28Q

AA2 52L;Interacts with:25C,24E

AA2 55C;Interacts with:21L

AA2 58N;Interacts with:21L

MUT2 N58S;dbSNP:rs141166047;dbSNP:rs141166047;

AA2 59V;Interacts with:21L

AA2 73Q;Interacts with:22E

AA2 74V;Interacts with:25C,29L,26A,22E

AA2 77K;Interacts with:26A,30R

AA2 78E;Interacts with:26A,30R,29L,33G

AA2 80E;Interacts with:30R

AA2 81D;Interacts with:30R

AA2 85N;Interacts with:34D,33G,37N

AA2 86W;Interacts with:37N

AA2 87G;Interacts with:36L,34D,37N,33G

AA2 88R;Interacts with:33G,37N,30R,34D

AA2 90V;Interacts with:36L

AA2 91T;Interacts with:29L,32F,33G,30R

AA2 95F;Interacts with:29L

//

MESH1 mesh:D002277

NAME1 Carcinoma

CLUSTER1 clust2

MESH2 mesh:D011471

NAME2 Prostatic Neoplasms

CLUSTER2 clust2

AC1 P55957

PFAM1 PF06393

PDB1 4BD2:C

AC2 Q07812

PFAM2 PF00452

PDB2 4BD2:A

INT semi-edgetic

AA1 102P;Interacts with:65R

AA1 103P;Interacts with:65R,66I

AA1 104G;Interacts with:66I,69E

AA1 105L;Interacts with:70L,69E,66I

MUT1 L105P;dbSNP:rs143734092;dbSNP:rs143734092;

AA1 79Q;Interacts with:82A,83V,84D

AA1 80E;Interacts with:94R

AA1 82I;Interacts with:83V,82A,79M

AA1 83I;Interacts with:91V,94R,83V,95V

AA1 84R;Interacts with:98D

MUT1 R84W;dbSNP:rs148107209;dbSNP:rs148107209;

AA1 86I;Interacts with:83V,116F,79M,80I,95V

AA1 87A;Interacts with:98D,99M,95V

AA1 89H;Interacts with:76L,75E,79M,73N

AA1 90L;Interacts with:116F,76L,95V,112A,99M,115Y

AA1 91A;Interacts with:109R,99M

AA1 93V;Interacts with:70L,112A

AA1 94G;Interacts with:108G,106N,112A,109R

MUT1 G94E;PMID:11121101;

AA1 95D;Interacts with:109R,106N

AA1 97M;Interacts with:66I,111V,108G

AA1 98D;Interacts with:106N

AA2 106N;Interacts with:98D,94G,95D

AA2 108G;Interacts with:94G,97M

MUT2 G108V;828239;humsavar:VAR_013576, phenotype=A Burkitt lymphoma;

AA2 109R;Interacts with:91A,95D,94G

AA2 111V;Interacts with:97M

AA2 112A;Interacts with:93V,94G,90L

AA2 115Y;Interacts with:90L

AA2 116F;Interacts with:90L,86I

AA2 65R;Interacts with:103P,102P

MUT2 R65L;dbSNP:rs142278713;dbSNP:rs142278713;dbSNP:rs142278713;

AA2 66I;Interacts with:104G,97M,105L,103P

AA2 69E;Interacts with:105L,104G

AA2 70L;Interacts with:105L,93V

AA2 73N;Interacts with:89H

AA2 75E;Interacts with:89H

AA2 76L;Interacts with:89H,90L

AA2 79M;Interacts with:89H,86I,82I

AA2 80I;Interacts with:86I

AA2 82A;Interacts with:79Q,82I

AA2 83V;Interacts with:82I,86I,79Q,83I

AA2 84D;Interacts with:79Q

AA2 91V;Interacts with:83I

AA2 94R;Interacts with:83I,80E

AA2 95V;Interacts with:83I,90L,87A,86I

AA2 98D;Interacts with:87A,84R

AA2 99M;Interacts with:91A,90L,87A

//

MESH1 mesh:D002277

NAME1 Carcinoma

CLUSTER1 clust2

MESH2 mesh:D011471

NAME2 Prostatic Neoplasms

CLUSTER2 clust2

AC1 P17655

PFAM1 PF01067

PDB1 1KFU:L

AC2 P04632

PFAM2 PF13833

PDB2 1KFU:S

INT edgetic

AA1 366R;Interacts with:156T,154D,155T

AA1 416R;Interacts with:154D

AA1 417R;Interacts with:114E,112D,113M,163E,160G,111D

MUT1 R417Q;dbSNP:rs148370103;

AA1 418R;Interacts with:112D,111D

MUT1 R418Q;COSMIC:1199521;

AA1 420R;Interacts with:111D

AA1 494D;Interacts with:158K

AA2 111D;Interacts with:418R,420R,417R

AA2 112D;Interacts with:417R,418R

AA2 113M;Interacts with:417R

AA2 114E;Interacts with:417R

AA2 154D;Interacts with:366R,416R

AA2 155T;Interacts with:366R

AA2 156T;Interacts with:366R

AA2 158K;Interacts with:494D

AA2 160G;Interacts with:417R

AA2 163E;Interacts with:417R

//

MESH1 mesh:D002277

NAME1 Carcinoma

CLUSTER1 clust2

MESH2 mesh:D011471

NAME2 Prostatic Neoplasms

CLUSTER2 clust2

AC1 P63098

PFAM1 PF13499

PDB1 3LL8:B

AC2 Q08209

PFAM2 PF00149

PDB2 3LL8:C

INT edgetic

AA1 122N;Interacts with:247H,245Q,246E

AA1 123N;Interacts with:249T,247H,248F,246E

AA1 124L;Interacts with:248F,250H

AA1 125K;Interacts with:246E

AA2 245Q;Interacts with:122N

AA2 246E;Interacts with:122N,125K,123N

AA2 247H;Interacts with:123N,122N

AA2 248F;Interacts with:123N,124L

AA2 249T;Interacts with:123N

AA2 250H;Interacts with:124L

//

MESH1 mesh:D002277

NAME1 Carcinoma

CLUSTER1 clust2

MESH2 mesh:D011471

NAME2 Prostatic Neoplasms

CLUSTER2 clust2

AC1 Q07817

PFAM1 PF00452

PDB1 1G5J:A

AC2 Q92934

PFAM2 PF10514

PDB2 1G5J:B

INT edgetic

AA1 100R;Interacts with:121F,120E

AA1 101Y;Interacts with:117M

AA1 104A;Interacts with:110Y,114L

AA1 105F;Interacts with:110Y

AA1 108L;Interacts with:110Y

MUT1 L108R;dbSNP:rs139457299;dbSNP:rs139457299;

AA1 111Q;Interacts with:105W,106A

AA1 112L;Interacts with:107A,106A,104L

AA1 113H;Interacts with:103N

AA1 121Q;Interacts with:104L

AA1 122S;Interacts with:107A,104L

AA1 125Q;Interacts with:107A,108Q

AA1 126V;Interacts with:107A,111G,110Y

AA1 129E;Interacts with:112R,111G,115R

AA1 130L;Interacts with:111G,115R,114L

AA1 133D;Interacts with:115R

AA1 138G;Interacts with:121F,122V

MUT1 G138A;827592;

AA1 139R;Interacts with:115R,119D,118S

AA1 141V;Interacts with:121F

AA1 142A;Interacts with:114L,121F

AA1 146F;Interacts with:111G,110Y,114L

AA1 93A;Interacts with:121F

AA1 96E;Interacts with:121F

AA1 97F;Interacts with:117M,121F,118S,114L

AA2 103N;Interacts with:113H

AA2 104L;Interacts with:122S,112L,121Q

AA2 105W;Interacts with:111Q

AA2 106A;Interacts with:112L,111Q

AA2 107A;Interacts with:112L,122S,126V,125Q

MUT2 A107S;119982;humsavar:VAR_015380;rs3729933;

AA2 108Q;Interacts with:125Q

AA2 110Y;Interacts with:104A,146F,108L,105F,126V

AA2 111G;Interacts with:130L,146F,129E,126V

AA2 112R;Interacts with:129E

AA2 114L;Interacts with:104A,142A,130L,146F,97F

AA2 115R;Interacts with:133D,139R,129E,130L

AA2 117M;Interacts with:97F,101Y

AA2 118S;Interacts with:97F,139R

AA2 119D;Interacts with:139R

AA2 120E;Interacts with:100R

AA2 121F;Interacts with:100R,138G,141V,97F,96E,93A,142A

AA2 122V;Interacts with:138G

//

MESH1 mesh:D002277

NAME1 Carcinoma

CLUSTER1 clust2

MESH2 mesh:D011471

NAME2 Prostatic Neoplasms

CLUSTER2 clust2

AC1 P40337

PFAM1 PF01847

PDB1 1LM8:V

AC2 Q16665

PFAM2 PF11413

PDB2 1LM8:V

INT edgetic

AA1 103P;Interacts with:573Q

AA1 104G;Interacts with:573Q,574L

MUT1 G104A;971728;humsavar:VAR_005710;

AA1 105T;Interacts with:573Q,574L,572F

MUT1 T105P;971729;humsavar:VAR_005711, phenotype=Von Hippel-Lindau disease (VHLD);

AA1 106G;Interacts with:571D,572F,574L

MUT1 G106D;971730;humsavar:VAR_005712, phenotype=Lung cancer;

AA1 107R;Interacts with:572F,571D

MUT1 R107P;971732;COSMIC:422837;humsavar:VAR_005713, phenotype=Von Hippel-Lindau disease (VHLD);clinvar:45561, phenotype=von Hippel-Lindau syndrome, modifier of;

MUT1 R107fs*58;COSMIC:423293;

MUT1 R107G;971731;humsavar:VAR_034991, phenotype=Pheochromocytoma (PCC);clinvar:52768;

AA1 108R;Interacts with:567P,566I,569D,568M

AA1 109I;Interacts with:566I,565Y

MUT1 I109S;COSMIC:30224;

MUT1 I109N;COSMIC:30221;clinvar:99235;

MUT1 I109fs*56;COSMIC:423294;

AA1 110H;Interacts with:567P,565Y,566I

MUT1 H110P;COSMIC:30410;

MUT1 H110fs*49;COSMIC:423186;

MUT1 H110Y;211342;dbSNP:rs17855706;humsavar:VAR_055087;rs17855706;

AA1 112Y;Interacts with:562L,565Y,563A

MUT1 Y112H;211349;dbSNP:rs104893824;humsavar:VAR_005717, phenotype=Von Hippel-Lindau disease (VHLD);clinvar:17261, phenotype=von Hippel-Lindau syndrome, modifier of;VON HIPPEL-LINDAU SYNDROME;

MUT1 Y112*;COSMIC:30227;

MUT1 Y112N;211348;dbSNP:rs104893824;humsavar:VAR_034992, phenotype=Von Hippel-Lindau disease (VHLD);clinvar:17267, phenotype=von Hippel-Lindau syndrome, modifier of;VON HIPPEL-LINDAU SYNDROME;

AA1 115H;Interacts with:562L,563A

MUT1 H115R;211331;humsavar:VAR_008098;rs5030812, phenotype=Von Hippel-Lindau disease (VHLD);

MUT1 H115Q;971739;humsavar:VAR_005723, phenotype=Von Hippel-Lindau disease (VHLD);

MUT1 H115Y;211330;humsavar:VAR_005722;rs5030811, phenotype=Von Hippel-Lindau disease (VHLD);

MUT1 H115N;COSMIC:17752;

AA1 67N;Interacts with:562L,1M,560E,561M

MUT1 N67fs*65;COSMIC:423289;

AA1 69R;Interacts with:562L,560E

AA1 75I;Interacts with:572F

MUT1 I75fs*54;COSMIC:423178;

AA1 77C;Interacts with:574L

AA1 79R;Interacts with:574L,576S

MUT1 R79P;971712;humsavar:VAR_005685, phenotype=Von Hippel-Lindau disease (VHLD);

AA1 88W;Interacts with:563A

MUT1 W88C;COSMIC:97142;

MUT1 W88S;211358;dbSNP:rs119103277;COSMIC:18415;humsavar:VAR_005698, phenotype=Von Hippel-Lindau disease (VHLD);clinvar:17259, phenotype=von Hippel-Lindau syndrome, modifier of;HEMANGIOBLASTOMA, SPORADIC CEREBELLAR;

MUT1 W88R;971720;COSMIC:17953;humsavar:VAR_005697, phenotype=Von Hippel-Lindau disease (VHLD);

AA1 91F;Interacts with:1M,563A,562L,561M

MUT1 F91fs*68;COSMIC:423182;

MUT1 F91L;971722;humsavar:VAR_005701;

AA1 98Y;Interacts with:565Y,566I

MUT1 Y98fs*1;COSMIC:423292;

MUT1 Y98*;COSMIC:17958;

MUT1 Y98N;971788;

MUT1 Y98H;211329;dbSNP:rs5030809;humsavar:VAR_005707;rs5030809, phenotype=Pheochromocytoma (PCC);clinvar:17262, phenotype=von Hippel-Lindau syndrome, modifier of;VON HIPPEL-LINDAU SYNDROME;

AA1 99P;Interacts with:566I

MUT1 P99fs*32;COSMIC:423184;

MUT1 P99L;COSMIC:30238;

AA2 1M;Interacts with:91F,67N

AA2 560E;Interacts with:69R,67N

MUT2 E560Q;PMID:11292861;

AA2 561M;Interacts with:91F,67N

AA2 562L;Interacts with:115H,69R,112Y,67N,91F

MUT2 L562A;PMID:11292862;

AA2 563A;Interacts with:88W,91F,115H,112Y

AA2 565Y;Interacts with:98Y,110H,112Y,109I

MUT2 Y565A;PMID:11292861;

AA2 566I;Interacts with:109I,108R,98Y,99P,110H

AA2 567P;Interacts with:108R,110H

MUT2 P567G;PMID:11292861;

AA2 568M;Interacts with:108R

MUT2 M568R;PMID:11292861;

AA2 569D;Interacts with:108R

MUT2 D569N;PMID:11292861;

AA2 571D;Interacts with:106G,107R

AA2 572F;Interacts with:106G,75I,107R,105T

MUT2 F572L;879027;

AA2 573Q;Interacts with:104G,105T,103P

AA2 574L;Interacts with:104G,77C,105T,106G,79R

AA2 576S;Interacts with:79R

MUT2 S576A;1368548;

//

MESH1 mesh:D002277

NAME1 Carcinoma

CLUSTER1 clust2

MESH2 mesh:D015179

NAME2 Colorectal Neoplasms

CLUSTER2 clust2

AC1 P10415

PFAM1 PF00452

PDB1 4AQ3:A

AC2 Q07817

PFAM2 PF00452

PDB2 4AQ3:C

INT semi-edgetic

AA1 102D;Interacts with:100R

AA1 103D;Interacts with:100R,99L,97F

AA1 104F;Interacts with:96E

AA1 106R;Interacts with:96E

AA1 107R;Interacts with:95D,96E,92E,91R

AA1 108Y;Interacts with:92E

AA1 115M;Interacts with:117G

AA1 118Q;Interacts with:116P,115T,117G

AA1 119L;Interacts with:117G

AA1 120H;Interacts with:115T

AA1 129R;Interacts with:117G

MUT1 R129C;828636;

AA1 132T;Interacts with:117G

AA1 133V;Interacts with:117G

AA1 135E;Interacts with:120Y

AA1 136E;Interacts with:120Y

MUT1 E136R;PMID:11461956;

AA1 98R;Interacts with:100R

AA1 99Q;Interacts with:100R,101Y

AA2 100R;Interacts with:103D,102D,99Q,98R

AA2 101Y;Interacts with:99Q

AA2 115T;Interacts with:118Q,120H

AA2 116P;Interacts with:118Q

AA2 117G;Interacts with:132T,129R,115M,133V,119L,118Q

AA2 120Y;Interacts with:135E,136E

AA2 91R;Interacts with:107R

AA2 92E;Interacts with:108Y,107R

AA2 95D;Interacts with:107R

AA2 96E;Interacts with:107R,104F,106R

AA2 97F;Interacts with:103D

AA2 99L;Interacts with:103D

//

MESH1 mesh:D002277

NAME1 Carcinoma

CLUSTER1 clust2

MESH2 mesh:D015179

NAME2 Colorectal Neoplasms

CLUSTER2 clust2

AC1 P10415

PFAM1 PF02180

PDB1 4AQ3:A

AC2 Q07817

PFAM2 PF00452

PDB2 4AQ3:A

INT semi-edgetic

AA1 11N;Interacts with:188W,171A,175N,174L,179E

AA1 12R;Interacts with:171A,175N

MUT1 R12Q;PMID:9463381;

AA1 14I;Interacts with:90L,188W,144F

AA1 15V;Interacts with:170M,174L,167A,144F,171A

AA1 16M;Interacts with:167A

AA1 17K;Interacts with:91R

AA1 18Y;Interacts with:145S,144F,90L,91R,148G,170M,95D

AA1 19I;Interacts with:163V,167A,170M

AA1 21Y;Interacts with:91R

AA1 22K;Interacts with:95D,152V,145S,98E,91R

AA1 23L;Interacts with:155V,163V,152V,151C

AA1 25Q;Interacts with:95D,183Q

AA1 26R;Interacts with:179E,184E,180P,183Q

AA1 27G;Interacts with:183Q

AA1 28Y;Interacts with:155V

AA1 30W;Interacts with:167A,163V,164S,160Q

AA1 92V;Interacts with:90L

AA1 9Y;Interacts with:188W

AA2 144F;Interacts with:18Y,15V,14I

AA2 145S;Interacts with:18Y,22K

AA2 148G;Interacts with:18Y

MUT2 G148E;827593;

AA2 151C;Interacts with:23L

AA2 152V;Interacts with:23L,22K

AA2 155V;Interacts with:23L,28Y

AA2 160Q;Interacts with:30W

AA2 163V;Interacts with:19I,23L,30W

AA2 164S;Interacts with:30W

AA2 167A;Interacts with:15V,19I,30W,16M

MUT2 A167V;1KG:1359392;

AA2 170M;Interacts with:15V,19I,18Y

AA2 171A;Interacts with:11N,15V,12R

AA2 174L;Interacts with:15V,11N

AA2 175N;Interacts with:11N,12R

AA2 179E;Interacts with:26R,11N

AA2 180P;Interacts with:26R

AA2 183Q;Interacts with:25Q,27G,26R

AA2 184E;Interacts with:26R

AA2 188W;Interacts with:11N,14I,9Y

AA2 90L;Interacts with:14I,18Y,92V

AA2 91R;Interacts with:17K,21Y,18Y,22K

AA2 95D;Interacts with:22K,25Q,18Y

AA2 98E;Interacts with:22K

//

MESH1 mesh:D002277

NAME1 Carcinoma

CLUSTER1 clust2

MESH2 mesh:D015179

NAME2 Colorectal Neoplasms

CLUSTER2 clust2

AC1 Q13794

PFAM1 PF15150

PDB1 3MQP:B

AC2 Q16548

PFAM2 PF00452

PDB2 3MQP:B

INT semi-edgetic

AA1 21L;Interacts with:55C,59V,58N

AA1 22E;Interacts with:74V,73Q

MUT1 E22K;dbSNP:rs1126436;

AA1 24E;Interacts with:52L

AA1 25C;Interacts with:74V,52L

AA1 26A;Interacts with:77K,78E,74V

AA1 28Q;Interacts with:51N

AA1 29L;Interacts with:91T,48V,74V,78E,95F

MUT1 L29A;823237;

MUT1 L29E;823238;

AA1 30R;Interacts with:78E,81D,77K,80E,88R,91T

MUT1 R30delR;COSMIC:166155;

AA1 32F;Interacts with:47E,91T,48V

MUT1 F32I;823240;

MUT1 F32E;823239;

AA1 33G;Interacts with:91T,88R,85N,87G,78E

AA1 34D;Interacts with:87G,85N,88R

AA1 36L;Interacts with:87G,44V,40V,90V

MUT1 L36E;823242;

AA1 37N;Interacts with:86W,87G,88R,85N

AA2 40V;Interacts with:36L

AA2 44V;Interacts with:36L

AA2 47E;Interacts with:32F

AA2 48V;Interacts with:32F,29L

AA2 51N;Interacts with:28Q

AA2 52L;Interacts with:25C,24E

AA2 55C;Interacts with:21L

AA2 58N;Interacts with:21L

MUT2 N58S;dbSNP:rs141166047;dbSNP:rs141166047;

AA2 59V;Interacts with:21L

AA2 73Q;Interacts with:22E

AA2 74V;Interacts with:25C,29L,26A,22E

AA2 77K;Interacts with:26A,30R

AA2 78E;Interacts with:26A,30R,29L,33G

AA2 80E;Interacts with:30R

AA2 81D;Interacts with:30R

AA2 85N;Interacts with:34D,33G,37N

AA2 86W;Interacts with:37N

AA2 87G;Interacts with:36L,34D,37N,33G

AA2 88R;Interacts with:33G,37N,30R,34D

AA2 90V;Interacts with:36L

AA2 91T;Interacts with:29L,32F,33G,30R

AA2 95F;Interacts with:29L

//

MESH1 mesh:D002294

NAME1 Carcinoma, Squamous Cell

CLUSTER1 clust1

MESH2 mesh:D018805

NAME2 Sepsis

CLUSTER2 clust1

AC1 P01033

PFAM1 PF00965

PDB1 1OO9:B

AC2 P08254

PFAM2 PF00413

PDB2 1OO9:B

INT non-edgetic

AA1 120T;Interacts with:180V,179N

AA1 121T;Interacts with:239L,228H

AA1 122C;Interacts with:179N

AA1 156L;Interacts with:240Y,209G,208T,210T,207T

AA1 157S;Interacts with:209G,207T,208T

AA1 158I;Interacts with:207T

MUT1 I158V;dbSNP:rs1803571;

AA1 173Q;Interacts with:241H,242S,240Y

AA1 176Q;Interacts with:241H,245D,244T

AA1 177G;Interacts with:241H

AA1 179E;Interacts with:241H

AA1 24C;Interacts with:222H,228H,238P,182A,183H,218H,219E,184A

MUT1 C24S;PMID:9774703;

AA1 25T;Interacts with:240Y,218H,181L,238P,215V,182A,239L,219E,180V

MUT1 T25Q;1375658;

MUT1 T25K;1375657;

MUT1 T25V;961993;

MUT1 T25G;1375656;

MUT1 T25E;1375655;

MUT1 T25R;1375659;

AA1 26C;Interacts with:179N,239L,181L,180V,238P,240Y

AA1 27V;Interacts with:240Y,238P,178G,181L,179N,180V

AA1 28P;Interacts with:179N,239L

AA1 29P;Interacts with:179N

AA1 57L;Interacts with:171F,172Y

AA1 58Y;Interacts with:172Y

AA1 87P;Interacts with:172Y

AA1 88A;Interacts with:172Y

AA1 89M;Interacts with:186A,172Y,227F

AA1 90E;Interacts with:222H,228H,229S

AA1 91S;Interacts with:228H,184A,222H,186A,185Y,183H

AA1 92V;Interacts with:185Y,172Y,183H,184A

AA1 94G;Interacts with:228H

AA2 171F;Interacts with:57L

AA2 172Y;Interacts with:58Y,87P,92V,88A,57L,89M

AA2 178G;Interacts with:27V

AA2 179N;Interacts with:26C,29P,28P,27V,122C,120T

AA2 180V;Interacts with:120T,26C,27V,25T

AA2 181L;Interacts with:26C,27V,25T

AA2 182A;Interacts with:24C,25T

AA2 183H;Interacts with:24C,92V,91S

AA2 184A;Interacts with:91S,92V,24C

AA2 185Y;Interacts with:92V,91S

AA2 186A;Interacts with:89M,91S

AA2 207T;Interacts with:157S,158I,156L

AA2 208T;Interacts with:156L,157S

AA2 209G;Interacts with:156L,157S

AA2 210T;Interacts with:156L

MUT2 T210Y;PMID:10871619;

AA2 215V;Interacts with:25T

AA2 218H;Interacts with:25T,24C

AA2 219E;Interacts with:25T,24C

AA2 222H;Interacts with:90E,24C,91S

AA2 227F;Interacts with:89M

AA2 228H;Interacts with:91S,24C,121T,90E,94G

AA2 229S;Interacts with:90E

AA2 238P;Interacts with:27V,24C,25T,26C

AA2 239L;Interacts with:26C,121T,25T,28P

AA2 240Y;Interacts with:27V,25T,26C,156L,173Q

AA2 241H;Interacts with:173Q,179E,177G,176Q

AA2 242S;Interacts with:173Q

AA2 244T;Interacts with:176Q

AA2 245D;Interacts with:176Q

//

MESH1 mesh:D002294

NAME1 Carcinoma, Squamous Cell

CLUSTER1 clust1

MESH2 mesh:D018805

NAME2 Sepsis

CLUSTER2 clust1

AC1 P08572

PFAM1 PF01413

PDB1 1LI1:C

AC2 P02462

PFAM2 PF01413

PDB2 1LI1:E

INT semi-edgetic

AA1 1523Q;Interacts with:1519Y,1627N,1591A,1589A,1590G

AA1 1524E;Interacts with:1480E,1519Y,1481R,1477Q,1590G

AA1 1554V;Interacts with:1533M

AA1 1556Y;Interacts with:1534A,1533M,1623N,1625Y

AA1 1559S;Interacts with:1623N,1617H,1625Y

AA1 1560R;Interacts with:1521Y,1519Y,1517N,1619R,1588S,1615E,1625Y,1627N,1617H

AA1 1561N;Interacts with:1517N,1518D,1519Y,1619R,1617H,1616C,1618G

AA1 1562D;Interacts with:1519Y,1517N

AA1 1563K;Interacts with:1517N,1480E

AA1 1565Y;Interacts with:1516R

AA2 1477Q;Interacts with:1524E

AA2 1480E;Interacts with:1524E,1563K

AA2 1481R;Interacts with:1524E

MUT2 R1481W;1KG:133742;

AA2 1516R;Interacts with:1565Y

AA2 1517N;Interacts with:1563K,1561N,1560R,1562D

AA2 1518D;Interacts with:1561N

AA2 1519Y;Interacts with:1524E,1562D,1560R,1523Q,1561N

MUT2 Y1519C;843325;

AA2 1521Y;Interacts with:1560R

AA2 1533M;Interacts with:1554V,1556Y

AA2 1534A;Interacts with:1556Y

AA2 1588S;Interacts with:1560R

AA2 1589A;Interacts with:1523Q

AA2 1590G;Interacts with:1524E,1523Q

AA2 1591A;Interacts with:1523Q

AA2 1615E;Interacts with:1560R

AA2 1616C;Interacts with:1561N

AA2 1617H;Interacts with:1561N,1559S,1560R

AA2 1618G;Interacts with:1561N

AA2 1619R;Interacts with:1561N,1560R

AA2 1623N;Interacts with:1559S,1556Y

AA2 1625Y;Interacts with:1560R,1559S,1556Y

AA2 1627N;Interacts with:1523Q,1560R

//

MESH1 mesh:D002294

NAME1 Carcinoma, Squamous Cell

CLUSTER1 clust1

MESH2 mesh:D018805

NAME2 Sepsis

CLUSTER2 clust1

AC1 P35625

PFAM1 PF00965

PDB1 3CKI:B

AC2 P78536

PFAM2 PF13688

PDB2 3CKI:B

INT semi-edgetic

AA1 107R;Interacts with:313D

MUT1 R107C;COSMIC:285737;

AA1 109Y;Interacts with:316M

AA1 117L;Interacts with:437P,415H

AA1 118C;Interacts with:345M

AA1 119N;Interacts with:345M

AA1 120F;Interacts with:345M

AA1 24C;Interacts with:405H,437P,351A,350L,415H,349G,406E,409H

AA1 25T;Interacts with:402V,349G,437P,347T,405H,350L,348L,406E

AA1 26C;Interacts with:346G,440V,438I,348L,439A,347T,437P

AA1 27S;Interacts with:345M,348L,440V,389N,390Y,439A,346G

AA1 28P;Interacts with:390Y

AA1 29S;Interacts with:390Y

AA1 30H;Interacts with:390Y

AA1 34A;Interacts with:345M

AA1 55G;Interacts with:369Y

AA1 56P;Interacts with:369Y

AA1 57F;Interacts with:380L,352Y,353V,362G

AA1 85E;Interacts with:315K

AA1 87S;Interacts with:353V

AA1 88E;Interacts with:415H,414E,409H

AA1 89S;Interacts with:353V,352Y,409H,415H,414E,351A,350L

AA1 90L;Interacts with:352Y,350L,351A

AA2 313D;Interacts with:107R

AA2 315K;Interacts with:85E

AA2 316M;Interacts with:109Y

AA2 345M;Interacts with:27S,120F,34A,119N,118C

AA2 346G;Interacts with:26C,27S

AA2 347T;Interacts with:25T,26C

AA2 348L;Interacts with:27S,26C,25T

AA2 349G;Interacts with:25T,24C

AA2 350L;Interacts with:90L,24C,25T,89S

AA2 351A;Interacts with:24C,90L,89S

AA2 352Y;Interacts with:90L,89S,57F

AA2 353V;Interacts with:89S,57F,87S

AA2 362G;Interacts with:57F

AA2 369Y;Interacts with:55G,56P

AA2 380L;Interacts with:57F

AA2 389N;Interacts with:27S

AA2 390Y;Interacts with:27S,29S,28P,30H

AA2 402V;Interacts with:25T

AA2 405H;Interacts with:24C,25T

AA2 406E;Interacts with:24C,25T

MUT2 E406*;COSMIC:1181783;

AA2 409H;Interacts with:89S,88E,24C

AA2 414E;Interacts with:88E,89S

AA2 415H;Interacts with:88E,117L,24C,89S

AA2 437P;Interacts with:117L,24C,25T,26C

AA2 438I;Interacts with:26C

AA2 439A;Interacts with:27S,26C

AA2 440V;Interacts with:26C,27S

//

MESH1 mesh:D002311

NAME1 Cardiomyopathy, Dilated

CLUSTER1 clust10

MESH2 mesh:D002312

NAME2 Hypertrophic Cardiomyopathy

CLUSTER2 clust10

AC1 P19429

PFAM1 PF00992

PDB1 1J1D:C

AC2 P63316

PFAM2 PF13499

PDB2 1J1D:C

INT non-edgetic

AA1 133F;Interacts with:136L,128I,120M,124T

AA1 134D;Interacts with:124T

AA1 135L;Interacts with:156F,136L,120M

AA1 136R;Interacts with:151D

AA1 139F;Interacts with:100L,157M

AA1 140K;Interacts with:96E

AA1 141R;Interacts with:96E

MUT1 R141Q;963663;humsavar:VAR_019872, phenotype=Cardiomyopathy, familial hypertrophic 7 (CMH7);clinvar:52551;

AA1 142P;Interacts with:96E

AA1 152D;Interacts with:86K

AA1 46K;Interacts with:136L,135E,139D

AA1 48Q;Interacts with:126E

AA1 49L;Interacts with:132D,121L,136L,128I,124T

AA1 50K;Interacts with:157M,156F,139D,155E

AA1 52L;Interacts with:124T

AA1 53L;Interacts with:121L,124T,156F,120M,157M,104F,136L

AA1 54L;Interacts with:97L,92K,157M,100L,104F

AA1 55Q;Interacts with:92K

AA1 56I;Interacts with:123A,120M,124T

AA1 57A;Interacts with:100L,104F,103M,120M

AA1 58K;Interacts with:96E,100L

AA1 60E;Interacts with:103M,106K,104F

AA1 61L;Interacts with:100L,99D,103M

AA1 64E;Interacts with:102R

AA2 100L;Interacts with:61L,57A,54L,58K,139F

AA2 102R;Interacts with:64E

MUT2 R102H;1KG:1338443;

MUT2 R102L;dbSNP:rs143020831;

AA2 103M;Interacts with:60E,61L,57A

AA2 104F;Interacts with:57A,53L,54L,60E

AA2 106K;Interacts with:60E

AA2 120M;Interacts with:53L,56I,57A,133F,135L

AA2 121L;Interacts with:53L,49L

AA2 123A;Interacts with:56I

AA2 124T;Interacts with:53L,56I,52L,49L,133F,134D

AA2 126E;Interacts with:48Q

AA2 128I;Interacts with:49L,133F

AA2 132D;Interacts with:49L

AA2 135E;Interacts with:46K

AA2 136L;Interacts with:46K,49L,53L,133F,135L

AA2 139D;Interacts with:46K,50K

AA2 151D;Interacts with:136R

AA2 155E;Interacts with:50K

AA2 156F;Interacts with:50K,53L,135L

AA2 157M;Interacts with:50K,53L,54L,139F

AA2 86K;Interacts with:152D

AA2 92K;Interacts with:54L,55Q

AA2 96E;Interacts with:58K,140K,141R,142P

AA2 97L;Interacts with:54L

AA2 99D;Interacts with:61L

//

MESH1 mesh:D002311

NAME1 Cardiomyopathy, Dilated

CLUSTER1 clust10

MESH2 mesh:D002312

NAME2 Hypertrophic Cardiomyopathy

CLUSTER2 clust10

AC1 P19429

PFAM1 PF00992

PDB1 1J1D:C

AC2 P63316

PFAM2 PF13833

PDB2 1J1D:C

INT non-edgetic

AA1 147V;Interacts with:56E,52P,60M

AA1 148R;Interacts with:63E,50Q,83R,49G,48L

AA1 149I;Interacts with:45M,80M,60M,84C,41L

AA1 150S;Interacts with:84C

AA1 151A;Interacts with:84C,81M

AA1 152D;Interacts with:84C

AA1 153A;Interacts with:48L

AA1 154M;Interacts with:45M,44V,48L,80M,41L

MUT1 M154I;clinvar:52555;

AA1 155M;Interacts with:81M

MUT1 M155T;clinvar:52557, phenotype=611880 CARDIOMYOPATHY, DILATED, 2A; CMD2A;

AA1 157A;Interacts with:44V,48L

MUT1 A157V;963665;humsavar:VAR_019873, phenotype=Cardiomyopathy, familial hypertrophic 7 (CMH7);clinvar:52558;

AA1 158L;Interacts with:44V,77F,47M

AA2 41L;Interacts with:154M,149I

AA2 44V;Interacts with:158L,154M,157A

AA2 45M;Interacts with:149I,154M

AA2 47M;Interacts with:158L

AA2 48L;Interacts with:157A,153A,154M,148R

AA2 49G;Interacts with:148R

AA2 50Q;Interacts with:148R

AA2 52P;Interacts with:147V

AA2 56E;Interacts with:147V

AA2 60M;Interacts with:147V,149I

AA2 63E;Interacts with:148R

AA2 77F;Interacts with:158L

AA2 80M;Interacts with:149I,154M

AA2 81M;Interacts with:151A,155M

AA2 83R;Interacts with:148R

AA2 84C;Interacts with:152D,151A,150S,149I

MUT2 C84Y;963647;humsavar:VAR_063071, phenotype=Cardiomyopathy, familial hypertrophic 13 (CMH13);

//

MESH1 mesh:D002311

NAME1 Cardiomyopathy, Dilated

CLUSTER1 clust10

MESH2 mesh:D002312

NAME2 Hypertrophic Cardiomyopathy

CLUSTER2 clust10

AC1 O15273

PFAM1 PF09470

PDB1 1YA5:T

AC2 Q8WZ42

PFAM2 PF07679

PDB2 1YA5:T

INT non-edgetic

AA1 10V;Interacts with:90T,92T,91S,11P,8F

AA1 11S;Interacts with:90T,89A,91S

MUT1 S11L;dbSNP:rs45495192;

AA1 12E;Interacts with:89A,90T,8F,88Q,7T,109R

AA1 13E;Interacts with:89A,87G,88Q

AA1 14N;Interacts with:87G,88Q,86S

AA1 15C;Interacts with:87G,86S

AA1 16E;Interacts with:86S

AA1 17R;Interacts with:86S,190E,112S,111Q

AA1 18R;Interacts with:112S,189A,190E

MUT1 R18Q;dbSNP:rs45614536;

AA1 19E;Interacts with:189A,188T,111Q,112S

AA1 20A;Interacts with:188T,187S,189A,109R

AA1 21F;Interacts with:185A,187S,188T,186T,106F

AA1 22W;Interacts with:188T,186T,187S,185A

AA1 23A;Interacts with:185A,184R

AA1 24E;Interacts with:184R,185A,183G

AA1 25W;Interacts with:183G,184R,182V

AA1 26K;Interacts with:181S,182V

AA1 27D;Interacts with:14S,13Q

AA1 28L;Interacts with:15V,14S,16V

AA1 30L;Interacts with:96L,16V

AA1 33R;Interacts with:94E

MUT1 R33W;dbSNP:rs145524909;

AA1 34P;Interacts with:94E

AA1 35E;Interacts with:183G,182V,184R

AA1 36E;Interacts with:77R

AA1 37G;Interacts with:93A,94E,77R,92T

AA1 38C;Interacts with:14S,93A,92T,94E

AA1 39S;Interacts with:91S,92T,93A,77R

AA1 40L;Interacts with:90T,92T,91S,8F,11P

AA1 41H;Interacts with:91S,89A,90T

AA1 42E;Interacts with:109R,88Q,7T,8F,90T,89A

AA1 43E;Interacts with:87G,89A,88Q,6P

AA1 44D;Interacts with:87G,86S,88Q

AA1 45T;Interacts with:86S,88Q,87G

AA1 46Q;Interacts with:86S

AA1 47R;Interacts with:112S,111Q,190E,86S

AA1 48H;Interacts with:190E,189A,112S

AA1 49E;Interacts with:112S,188T,111Q,189A

MUT1 E49K;dbSNP:rs45513698;

AA1 50T;Interacts with:188T,187S,189A

AA1 51Y;Interacts with:105N,187S,186T,188T,106F,185A,109R

AA1 52H;Interacts with:186T,185A,187S,184R

AA1 53Q;Interacts with:105N,185A,184R,104P,186T

MUT1 Q53Ter;clinvar:20564, phenotype=611880 CARDIOMYOPATHY, DILATED, 2A; CMD2A;Muscular dystrophy, limb-girdle, type 2G;MUSCULAR DYSTROPHY, LIMB-GIRDLE, TYPE 2G;

AA1 54Q;Interacts with:183G,184R,185A

AA1 55G;Interacts with:182V,184R,183G

AA1 56Q;Interacts with:181S,182V

AA1 57C;Interacts with:14S

AA1 59V;Interacts with:96L,16V

AA1 60L;Interacts with:16V

AA1 61V;Interacts with:16V

AA1 62Q;Interacts with:13Q,15V,14S

AA1 63R;Interacts with:13Q

AA1 64S;Interacts with:13Q

MUT1 S64L;dbSNP:rs45458802;

AA1 65P;Interacts with:105N

AA1 66W;Interacts with:11P,13Q,10Q,105N,8F,9T

AA1 67L;Interacts with:130I,128T

AA1 68M;Interacts with:13Q

AA1 6L;Interacts with:96L,16V,15V,94E,14S

MUT1 L6M;1KG:1183237;clinvar:53871;

AA1 70R;Interacts with:13Q

MUT1 R70W;960144;humsavar:VAR_026650, phenotype=Cardiomyopathy, dilated 1N (CMD1N);

AA1 71M;Interacts with:17V,18L

MUT1 M71T;dbSNP:rs143465226;

AA1 72G;Interacts with:16V

AA1 73I;Interacts with:14S,16V,15V,96L

AA1 74L;Interacts with:16V,18L,17V

MUT1 L74H;218581;humsavar:VAR_029445;rs17851031;

AA1 75G;Interacts with:18L

AA1 76R;Interacts with:10Q,14S,13Q

AA1 7S;Interacts with:14S,94E,93A,77R

AA1 81Y;Interacts with:17V,18L

AA1 83L;Interacts with:18L,130I

AA1 84P;Interacts with:130I

AA1 85Y;Interacts with:22T,20G,21S,18L

AA1 87R;Interacts with:19E,20G,22T,21S,68P

MUT1 R87Q;218589;humsavar:VAR_015397, phenotype=Cardiomyopathy, dilated 1N (CMD1N);

AA1 8C;Interacts with:92T,93A,94E,13Q,14S

AA1 9E;Interacts with:93A,77R,92T,91S

AA2 104P;Interacts with:53Q

AA2 105N;Interacts with:53Q,51Y,65P,66W

AA2 106F;Interacts with:51Y,21F

AA2 109R;Interacts with:42E,51Y,12E,20A

MUT2 R109*;COSMIC:287595;

AA2 10Q;Interacts with:66W,76R

AA2 111Q;Interacts with:49E,47R,19E,17R

AA2 112S;Interacts with:47R,49E,48H,18R,17R,19E

AA2 11P;Interacts with:66W,10V,40L

AA2 128T;Interacts with:67L

AA2 130I;Interacts with:67L,83L,84P

AA2 13Q;Interacts with:62Q,8C,63R,66W,27D,64S,76R,68M,70R

AA2 14S;Interacts with:7S,28L,27D,62Q,8C,73I,76R,38C,6L,57C

AA2 15V;Interacts with:28L,62Q,6L,73I

AA2 16V;Interacts with:6L,74L,60L,30L,28L,72G,73I,61V,59V

AA2 17V;Interacts with:81Y,71M,74L

AA2 181S;Interacts with:56Q,26K

MUT2 S181N;dbSNP:rs72647843;dbSNP:rs72647843;dbSNP:rs72647843;dbSNP:rs72647843;dbSNP:rs72647843;

AA2 182V;Interacts with:55G,35E,56Q,25W,26K

AA2 183G;Interacts with:35E,55G,54Q,25W,24E

AA2 184R;Interacts with:55G,53Q,54Q,52H,35E,25W,24E,23A

AA2 185A;Interacts with:53Q,52H,51Y,54Q,21F,24E,23A,22W

AA2 186T;Interacts with:52H,51Y,22W,21F,53Q

AA2 187S;Interacts with:50T,51Y,52H,20A,21F,22W

AA2 188T;Interacts with:50T,49E,51Y,20A,19E,22W,21F

AA2 189A;Interacts with:48H,50T,49E,19E,18R,20A

AA2 18L;Interacts with:75G,74L,81Y,85Y,83L,71M

MUT2 L18R;dbSNP:rs140459891;dbSNP:rs140459891;dbSNP:rs140459891;dbSNP:rs140459891;dbSNP:rs140459891;

AA2 190E;Interacts with:48H,47R,17R,18R

AA2 19E;Interacts with:87R

AA2 20G;Interacts with:85Y,87R

AA2 21S;Interacts with:85Y,87R

AA2 22T;Interacts with:85Y,87R

AA2 68P;Interacts with:87R

AA2 6P;Interacts with:43E

AA2 77R;Interacts with:9E,37G,36E,7S,39S

AA2 7T;Interacts with:12E,42E

AA2 86S;Interacts with:17R,15C,14N,16E,45T,47R,44D,46Q

AA2 87G;Interacts with:15C,14N,13E,44D,43E,45T

AA2 88Q;Interacts with:14N,13E,12E,42E,45T,44D,43E

AA2 89A;Interacts with:12E,13E,11S,43E,41H,42E

AA2 8F;Interacts with:12E,10V,42E,40L,66W

AA2 90T;Interacts with:11S,12E,10V,40L,42E,41H

AA2 91S;Interacts with:9E,11S,10V,39S,40L,41H

AA2 92T;Interacts with:8C,9E,10V,39S,40L,38C,37G

AA2 93A;Interacts with:8C,9E,7S,37G,38C,39S

AA2 94E;Interacts with:7S,8C,6L,37G,33R,34P,38C

AA2 96L;Interacts with:6L,30L,59V,73I

AA2 9T;Interacts with:66W

MUT2 T9M;dbSNP:rs146123323;dbSNP:rs146123323;dbSNP:rs146123323;dbSNP:rs146123323;dbSNP:rs146123323;

//

MESH1 mesh:D002311

NAME1 Cardiomyopathy, Dilated

CLUSTER1 clust10

MESH2 mesh:D002312

NAME2 Hypertrophic Cardiomyopathy

CLUSTER2 clust10

AC1 O75147

PFAM1 PF07679

PDB1 2WP3:O

AC2 Q8WZ42

PFAM2 PF07679

PDB2 2WP3:T

INT non-edgetic

AA1 14F;Interacts with:34274T,34276A

AA1 17F;Interacts with:34312L,34276A,34278A,34314T

AA1 19R;Interacts with:34311D,34259E

AA1 20P;Interacts with:34259E

AA1 81V;Interacts with:34260A,34261L

AA1 83V;Interacts with:34262P

AA1 85R;Interacts with:34266S

AA1 87R;Interacts with:34268D

AA1 88N;Interacts with:34271K

AA1 89A;Interacts with:34271K

AA1 90A;Interacts with:34272V,34271K

AA1 91G;Interacts with:34267I,34271K,34272V

AA1 92E;Interacts with:34266S,34267I,34268D,34272V,34273L,34274T

AA1 93A;Interacts with:34272V,34274T

AA1 94Y;Interacts with:34273L,34265I,34266S,34274T,34276A,34275V,34267I

AA1 95A;Interacts with:34276A

AA1 96A;Interacts with:34262P,34276A,34261L,34260A

AA1 97A;Interacts with:34260A

AA1 98A;Interacts with:34260A

AA2 34259E;Interacts with:20P,19R

AA2 34260A;Interacts with:98A,81V,97A,96A

AA2 34261L;Interacts with:96A,81V

AA2 34262P;Interacts with:96A,83V

AA2 34265I;Interacts with:94Y

AA2 34266S;Interacts with:92E,85R,94Y

AA2 34267I;Interacts with:91G,92E,94Y

AA2 34268D;Interacts with:92E,87R

AA2 34271K;Interacts with:91G,89A,90A,88N

AA2 34272V;Interacts with:93A,91G,92E,90A

AA2 34273L;Interacts with:94Y,92E

AA2 34274T;Interacts with:14F,94Y,92E,93A

AA2 34275V;Interacts with:94Y

AA2 34276A;Interacts with:17F,96A,14F,94Y,95A

AA2 34278A;Interacts with:17F

AA2 34311D;Interacts with:19R

AA2 34312L;Interacts with:17F

AA2 34314T;Interacts with:17F

//

MESH1 mesh:D002311

NAME1 Cardiomyopathy, Dilated

CLUSTER1 clust10

MESH2 mesh:D002312

NAME2 Hypertrophic Cardiomyopathy

CLUSTER2 clust10

AC1 P19429

PFAM1 PF00992

PDB1 1J1D:C

AC2 P45379

PFAM2 PF00992

PDB2 1J1D:C

INT non-edgetic

AA1 100L;Interacts with:246L,243A,247W

AA1 101H;Interacts with:231I,223A,230A,246L,227K,226R

AA1 104V;Interacts with:246L

AA1 105D;Interacts with:226R

AA1 79R;Interacts with:247W

MUT1 R79L;clinvar:52538;

MUT1 R79C;203391;humsavar:VAR_029453;rs3729712;clinvar:52537;

AA1 80C;Interacts with:247W

AA1 81Q;Interacts with:247W

AA1 82P;Interacts with:247W

MUT1 P82T;dbSNP:rs77615401;

MUT1 P82S;203392;humsavar:VAR_016078;rs77615401, phenotype=Cardiomyopathy, familial hypertrophic 7 (CMH7);clinvar:27460, phenotype=Cardiomyopathy, familial hypertrophic, 7;CARDIOMYOPATHY, FAMILIAL HYPERTROPHIC, 7;

AA1 83L;Interacts with:244K,247W,243A

AA1 85L;Interacts with:240R,243A,239L

AA1 86A;Interacts with:240R

AA1 88L;Interacts with:240R

AA1 89G;Interacts with:236E

AA1 90F;Interacts with:235N,236E,239L,234L

AA1 93L;Interacts with:239L,240R,236E,243A

AA1 94Q;Interacts with:231I,239L,232D,234L

AA1 96L;Interacts with:243A

AA1 97C;Interacts with:246L,231I,239L,243A

AA1 98R;Interacts with:224E,231I,227K,232D

AA1 99Q;Interacts with:227K

AA2 223A;Interacts with:101H

AA2 224E;Interacts with:98R

AA2 226R;Interacts with:101H,105D

AA2 227K;Interacts with:98R,99Q,101H

MUT2 K227E;clinvar:52830;

AA2 230A;Interacts with:101H

AA2 231I;Interacts with:98R,94Q,101H,97C

MUT2 I231T;963697;dbSNP:rs45520032;humsavar:VAR_057310;rs45520032;

AA2 232D;Interacts with:98R,94Q

AA2 234L;Interacts with:94Q,90F

AA2 235N;Interacts with:90F

AA2 236E;Interacts with:89G,90F,93L

AA2 239L;Interacts with:93L,90F,97C,94Q,85L

AA2 240R;Interacts with:85L,86A,93L,88L

AA2 243A;Interacts with:100L,85L,96L,93L,97C,83L

AA2 244K;Interacts with:83L

AA2 246L;Interacts with:100L,97C,104V,101H

AA2 247W;Interacts with:82P,80C,81Q,83L,100L,79R

//

MESH1 mesh:D002318

NAME1 Cardiovascular Diseases

CLUSTER1 clust3

MESH2 mesh:D020246

NAME2 Venous Thrombosis

CLUSTER2 clust3

AC1 P49257

PFAM1 PF03388

PDB1 3A4U:A

AC2 Q8NI22

PFAM2 PF13499

PDB2 3A4U:A

INT semi-edgetic

AA1 265F;Interacts with:135Y

AA1 44R;Interacts with:133D

AA1 45R;Interacts with:132N,133D,129D

AA1 46F;Interacts with:133D,135Y,91L,134G,89D

AA1 48Y;Interacts with:119N,122D,91L,118I,125L,121I,90G

AA1 49K;Interacts with:122D

AA1 51S;Interacts with:91L

AA1 52F;Interacts with:91L

AA1 53K;Interacts with:92E,89D,83D

AA1 56H;Interacts with:82Y,95T,99H

AA1 59Q;Interacts with:114E,98T,112M

AA1 65P;Interacts with:114E

MUT1 P65L;dbSNP:rs140538038;

AA1 66F;Interacts with:118I,117L,91L,114E,121I

MUT1 F66L;dbSNP:rs142737804;

AA1 96K;Interacts with:114E

AA2 112M;Interacts with:59Q

AA2 114E;Interacts with:59Q,96K,65P,66F

MUT2 E114D;dbSNP:rs141584198;dbSNP:rs141584198;

AA2 117L;Interacts with:66F

AA2 118I;Interacts with:66F,48Y

AA2 119N;Interacts with:48Y

AA2 121I;Interacts with:48Y,66F

AA2 122D;Interacts with:48Y,49K

MUT2 D122Y;COSMIC:257388;

AA2 125L;Interacts with:48Y

AA2 129D;Interacts with:45R

MUT2 D129E;898677;dbSNP:rs137852913;humsavar:VAR_019076;rs28942113, phenotype=Factor V and factor VIII combined deficiency 2 (F5F8D2);clinvar:17909, phenotype=Factor V and factor VIII, combined deficiency of;FACTOR V AND FACTOR VIII, COMBINED DEFICIENCY OF, 2;

AA2 132N;Interacts with:45R

MUT2 N132D;1KG:1176694;1KG:1251405;

AA2 133D;Interacts with:46F,45R,44R

AA2 134G;Interacts with:46F

AA2 135Y;Interacts with:265F,46F

AA2 82Y;Interacts with:56H

AA2 83D;Interacts with:53K

AA2 89D;Interacts with:53K,46F

AA2 90G;Interacts with:48Y

AA2 91L;Interacts with:51S,48Y,66F,46F,52F

AA2 92E;Interacts with:53K

AA2 95T;Interacts with:56H

AA2 98T;Interacts with:59Q

AA2 99H;Interacts with:56H

//

MESH1 mesh:D002446

NAME1 Celiac Disease

CLUSTER1 clust7

MESH2 mesh:D009103

NAME2 Multiple Sclerosis

CLUSTER2 clust7

AC1 P28068

PFAM1 PF07654

PDB1 4I0P:B

AC2 Q6ICR9

PFAM2 PF00993

PDB2 4I0P:A

INT non-edgetic

AA1 141Y;Interacts with:63Y

AA1 168P;Interacts with:65E

AA1 169N;Interacts with:66D,81R

AA1 170G;Interacts with:66D,81R

AA1 171D;Interacts with:66D,82L

AA1 172W;Interacts with:66D,68L,63Y,85F,67Q,82L

AA1 173T;Interacts with:66D

AA1 174Y;Interacts with:65E,66D

AA2 63Y;Interacts with:172W,141Y

AA2 65E;Interacts with:174Y,168P

AA2 66D;Interacts with:172W,173T,174Y,169N,170G,171D

AA2 67Q;Interacts with:172W

AA2 68L;Interacts with:172W

AA2 81R;Interacts with:170G,169N

AA2 82L;Interacts with:171D,172W

AA2 85F;Interacts with:172W

//

MESH1 mesh:D002446

NAME1 Celiac Disease

CLUSTER1 clust7

MESH2 mesh:D009103

NAME2 Multiple Sclerosis

CLUSTER2 clust7

AC1 P04440

PFAM1 PF07654

PDB1 3LQZ:B

AC2 P20036

PFAM2 PF00993

PDB2 3LQZ:A

INT non-edgetic

AA1 176R;Interacts with:59E,58D,60D

AA1 178G;Interacts with:60D,75H

AA1 179D;Interacts with:60D,75H

AA1 180W;Interacts with:62M,60D,61E,75H,57F,79F,76L

AA1 181T;Interacts with:60D

AA2 57F;Interacts with:180W

AA2 58D;Interacts with:176R

AA2 59E;Interacts with:176R

MUT2 E59D;157591;humsavar:VAR_058835;rs2308910;

AA2 60D;Interacts with:180W,179D,178G,176R,181T

AA2 61E;Interacts with:180W

AA2 62M;Interacts with:180W

MUT2 M62L;157592;humsavar:VAR_047685;rs2308911;

MUT2 M62K;157593;humsavar:VAR_058836;rs2308912;

MUT2 M62Q;157599;humsavar:VAR_058850;rs36013091;

AA2 75H;Interacts with:180W,179D,178G

AA2 76L;Interacts with:180W

AA2 79F;Interacts with:180W

//

MESH1 mesh:D002446

NAME1 Celiac Disease

CLUSTER1 clust7

MESH2 mesh:D009103

NAME2 Multiple Sclerosis

CLUSTER2 clust7

AC1 P01909

PFAM1 PF07654

PDB1 1JK8:A

AC2 P01920

PFAM2 PF07654

PDB2 1JK8:B

INT non-edgetic

AA1 118S;Interacts with:188Q

AA1 119K;Interacts with:184D,186T,153D,188Q,182N,152T

AA1 120S;Interacts with:152T,188Q,153D

MUT1 S120F;dbSNP:rs1048122;

AA1 121P;Interacts with:150S,152T,132T,188Q

AA1 131I;Interacts with:182N

AA1 160T;Interacts with:183G

MUT1 T160I;856079;dbSNP:rs41545514;humsavar:VAR_060522;

AA1 173I;Interacts with:181R,182N,183G

AA1 175Y;Interacts with:183G,182N,184D

MUT1 Y175H;856082;dbSNP:rs41550317;humsavar:VAR_060525;

AA2 132T;Interacts with:121P

AA2 150S;Interacts with:121P

AA2 152T;Interacts with:120S,119K,121P

MUT2 T152S;dbSNP:rs9274004;

AA2 153D;Interacts with:119K,120S

MUT2 D153E;dbSNP:rs9274002;

AA2 181R;Interacts with:173I

MUT2 R181G;dbSNP:rs9273967;

MUT2 R181S;dbSNP:rs9273966;

AA2 182N;Interacts with:131I,175Y,119K,173I

MUT2 N182D;dbSNP:rs9273965;

AA2 183G;Interacts with:175Y,160T,173I

MUT2 G183A;dbSNP:rs9273964;

AA2 184D;Interacts with:119K,175Y

MUT2 D184E;dbSNP:rs9273963;

AA2 186T;Interacts with:119K

AA2 188Q;Interacts with:119K,118S,120S,121P

//

MESH1 mesh:D002446

NAME1 Celiac Disease

CLUSTER1 clust7

MESH2 mesh:D009103

NAME2 Multiple Sclerosis

CLUSTER2 clust7

AC1 P01909

PFAM1 PF07654

PDB1 1JK8:A

AC2 P01920

PFAM2 PF00969

PDB2 1JK8:B

INT non-edgetic

AA1 136N;Interacts with:66R

AA1 138F;Interacts with:66R,65N

AA1 149N;Interacts with:105A,112R,108D,107L

AA1 150G;Interacts with:104R

AA1 151H;Interacts with:105A,107L,108D,104R

MUT1 H151Q;390300;humsavar:VAR_050386;rs707950;

AA1 152S;Interacts with:101E

MUT1 S152A;390294;dbSNP:rs34078100,dbSNP:rs41547417,dbSNP:rs74646061;humsavar:VAR_060521;

AA1 167D;Interacts with:66R

AA1 168H;Interacts with:68E,63I,66R,67E

AA1 169S;Interacts with:66R

AA1 182A;Interacts with:55R,112R,54E

MUT1 A182D;856083;dbSNP:rs7990,dbSNP:rs79809169;humsavar:VAR_060527;

MUT1 A182S;856084;dbSNP:rs41561312;humsavar:VAR_060528;

AA1 183E;Interacts with:112R,53T,116Q,54E

MUT1 E183D;390299;humsavar:VAR_060529;rs707963;

AA1 189K;Interacts with:101E

AA2 101E;Interacts with:152S,189K

AA2 104R;Interacts with:150G,151H

AA2 105A;Interacts with:151H,149N

AA2 107L;Interacts with:151H,149N

MUT2 L107V;856149;dbSNP:rs9274384;humsavar:VAR_062729;rs9274384;

AA2 108D;Interacts with:151H,149N

AA2 112R;Interacts with:183E,149N,182A

AA2 116Q;Interacts with:183E

MUT2 Q116E;856151;humsavar:VAR_062731;rs1140316;

AA2 53T;Interacts with:183E

AA2 54E;Interacts with:182A,183E

AA2 55R;Interacts with:182A

MUT2 R55L;856112;dbSNP:rs41540813;humsavar:VAR_062692;rs41540813;

AA2 63I;Interacts with:168H

AA2 65N;Interacts with:138F

AA2 66R;Interacts with:167D,169S,136N,138F,168H

MUT2 R66P;dbSNP:rs75769070;

MUT2 R66Q;dbSNP:rs75769070;

AA2 67E;Interacts with:168H

AA2 68E;Interacts with:168H

//

MESH1 mesh:D002446

NAME1 Celiac Disease

CLUSTER1 clust7

MESH2 mesh:D009103

NAME2 Multiple Sclerosis

CLUSTER2 clust7

AC1 P01909

PFAM1 PF00993

PDB1 1UVQ:A

AC2 P01920

PFAM2 PF00969

PDB2 1UVQ:B

INT non-edgetic

AA1 101R;Interacts with:88P,89D,85L

MUT1 R101C;856076;dbSNP:rs41542116;humsavar:VAR_060519;

AA1 102S;Interacts with:69Y,64Y,85L

MUT1 S102Y;856077;humsavar:VAR_050384;rs1129808;

AA1 104S;Interacts with:64Y

AA1 105T;Interacts with:65N,64Y

AA1 106A;Interacts with:65N,64Y

MUT1 A106T;dbSNP:rs142591955;

AA1 107A;Interacts with:65N

MUT1 A107P;856092;

AA1 29D;Interacts with:52G,49F,50T,51N

AA1 30H;Interacts with:49F,115Y,50T,48Y

AA1 31V;Interacts with:46M,49F,48Y,47C

AA1 32A;Interacts with:48Y,47C,49F,46M

AA1 33S;Interacts with:47C,46M,45A

AA1 34Y;Interacts with:45A,110V

MUT1 Y34C;390303;humsavar:VAR_060494;rs1129740;

AA1 35G;Interacts with:45A

AA1 50H;Interacts with:114N

AA1 52F;Interacts with:49F

AA1 77F;Interacts with:118E,117L

MUT1 F77I;dbSNP:rs3188043;

MUT1 F77L;856064;dbSNP:rs3188043;humsavar:VAR_060509;rs3188043;

AA1 78R;Interacts with:117L

MUT1 R78S;856065;humsavar:VAR_033407;rs36219345;

AA1 94N;Interacts with:69Y

AA1 95L;Interacts with:64Y

AA1 98L;Interacts with:85L,64Y,69Y

MUT1 L98V;856075;dbSNP:rs1064944;humsavar:VAR_060518;

MUT1 L98M;856074;humsavar:VAR_060517;

AA1 99I;Interacts with:64Y

AA2 110V;Interacts with:34Y

AA2 114N;Interacts with:50H

AA2 115Y;Interacts with:30H

AA2 117L;Interacts with:78R,77F

MUT2 L117V;856152;humsavar:VAR_062732;rs1140317;

AA2 118E;Interacts with:77F

MUT2 E118G;856154;dbSNP:rs9274380;humsavar:VAR_062734;

MUT2 E118A;856153;humsavar:VAR_062733;

AA2 45A;Interacts with:34Y,33S,35G

MUT2 A45G;856110;humsavar:VAR_061472;rs1130375;

AA2 46M;Interacts with:31V,33S,32A

MUT2 M46L;856111;dbSNP:rs1130368;humsavar:VAR_061473;rs1130368;

MUT2 M46R;dbSNP:rs41556424;

MUT2 M46T;dbSNP:rs41556424;

AA2 47C;Interacts with:32A,33S,31V

AA2 48Y;Interacts with:32A,31V,30H

AA2 49F;Interacts with:30H,29D,31V,52F,32A

AA2 50T;Interacts with:30H,29D

AA2 51N;Interacts with:29D

AA2 52G;Interacts with:29D

MUT2 G52R;dbSNP:rs188170056;

AA2 64Y;Interacts with:104S,98L,106A,105T,102S,95L,99I

AA2 65N;Interacts with:105T,106A,107A

AA2 69Y;Interacts with:94N,102S,98L

MUT2 Y69N;dbSNP:rs1130370;

MUT2 Y69D;856119;dbSNP:rs1130370;humsavar:VAR_062699;rs1130370;

MUT2 Y69F;dbSNP:rs9274400;

MUT2 Y69I;856120;humsavar:VAR_062700;

AA2 85L;Interacts with:98L,102S,101R

MUT2 L85Q;856128;humsavar:VAR_062708;rs1140313;

AA2 88P;Interacts with:101R

MUT2 P88L;856132;dbSNP:rs1130381;humsavar:VAR_062712;rs1130381;

AA2 89D;Interacts with:101R

MUT2 D89N;dbSNP:rs41552812;

MUT2 D89A;856133;dbSNP:rs1071637;humsavar:VAR_062713;rs1071637;

MUT2 D89G;dbSNP:rs1071637;

MUT2 D89V;856135;dbSNP:rs1071637;humsavar:VAR_062715;

MUT2 D89S;856134;humsavar:VAR_062714;

//

MESH1 mesh:D002446

NAME1 Celiac Disease

CLUSTER1 clust7

MESH2 mesh:D009103

NAME2 Multiple Sclerosis

CLUSTER2 clust7

AC1 P01589

PFAM1 PF00084

PDB1 1Z92:B

AC2 P60568

PFAM2 PF00715

PDB2 1Z92:B

INT non-edgetic

AA1 24C;Interacts with:58R,55K

MUT1 C24A;PMID:2832473;

AA1 25D;Interacts with:54P,55K,58R

MUT1 D25K;PMID:3135551;

MUT1 D25N;1KG:1340011;

AA1 26D;Interacts with:58R

MUT1 D26H;dbSNP:rs55868253;

AA1 27D;Interacts with:58R

AA1 46M;Interacts with:92L

AA1 48N;Interacts with:62F,61T

AA1 50E;Interacts with:63K

AA1 56R;Interacts with:65Y,126E,128A,125C,127Y,82E

AA1 57R;Interacts with:81E,82E,65Y,63K

AA1 59K;Interacts with:81E,127Y,125C

AA1 60S;Interacts with:82E,127Y,81E,85P,84K

AA1 61G;Interacts with:82E,81E,84K,85P

MUT1 G61R;1KG:1340008;

AA1 62S;Interacts with:88E,84K

AA1 63L;Interacts with:63K,88E,85P,62F

AA1 64Y;Interacts with:92L,88E,91N,62F

AA1 78N;Interacts with:88E,84K

AA2 125C;Interacts with:56R,59K

AA2 126E;Interacts with:56R

AA2 127Y;Interacts with:60S,56R,59K

AA2 128A;Interacts with:56R

AA2 54P;Interacts with:25D

MUT2 P54S;1KG:1244597;

AA2 55K;Interacts with:25D,24C

AA2 58R;Interacts with:26D,25D,24C,27D

AA2 61T;Interacts with:48N

AA2 62F;Interacts with:48N,63L,64Y

AA2 63K;Interacts with:63L,50E,57R

AA2 65Y;Interacts with:56R,57R

AA2 81E;Interacts with:57R,59K,60S,61G

AA2 82E;Interacts with:60S,61G,57R,56R

AA2 84K;Interacts with:62S,61G,60S,78N

AA2 85P;Interacts with:63L,60S,61G

AA2 88E;Interacts with:64Y,78N,63L,62S

AA2 91N;Interacts with:64Y

AA2 92L;Interacts with:64Y,46M

//

MESH1 mesh:D002446

NAME1 Celiac Disease

CLUSTER1 clust7

MESH2 mesh:D009103

NAME2 Multiple Sclerosis

CLUSTER2 clust7

AC1 P60568

PFAM1 PF00715

PDB1 2B5I:A

AC2 P31785

PFAM2 PF00041

PDB2 2B5I:C

INT semi-edgetic

AA1 124M;Interacts with:222R,197W,220T

AA1 125C;Interacts with:190R,197W

AA1 126E;Interacts with:197W,222R

AA1 127Y;Interacts with:195H,190R

AA1 146Q;Interacts with:232G,229P,230L,231C,228N

AA1 147S;Interacts with:231C,230L

AA1 149I;Interacts with:230L,181H

AA1 150S;Interacts with:230L,181H,231C

AA1 31Q;Interacts with:181H

AA1 35E;Interacts with:230L,181H

AA1 38L;Interacts with:230L,229P

MUT1 L38R;dbSNP:rs3087209;

AA1 39L;Interacts with:230L

AA1 42Q;Interacts with:232G,229P,230L,228N

AA1 65Y;Interacts with:195H

AA1 81E;Interacts with:190R

AA2 181H;Interacts with:31Q,150S,149I,35E

AA2 190R;Interacts with:125C,127Y,81E

AA2 195H;Interacts with:65Y,127Y

AA2 197W;Interacts with:125C,126E,124M

AA2 220T;Interacts with:124M

AA2 222R;Interacts with:124M,126E

MUT2 R222C;160917;humsavar:VAR_002688, phenotype=X-linked combined immunodeficiency (XCID);

AA2 228N;Interacts with:146Q,42Q

AA2 229P;Interacts with:38L,146Q,42Q

AA2 230L;Interacts with:38L,35E,150S,149I,146Q,147S,39L,42Q

MUT2 L230P;884416;humsavar:VAR_002693, phenotype=Severe combined immunodeficiency X-linked T-cell-negative/B-cell-positive/NK-cell-negative (XSCID);

AA2 231C;Interacts with:147S,146Q,150S

AA2 232G;Interacts with:42Q,146Q

//

MESH1 mesh:D002446

NAME1 Celiac Disease

CLUSTER1 clust7

MESH2 mesh:D009103

NAME2 Multiple Sclerosis

CLUSTER2 clust7

AC1 P60568

PFAM1 PF00715

PDB1 2B5I:A

AC2 P31785

PFAM2 PF09240

PDB2 2B5I:C

INT semi-edgetic

AA1 130E;Interacts with:93N,92K

AA1 139N;Interacts with:147K,149Q,150N

AA1 140R;Interacts with:125Y

AA1 143T;Interacts with:125Y,149Q

AA1 146Q;Interacts with:150N,125Y,149Q

AA1 147S;Interacts with:125Y

AA1 150S;Interacts with:125Y

AA1 68K;Interacts with:93N

AA1 69K;Interacts with:95D

AA2 125Y;Interacts with:143T,146Q,147S,150S,140R

MUT2 Y125N;884404;humsavar:VAR_002679, phenotype=Severe combined immunodeficiency X-linked T-cell-negative/B-cell-positive/NK-cell-negative (XSCID);

AA2 147K;Interacts with:139N

AA2 149Q;Interacts with:143T,146Q,139N

AA2 150N;Interacts with:146Q,139N

AA2 92K;Interacts with:130E

AA2 93N;Interacts with:130E,68K

AA2 95D;Interacts with:69K

//

MESH1 mesh:D002446

NAME1 Celiac Disease

CLUSTER1 clust7

MESH2 mesh:D009103

NAME2 Multiple Sclerosis

CLUSTER2 clust7

AC1 Q30154

PFAM1 PF07654

PDB1 1ZGL:B

AC2 P01903

PFAM2 PF00993

PDB2 1ZGL:A

INT semi-edgetic

AA1 152Y;Interacts with:54D,51F

AA1 178Q;Interacts with:53G,54D,52D

MUT1 Q178H;dbSNP:rs139485758;

AA1 179N;Interacts with:69R,54D

AA1 180G;Interacts with:55E,54D,69R

MUT1 G180E;dbSNP:rs1059353;

AA1 181D;Interacts with:70L,54D,69R

AA1 182W;Interacts with:51F,73F,55E,56I,70L,54D,69R

AA1 183T;Interacts with:54D

AA2 51F;Interacts with:182W,152Y

AA2 52D;Interacts with:178Q

AA2 53G;Interacts with:178Q

AA2 54D;Interacts with:181D,178Q,152Y,180G,183T,182W,179N

MUT2 D54T;856207;

AA2 55E;Interacts with:180G,182W

AA2 56I;Interacts with:182W

MUT2 I56E;PMID:7528190,PMID:7869051,PMID:7636246;

AA2 69R;Interacts with:179N,181D,180G,182W

MUT2 R69L;856211;

AA2 70L;Interacts with:181D,182W

AA2 73F;Interacts with:182W

//

MESH1 mesh:D002446

NAME1 Celiac Disease

CLUSTER1 clust7

MESH2 mesh:D009103

NAME2 Multiple Sclerosis

CLUSTER2 clust7

AC1 P04233

PFAM1 PF07654

PDB1 4AH2:B

AC2 P01903

PFAM2 PF00993

PDB2 4AH2:A

INT semi-edgetic

AA1 224H;Interacts with:51F

AA1 245G;Interacts with:53G,51F,52D,54D

AA1 246S;Interacts with:69R

AA1 247I;Interacts with:54D,69R

AA1 248G;Interacts with:70L,54D

AA1 249Y;Interacts with:51F,73F,55E,56I,70L,54D

AA1 250C;Interacts with:54D

AA2 51F;Interacts with:249Y,245G,224H

AA2 52D;Interacts with:245G

AA2 53G;Interacts with:245G

AA2 54D;Interacts with:248G,245G,247I,250C,249Y

MUT2 D54T;856207;

AA2 55E;Interacts with:249Y

AA2 56I;Interacts with:249Y

MUT2 I56E;PMID:7528190,PMID:7869051,PMID:7636246;

AA2 69R;Interacts with:246S,247I

MUT2 R69L;856211;

AA2 70L;Interacts with:248G,249Y

AA2 73F;Interacts with:249Y

//

MESH1 mesh:D002446

NAME1 Celiac Disease

CLUSTER1 clust7

MESH2 mesh:D009103

NAME2 Multiple Sclerosis

CLUSTER2 clust7

AC1 P01911

PFAM1 PF07654

PDB1 1BX2:B

AC2 P01903

PFAM2 PF00993

PDB2 1BX2:A

INT semi-edgetic

AA1 178Q;Interacts with:53G,52D,54D,51F

MUT1 Q178H;dbSNP:rs77637983,dbSNP:rs113627837;

AA1 179N;Interacts with:69R

AA1 180G;Interacts with:54D,69R

MUT1 G180E;dbSNP:rs1059353;

AA1 181D;Interacts with:70L,54D,69R

AA1 182W;Interacts with:51F,73F,55E,56I,70L,54D

AA1 183T;Interacts with:54D

AA2 51F;Interacts with:182W,178Q

AA2 52D;Interacts with:178Q

AA2 53G;Interacts with:178Q

AA2 54D;Interacts with:181D,178Q,180G,183T,182W

MUT2 D54T;856207;

AA2 55E;Interacts with:182W

AA2 56I;Interacts with:182W

MUT2 I56E;PMID:7528190,PMID:7869051,PMID:7636246;

AA2 69R;Interacts with:179N,181D,180G

MUT2 R69L;856211;

AA2 70L;Interacts with:181D,182W

AA2 73F;Interacts with:182W

//

MESH1 mesh:D002446

NAME1 Celiac Disease

CLUSTER1 clust7

MESH2 mesh:D009103

NAME2 Multiple Sclerosis

CLUSTER2 clust7

AC1 P13232

PFAM1 PF01415

PDB1 3DI2:A

AC2 P16871

PFAM2 PF00041

PDB2 3DI2:B

INT semi-edgetic

AA1 103H;Interacts with:159Y

AA1 106K;Interacts with:159Y

AA1 35K;Interacts with:212Y

AA1 36Q;Interacts with:212Y

AA1 39S;Interacts with:211H,159Y,212Y,213F

AA1 40V;Interacts with:213F,212Y,159Y

AA1 43V;Interacts with:159Y,158K

AA1 44S;Interacts with:159Y

AA1 47Q;Interacts with:158K

AA2 158K;Interacts with:43V,47Q

AA2 159Y;Interacts with:39S,103H,43V,106K,40V,44S

AA2 211H;Interacts with:39S

AA2 212Y;Interacts with:40V,36Q,39S,35K

AA2 213F;Interacts with:40V,39S

//

MESH1 mesh:D002446

NAME1 Celiac Disease

CLUSTER1 clust7

MESH2 mesh:D009103

NAME2 Multiple Sclerosis

CLUSTER2 clust7

AC1 P16410

PFAM1 PF07686

PDB1 1I85:D

AC2 P42081

PFAM2 PF07686

PDB2 1I85:D

INT semi-edgetic

AA1 132E;Interacts with:122R

AA1 134M;Interacts with:121I,113H,120M,122R

AA1 135Y;Interacts with:113H,69Y,74K,54V,120M

AA1 137P;Interacts with:64V,77S,67E,56F

AA1 138P;Interacts with:64V,58Q,56F,62N

AA1 139Y;Interacts with:58Q,111I,113H,122R,56F

AA1 140Y;Interacts with:62N

AA1 68E;Interacts with:115K,120M

AA1 70R;Interacts with:119G,120M

AA1 88T;Interacts with:118T,121I,120M,119G

AA2 111I;Interacts with:139Y

AA2 113H;Interacts with:135Y,134M,139Y

AA2 115K;Interacts with:68E

AA2 118T;Interacts with:88T

AA2 119G;Interacts with:70R,88T

AA2 120M;Interacts with:88T,134M,68E,70R,135Y

AA2 121I;Interacts with:134M,88T

AA2 122R;Interacts with:132E,134M,139Y

AA2 54V;Interacts with:135Y

AA2 56F;Interacts with:138P,137P,139Y

AA2 58Q;Interacts with:139Y,138P

AA2 62N;Interacts with:140Y,138P

AA2 64V;Interacts with:138P,137P

AA2 67E;Interacts with:137P

AA2 69Y;Interacts with:135Y

AA2 74K;Interacts with:135Y

AA2 77S;Interacts with:137P

//

MESH1 mesh:D002446

NAME1 Celiac Disease

CLUSTER1 clust7

MESH2 mesh:D009103

NAME2 Multiple Sclerosis

CLUSTER2 clust7

AC1 P16410

PFAM1 PF07686

PDB1 1I8L:C

AC2 P33681

PFAM2 PF07686

PDB2 1I8L:C

INT semi-edgetic

AA1 100D;Interacts with:123K

AA1 132E;Interacts with:128R

AA1 134M;Interacts with:126F,127K,119L,63R,65Y,128R

AA1 135Y;Interacts with:65Y,77M

AA1 137P;Interacts with:65Y,72M

AA1 138P;Interacts with:65Y,67Q,70K,72M

AA1 139Y;Interacts with:128R,67Q,70K,117V,65Y,119L,131L

AA1 140Y;Interacts with:70K

AA1 141L;Interacts with:131L

AA1 68E;Interacts with:63R,126F

AA1 70R;Interacts with:124D,125A,126F

AA1 88T;Interacts with:124D,126F,125A

AA2 117V;Interacts with:139Y

AA2 119L;Interacts with:134M,139Y

AA2 123K;Interacts with:100D

AA2 124D;Interacts with:88T,70R

AA2 125A;Interacts with:88T,70R

AA2 126F;Interacts with:134M,88T,70R,68E

AA2 127K;Interacts with:134M

AA2 128R;Interacts with:139Y,132E,134M

AA2 131L;Interacts with:141L,139Y

AA2 63R;Interacts with:68E,134M

AA2 65Y;Interacts with:135Y,138P,139Y,137P,134M

AA2 67Q;Interacts with:139Y,138P

AA2 70K;Interacts with:139Y,138P,140Y

AA2 72M;Interacts with:138P,137P

AA2 77M;Interacts with:135Y

//

MESH1 mesh:D002446

NAME1 Celiac Disease

CLUSTER1 clust7

MESH2 mesh:D009103

NAME2 Multiple Sclerosis

CLUSTER2 clust7

AC1 P01912

PFAM1 PF07654

PDB1 1A6A:B

AC2 P01903

PFAM2 PF00993

PDB2 1A6A:A

INT semi-edgetic

AA1 178H;Interacts with:53G,54D,52D

AA1 179N;Interacts with:69R

AA1 180G;Interacts with:54D,69R

AA1 181D;Interacts with:69R

AA1 182W;Interacts with:51F,73F,55E,56I,70L,54D

AA1 183T;Interacts with:54D

AA2 51F;Interacts with:182W

AA2 52D;Interacts with:178H

AA2 53G;Interacts with:178H

AA2 54D;Interacts with:178H,180G,183T,182W

MUT2 D54T;856207;

AA2 55E;Interacts with:182W

AA2 56I;Interacts with:182W

MUT2 I56E;PMID:7528190,PMID:7869051,PMID:7636246;

AA2 69R;Interacts with:179N,181D,180G

MUT2 R69L;856211;

AA2 70L;Interacts with:182W

AA2 73F;Interacts with:182W

//

MESH1 mesh:D003093

NAME1 Colitis, Ulcerative

CLUSTER1 clust1

MESH2 mesh:D003424

NAME2 Crohn Disease

CLUSTER2 clust1

AC1 P29459

PFAM1 PF03039

PDB1 3HMX:B

AC2 P29460

PFAM2 PF10420

PDB2 3HMX:A

INT non-edgetic

AA1 205R;Interacts with:203E

AA1 208T;Interacts with:203E,202A

AA1 211R;Interacts with:136Y

AA1 71H;Interacts with:205S

AA1 76K;Interacts with:205S

AA1 82V;Interacts with:201A,202A,203E

AA1 83E;Interacts with:201A,202A

AA1 85C;Interacts with:202A

AA1 86L;Interacts with:202A,200P,201A

AA2 136Y;Interacts with:211R

AA2 200P;Interacts with:86L

AA2 201A;Interacts with:82V,83E,86L

AA2 202A;Interacts with:85C,82V,86L,83E,208T

AA2 203E;Interacts with:208T,82V,205R

AA2 205S;Interacts with:71H,76K

//

MESH1 mesh:D003093

NAME1 Colitis, Ulcerative

CLUSTER1 clust1

MESH2 mesh:D003424

NAME2 Crohn Disease

CLUSTER2 clust1

AC1 Q13324

PFAM1 PF02793

PDB1 3N95:C

AC2 Q96RP3

PFAM2 PF11613

PDB2 3N95:E

INT non-edgetic

AA1 47I;Interacts with:105I,106L,109V

AA1 68F;Interacts with:105I,106L,102N

AA1 69N;Interacts with:98Q,102N

AA1 71V;Interacts with:99A,102N,98Q

AA1 73Y;Interacts with:106L,103A,102N

AA1 92K;Interacts with:107A,109V

MUT1 K92N;dbSNP:rs148406223;dbSNP:rs148406223;

AA1 93I;Interacts with:109V

MUT1 I93S;dbSNP:rs144584262;dbSNP:rs144584262;

AA1 94N;Interacts with:109V

AA1 95Y;Interacts with:106L,109V

AA1 96S;Interacts with:106L

AA2 102N;Interacts with:71V,69N,73Y,68F

AA2 103A;Interacts with:73Y

AA2 105I;Interacts with:47I,68F

AA2 106L;Interacts with:96S,47I,73Y,95Y,68F

AA2 107A;Interacts with:92K

AA2 109V;Interacts with:47I,94N,95Y,92K,93I

AA2 98Q;Interacts with:69N,71V

AA2 99A;Interacts with:71V

//

MESH1 mesh:D003093

NAME1 Colitis, Ulcerative

CLUSTER1 clust1

MESH2 mesh:D003424

NAME2 Crohn Disease

CLUSTER2 clust1

AC1 P14778

PFAM1 PF13895

PDB1 1IRA:Y

AC2 P18510

PFAM2 PF00340

PDB2 1IRA:Y

INT non-edgetic

AA1 125Q;Interacts with:63P,62G,61Q

MUT1 Q125K;dbSNP:rs76949245;

AA1 126A;Interacts with:61Q

AA1 127I;Interacts with:61Q,62G

AA1 128F;Interacts with:60L,61Q

AA1 129K;Interacts with:60L

AA1 131K;Interacts with:68E

AA1 132L;Interacts with:172Y

AA1 139G;Interacts with:172Y

AA1 144Y;Interacts with:152A,60L

AA1 146E;Interacts with:152A,151E

AA2 151E;Interacts with:146E

MUT2 E151G;PMID:7744786;

AA2 152A;Interacts with:146E,144Y

AA2 172Y;Interacts with:139G,132L

MUT2 Y172T;PMID:7744786;

MUT2 Y172M;PMID:7744786;

MUT2 Y172G;PMID:7744786;

MUT2 Y172K;PMID:7744786;

MUT2 Y172H;PMID:7744786;

AA2 60L;Interacts with:128F,129K,144Y

AA2 61Q;Interacts with:127I,126A,125Q,128F

MUT2 Q61G;PMID:7744786;

MUT2 Q61F;PMID:7744786;

AA2 62G;Interacts with:125Q,127I

AA2 63P;Interacts with:125Q

MUT2 P63G;PMID:7744786;

AA2 68E;Interacts with:131K

MUT2 E68G;PMID:7744786;

//

MESH1 mesh:D003093

NAME1 Colitis, Ulcerative

CLUSTER1 clust1

MESH2 mesh:D003424

NAME2 Crohn Disease

CLUSTER2 clust1

AC1 Q8IU54

PFAM1 PF15177

PDB1 3OG4:A

AC2 Q8IU57

PFAM2 PF01108

PDB2 3OG4:B

INT non-edgetic

AA1 168S;Interacts with:64T

AA1 171F;Interacts with:63P,64T,94N

AA1 174F;Interacts with:93Y

AA1 175R;Interacts with:93Y,91D

AA1 178T;Interacts with:93Y

AA1 179R;Interacts with:93Y

AA1 54R;Interacts with:93Y,94N

AA1 55D;Interacts with:95K

AA1 58E;Interacts with:95K,66R

AA1 59E;Interacts with:95K

MUT1 E59D;dbSNP:rs142971889;

AA1 66W;Interacts with:64T,63P

AA1 71P;Interacts with:65R

AA1 73F;Interacts with:65R

AA1 74P;Interacts with:65R

AA1 75G;Interacts with:65R,89K

MUT1 G75R;dbSNP:rs185252878;

AA2 63P;Interacts with:171F,66W

AA2 64T;Interacts with:66W,171F,168S

AA2 65R;Interacts with:74P,75G,71P,73F

AA2 66R;Interacts with:58E

AA2 89K;Interacts with:75G

AA2 91D;Interacts with:175R

AA2 93Y;Interacts with:179R,54R,174F,175R,178T

AA2 94N;Interacts with:54R,171F

AA2 95K;Interacts with:59E,58E,55D

//

MESH1 mesh:D003093

NAME1 Colitis, Ulcerative

CLUSTER1 clust1

MESH2 mesh:D003424

NAME2 Crohn Disease

CLUSTER2 clust1

AC1 Q13324

PFAM1 PF02793

PDB1 3N93:B

AC2 Q969E3

PFAM2 PF11613

PDB2 3N93:C

INT non-edgetic

AA1 47I;Interacts with:157I,154M,153L

AA1 68F;Interacts with:154M,153L,150N

AA1 69N;Interacts with:146Q,150N

AA1 70G;Interacts with:146Q,143L

AA1 71V;Interacts with:147A,150N,143L,146Q

AA1 73Y;Interacts with:151A,147A,154M,150N

AA1 92K;Interacts with:157I,156Q,155A

MUT1 K92N;dbSNP:rs148406223;dbSNP:rs148406223;

AA1 93I;Interacts with:157I

MUT1 I93S;dbSNP:rs144584262;dbSNP:rs144584262;

AA1 95Y;Interacts with:154M,157I

AA2 143L;Interacts with:71V,70G

AA2 146Q;Interacts with:70G,69N,71V

AA2 147A;Interacts with:71V,73Y

MUT2 A147V;1KG:1127728;

AA2 150N;Interacts with:71V,69N,73Y,68F

AA2 151A;Interacts with:73Y

AA2 153L;Interacts with:68F,47I

AA2 154M;Interacts with:68F,95Y,73Y,47I

AA2 155A;Interacts with:92K

AA2 156Q;Interacts with:92K

AA2 157I;Interacts with:47I,92K,95Y,93I

//

MESH1 mesh:D003093

NAME1 Colitis, Ulcerative

CLUSTER1 clust1

MESH2 mesh:D003424

NAME2 Crohn Disease

CLUSTER2 clust1

AC1 Q969J5

PFAM1 PF09294

PDB1 3G9V:C

AC2 Q9GZX6

PFAM2 PF14565

PDB2 3G9V:C

INT non-edgetic

AA1 200E;Interacts with:175R

AA1 246P;Interacts with:50P

AA1 247M;Interacts with:54N,50P,51Y,53T,48Q

AA1 248L;Interacts with:54N,53T,50P

AA1 249D;Interacts with:54N,50P

AA1 250R;Interacts with:54N

AA2 175R;Interacts with:200E

AA2 48Q;Interacts with:247M

AA2 50P;Interacts with:247M,249D,248L,246P

AA2 51Y;Interacts with:247M

AA2 53T;Interacts with:247M,248L

AA2 54N;Interacts with:247M,248L,250R,249D

//

MESH1 mesh:D003093

NAME1 Colitis, Ulcerative

CLUSTER1 clust1

MESH2 mesh:D003424

NAME2 Crohn Disease

CLUSTER2 clust1

AC1 Q969J5

PFAM1 PF01108

PDB1 3G9V:A

AC2 Q9GZX6

PFAM2 PF14565

PDB2 3G9V:A

INT non-edgetic

AA1 100G;Interacts with:70T,71D,72V,73R

AA1 101Q;Interacts with:71D,72V,70T

AA1 102R;Interacts with:71D

AA1 103Q;Interacts with:71D

AA1 104W;Interacts with:70T,71D

AA1 128Q;Interacts with:175R,73R,176N

AA1 129E;Interacts with:73R

AA1 130P;Interacts with:73R

AA1 132Y;Interacts with:70T

AA1 65K;Interacts with:70T,71D

AA1 66I;Interacts with:73R

AA1 99Y;Interacts with:70T,169L,72V,69N,73R

AA2 169L;Interacts with:99Y

AA2 175R;Interacts with:128Q

AA2 176N;Interacts with:128Q

AA2 69N;Interacts with:99Y

AA2 70T;Interacts with:104W,99Y,132Y,100G,65K,101Q

AA2 71D;Interacts with:101Q,103Q,100G,104W,65K,102R

AA2 72V;Interacts with:99Y,100G,101Q

AA2 73R;Interacts with:129E,130P,128Q,100G,99Y,66I

MUT2 R73H;dbSNP:rs149366319;

//

MESH1 mesh:D003093

NAME1 Colitis, Ulcerative

CLUSTER1 clust1

MESH2 mesh:D003424

NAME2 Crohn Disease

CLUSTER2 clust1

AC1 Q8N6P7

PFAM1 PF09294

PDB1 3DGC:R

AC2 Q9GZX6

PFAM2 PF14565

PDB2 3DGC:R

INT non-edgetic

AA1 133R;Interacts with:116Q,117E

AA1 134S;Interacts with:124R

AA1 136Q;Interacts with:124R

AA1 162D;Interacts with:175R,48Q,44K

AA1 175Y;Interacts with:51Y

AA1 179L;Interacts with:45S

AA1 180G;Interacts with:48Q

AA1 183Q;Interacts with:45S,43D,44K

AA1 185E;Interacts with:128R,124R,43D

AA1 186Y;Interacts with:128R

AA1 187E;Interacts with:124R,49Q,128R,45S

AA1 189F;Interacts with:46N,52I,49Q,121F,124R

AA1 190G;Interacts with:51Y,52I,49Q

AA1 191L;Interacts with:117E

AA1 206P;Interacts with:48Q

AA1 207T;Interacts with:172M,175R,48Q,44K

AA1 208W;Interacts with:57F,172M

AA2 116Q;Interacts with:133R

AA2 117E;Interacts with:191L,133R

AA2 121F;Interacts with:189F

AA2 124R;Interacts with:187E,185E,189F,134S,136Q

AA2 128R;Interacts with:187E,185E,186Y

AA2 172M;Interacts with:207T,208W

MUT2 M172V;dbSNP:rs147812317;

AA2 175R;Interacts with:162D,207T

AA2 43D;Interacts with:183Q,185E

AA2 44K;Interacts with:162D,207T,183Q

AA2 45S;Interacts with:179L,183Q,187E

AA2 46N;Interacts with:189F

AA2 48Q;Interacts with:206P,162D,207T,180G

AA2 49Q;Interacts with:187E,189F,190G

AA2 51Y;Interacts with:190G,175Y

AA2 52I;Interacts with:189F,190G

AA2 57F;Interacts with:208W

//

MESH1 mesh:D003093

NAME1 Colitis, Ulcerative

CLUSTER1 clust1

MESH2 mesh:D003424

NAME2 Crohn Disease

CLUSTER2 clust1

AC1 Q8N6P7

PFAM1 PF01108

PDB1 3DGC:R

AC2 Q9GZX6

PFAM2 PF14565

PDB2 3DGC:R

INT non-edgetic

AA1 58K;Interacts with:71D,70T

MUT1 K58A;882419;

AA1 60Y;Interacts with:72V,70T,169L,73R,69N,74L

MUT1 Y60A;1368880;

MUT1 Y60R;1368881;

AA1 61G;Interacts with:73R,72V,70T,71D

AA1 62E;Interacts with:72V,70T,71D

AA1 63R;Interacts with:71D

AA1 64D;Interacts with:71D

AA1 65W;Interacts with:70T

AA1 89T;Interacts with:77E,73R

AA1 90E;Interacts with:73R

AA1 91L;Interacts with:73R,169L

MUT1 L91F;dbSNP:rs144035143;

AA1 93Y;Interacts with:70T

AA2 169L;Interacts with:60Y,91L

AA2 69N;Interacts with:60Y

AA2 70T;Interacts with:58K,61G,60Y,62E,93Y,65W

AA2 71D;Interacts with:58K,61G,63R,64D,62E

AA2 72V;Interacts with:62E,60Y,61G

AA2 73R;Interacts with:61G,90E,89T,60Y,91L

MUT2 R73H;dbSNP:rs149366319;

AA2 74L;Interacts with:60Y

AA2 77E;Interacts with:89T

//

MESH1 mesh:D003093

NAME1 Colitis, Ulcerative

CLUSTER1 clust1

MESH2 mesh:D003424

NAME2 Crohn Disease

CLUSTER2 clust1

AC1 P13232

PFAM1 PF01415

PDB1 3DI2:A

AC2 P16871

PFAM2 PF00041

PDB2 3DI2:B

INT non-edgetic

AA1 103H;Interacts with:159Y

AA1 106K;Interacts with:159Y

AA1 35K;Interacts with:212Y

AA1 36Q;Interacts with:212Y

AA1 39S;Interacts with:211H,159Y,212Y,213F

AA1 40V;Interacts with:213F,212Y,159Y

AA1 43V;Interacts with:159Y,158K

AA1 44S;Interacts with:159Y

AA1 47Q;Interacts with:158K

AA2 158K;Interacts with:43V,47Q

AA2 159Y;Interacts with:39S,103H,43V,106K,40V,44S

AA2 211H;Interacts with:39S

AA2 212Y;Interacts with:40V,36Q,39S,35K

AA2 213F;Interacts with:40V,39S

//

MESH1 mesh:D003093

NAME1 Colitis, Ulcerative

CLUSTER1 clust1

MESH2 mesh:D003424

NAME2 Crohn Disease

CLUSTER2 clust1

AC1 P22301

PFAM1 PF00726

PDB1 1J7V:L

AC2 Q13651

PFAM2 PF09294

PDB2 1J7V:R

INT non-edgetic

AA1 38P;Interacts with:212R,211S,164F,210A

AA1 39N;Interacts with:210A

AA1 41L;Interacts with:211S

AA1 42R;Interacts with:211S,214N,208S,212R,166E,210A,213S

AA1 43D;Interacts with:211S

AA1 45R;Interacts with:211S

AA1 46D;Interacts with:213S

AA2 164F;Interacts with:38P

AA2 166E;Interacts with:42R

AA2 208S;Interacts with:42R

AA2 210A;Interacts with:39N,42R,38P

AA2 211S;Interacts with:42R,38P,41L,45R,43D

AA2 212R;Interacts with:38P,42R

AA2 213S;Interacts with:46D,42R

AA2 214N;Interacts with:42R

//

MESH1 mesh:D003093

NAME1 Colitis, Ulcerative

CLUSTER1 clust1

MESH2 mesh:D003424

NAME2 Crohn Disease

CLUSTER2 clust1

AC1 P22301

PFAM1 PF00726

PDB1 1J7V:L

AC2 Q13651

PFAM2 PF01108

PDB2 1J7V:R

INT non-edgetic

AA1 56Q;Interacts with:97R

AA1 59D;Interacts with:97R

AA1 60Q;Interacts with:97R

MUT1 Q60E;PMID:10637267;

AA1 61L;Interacts with:97R,64Y

AA1 62D;Interacts with:69W,66I,65G,67E,97R,64Y

AA1 63N;Interacts with:67E,65G,64Y

AA1 64L;Interacts with:64Y,67E,65G

AA1 65L;Interacts with:64Y

AA1 66L;Interacts with:94N

AA2 64Y;Interacts with:65L,64L,61L,62D,63N

AA2 65G;Interacts with:62D,63N,64L

AA2 66I;Interacts with:62D

AA2 67E;Interacts with:63N,62D,64L

AA2 69W;Interacts with:62D

AA2 94N;Interacts with:66L

AA2 97R;Interacts with:60Q,61L,59D,62D,56Q

//

MESH1 mesh:D003093

NAME1 Colitis, Ulcerative

CLUSTER1 clust1

MESH2 mesh:D003424

NAME2 Crohn Disease

CLUSTER2 clust1

AC1 P14778

PFAM1 PF13895

PDB1 4DEP:B

AC2 Q9NPH3

PFAM2 PF13895

PDB2 4DEP:C

INT non-edgetic

AA1 137D;Interacts with:152E,201I,154G,153Y

AA1 138G;Interacts with:201I,152E

AA1 139G;Interacts with:201I

AA1 149K;Interacts with:186N

AA1 171N;Interacts with:157R

AA1 177V;Interacts with:200L,191I,155I

AA1 178K;Interacts with:186N

AA1 180R;Interacts with:187F,188N

AA1 182I;Interacts with:201I,155I,200L,154G

AA1 184M;Interacts with:154G,155I

AA2 152E;Interacts with:137D,138G

AA2 153Y;Interacts with:137D

AA2 154G;Interacts with:184M,137D,182I

AA2 155I;Interacts with:182I,184M,177V

AA2 157R;Interacts with:171N

AA2 186N;Interacts with:178K,149K

AA2 187F;Interacts with:180R

AA2 188N;Interacts with:180R

AA2 191I;Interacts with:177V

AA2 200L;Interacts with:177V,182I

AA2 201I;Interacts with:138G,137D,182I,139G

//

MESH1 mesh:D003093

NAME1 Colitis, Ulcerative

CLUSTER1 clust1

MESH2 mesh:D003424

NAME2 Crohn Disease

CLUSTER2 clust1

AC1 P27930

PFAM1 PF13895

PDB1 3O4O:C

AC2 Q9NPH3

PFAM2 PF13895

PDB2 3O4O:C

INT non-edgetic

AA1 147S;Interacts with:152E,201I,154G,153Y

AA1 148G;Interacts with:201I

AA1 184L;Interacts with:155I

AA1 186V;Interacts with:191I,200L

AA1 187R;Interacts with:192P,193E

AA1 191H;Interacts with:188N,200L

AA1 193L;Interacts with:154G,155I,201I

AA1 195H;Interacts with:155I,154G

AA2 152E;Interacts with:147S

AA2 153Y;Interacts with:147S

AA2 154G;Interacts with:193L,147S,195H

AA2 155I;Interacts with:184L,195H,193L

AA2 188N;Interacts with:191H

AA2 191I;Interacts with:186V

AA2 192P;Interacts with:187R

AA2 193E;Interacts with:187R

AA2 200L;Interacts with:186V,191H

AA2 201I;Interacts with:148G,147S,193L

//

MESH1 mesh:D003093

NAME1 Colitis, Ulcerative

CLUSTER1 clust1

MESH2 mesh:D003424

NAME2 Crohn Disease

CLUSTER2 clust1

AC1 P15018

PFAM1 PF01291

PDB1 1PVH:B

AC2 P40189

PFAM2 PF09240

PDB2 1PVH:B

INT semi-edgetic

AA1 142D;Interacts with:189V,187S

AA1 143I;Interacts with:189V

AA1 145R;Interacts with:164W

AA1 146G;Interacts with:192V,164W,189V

AA1 149S;Interacts with:165A,166T

AA1 150N;Interacts with:191F,193N,192V

AA1 152L;Interacts with:166T

AA1 47Q;Interacts with:191F,192V,215D,193N

AA1 50S;Interacts with:191F

AA1 51Q;Interacts with:191F,192V

AA1 54Q;Interacts with:190Y

AA2 164W;Interacts with:146G,145R

AA2 165A;Interacts with:149S

AA2 166T;Interacts with:149S,152L

AA2 187S;Interacts with:142D

AA2 189V;Interacts with:143I,142D,146G

AA2 190Y;Interacts with:54Q

AA2 191F;Interacts with:150N,47Q,51Q,50S

AA2 192V;Interacts with:146G,47Q,51Q,150N

AA2 193N;Interacts with:150N,47Q

MUT2 N193delN;COSMIC:251360;

AA2 215D;Interacts with:47Q

//

MESH1 mesh:D003093

NAME1 Colitis, Ulcerative

CLUSTER1 clust1

MESH2 mesh:D003424

NAME2 Crohn Disease

CLUSTER2 clust1

AC1 P01563

PFAM1 PF00143

PDB1 2HYM:B

AC2 P48551

PFAM2 PF01108

PDB2 2HYM:A

INT semi-edgetic

AA1 145Y;Interacts with:75K

AA1 164E;Interacts with:73M

AA1 165V;Interacts with:73M

AA1 168A;Interacts with:73M,74S,105A

MUT1 A168T;dbSNP:rs143535431;

MUT1 A168G;PMID:11698684;

MUT1 A168M;PMID:10984492;

AA1 169E;Interacts with:73M,75K,74S

AA1 171M;Interacts with:73M

MUT1 M171A;PMID:10984492;PMID:11698684;

MUT1 M171I;dbSNP:rs77047588;

AA1 172R;Interacts with:104E,103H,74S,101S

AA1 175S;Interacts with:103H,104E

MUT1 S175P;dbSNP:rs138547087;

AA1 176L;Interacts with:103H

AA1 178T;Interacts with:103H

AA1 179N;Interacts with:103H,102T

AA1 182E;Interacts with:103H

AA1 185R;Interacts with:96T,99W,102T,100R,97D

AA1 186S;Interacts with:100R

AA1 48S;Interacts with:107V

MUT1 S48A;PMID:10984492;PMID:10556041;

AA1 49L;Interacts with:76P,73M,74S,107V,105A

MUT1 L49A;PMID:10984492;PMID:11698684,PMID:10556041;

AA1 50F;Interacts with:107V,79L,109V,71T,108T,70Y

AA1 52C;Interacts with:76P,77E

AA1 53L;Interacts with:79L,76P,77E,107V,71T,73M

MUT1 L53I;PMID:3081003;

MUT1 L53S;dbSNP:rs145655120;

AA1 54K;Interacts with:77E

AA1 55D;Interacts with:77E

MUT1 D55A;PMID:10984492;PMID:3081003,PMID:10556041;

AA1 56R;Interacts with:77E,75K,78D,76P,73M,71T,72I

MUT1 R56K;PMID:10984492;PMID:3081003;

AA1 57H;Interacts with:75K,77E,73M,74S

MUT1 H57R;883731;dbSNP:rs73420190;humsavar:VAR_013001;

AA1 58D;Interacts with:74S,75K,77E

AA1 59F;Interacts with:74S

MUT1 F59S;dbSNP:rs147918701;

AA2 100R;Interacts with:186S,185R

AA2 101S;Interacts with:172R

AA2 102T;Interacts with:185R,179N

AA2 103H;Interacts with:176L,175S,172R,178T,179N,182E

AA2 104E;Interacts with:172R,175S

AA2 105A;Interacts with:49L,168A

AA2 107V;Interacts with:50F,49L,48S,53L

AA2 108T;Interacts with:50F

AA2 109V;Interacts with:50F

MUT2 V109I;dbSNP:rs140084698;dbSNP:rs140084698;

AA2 70Y;Interacts with:50F

AA2 71T;Interacts with:50F,56R,53L

MUT2 T71A;PMID:11698684,PMID:9737924;

AA2 72I;Interacts with:56R

AA2 73M;Interacts with:49L,168A,169E,171M,56R,57H,164E,165V,53L

MUT2 M73A;PMID:9737924;

MUT2 M73V;dbSNP:rs142850110;dbSNP:rs142850110;

AA2 74S;Interacts with:58D,49L,172R,169E,168A,59F,57H

AA2 75K;Interacts with:58D,56R,169E,57H,145Y

MUT2 K75A;PMID:11698684,PMID:9737924;

AA2 76P;Interacts with:49L,53L,52C,56R

AA2 77E;Interacts with:56R,52C,55D,53L,54K,57H,58D

AA2 78D;Interacts with:56R

AA2 79L;Interacts with:53L,50F

AA2 96T;Interacts with:185R

AA2 97D;Interacts with:185R

AA2 99W;Interacts with:185R

MUT2 W99A;PMID:9737924;

//

MESH1 mesh:D003093

NAME1 Colitis, Ulcerative

CLUSTER1 clust1

MESH2 mesh:D003424

NAME2 Crohn Disease

CLUSTER2 clust1

AC1 P01584

PFAM1 PF00340

PDB1 4DEP:A

AC2 P14778

PFAM2 PF13895

PDB2 4DEP:B

INT semi-edgetic

AA1 147L;Interacts with:129K,128F,144Y

AA1 148Q;Interacts with:125Q,127I,126A,128F

MUT1 Q148G;PMID:7744786;

AA1 149G;Interacts with:127I,129K,125Q

AA1 150Q;Interacts with:127I,126A,125Q,124A

MUT1 Q150E;PMID:8073036;

AA1 152M;Interacts with:129K

AA1 224N;Interacts with:133P,134V

MUT1 N224A;PMID:1837145;

AA1 225K;Interacts with:137D

AA1 242Q;Interacts with:180R,178K

AA1 243A;Interacts with:146E

AA1 244E;Interacts with:146E,144Y,143P

AA1 245N;Interacts with:144Y

AA1 263T;Interacts with:132L,139G,138G

MUT1 T263G;PMID:7744786;PMID:7744786;

AA2 124A;Interacts with:150Q

MUT2 A124G;160853;humsavar:VAR_019131;rs2228139;

AA2 125Q;Interacts with:148Q,150Q,149G

MUT2 Q125K;dbSNP:rs76949245;

AA2 126A;Interacts with:150Q,148Q

AA2 127I;Interacts with:150Q,148Q,149G

AA2 128F;Interacts with:147L,148Q

AA2 129K;Interacts with:147L,149G,152M

AA2 132L;Interacts with:263T

AA2 133P;Interacts with:224N

AA2 134V;Interacts with:224N

MUT2 V134I;dbSNP:rs144901478;

AA2 137D;Interacts with:225K

AA2 138G;Interacts with:263T

AA2 139G;Interacts with:263T

AA2 143P;Interacts with:244E

AA2 144Y;Interacts with:244E,245N,147L

AA2 146E;Interacts with:244E,243A

AA2 178K;Interacts with:242Q

AA2 180R;Interacts with:242Q

//

MESH1 mesh:D003093

NAME1 Colitis, Ulcerative

CLUSTER1 clust1

MESH2 mesh:D003424

NAME2 Crohn Disease

CLUSTER2 clust1

AC1 P01584

PFAM1 PF00340

PDB1 1ITB:A

AC2 P14778

PFAM2 PF13927

PDB2 1ITB:B

INT semi-edgetic

AA1 160M;Interacts with:255S

MUT1 M160S;PMID:1837145;

AA1 162F;Interacts with:257I,255S,254L

AA1 164Q;Interacts with:278Y,276E,267I,268D,269E

MUT1 Q164G;PMID:7744786;

MUT1 Q164E;PMID:8073036;

AA1 166E;Interacts with:315K

AA1 167E;Interacts with:315K,261K,266V

AA1 168S;Interacts with:315K

AA1 169N;Interacts with:316N,315K

AA1 170D;Interacts with:316N

AA1 171K;Interacts with:315K,316N

AA1 172I;Interacts with:316N,257I,255S,256D,315K

AA1 208K;Interacts with:269E

MUT1 K208Q;PMID:7685764,PMID:8073036;

MUT1 K208R;PMID:7683661,PMID:8394358,PMID:7878046;

MUT1 K208M;PMID:8394358;

MUT1 K208S;PMID:7683661,PMID:7878046;

AA1 209K;Interacts with:269E,268D,267I,278Y,276E

MUT1 K209N;PMID:7685764;

MUT1 K209Q;PMID:7685764,PMID:7744786;

MUT1 K209M;PMID:7683661,PMID:8394358,PMID:7878046,PMID:7744786;

MUT1 K209A;PMID:1837145,PMID:1835838;

MUT1 K209L;PMID:1837145,PMID:7685764;

AA1 210K;Interacts with:267I,269E,268D,270D

MUT1 K210G;PMID:7683661,PMID:8394358;

MUT1 K210A;PMID:1837145,PMID:1835838;PMID:7744786;

AA1 219K;Interacts with:255S

MUT1 K219G;PMID:7744786;PMID:2138611,PMID:1909326,PMID:1386253,PMID:7744786;

MUT1 K219A;PMID:1386253,PMID:7744786;

MUT1 K219Q;PMID:8436117,PMID:8073036;

AA1 221E;Interacts with:253Q,255S,256D

MUT1 E221K;PMID:1532325,PMID:8073036;

AA1 224N;Interacts with:253Q,288R

AA1 264M;Interacts with:255S

MUT1 M264A;PMID:1837145;PMID:7744786;

AA2 253Q;Interacts with:221E,224N

MUT2 Q253K;dbSNP:rs75108697;

AA2 254L;Interacts with:162F

AA2 255S;Interacts with:264M,172I,160M,221E,219K,162F

AA2 256D;Interacts with:172I,221E

AA2 257I;Interacts with:172I,162F

AA2 261K;Interacts with:167E

AA2 266V;Interacts with:167E

AA2 267I;Interacts with:210K,209K,164Q

AA2 268D;Interacts with:209K,210K,164Q

AA2 269E;Interacts with:210K,208K,209K,164Q

AA2 270D;Interacts with:210K

AA2 276E;Interacts with:209K,164Q

AA2 278Y;Interacts with:164Q,209K

AA2 288R;Interacts with:224N

AA2 315K;Interacts with:171K,167E,172I,169N,168S,166E

MUT2 K315N;COSMIC:1210541;

AA2 316N;Interacts with:172I,169N,170D,171K

//

MESH1 mesh:D003093

NAME1 Colitis, Ulcerative

CLUSTER1 clust1

MESH2 mesh:D003424

NAME2 Crohn Disease

CLUSTER2 clust1

AC1 P01584

PFAM1 PF00340

PDB1 3O4O:A

AC2 Q9NPH3

PFAM2 PF13895

PDB2 3O4O:B

INT semi-edgetic

AA1 220I;Interacts with:204I

MUT1 I220G;PMID:7744786;

AA1 222I;Interacts with:204I,151I

AA1 223N;Interacts with:152E,151I

AA1 225K;Interacts with:152E

AA1 227E;Interacts with:205S,204I

MUT1 E227N;PMID:8073036;

AA1 242Q;Interacts with:187F,189N,188N,186N

AA1 253T;Interacts with:182Y

AA1 254K;Interacts with:204I,205S

MUT1 K254C;PMID:10852706;

AA1 255G;Interacts with:205S,188N,187F,179M,206N

AA1 256G;Interacts with:189N,187F,188N

AA1 257Q;Interacts with:187F,186N,185Q,188N

AA1 258D;Interacts with:188N

AA1 259I;Interacts with:188N

AA1 261D;Interacts with:204I,205S,188N

MUT1 D261K;PMID:1594572;

AA2 151I;Interacts with:222I,223N

AA2 152E;Interacts with:223N,225K

AA2 179M;Interacts with:255G

AA2 182Y;Interacts with:253T

AA2 185Q;Interacts with:257Q

AA2 186N;Interacts with:257Q,242Q

AA2 187F;Interacts with:242Q,257Q,255G,256G

AA2 188N;Interacts with:258D,255G,242Q,257Q,256G,261D,259I

AA2 189N;Interacts with:242Q,256G

AA2 204I;Interacts with:261D,222I,254K,227E,220I

AA2 205S;Interacts with:227E,255G,261D,254K

AA2 206N;Interacts with:255G

//

MESH1 mesh:D003093

NAME1 Colitis, Ulcerative

CLUSTER1 clust1

MESH2 mesh:D003424

NAME2 Crohn Disease

CLUSTER2 clust1

AC1 P01584

PFAM1 PF00340

PDB1 3O4O:A

AC2 P27930

PFAM2 PF00047

PDB2 3O4O:C

INT semi-edgetic

AA1 162F;Interacts with:271M

MUT1 F162D;PMID:1837145;

AA1 164Q;Interacts with:280H,273W

MUT1 Q164G;PMID:7744786;

MUT1 Q164E;PMID:8073036;

AA1 167E;Interacts with:280H

AA1 209K;Interacts with:282E,292E

MUT1 K209N;PMID:7685764;

MUT1 K209Q;PMID:7685764,PMID:7744786;

MUT1 K209M;PMID:7683661,PMID:8394358,PMID:7878046,PMID:7744786;

MUT1 K209A;PMID:1837145,PMID:1835838;

MUT1 K209L;PMID:1837145,PMID:7685764;

MUT1 K209R;PMID:7683661,PMID:8394358,PMID:7878046;

AA1 210K;Interacts with:282E,280H

MUT1 K210G;PMID:7683661,PMID:8394358;

MUT1 K210A;PMID:1837145,PMID:1835838;PMID:7744786;

AA1 219K;Interacts with:269T

MUT1 K219G;PMID:7744786;PMID:2138611,PMID:1909326,PMID:1386253,PMID:7744786;

MUT1 K219A;PMID:1386253,PMID:7744786;

MUT1 K219Q;PMID:8436117,PMID:8073036;

AA2 269T;Interacts with:219K

AA2 271M;Interacts with:162F

AA2 273W;Interacts with:164Q

AA2 280H;Interacts with:164Q,210K,167E

AA2 282E;Interacts with:209K,210K

AA2 292E;Interacts with:209K

MUT2 E292K;214598;humsavar:VAR_019133;rs3218976;

//

MESH1 mesh:D003093

NAME1 Colitis, Ulcerative

CLUSTER1 clust1

MESH2 mesh:D003424

NAME2 Crohn Disease

CLUSTER2 clust1

AC1 P08887

PFAM1 PF09240

PDB1 1P9M:C

AC2 P05231

PFAM2 PF00489

PDB2 1P9M:C

INT semi-edgetic

AA1 155N;Interacts with:89N,94K

AA1 182E;Interacts with:97E,102F,207R

AA1 183G;Interacts with:102F

AA1 185S;Interacts with:94K

AA1 187F;Interacts with:82K

AA1 209Q;Interacts with:82K

AA1 211C;Interacts with:82K

MUT1 C211A;884560;

AA1 212G;Interacts with:82K

AA2 102F;Interacts with:183G,182E

AA2 207R;Interacts with:182E

MUT2 R207S;PMID:8436132,PMID:8483922;

MUT2 R207A;PMID:8436132,PMID:8483922;

MUT2 R207P;PMID:8436132,PMID:8483922;

MUT2 R207G;PMID:8436132,PMID:8483922;

MUT2 R207H;PMID:8436132;

MUT2 R207K;PMID:1321818,PMID:8436132,PMID:8483922;

MUT2 R207N;PMID:8436132,PMID:8483922;

MUT2 R207W;PMID:8436132;

AA2 82K;Interacts with:212G,209Q,211C,187F

AA2 89N;Interacts with:155N

AA2 94K;Interacts with:185S,155N

AA2 97E;Interacts with:182E

//

MESH1 mesh:D003093

NAME1 Colitis, Ulcerative

CLUSTER1 clust1

MESH2 mesh:D013274

NAME2 Stomach Neoplasms

CLUSTER2 clust1

AC1 P29459

PFAM1 PF03039

PDB1 3HMX:B

AC2 P29460

PFAM2 PF10420

PDB2 3HMX:A

INT non-edgetic

AA1 205R;Interacts with:203E

AA1 208T;Interacts with:203E,202A

AA1 211R;Interacts with:136Y

AA1 71H;Interacts with:205S

AA1 76K;Interacts with:205S

AA1 82V;Interacts with:201A,202A,203E

AA1 83E;Interacts with:201A,202A

AA1 85C;Interacts with:202A

AA1 86L;Interacts with:202A,200P,201A

AA2 136Y;Interacts with:211R

AA2 200P;Interacts with:86L

AA2 201A;Interacts with:82V,83E,86L

AA2 202A;Interacts with:85C,82V,86L,83E,208T

AA2 203E;Interacts with:208T,82V,205R

AA2 205S;Interacts with:71H,76K

//

MESH1 mesh:D003093

NAME1 Colitis, Ulcerative

CLUSTER1 clust1

MESH2 mesh:D013274

NAME2 Stomach Neoplasms

CLUSTER2 clust1

AC1 P14778

PFAM1 PF13895

PDB1 1IRA:Y

AC2 P18510

PFAM2 PF00340

PDB2 1IRA:Y

INT non-edgetic

AA1 125Q;Interacts with:63P,62G,61Q

MUT1 Q125K;dbSNP:rs76949245;

AA1 126A;Interacts with:61Q

AA1 127I;Interacts with:61Q,62G

AA1 128F;Interacts with:60L,61Q

AA1 129K;Interacts with:60L

AA1 131K;Interacts with:68E

AA1 132L;Interacts with:172Y

AA1 139G;Interacts with:172Y

AA1 144Y;Interacts with:152A,60L

AA1 146E;Interacts with:152A,151E

AA2 151E;Interacts with:146E

MUT2 E151G;PMID:7744786;

AA2 152A;Interacts with:146E,144Y

AA2 172Y;Interacts with:139G,132L

MUT2 Y172T;PMID:7744786;

MUT2 Y172M;PMID:7744786;

MUT2 Y172G;PMID:7744786;

MUT2 Y172K;PMID:7744786;

MUT2 Y172H;PMID:7744786;

AA2 60L;Interacts with:128F,129K,144Y

AA2 61Q;Interacts with:127I,126A,125Q,128F

MUT2 Q61G;PMID:7744786;

MUT2 Q61F;PMID:7744786;

AA2 62G;Interacts with:125Q,127I

AA2 63P;Interacts with:125Q

MUT2 P63G;PMID:7744786;

AA2 68E;Interacts with:131K

MUT2 E68G;PMID:7744786;

//

MESH1 mesh:D003093

NAME1 Colitis, Ulcerative

CLUSTER1 clust1

MESH2 mesh:D013274

NAME2 Stomach Neoplasms

CLUSTER2 clust1

AC1 P01584

PFAM1 PF00340

PDB1 4DEP:A

AC2 P14778

PFAM2 PF13895

PDB2 4DEP:B

INT non-edgetic

AA1 147L;Interacts with:129K,128F,144Y

AA1 148Q;Interacts with:125Q,127I,126A,128F

MUT1 Q148G;PMID:7744786;

AA1 149G;Interacts with:127I,129K,125Q

AA1 150Q;Interacts with:127I,126A,125Q,124A

MUT1 Q150E;PMID:8073036;

AA1 152M;Interacts with:129K

AA1 224N;Interacts with:133P,134V

MUT1 N224A;PMID:1837145;

AA1 225K;Interacts with:137D

AA1 242Q;Interacts with:180R,178K

AA1 243A;Interacts with:146E

AA1 244E;Interacts with:146E,144Y,143P

AA1 245N;Interacts with:144Y

AA1 263T;Interacts with:132L,139G,138G

MUT1 T263G;PMID:7744786;PMID:7744786;

AA2 124A;Interacts with:150Q

MUT2 A124G;160853;humsavar:VAR_019131;rs2228139;

AA2 125Q;Interacts with:148Q,150Q,149G

MUT2 Q125K;dbSNP:rs76949245;

AA2 126A;Interacts with:150Q,148Q

AA2 127I;Interacts with:150Q,148Q,149G

AA2 128F;Interacts with:147L,148Q

AA2 129K;Interacts with:147L,149G,152M

AA2 132L;Interacts with:263T

AA2 133P;Interacts with:224N

AA2 134V;Interacts with:224N

MUT2 V134I;dbSNP:rs144901478;

AA2 137D;Interacts with:225K

AA2 138G;Interacts with:263T

AA2 139G;Interacts with:263T

AA2 143P;Interacts with:244E

AA2 144Y;Interacts with:244E,245N,147L

AA2 146E;Interacts with:244E,243A

AA2 178K;Interacts with:242Q

AA2 180R;Interacts with:242Q

//

MESH1 mesh:D003093

NAME1 Colitis, Ulcerative

CLUSTER1 clust1

MESH2 mesh:D013274

NAME2 Stomach Neoplasms

CLUSTER2 clust1

AC1 P01584

PFAM1 PF00340

PDB1 1ITB:A

AC2 P14778

PFAM2 PF13927

PDB2 1ITB:B

INT non-edgetic

AA1 160M;Interacts with:255S

MUT1 M160S;PMID:1837145;

AA1 162F;Interacts with:257I,255S,254L

AA1 164Q;Interacts with:278Y,276E,267I,268D,269E

MUT1 Q164G;PMID:7744786;

MUT1 Q164E;PMID:8073036;

AA1 166E;Interacts with:315K

AA1 167E;Interacts with:315K,261K,266V

AA1 168S;Interacts with:315K

AA1 169N;Interacts with:316N,315K

AA1 170D;Interacts with:316N

AA1 171K;Interacts with:315K,316N

AA1 172I;Interacts with:316N,257I,255S,256D,315K

AA1 208K;Interacts with:269E

MUT1 K208Q;PMID:7685764,PMID:8073036;

MUT1 K208R;PMID:7683661,PMID:8394358,PMID:7878046;

MUT1 K208M;PMID:8394358;

MUT1 K208S;PMID:7683661,PMID:7878046;

AA1 209K;Interacts with:269E,268D,267I,278Y,276E

MUT1 K209N;PMID:7685764;

MUT1 K209Q;PMID:7685764,PMID:7744786;

MUT1 K209M;PMID:7683661,PMID:8394358,PMID:7878046,PMID:7744786;

MUT1 K209A;PMID:1837145,PMID:1835838;

MUT1 K209L;PMID:1837145,PMID:7685764;

AA1 210K;Interacts with:267I,269E,268D,270D

MUT1 K210G;PMID:7683661,PMID:8394358;

MUT1 K210A;PMID:1837145,PMID:1835838;PMID:7744786;

AA1 219K;Interacts with:255S

MUT1 K219G;PMID:7744786;PMID:2138611,PMID:1909326,PMID:1386253,PMID:7744786;

MUT1 K219A;PMID:1386253,PMID:7744786;

MUT1 K219Q;PMID:8436117,PMID:8073036;

AA1 221E;Interacts with:253Q,255S,256D

MUT1 E221K;PMID:1532325,PMID:8073036;

AA1 224N;Interacts with:253Q,288R

AA1 264M;Interacts with:255S

MUT1 M264A;PMID:1837145;PMID:7744786;

AA2 253Q;Interacts with:221E,224N

MUT2 Q253K;dbSNP:rs75108697;

AA2 254L;Interacts with:162F

AA2 255S;Interacts with:264M,172I,160M,221E,219K,162F

AA2 256D;Interacts with:172I,221E

AA2 257I;Interacts with:172I,162F

AA2 261K;Interacts with:167E

AA2 266V;Interacts with:167E

AA2 267I;Interacts with:210K,209K,164Q

AA2 268D;Interacts with:209K,210K,164Q

AA2 269E;Interacts with:210K,208K,209K,164Q

AA2 270D;Interacts with:210K

AA2 276E;Interacts with:209K,164Q

AA2 278Y;Interacts with:164Q,209K

AA2 288R;Interacts with:224N

AA2 315K;Interacts with:171K,167E,172I,169N,168S,166E

MUT2 K315N;COSMIC:1210541;

AA2 316N;Interacts with:172I,169N,170D,171K

//

MESH1 mesh:D003093

NAME1 Colitis, Ulcerative

CLUSTER1 clust1

MESH2 mesh:D013274

NAME2 Stomach Neoplasms

CLUSTER2 clust1

AC1 P01584

PFAM1 PF00340

PDB1 3O4O:A

AC2 P27930

PFAM2 PF00047

PDB2 3O4O:C

INT non-edgetic

AA1 162F;Interacts with:271M

MUT1 F162D;PMID:1837145;

AA1 164Q;Interacts with:280H,273W

MUT1 Q164G;PMID:7744786;

MUT1 Q164E;PMID:8073036;

AA1 167E;Interacts with:280H

AA1 209K;Interacts with:282E,292E

MUT1 K209N;PMID:7685764;

MUT1 K209Q;PMID:7685764,PMID:7744786;

MUT1 K209M;PMID:7683661,PMID:8394358,PMID:7878046,PMID:7744786;

MUT1 K209A;PMID:1837145,PMID:1835838;

MUT1 K209L;PMID:1837145,PMID:7685764;

MUT1 K209R;PMID:7683661,PMID:8394358,PMID:7878046;

AA1 210K;Interacts with:282E,280H

MUT1 K210G;PMID:7683661,PMID:8394358;

MUT1 K210A;PMID:1837145,PMID:1835838;PMID:7744786;

AA1 219K;Interacts with:269T

MUT1 K219G;PMID:7744786;PMID:2138611,PMID:1909326,PMID:1386253,PMID:7744786;

MUT1 K219A;PMID:1386253,PMID:7744786;

MUT1 K219Q;PMID:8436117,PMID:8073036;

AA2 269T;Interacts with:219K

AA2 271M;Interacts with:162F

AA2 273W;Interacts with:164Q

AA2 280H;Interacts with:164Q,210K,167E

AA2 282E;Interacts with:209K,210K

AA2 292E;Interacts with:209K

MUT2 E292K;214598;humsavar:VAR_019133;rs3218976;

//

MESH1 mesh:D003093

NAME1 Colitis, Ulcerative

CLUSTER1 clust1

MESH2 mesh:D013274

NAME2 Stomach Neoplasms

CLUSTER2 clust1

AC1 P01584

PFAM1 PF00340

PDB1 3O4O:A

AC2 Q9NPH3

PFAM2 PF13895

PDB2 3O4O:B

INT semi-edgetic

AA1 220I;Interacts with:204I

MUT1 I220G;PMID:7744786;

AA1 222I;Interacts with:204I,151I

AA1 223N;Interacts with:152E,151I

AA1 225K;Interacts with:152E

AA1 227E;Interacts with:205S,204I

MUT1 E227N;PMID:8073036;

AA1 242Q;Interacts with:187F,189N,188N,186N

AA1 253T;Interacts with:182Y

AA1 254K;Interacts with:204I,205S

MUT1 K254C;PMID:10852706;

AA1 255G;Interacts with:205S,188N,187F,179M,206N

AA1 256G;Interacts with:189N,187F,188N

AA1 257Q;Interacts with:187F,186N,185Q,188N

AA1 258D;Interacts with:188N

AA1 259I;Interacts with:188N

AA1 261D;Interacts with:204I,205S,188N

MUT1 D261K;PMID:1594572;

AA2 151I;Interacts with:222I,223N

AA2 152E;Interacts with:223N,225K

AA2 179M;Interacts with:255G

AA2 182Y;Interacts with:253T

AA2 185Q;Interacts with:257Q

AA2 186N;Interacts with:257Q,242Q

AA2 187F;Interacts with:242Q,257Q,255G,256G

AA2 188N;Interacts with:258D,255G,242Q,257Q,256G,261D,259I

AA2 189N;Interacts with:242Q,256G

AA2 204I;Interacts with:261D,222I,254K,227E,220I

AA2 205S;Interacts with:227E,255G,261D,254K

AA2 206N;Interacts with:255G

//

MESH1 mesh:D003093

NAME1 Colitis, Ulcerative

CLUSTER1 clust1

MESH2 mesh:D013274

NAME2 Stomach Neoplasms

CLUSTER2 clust1

AC1 P14778

PFAM1 PF13895

PDB1 4DEP:B

AC2 Q9NPH3

PFAM2 PF13895

PDB2 4DEP:C

INT semi-edgetic

AA1 137D;Interacts with:152E,201I,154G,153Y

AA1 138G;Interacts with:201I,152E

AA1 139G;Interacts with:201I

AA1 149K;Interacts with:186N

AA1 171N;Interacts with:157R

AA1 177V;Interacts with:200L,191I,155I

AA1 178K;Interacts with:186N

AA1 180R;Interacts with:187F,188N

AA1 182I;Interacts with:201I,155I,200L,154G

AA1 184M;Interacts with:154G,155I

AA2 152E;Interacts with:137D,138G

AA2 153Y;Interacts with:137D

AA2 154G;Interacts with:184M,137D,182I

AA2 155I;Interacts with:182I,184M,177V

AA2 157R;Interacts with:171N

AA2 186N;Interacts with:178K,149K

AA2 187F;Interacts with:180R

AA2 188N;Interacts with:180R

AA2 191I;Interacts with:177V

AA2 200L;Interacts with:177V,182I

AA2 201I;Interacts with:138G,137D,182I,139G

//

MESH1 mesh:D003093

NAME1 Colitis, Ulcerative

CLUSTER1 clust1

MESH2 mesh:D013274

NAME2 Stomach Neoplasms

CLUSTER2 clust1

AC1 P27930

PFAM1 PF13895

PDB1 3O4O:C

AC2 Q9NPH3

PFAM2 PF13895

PDB2 3O4O:C

INT semi-edgetic

AA1 147S;Interacts with:152E,201I,154G,153Y

AA1 148G;Interacts with:201I

AA1 184L;Interacts with:155I

AA1 186V;Interacts with:191I,200L

AA1 187R;Interacts with:192P,193E

AA1 191H;Interacts with:188N,200L

AA1 193L;Interacts with:154G,155I,201I

AA1 195H;Interacts with:155I,154G

AA2 152E;Interacts with:147S

AA2 153Y;Interacts with:147S

AA2 154G;Interacts with:193L,147S,195H

AA2 155I;Interacts with:184L,195H,193L

AA2 188N;Interacts with:191H

AA2 191I;Interacts with:186V

AA2 192P;Interacts with:187R

AA2 193E;Interacts with:187R

AA2 200L;Interacts with:186V,191H

AA2 201I;Interacts with:148G,147S,193L

//

MESH1 mesh:D003093

NAME1 Colitis, Ulcerative

CLUSTER1 clust1

MESH2 mesh:D013274

NAME2 Stomach Neoplasms

CLUSTER2 clust1

AC1 P15260

PFAM1 PF07140

PDB1 1FYH:E

AC2 P01579

PFAM2 PF00714

PDB2 1FYH:E

INT edgetic

AA1 183I;Interacts with:147A

AA1 185Y;Interacts with:146A,147A,149T

AA1 186K;Interacts with:146A

AA1 203A;Interacts with:146A

AA1 205P;Interacts with:150G,146A

AA1 207S;Interacts with:151K,150G

AA1 208S;Interacts with:150G,151K

AA1 209L;Interacts with:151K

AA2 146A;Interacts with:186K,203A,205P,185Y

AA2 147A;Interacts with:183I,185Y

MUT2 A147L;PMID:1662603;

AA2 149T;Interacts with:185Y

AA2 150G;Interacts with:208S,205P,207S

AA2 151K;Interacts with:209L,207S,208S

MUT2 K151R;PMID:2127102;

MUT2 K151E;PMID:1830392;

//

MESH1 mesh:D003093

NAME1 Colitis, Ulcerative

CLUSTER1 clust1

MESH2 mesh:D013274

NAME2 Stomach Neoplasms

CLUSTER2 clust1

AC1 P15260

PFAM1 PF01108

PDB1 1FYH:B

AC2 P01579

PFAM2 PF00714

PDB2 1FYH:B

INT edgetic

AA1 66Y;Interacts with:163R

MUT1 Y66A;PMID:9878445;

AA2 163R;Interacts with:66Y

//

MESH1 mesh:D003093

NAME1 Colitis, Ulcerative

CLUSTER1 clust1

MESH2 mesh:D015451

NAME2 Leukemia, B-cell, Chronic

CLUSTER2 clust1

AC1 P08887

PFAM1 PF09240

PDB1 1P9M:C

AC2 P05231

PFAM2 PF00489

PDB2 1P9M:C

INT edgetic

AA1 155N;Interacts with:89N,94K

AA1 182E;Interacts with:97E,102F,207R

AA1 183G;Interacts with:102F

AA1 185S;Interacts with:94K

AA1 187F;Interacts with:82K

AA1 209Q;Interacts with:82K

AA1 211C;Interacts with:82K

MUT1 C211A;884560;

AA1 212G;Interacts with:82K

AA2 102F;Interacts with:183G,182E

AA2 207R;Interacts with:182E

MUT2 R207S;PMID:8436132,PMID:8483922;

MUT2 R207A;PMID:8436132,PMID:8483922;

MUT2 R207P;PMID:8436132,PMID:8483922;

MUT2 R207G;PMID:8436132,PMID:8483922;

MUT2 R207H;PMID:8436132;

MUT2 R207K;PMID:1321818,PMID:8436132,PMID:8483922;

MUT2 R207N;PMID:8436132,PMID:8483922;

MUT2 R207W;PMID:8436132;

AA2 82K;Interacts with:212G,209Q,211C,187F

AA2 89N;Interacts with:155N

AA2 94K;Interacts with:185S,155N

AA2 97E;Interacts with:182E

//

MESH1 mesh:D003093

NAME1 Colitis, Ulcerative

CLUSTER1 clust1

MESH2 mesh:D015663

NAME2 Osteoporosis, Postmenopausal

CLUSTER2 clust1

AC1 P14778

PFAM1 PF13895

PDB1 1IRA:Y

AC2 P18510

PFAM2 PF00340

PDB2 1IRA:Y

INT non-edgetic

AA1 125Q;Interacts with:63P,62G,61Q

MUT1 Q125K;dbSNP:rs76949245;

AA1 126A;Interacts with:61Q

AA1 127I;Interacts with:61Q,62G

AA1 128F;Interacts with:60L,61Q

AA1 129K;Interacts with:60L

AA1 131K;Interacts with:68E

AA1 132L;Interacts with:172Y

AA1 139G;Interacts with:172Y

AA1 144Y;Interacts with:152A,60L

AA1 146E;Interacts with:152A,151E

AA2 151E;Interacts with:146E

MUT2 E151G;PMID:7744786;

AA2 152A;Interacts with:146E,144Y

AA2 172Y;Interacts with:139G,132L

MUT2 Y172T;PMID:7744786;

MUT2 Y172M;PMID:7744786;

MUT2 Y172G;PMID:7744786;

MUT2 Y172K;PMID:7744786;

MUT2 Y172H;PMID:7744786;

AA2 60L;Interacts with:128F,129K,144Y

AA2 61Q;Interacts with:127I,126A,125Q,128F

MUT2 Q61G;PMID:7744786;

MUT2 Q61F;PMID:7744786;

AA2 62G;Interacts with:125Q,127I

AA2 63P;Interacts with:125Q

MUT2 P63G;PMID:7744786;

AA2 68E;Interacts with:131K

MUT2 E68G;PMID:7744786;

//

MESH1 mesh:D003093

NAME1 Colitis, Ulcerative

CLUSTER1 clust1

MESH2 mesh:D015663

NAME2 Osteoporosis, Postmenopausal

CLUSTER2 clust1

AC1 P01584

PFAM1 PF00340

PDB1 4DEP:A

AC2 P14778

PFAM2 PF13895

PDB2 4DEP:B

INT non-edgetic

AA1 147L;Interacts with:129K,128F,144Y

AA1 148Q;Interacts with:125Q,127I,126A,128F

MUT1 Q148G;PMID:7744786;

AA1 149G;Interacts with:127I,129K,125Q

AA1 150Q;Interacts with:127I,126A,125Q,124A

MUT1 Q150E;PMID:8073036;

AA1 152M;Interacts with:129K

AA1 224N;Interacts with:133P,134V

MUT1 N224A;PMID:1837145;

AA1 225K;Interacts with:137D

AA1 242Q;Interacts with:180R,178K

AA1 243A;Interacts with:146E

AA1 244E;Interacts with:146E,144Y,143P

AA1 245N;Interacts with:144Y

AA1 263T;Interacts with:132L,139G,138G

MUT1 T263G;PMID:7744786;PMID:7744786;

AA2 124A;Interacts with:150Q

MUT2 A124G;160853;humsavar:VAR_019131;rs2228139;

AA2 125Q;Interacts with:148Q,150Q,149G

MUT2 Q125K;dbSNP:rs76949245;

AA2 126A;Interacts with:150Q,148Q

AA2 127I;Interacts with:150Q,148Q,149G

AA2 128F;Interacts with:147L,148Q

AA2 129K;Interacts with:147L,149G,152M

AA2 132L;Interacts with:263T

AA2 133P;Interacts with:224N

AA2 134V;Interacts with:224N

MUT2 V134I;dbSNP:rs144901478;

AA2 137D;Interacts with:225K

AA2 138G;Interacts with:263T

AA2 139G;Interacts with:263T

AA2 143P;Interacts with:244E

AA2 144Y;Interacts with:244E,245N,147L

AA2 146E;Interacts with:244E,243A

AA2 178K;Interacts with:242Q

AA2 180R;Interacts with:242Q

//

MESH1 mesh:D003093

NAME1 Colitis, Ulcerative

CLUSTER1 clust1

MESH2 mesh:D015663

NAME2 Osteoporosis, Postmenopausal

CLUSTER2 clust1

AC1 P01584

PFAM1 PF00340

PDB1 1ITB:A

AC2 P14778

PFAM2 PF13927

PDB2 1ITB:B

INT non-edgetic

AA1 160M;Interacts with:255S

MUT1 M160S;PMID:1837145;

AA1 162F;Interacts with:257I,255S,254L

AA1 164Q;Interacts with:278Y,276E,267I,268D,269E

MUT1 Q164G;PMID:7744786;

MUT1 Q164E;PMID:8073036;

AA1 166E;Interacts with:315K

AA1 167E;Interacts with:315K,261K,266V

AA1 168S;Interacts with:315K

AA1 169N;Interacts with:316N,315K

AA1 170D;Interacts with:316N

AA1 171K;Interacts with:315K,316N

AA1 172I;Interacts with:316N,257I,255S,256D,315K

AA1 208K;Interacts with:269E

MUT1 K208Q;PMID:7685764,PMID:8073036;

MUT1 K208R;PMID:7683661,PMID:8394358,PMID:7878046;

MUT1 K208M;PMID:8394358;

MUT1 K208S;PMID:7683661,PMID:7878046;

AA1 209K;Interacts with:269E,268D,267I,278Y,276E

MUT1 K209N;PMID:7685764;

MUT1 K209Q;PMID:7685764,PMID:7744786;

MUT1 K209M;PMID:7683661,PMID:8394358,PMID:7878046,PMID:7744786;

MUT1 K209A;PMID:1837145,PMID:1835838;

MUT1 K209L;PMID:1837145,PMID:7685764;

AA1 210K;Interacts with:267I,269E,268D,270D

MUT1 K210G;PMID:7683661,PMID:8394358;

MUT1 K210A;PMID:1837145,PMID:1835838;PMID:7744786;

AA1 219K;Interacts with:255S

MUT1 K219G;PMID:7744786;PMID:2138611,PMID:1909326,PMID:1386253,PMID:7744786;

MUT1 K219A;PMID:1386253,PMID:7744786;

MUT1 K219Q;PMID:8436117,PMID:8073036;

AA1 221E;Interacts with:253Q,255S,256D

MUT1 E221K;PMID:1532325,PMID:8073036;

AA1 224N;Interacts with:253Q,288R

AA1 264M;Interacts with:255S

MUT1 M264A;PMID:1837145;PMID:7744786;

AA2 253Q;Interacts with:221E,224N

MUT2 Q253K;dbSNP:rs75108697;

AA2 254L;Interacts with:162F

AA2 255S;Interacts with:264M,172I,160M,221E,219K,162F

AA2 256D;Interacts with:172I,221E

AA2 257I;Interacts with:172I,162F

AA2 261K;Interacts with:167E

AA2 266V;Interacts with:167E

AA2 267I;Interacts with:210K,209K,164Q

AA2 268D;Interacts with:209K,210K,164Q

AA2 269E;Interacts with:210K,208K,209K,164Q

AA2 270D;Interacts with:210K

AA2 276E;Interacts with:209K,164Q

AA2 278Y;Interacts with:164Q,209K

AA2 288R;Interacts with:224N

AA2 315K;Interacts with:171K,167E,172I,169N,168S,166E

MUT2 K315N;COSMIC:1210541;

AA2 316N;Interacts with:172I,169N,170D,171K

//

MESH1 mesh:D003093

NAME1 Colitis, Ulcerative

CLUSTER1 clust1

MESH2 mesh:D015663

NAME2 Osteoporosis, Postmenopausal

CLUSTER2 clust1

AC1 P01584

PFAM1 PF00340

PDB1 3O4O:A

AC2 Q9NPH3

PFAM2 PF13895

PDB2 3O4O:B

INT non-edgetic

AA1 220I;Interacts with:204I

MUT1 I220G;PMID:7744786;

AA1 222I;Interacts with:204I,151I

AA1 223N;Interacts with:152E,151I

AA1 225K;Interacts with:152E

AA1 227E;Interacts with:205S,204I

MUT1 E227N;PMID:8073036;

AA1 242Q;Interacts with:187F,189N,188N,186N

AA1 253T;Interacts with:182Y

AA1 254K;Interacts with:204I,205S

MUT1 K254C;PMID:10852706;

AA1 255G;Interacts with:205S,188N,187F,179M,206N

AA1 256G;Interacts with:189N,187F,188N

AA1 257Q;Interacts with:187F,186N,185Q,188N

AA1 258D;Interacts with:188N

AA1 259I;Interacts with:188N

AA1 261D;Interacts with:204I,205S,188N

MUT1 D261K;PMID:1594572;

AA2 151I;Interacts with:222I,223N

AA2 152E;Interacts with:223N,225K

AA2 179M;Interacts with:255G

AA2 182Y;Interacts with:253T

AA2 185Q;Interacts with:257Q

AA2 186N;Interacts with:257Q,242Q
[truncated: 3,668,675 more chars]
